# Visible Light-Driven, Gold(I)-Catalyzed Preparation of Symmetrical (Hetero)biaryls by Homocoupling of Arylazo Sulfones

**DOI:** 10.1021/acs.joc.2c00225

**Published:** 2022-03-22

**Authors:** Lorenzo Di Terlizzi, Simone Scaringi, Carlotta Raviola, Riccardo Pedrazzani, Marco Bandini, Maurizio Fagnoni, Stefano Protti

**Affiliations:** † PhotoGreen Lab, Department of Chemistry, University of Pavia, Viale Taramelli 12, 27100 Pavia, Italy; ‡ Department of Organic Chemistry, University of Geneva, 30 quai Ernest Ansermet, 1211 Geneva, Switzerland; § Dipartimento di Chimica ″Giacomo Ciamician″, Alma Mater Studiorum-University of Bologna, Via Selmi 2, 40126 Bologna, Italy

## Abstract

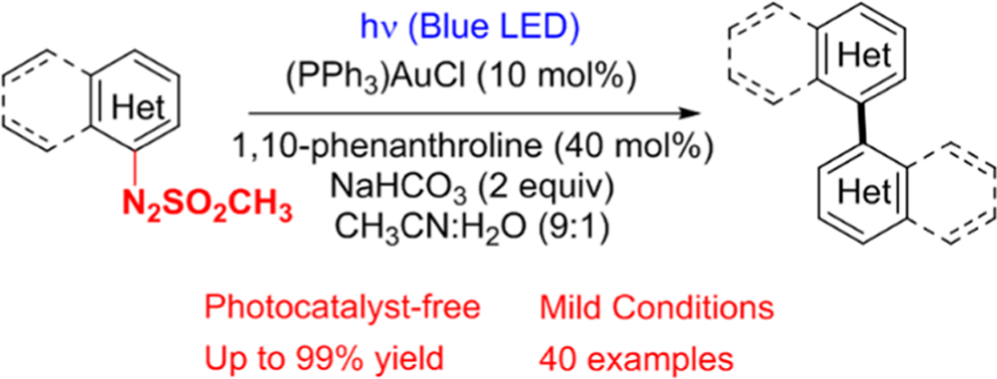

The preparation of symmetrical (hetero)biaryls via arylazo sulfones has been successfully carried out upon visible light irradiation in the presence of PPh_3_AuCl as the catalyst. The present protocol led to the efficient synthesis of a wide range of target compounds in an organic-aqueous solvent under photocatalyst-free conditions.

## Introduction

**Scheme 1:**
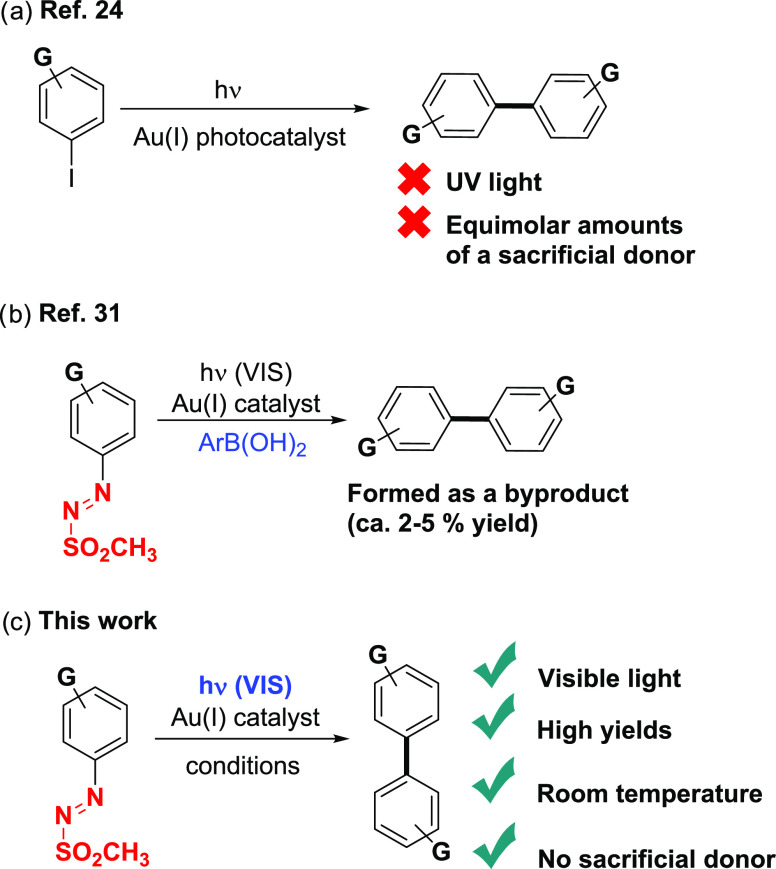
Photoinduced Homocoupling for the Synthesis of Biaryls (a) [Au(I)]-catalyzed homocoupling of aryl iodides; (b) trace amount of biaryl byproducts from the [Au(I)] gold-catalyzed Suzuki-type coupling via arylazo sulfones; and (c) our proposal.

The symmetrical biaryl scaffold is a ubiquitous chemical motive in naturally occurring products^1^ as well as artificial bioactive species.^2^ In addition, the current growing interest for these biaryls is ascribable to their multifaced applications as (stereogenic) ligands,^3^ conducting and electroluminescent materials,^4^ and key constituents of molecular switches and devices.^5^ Thus, it is not surprising that starting from the early synthetic approaches based on the copper-catalyzed coupling of aryl halides under reductive conditions (the so-called Ullmann reaction),^6^ an impressive number of transition metal-catalyzed protocols was developed.^7−12^ In this context, the use of Au salts, complexes, or nanoparticles as the catalysts for homocoupling processes has been widely explored, and different substrates have been successfully employed, including, among the others, aryl boronic acids (in the presence of a stoichiometric amount of an inorganic base),^13^ haloarenes (mainly iodides, by following an Ullman-type coupling),^14^ and aromatics (via direct C–H bond functionalization under oxidative conditions).^15^ On the other hand, the opportunity to perform efficient and selective syntheses under mild conditions upon visible light irradiation allowed for the emergence of photochemistry as a sustainable synthetic approach. Currently, two strategies are mainly exploited to achieve these targets, namely, the use of a colored photocatalyst able to activate the substrates via electron^16^ or atom (hydrogen^17^ or halogen^18^) transfer and the use of commercially available or properly designed organic molecules that absorb in the visible region.^19^ However, although the synthesis of asymmetric biphenyls under either photochemical^20^ or photocatalyzed^21^ conditions has been largely documented, the related photoinduced homocoupling processes are still largely underdeveloped.^22^ These include the photocatalyzed Ullmann reaction of aryl halides using KNb_3_O_8_@AuNP,^23^ the [Au(I)]-photoredox-catalyzed coupling of aryl iodides developed by Barriault and co-workers^24^ (Scheme 1a) or the dual photoredox/nickel-catalyzed dimerization of aryl bromides.^25^ It should be noted that in most cases a high-energy-demanding UV radiation is required,^26^ and the use of visible light is relegated to the use of elaborated bimetallic (Ti/Pd) nanostructured composites as the photocatalyst at high temperatures.^27^

We recently focused on arylazo sulfones that incorporate a dyedauxiliary group (−N_2_SO_2_CH_3_) responsible for their color and their photoreactivity, useful precursors of reactive intermediates such as aryl, alkyl(aryl)sulfonyl, and aryldiazenyl radicals.^28^ These intermediates have been exploited in different synthetic protocols under photocatalyst-free conditions for the visible light-driven forging of C–C^29^ as well as C-heteroatom^30^ bonds.

In particular, the *in situ* generation of aryl radicals from arylazo sulfones has been recently merged with [Au(I)] catalysis by our groups for a Suzuki-type reaction.^31^ During this study, we were intrigued to detect in selected cases the corresponding homocoupling biaryl as a minor side product (2–5% yield in most cases; Scheme 1b).^31^ Stimulated by these early observations, we wondered if it was possible to design an unprecedented photocatalyst-free visible light-driven protocol for the synthesis of symmetrical (hetero)biaryls (Scheme 1c).

The present approach would represent an innovative procedure for the preparation of symmetrical biarenes via activation of an aromatic substrate upon direct visible light irradiation without the need for any sacrificial electron donors in stoichiometric amounts.

## Results and Discussion

To test our proposal, we first repeated the conditions applied in the Suzuki coupling but by omitting the boronic acid by electing the *p*-cyanophenylazo sulfone **1a** as the model substrate. However, the desired biaryl **2** was formed only in small amounts (see Table S1 in the Supporting Information for further information). We then carried out an extensive survey of reaction parameters (*i.e*., reaction media, light source, and the cocatalyst; see Table S1 for details) aiming to push the forging of the desired Ar–Ar bond. A representative list of control experiments is organized in Table 1. Upon this preliminary optimization stage, we were pleased to verify that by irradiating (Kessil lamp, 40 W, λ_em_ = 427 nm) an argon-equilibrated mixture of **1a** (0.1 M in CH_3_CN/H_2_O 9:1), (PPh_3_)AuCl (10 mol %), 1,10-phenanthroline (40 mol %), and NaHCO_3_ (2 equiv) for 24 h led to biaryl **2** in a 75% yield (entry 1, Table 1).

**Table 1:**
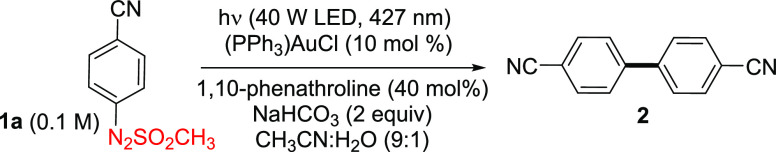
Optimization of the Reaction Conditions

entry	deviation from optimal conditions	**2** (% yield)^a^
1		75^b^
2	no (PPh_3_)AuCl	0^c^
3	dark	0
4	no NaHCO_3_	0
5	*h*ν (390 nm)	36
6	1,10-phenanthroline (20 mol %)	60
7	*p*-CNC_6_H_4_N_2_BF_4_ instead of **1a**	<5

a GC yield.

b Isolated yield after flash chromatography.

c Benzonitrile (88% yield) was found as the only product.

The presence of a buffering agent (NaHCO_3_) and the gold complex along with light irradiation is essential to the optimal outcome of the process (compare entry 1 *vs* entries 2–4). A lower **2** isolated yield was observed both by shifting to 390 nm (36%, entry 5) and by decreasing the loading of the 1,10-phenanthroline cocatalyst to 20 mol % (entry 6). Finally, the possible role of diazonium salts in the present methodology was excluded by replacing **1a** with the *p*-cyanophenyl diazonium tetrafluoroborate salt; under optimal conditions, a trace amount of **2** was detected (entry 7).

With the optimized protocol in our hand, we explored the scope of the reaction by testing a broad range of diversely functionalized monosubstituted arylazo sulfones **1a**–**1ac** (Figure S1 and Table 2). Gratifyingly, the corresponding biaryls were obtained in good to quantitative yields and with excellent functional group tolerance starting from 4- (**2**–**15**) and 3-substituted aromatic substrates (**16–21**), including the 4,4′-diacetyl derivative **12** (a precursor of antifungal *N,N*′-diaryl-bishydrazones)^32^ and the benzophenone dimer **15** (83% yield). In the case of **4**, however, a higher amount of the catalyst was mandatory to achieve a 93% yield. In this series, however, the formation of **9** was not observed with 4-ethinylphenyl azosulfone **1h**, and this is probably due to the competitive reactivity of [Au(I)] complexes with alkynes.^33^

**Table 2:**
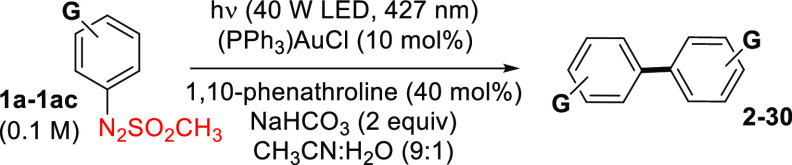

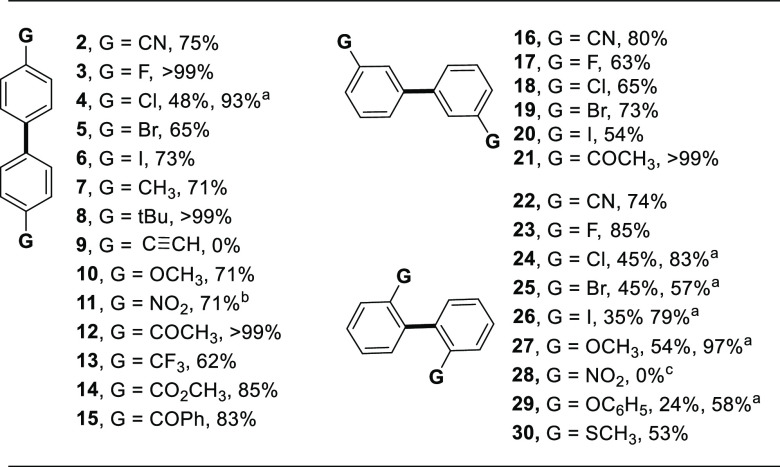
Synthesis of Symmetrical Biaryls from Monosubstituted Arylazo Sulfones

a (PPh_3_)AuCl (15 mol %) was employed.

b 1,2-Bis(4-nitrophenyl)diazene (**11a**, 7% yield) was isolated as the minor product.

c Nitrobenzene was found as the only product.

The reaction proved slightly less efficient with *ortho*-substituted arylazo sulfones (**1u–1ac**). In most cases, a 15 mol % amount of (PPh_3_)AuCl to achieve satisfactory results (see dihaloderivatives **24**–**26** and 2,2′-diphenoxybiphenyl **29**). As observed in previous works,^30^ the presence of a nitro group in the *ortho*-position in arylazo sulfones prevented the arylation as confirmed here, leading to the exclusive formation of nitrobenzene instead of the desired compound **28**.

The protocol was next successfully extended to the synthesis of polysubstituted biaryls **31–38** (Table 3), such as the polychlorinated biaryls **31** and **32** known as alkoxyresorufin *O*-dealkylase inhibitors.^34^ The only exception is represented by the 2,2′-dinitro derivative **38**, where again the hydrodeaminated *meta*-nitroanisole was formed instead. Gratifyingly, binaphthyls **39** and **40** and heteroarenes **41–43** were also isolated in up to quantitative yields. Interestingly, most protocols currently available for the synthesis of bipyridines present several limitations, including the low efficiency and limited scope.^35^ Furthermore, compound **42** found interesting application in the synthesis of binaphthyl–bipyridyl-based chiroptical switches.^36^

**Table 3:**
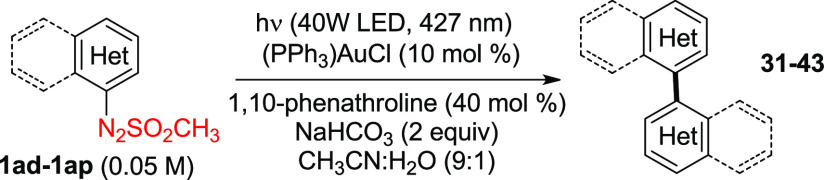

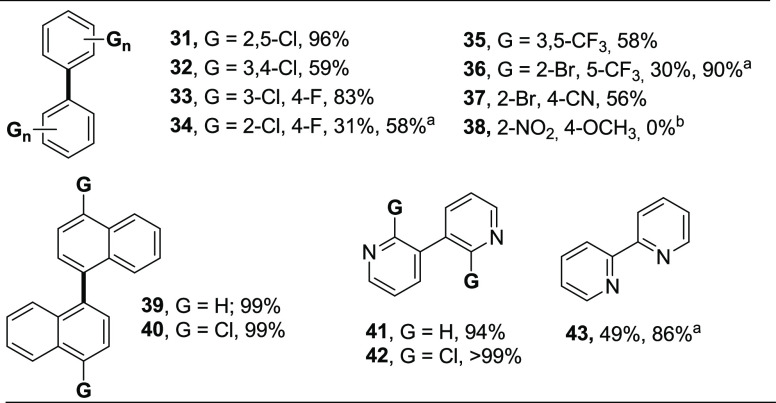
Visible Light-Driven Preparation of (Hetero)biaryls **31–43**

a (PPh_3_)AuCl (15 mol %) was employed.

b 3-Nitroanisole (70% yield) was found as the main product.

**Scheme 2:**
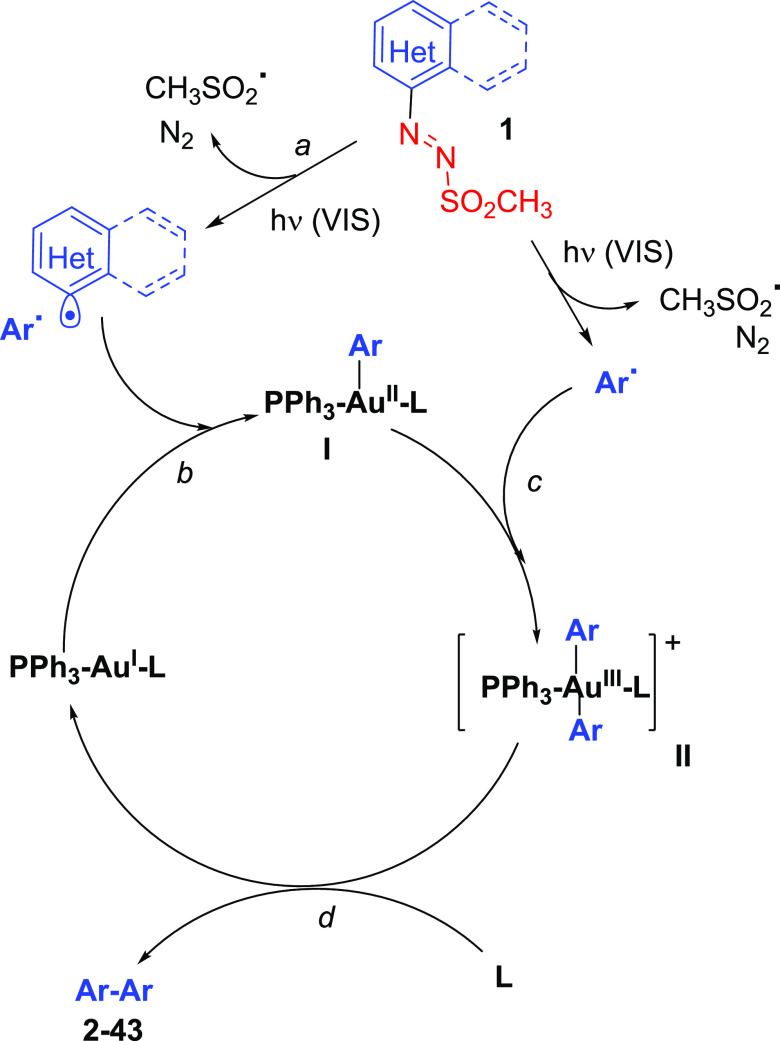
Proposed Mechanism

The mechanism proposed for the homocoupling is summarized in Scheme 2. Photolysis of arylazo sulfones to visible light causes the homolytic cleavage of the N–S bond, to release, after nitrogen loss from the first formed aryldiazenyl radical, an aryl (Ar^•^)/methanesulfonyl radical pair (Scheme 2, path a).^31^ Oxidative addition of Ar^•^ onto the PPh_3_Au^I^L catalyst (path b) resulted in the formation of the PPh_3_Au^II^LAr species **I**, which, in turn, intercepts a further Ar^•^ intermediate to afford the Au^III^ complex **II** (path c).^37^ The latter undergoes reductive elimination to release Ar–Ar while restoring the starting PPh_3_Au^I^L catalyst (path d).^38^ The intermediacy of an aryl radical was ascertained by an experiment carried out in the presence of TEMPO (0.05 M), showing a significant lowering of the biphenyl yield (from 75% to 29% in the case of **2**). As for the role of the cocatalyst, bis-pyridyl and phenanthryl ligands have been frequently adopted as beneficial additives in Au-mediated photo- and electrochemical coupling reactions.^39^ Although a conclusive answer on the real role of pyridine-based additives in Au(I)-mediated processes was not completely ascertained to date, their role as stabilizing agents of the high-oxidation-state gold complexes has been postulated.

In the field of light-driven processes, the present Au-catalyzed homocoupling results competitive in terms of efficiency and feasibility, with the other approaches already reported in the literature.^24−26^ The use of an easily available Au complex and the absence of a redox agent characterize the present procedure.^24^

## Conclusions

We presented herein the first visible light/Au(I)-catalyzed protocol for the preparation of symmetrical (hetero)biaryls by homocoupling of arylazo sulfones at room temperature in organic/aqueous media. The method exploits the properties of the N_2_SO_2_CH_3_ moiety as a dyedauxiliary group^28^ able to be activated directly with visible light without the intermediacy of a photocatalyst and exhibits an excellent functional group tolerance. The strategy has been exploited for the preparation of a wide range of symmetrical (hetero)biaryls in good to excellent yields with an easy setup.

## Experimental Section

### General

^1^H and ^13^C{^1^H} NMR spectra were recorded on a 300 MHz and 75 MHz spectrometer, respectively. The attributions were made on the basis of ^1^H and ^13^C NMR experiments; chemical shifts are reported in ppm downfield from TMS. GC analyses were performed using a HP SERIES 5890 II equipped with a fire ion detector (FID, temperature 350 °C). Analytes were separated using a Restek Rtx-5MS (30 m × 0.25 mm × 0.25 μm) capillary column with nitrogen as a carrier gas at 1 mL min^–1^. The injector temperature was 250 °C. The GC oven temperature was held at 80 °C for 2 min, increased to 250 °C by a temperature ramp of 10 °C min^–1^, and held for 10 min.

#### General Procedure for the Synthesis of Arylazo Sulfones **1a–1ap**

Arylazo sulfones **1a–ap** have been synthesized by following a known procedure starting from the corresponding aryl diazonium salts.^29c,30b^ Diazonium salts were freshly prepared prior to use from the corresponding anilines and purified by dissolving in acetonitrile and precipitation by adding cold diethyl ether. To a cooled (0 °C) suspension of the chosen diazonium salt (1 equiv, 0.3 M) in CH_2_Cl_2_ was added sodium methanesulfinate (1.2 equiv) in one portion. The temperature was allowed to increase to room temperature, and the solution was stirred overnight. The resulting mixture was then filtered, and the obtained solution was evaporated affording the desired arylazo sulfone. The crude product was finally dissolved in CH_2_Cl_2_ and precipitated by adding cold *n*-hexane. Compounds **1a–1aa**, **1ac–1al**, **1an**, and **1ao** have been fully characterized in previous works.^29c,30b^

##### 1-(Methylsulfonyl)-2-(2-phenoxyphenyl)diazene (**1ab**)

Orange solid, 56% yield, *T*_dec_: 87–88 °C. ^1^H NMR (300 MHz, CDCl_3_) δ 7.82 (dd, *J* = 8.2, 1.7 Hz, 1H), 7.67–7.61 (m, 1H), 7.40–7.32 (m, 2H), 7.29–7.23 (m, 1H), 7.21–7.12 (m, 2H), 7.10–6.99 (m, 2H), 2.86 (s, 3H). ^13^C{^1^H} NMR (75 MHz, CDCl_3_) δ 157.9, 157.0, 139.8, 137.3, 130.2, 124.3, 124.0, 121.3, 118.5, 118.1, 34.1. HRMS (ESI) *m*/*z*: calcd for C_13_H_12_N_2_O_3_S^+^ ([M + H]^+^) 299.0442; found 299.0461.

##### 3-Bromo-4-((methylsulfonyl)diazenyl)benzonitrile (**1aj**)

Orange solid, 69% yield, *T*_dec_: 132–132.5 °C. ^1^H NMR (300 MHz, CDCl_3_) δ 8.36–7.93 (m, 1H), 7.82–7.76 (m, 2H), 3.27 (s, 3H). ^13^C{^1^H} NMR (75 MHz, CDCl_3_) δ 148.6, 138.1, 132.2, 128.0, 119.2, 119.1, 116.2, 35.1. HRMS (ESI) *m*/*z*: calcd for C_8_H_6_N_3_O_2_SBr^+^ ([M + H]^+^) 287.9442; found 287.9425.

##### 1-(4-Methoxy-2-nitrophenyl)-2-(methylsulfonyl)diazene (**1ak**)

Orange solid, 54% yield, *T*_dec_: 95–97 °C. ^1^H NMR (300 MHz, CDCl_3_) δ 7.85 (d, *J* = 9.1 Hz, 1H), 7.45 (d, *J* = 2.7 Hz, 1H), 7.34–7.13 (m, 2H), 4.03 (s, 3H), 3.16 (s, 3H). ^13^C{^1^H} NMR (75 MHz, CDCl_3_) δ 165.20, 134.47, 119.71, 119.06, 109.69, 56.89. HRMS (ESI) *m*/*z*: calcd for C_8_H_6_N_3_O_2_SBr^+^ ([M + H]^+^) 286.9364; found 286.9347.

##### 1-(4-Chloronaphthalen-1-yl)-2-(methylsulfonyl)diazene (**1am**)

Orange solid, 34% yield, *T*_dec_: 125–127 °C. ^1^H NMR (300 MHz, CDCl_3_) δ 8.76–8.49 (m, 1H), 8.42–8.18 (m, 1H), 7.83 (d, *J* = 8.2 Hz, 1H), 7.77–7.67 (m, 2H), 7.62 (d, *J* = 8.3 Hz, 1H), 3.33 (s, 3H). ^13^C{^1^H} NMR (75 MHz, CDCl_3_) δ 142.7, 140.7, 132.4, 131.5, 129.4, 128.5, 126.1, 125.0, 123.0, 114.4, 35.4. HRMS (ESI) *m*/*z* calcd for C_11_H_9_N_2_O_2_SCl^+^ ([M + H]^+^) 269.0152; found 269.0158.

#### Procedure for the Preparation of 2-((Methylsulfonyl)diazenyl)pyridine (**1ap**)

*N*′-(Pyridin-2-yl)methanesulfonohydrazide was initially prepared by mixing 2-hydrazinylpyridine (0.300 g, 2.7 mmol) and methanesulfonyl chloride (0.212 mL, 2.7 mmol) in 3 mL of pyridine (37.4 mmol), as previously described.^40^ The crude mixture containing the sulfonohydrazide was oxidized by treatment with *N*-bromosuccinimide (0.475 g, 2.7 mmol) following a known procedure^41^ to give **1ap** as a yellow solid (134.7 mg, 0.72 mmol, 26% yield, *T*_dec_: 72–73 °C).

**1ap**: ^1^H NMR (300 MHz, CDCl_3_) δ 8.91–8.64 (m, 1H), 8.01 (td, *J* = 7.6, 1.8 Hz, 1H), 7.94–7.85 (m, 1H), 7.59 (ddd, *J* = 7.4, 4.6, 1.3 Hz, 1H), 3.29 (s, 3H). ^13^C{^1^H} NMR (75 MHz, CDCl_3_) δ 150.3, 139.8, 139.3, 118.3, 117.7, 43.0. HRMS (EsI) *m*/*z* calcd for C_6_H_7_N_3_O_2_S^+^ ([M + H^+^]) 186.0337; found 186.0392.

#### General Procedure for the Photochemical Synthesis of Biaryls

A pyrex glass vessel was charged with the chosen arylazo sulfone (**1a–ap**, 0.5 mmol, 1.0 equiv, 0.1 M) and 40 mg of sodium bicarbonate (1.0 mmol, 0.2 M), and the solid was dissolved in degassed acetonitrile/water (9:1, 5.0 mL); then, triphenylphosphine gold(I) chloride (0.05 mmol, 10 mol %) and 1,10-phenanthroline (40 mol %, 0.04 M) were added and the obtained mixture was flushed with argon. Irradiation was carried out for 24 h by means of a 40 W Kessil lamp (emission at 427 nm; see Figure S2 for further information). The photolyzed solution was concentrated under reduced pressure and purified by silica gel column chromatography (cyclohexane–ethyl acetate mixture as eluant).

##### [1,1′-Biphenyl]-4,4′-dicarbonitrile (**2**)

From 104.5 mg (0.500 mmol) of **1a**, 25.0 mg (0.05 mol, 10 mol %) of (PPh_3_)AuCl, 40.0 mg of NaHCO_3_ (1.0 mmol), and 40.0 mg of 1,10-phenanthroline (0.1 mmol, 40 mol %) in 5 mL of degassed acetonitrile/water (9:1). Purification was carried out by silica gel chromatographic column (eluant: neat cyclohexane) to afford 38.3 mg of **2** (75% yield, white solid, mp = 233–234 °C). Spectroscopic data were in accordance with the literature data.^42^ When the reaction was carried out in the presence of TEMPO (0.1 M), product **2** was obtained in only 29% yield.

^1^H NMR (300 MHz, CDCl_3_) δ 7.80 (d, *J* = 8.4 Hz, 4H), 7.71 (d, *J* = 8.5 Hz, 4H). ^13^C{^1^H} NMR (75 MHz, CDCl_3_) δ 143.7, 133.1, 128.1, 118.5, 112.6.

##### 4,4′-Difluoro-1,1′-biphenyl (**3**)

From 101.0 mg (0.500 mmol) of **1b**, 25.0 mg (0.05 mol, 10 mol %) of (PPh_3_)AuCl, 40.0 mg of NaHCO_3_ (1.0 mmol), and 40.0 mg of 1,10-phenanthroline (0.2 mmol, 40 mol %) in 5 mL of degassed acetonitrile/water (9:1). Purification was carried out by silica gel chromatographic column (eluant: neat cyclohexane) to afford 47.5 mg of **3** (>99% yield, white solid, mp = 93–95 °C). Spectroscopic data were in accordance with the literature data.^42^^1^H NMR (300 MHz, CDCl_3_) δ 7.49–7.40 (m, 2H), 7.39 (s, 2H), 7.32–7.25 (m, 2H), 7.14–7.05 (m, 2H). ^13^C{^1^H} NMR (75 MHz, CDCl_3_) δ 164.6 (d, *J* = 244.5 Hz), 137.0 (d, *J* = 3 Hz), 129.2 (d, *J* = 8.3 Hz), 116.4 (d, *J* = 21.8 Hz).

##### 4,4′-Dichloro-1,1′-biphenyl (**4**)

From 109.7 mg (0.501 mmol) of **1c**, 25.0 mg (0.05 mol, 10 mol %) of (PPh_3_)AuCl, 40.0 mg of NaHCO_3_ (1.0 mmol), and 40.0 mg of 1,10-phenanthroline (0.2 mmol, 40 mol %) in 5 mL of degassed acetonitrile/water (9:1). Purification was carried out by silica gel chromatographic column (eluant: neat cyclohexane) to afford 26.8 mg of **4** (48% yield, white solid, mp = 145–146 °C). The same reaction performed with 37.5 mg of (PPh_3_)AuCl (15 mol %) gave **4** in a 93% yield. Spectroscopic data were in accordance with the literature data.^42^^1^H NMR (300 MHz, CDCl_3_) δ 7.52–7.46 (m, 4H), 7.46–7.40 (m, 4H). ^13^C{^1^H} NMR (75 MHz, CDCl_3_) δ 138.3, 133.6, 128.9, 128.1.

##### 4,4′-Dibromo-1,1′-biphenyl (**5**)

From 132.5 mg (0.502 mmol) of **1d**, 25.0 mg (0.05 mol, 10 mol %) of (PPh_3_)AuCl, 40.0 mg of NaHCO_3_ (1.0 mmol), and 40.0 mg of 1,10-phenanthroline (0.2 mmol, 40 mol %) in 5 mL of degassed acetonitrile/water (9:1). Purification was carried out by silica gel chromatographic column (eluant: neat cyclohexane) to afford 50.9 mg of **5** (65% yield, slightly orange solid, mp = 165–166 °C). Spectroscopic data were in accordance with the literature data.^42^^1^H NMR (300 MHz, CDCl_3_) δ 7.58 (d, *J* = 8.6 Hz, 4H), 7.43 (d, *J* = 8.6 Hz, 4H). ^13^C{^1^H} NMR (75 MHz, CDCl_3_) δ 138.8, 131.9, 128.4, 121.9.

##### 4,4′-Diiodo-1,1′-biphenyl (**6**)

From 155.8 mg (0.502 mmol) of **1e**, 25.0 mg (0.05 mmol, 10 mol %) of (PPh_3_)AuCl, 40.0 mg of NaHCO_3_ (1.0 mmol), and 40.0 mg of 1,10-phenanthroline (0.2 mmol, 40 mol %) in 5 mL of degassed acetonitrile/water (9:1). Purification was carried out by silica gel chromatographic column (eluant: neat cyclohexane) to afford 74.4 mg of **6** (73% yield, slightly yellow solid, mp = 202–203 °C). Spectroscopic data were in accordance with the literature data.^43^^1^H NMR (300 MHz, CDCl_3_) δ 7.61 (d, *J* = 8.6 Hz, 4H), 7.13 (d, *J* = 8.6 Hz, 4H). ^13^C{^1^H} NMR (75 MHz, CDCl_3_) δ 139.5, 138.2, 128.8, 93.6.

##### 4,4′-Dimethyl-1,1′-biphenyl (**7**)

From 90.0 mg (0.502 mmol) of **1f**, 25.0 mg (0.05 mmol, 10 mol %) of (PPh_3_)AuCl, 40.0 mg of NaHCO_3_ (1.0 mmol), and 40.0 mg of 1,10-phenanthroline (0.2 mmol, 40 mol %) in 5 mL of degassed acetonitrile/water (9:1). Purification was carried out by silica gel chromatographic column (eluant: neat cyclohexane) to afford 28.2 mg of **7** (71% yield, white solid, mp = 118–120 °C). Spectroscopic data were in accordance with the literature data.^44^^1^H NMR (300 MHz, CDCl_3_) δ 7.52 (d, *J* = 8.1 Hz, 4H), 7.28 (d, *J* = 7.8 Hz, 4H), 2.43 (s, 6H). ^13^C{^1^H} NMR (75 MHz, CDCl_3_) δ 138.2, 136.6, 129.3, 126.7, 21.0.

##### 4,4′-Di-*tert*-butyl-1,1′-biphenyl (**8**)

From 120.0 mg (0.500 mmol) of **1g**, 25.0 mg (0.05 mmol, 10 mol %) of (PPh_3_)AuCl, 40.0 mg of NaHCO_3_ (1.0 mmol), and 40.0 mg of 1,10-phenanthroline (0.2 mmol, 40 mol %) in 5 mL of degassed acetonitrile/water (9:1). Purification was carried out by silica gel chromatographic column (eluant: neat cyclohexane) to afford 66.5 mg of **8** (>99% yield, white solid, mp = 126–127 °C). Spectroscopic data were in accordance with the literature data.^45^^1^H NMR (300 MHz, CDCl_3_) δ 7.57–7.50 (m, 4H), 7.49–7.43 (m, 4H), 1.37 (s, 18H). ^13^C{^1^H} NMR (75 MHz, CDCl_3_) δ 150.1, 138.3, 126.8, 125.8, 34.6, 31.5.

##### 4,4′-Dimethoxy-1,1′-biphenyl (**10**)

From 119.5 mg (0.504 mmol) of **1i**, 25.0 mg (0.05 mmol, 10 mol %) of (PPh_3_)AuCl, 40.0 mg of NaHCO_3_ (1.0 mmol), and 40.0 mg of 1,10-phenanthroline (0.2 mmol, 40 mol %) in 5 mL of degassed acetonitrile/water (9:1). Purification was carried out by silica gel chromatographic column (eluant: cyclohexane/ethyl acetate 95:5) to afford 38.3 mg of **10** (71% yield, white solid, mp = 178–180 °C). Spectroscopic data were in accordance with the literature data.^44^^1^H NMR (300 MHz, CDCl_3_) δ 7.49 (d, *J* = 8.8 Hz, 4H), 6.97 (d, *J* = 8.8 Hz, 4H), 3.85 (s, 6H). ^13^C{^1^H} NMR (75 MHz, CDCl_3_) δ 158.6, 133.4, 127.6, 114.1, 55.2.

##### 4,4′-Dinitro-1,1′-biphenyl (**11**)

From 115.5 mg (0.502 mmol) of **1j**, 25.0 mg (0.05 mmol, 10 mol %) of (PPh_3_)AuCl, 40.0 mg of NaHCO_3_ (1.0 mmol), and 40.0 mg of 1,10-phenanthroline (0.2 mmol, 40 mol %) in 5 mL of degassed acetonitrile/water (9:1). Purification was carried out by silica gel chromatographic column (eluant: neat cyclohexane) to afford 43.4 mg of **11** (71% yield, white solid, mp 225–226 °C) and 4.8 mg of 1,2-bis(4-nitrophenyl)diazene **11a** (7% yield, red oil). Spectroscopic data were in accordance with the literature data.^44^^1^H NMR (300 MHz, CDCl_3_) δ 8.37 (d, *J* = 8.8 Hz, 4H), 7.79 (d, *J* = 8.8 Hz, 4H). ^13^C{^1^H} NMR (75 MHz, CDCl_3_) δ 148.2, 145.1, 128.5, 124.5.

##### 1,1′-([1,1′-Biphenyl]-4,4′-diyl)bis(ethan-1-one) (**12**)

From 113.0 mg (0.500 mmol) of **1k**, 25.0 mg (0.05 mmol, 10 mol %) of (PPh_3_)AuCl, 40.0 mg of NaHCO_3_ (1.0 mmol), and 40.0 mg of 1,10-phenanthroline (0.2 mmol, 40 mol %) in 5 mL of degassed acetonitrile/water (9:1). Purification was carried out by silica gel chromatographic column (eluant: cyclohexane/ethyl acetate 92:8) to afford 60.3 mg of **12** (>99% yield, white solid, mp 187–189 °C). Spectroscopic data were in accordance with the literature data.^42^^1^H NMR (300 MHz, CDCl_3_) δ 8.05 (d, *J* = 8.4 Hz, 4H), 7.71 (d, *J* = 8.5 Hz, 4H), 2.64 (s, 6H). ^13^C{^1^H} NMR (75 MHz, CDCl_3_) δ 197.5, 144.2, 136.5, 128.9, 127.3, 26.6.

##### 4,4′-Bis(trifluoromethyl)-1,1′-biphenyl (**13**)

From 127.3 mg (0.500 mmol) of **1l**, 25.0 mg (0.05 mmol, 10 mol %) of (PPh_3_)AuCl, 40.0 mg of NaHCO_3_ (1.0 mmol), and 40.0 mg of 1,10-phenanthroline (0.2 mmol, 40 mol %) in 5 mL of degassed acetonitrile/water (9:1). Purification was carried out by silica gel chromatographic column (eluant: cyclohexane) to afford 45.0 mg of **13** (62% yield, white solid, mp 85–87 °C). Spectroscopic data were in accordance with the literature data.^42^^1^H NMR (300 MHz, CDCl_3_) δ 7.83–7.58 (m, 8H). ^13^C{^1^H} NMR (75 MHz, CDCl_3_) δ 143.4, 130.7 (q, *J* = 32.3 Hz), 127.8, 126.2 (q, *J* = 3.8 Hz), 123.5 (q, *J* = 270 Hz).

##### Dimethyl [1,1′-Biphenyl]-4,4′-dicarboxylate (**14**)

From 121.7 mg (0.500 mmol) of **1m**, 25.0 mg (0.05 mmol, 10 mol %) of (PPh_3_)AuCl, 40.0 mg of NaHCO_3_ (1.0 mmol), and 40.0 mg of 1,10-phenanthroline (0.2 mmol, 40 mol %) in 5 mL of degassed acetonitrile/water (9:1). Purification was carried out by silica gel chromatographic column (eluant: cyclohexane/ethyl acetate 9:1) to afford 57.8 mg of **14** (85% yield, white solid, mp 215–216 °C). Spectroscopic data were in accordance with the literature data.^42^^1^H NMR (300 MHz, CDCl_3_) δ 8.13 (d, *J* = 8.3 Hz, 4H), 7.69 (d, *J* = 8.3 Hz, 4H), 3.95 (s, 6H). ^13^C{^1^H} NMR (75 MHz, CDCl_3_) δ 167.4, 144.9, 130.8, 130.3, 127.8, 52.8.

##### [1,1′-Biphenyl]-4,4′-diylbis(phenylmethanone) (**15**)

From 144.0 mg (0.500 mmol) of **1n**, 25.0 mg (0.05 mmol, 10 mol %) of (PPh_3_)AuCl, 40.0 mg of NaHCO_3_ (1.0 mmol), and 40.0 mg of 1,10-phenanthroline (0.2 mmol, 40 mol %) in 5 mL of degassed acetonitrile/water (9:1). Purification was carried out by silica gel chromatographic column (eluant: neat cyclohexane) to afford 75.2 mg of **15** (83% yield, slightly yellow solid, mp 215–216 °C). Spectroscopic data were in accordance with the literature data.^46^^1^H NMR (300 MHz, CDCl_3_) δ 7.93 (d, *J* = 8.3 Hz, 4H), 7.87–7.82 (m, 4H), 7.77 (d, *J* = 8.3 Hz, 4H), 7.65–7.59 (m, 2H), 7.52 (dd, *J* = 8.2, 6.8 Hz, 4H). ^13^C{^1^H} NMR (75 MHz, CDCl_3_) δ 196.3, 144.0, 137.7, 137.2, 132.7, 130.9, 130.2, 128.5, 127.3.

##### [1,1′-Biphenyl]-3,3′-dicarbonitrile (**16**)

From 104.5 mg (0.500 mmol) of **1o**, 25.0 mg (0.05 mmol, 10 mol %) of (PPh_3_)AuCl, 40.0 mg of NaHCO_3_ (1.0 mmol), and 40.0 mg of 1,10-phenanthroline (0.2 mmol, 40 mol %) in 5 mL of degassed acetonitrile/water (9:1). Purification was carried out by silica gel chromatographic column (eluant: cyclohexane/ethyl acetate 95:5) to afford 40.8 mg of **16** (80% yield, white solid, mp = 190–192 °C). Spectroscopic data were in accordance with the literature data.^42^^1^H NMR (300 MHz, CDCl_3_) δ 7.90–7.75 (m, 4H), 7.71 (d, *J* = 7.7 Hz, 2H), 7.61 (t, *J* = 7.7 Hz, 2H). ^13^C{^1^H} NMR (75 MHz, CDCl_3_) δ 140.3, 131.9, 131.6, 130.8, 130.2, 118.5, 113.7.

##### 3,3′-Difluoro-1,1′-biphenyl (**17**)

From 101.0 mg (0.500 mmol) of **1p**, 25.0 mg (0.05 mmol, 10 mol %) of (PPh_3_)AuCl, 40.0 mg of NaHCO_3_ (1.0 mmol), and 40.0 mg of 1,10-phenanthroline (0.2 mmol, 40 mol %) in 5 mL of degassed acetonitrile/water (9:1). Purification was carried out by silica gel chromatographic column (eluant: neat cyclohexane) to afford 29.8 mg of **17** (63% yield, colorless oil). Spectroscopic data were in accordance with literature data.^47^^1^H NMR (300 MHz, CDCl_3_) δ 7.49–7.34 (m, 4H), 7.29 (dt, *J* = 10.1, 2.1 Hz, 2H), 7.19–6.92 (m, 2H). ^13^C{^1^H} NMR (75 MHz, CDCl_3_) δ 164.5 (d, *J* = 244.5 Hz), 142.0 (q, *J* = 3.8 Hz), 130.2 (d, *J* = 8.3 Hz), 122.5 (d, *J* = 3 Hz), 114.5 (d, *J* = 21 Hz), 113.9 (d, *J* = 22.5 Hz).

##### 3,3′-Dichloro-1,1′-biphenyl (**18**)

From 108.5 mg (0.500 mmol) of **1q**, 25.0 mg (0.05 mmol, 10 mol %) of (PPh_3_)AuCl, 40.0 mg of NaHCO_3_ (1.0 mmol), and 40.0 mg of 1,10-phenanthroline (0.2 mmol, 40 mol %) in 5 mL of degassed acetonitrile/water (9:1). Purification was carried out by silica gel chromatographic column (eluant: neat cyclohexane) to afford 35.8 mg of **18** (65% yield, slightly orange oil). Spectroscopic data were in accordance with the literature data.^42^^1^H NMR (300 MHz, CDCl_3_) δ 7.55 (d, *J* = 1.7 Hz, 2H), 7.45–7.41 (m, 2H), 7.38–7.33 (m, 4H). ^13^C{^1^H} NMR (75 MHz, CDCl_3_) δ 141.8, 135.0, 130.3, 128.0, 127.4, 125.4.

##### 3,3′-Dibromo-1,1′-biphenyl (**19**)

From 132.3 mg (0.501 mmol) of **1r**, 25.0 mg (0.05 mmol, 10 mol %) of (PPh_3_)AuCl, 40.0 mg of NaHCO_3_ (1.0 mmol), and 40.0 mg of 1,10-phenanthroline (0.2 mmol, 40 mol %) in 5 mL of degassed acetonitrile/water (9:1). Purification was carried out by silica gel chromatographic column (eluant: neat cyclohexane) to afford 56.6 mg of **19** (73% yield, white solid, mp = 52–54 °C). Spectroscopic data were in accordance with literature data.^48^^1^H NMR (300 MHz, CDCl_3_) δ 7.72 (t, *J* = 1.9 Hz, 1H), 7.51 (ddt, *J* = 9.7, 7.9, 1.1 Hz, 2H), 7.35 (d, *J* = 7.9 Hz, 1H). ^13^C{^1^H} NMR (75 MHz, CDCl_3_) δ 141.9, 131.0, 130.5, 130.3, 125.9, 123.1.

##### 3,3′-Diiodo-1,1′-biphenyl (**20**)

From 155.4 mg (0.501 mmol) of **1s**, 25.0 mg (0.05 mmol, 10 mol %) of (PPh_3_)AuCl, 40.0 mg of NaHCO_3_ (1.0 mmol), and 40.0 mg of 1,10-phenanthroline (0.2 mmol, 40 mol %) in 5 mL of degassed acetonitrile/water (9:1). Purification was carried out by silica gel chromatographic column (eluant: neat cyclohexane) to afford 54.8 mg of **20** (54% yield, white solid, mp 73–74 °C). Spectroscopic data were in accordance with the literature data.^49^^1^H NMR (300 MHz, CDCl_3_) δ 8.00 (d, *J* = 54.4 Hz, 1H), 7.73–7.64 (m, 1H), 7.59–7.47 (m, 1H), 7.19 (t, *J* = 7.9 Hz, 1H). ^13^C{^1^H} NMR (75 MHz, CDCl_3_) δ 135.7, 131.3, 130.2, 126.1, 94.5.

##### 1,1′-([1,1′-Biphenyl]-3,3′-diyl)bis(ethan-1-one) (**21**)

From 113.0 mg (0.500 mmol) of **1t**, 25.0 mg (0.05 mmol, 10 mol %) of (PPh_3_)AuCl, 40.0 mg of NaHCO_3_ (1.0 mmol), and 40.0 mg of 1,10-phenanthroline (0.2 mmol, 40 mol %) in 5 mL of degassed acetonitrile/water (9:1). Purification was carried out by silica gel chromatographic column (eluant: cyclohexane/ethyl acetate 92:8) to afford 59.5 mg of **21** (>99% yield, white solid, mp 125–126 °C). Spectroscopic data were in accordance with the literature data.^48^^1^H NMR (300 MHz, CDCl_3_) δ 8.19 (t, *J* = 1.8 Hz, 2H), 7.96 (m, *J* = 7.7, 1.8, 1.1 Hz, 2H), 7.81 (m, *J* = 7.7, 1.9, 1.1 Hz, 2H), 7.56 (t, *J* = 7.7 Hz, 2H), 2.66 (s, 6H). ^13^C{^1^H} NMR (75 MHz, CDCl_3_) δ 198.0, 140.8, 137.9, 131.9, 129.3, 127.9, 127.0, 26.9.

##### [1,1′-Biphenyl]-2,2′-dicarbonitrile (**22**)

From 104.5 mg (0.500 mmol) of **1u**, 25.0 mg (0.05 mmol, 10 mmol %) of (PPh_3_)AuCl, 40.0 mg of NaHCO_3_ (1.0 mmol), and 40.0 mg of 1,10-phenanthroline (0.2 mmol, 40 mol %) in 5 mL of degassed acetonitrile/water (9:1). Purification was carried out by silica gel chromatographic column (eluant: cyclohexane/ethyl acetate 95:5) to afford 37.7 mg of **22** (74% yield, white solid, mp = 171–173 °C). Spectroscopic data were in accordance with the literature data.^50^^1^H NMR (300 MHz, CDCl_3_) δ 7.87–7.80 (m, 2H), 7.75–7.69 (m, 2H), 7.62–7.55 (m, 4H). ^13^C{^1^H} NMR (75 MHz, CDCl_3_) δ 141.3, 133.3, 132.6, 130.3, 128.9, 117.2, 112.1.

##### 2,2′-Difluoro-1,1′-biphenyl (**23**)

From 102.0 mg (0.502 mmol) of **1v**, 25.0 mg (0.05 mmol, 10 mol %) of (PPh_3_)AuCl, 40.0 mg of NaHCO_3_ (1.0 mmol), and 40.0 mg of 1,10-phenanthroline (0.2 mmol, 40 mol %) in 5 mL of degassed acetonitrile/water (9:1). Purification was carried out by silica gel chromatographic column (eluant: neat cyclohexane) to afford 40.6 mg of **23** (85% yield, white solid, mp = 116–118 °C). Spectroscopic data were in accordance with the literature data.^47^^1^H NMR (300 MHz, CDCl_3_) δ 7.42 (dddd, *J* = 10.6, 7.9, 4.9, 2.3 Hz, 4H), 7.31–7.16 (m, 4H). ^13^C{^1^H} NMR (75 MHz, CDCl_3_) δ 161.2, 157.9, 131.3 (t, *J* = 2.3 Hz), 129.5 (t, *J* = 4.5 Hz), 123.8 (t, *J* = 2.3 Hz), 115.6 (q, *J* = 7.5 Hz).

##### 2,2′-Dichloro-1,1′-biphenyl (**24**)

From 108.5 mg (0.500 mmol) of **1w**, 25.0 mg (0.05 mmol, 10 mol %) of (PPh_3_)AuCl, 40.0 mg of NaHCO_3_ (1.0 mmol), and 40.0 mg of 1,10-phenanthroline (0.2 mmol, 40 mol %) in 5 mL of degassed acetonitrile/water (9:1). Purification was carried out by silica gel chromatographic column (eluant: neat cyclohexane) to afford 24.8 mg of **24** (45% yield, white solid, mp = 60–61 °C). The same reaction performed with 37.5 mg of (PPh_3_)AuCl (15 mol %) afforded **24** in an 83% yield. Spectroscopic data were in accordance with the literature data.^42^^1^H NMR (300 MHz, CDCl_3_) δ 7.58–7.46 (m, 2H), 7.40–7.33 (m, 4H), 7.31–7.26 (m, 2H). ^13^C{^1^H} NMR (75 MHz, CDCl_3_) δ 139.0, 134.1, 131.8, 130.0, 129.8, 127.1.

##### 2,2′-Dibromo-1,1′-biphenyl (**25**)

From 130.8 mg (0.500 mmol) of **1x**, 25.0 mg (0.05 mmol, 10 mol %) of (PPh_3_)AuCl, 40.0 mg of NaHCO_3_ (1.0 mmol), and 40.0 mg of 1,10-phenanthroline (0.2 mmol, 40 mol %) in 5 mL of degassed acetonitrile/water (9:1). Purification was carried out by silica gel chromatographic column (eluant: neat cyclohexane) to afford 36.5 mg of **25** (45% yield, white solid, mp = 77–79 °C). The same reaction performed with 34.9 mg of (PPh_3_)AuCl (15 mol %) gave **25** in a 57% yield. Spectroscopic data were in accordance with the literature data.^51^^1^H NMR (300 MHz, CDCl_3_) δ 7.72–7.67 (m, 2H), 7.42–7.37 (m, 2H), 7.31–7.26 (m, 4H). ^13^C{^1^H} NMR (75 MHz, CDCl_3_) δ 142.6, 133.1, 131.5, 129.9, 127.7, 124.1.

##### 2,2′-Diiodo-1,1′-biphenyl (**26**)

From 155.0 mg (0.501 mmol) of **1y**, 25.0 mg (0.05 mmol, 10 mol %) of (PPh_3_)AuCl, 40.0 mg of NaHCO_3_ (1.0 mmol), and 40.0 mg of 1,10-phenanthroline (0.2 mmol, 40 mol %) in 5 mL of degassed acetonitrile/water (9:1). Purification was carried out by silica gel chromatographic column (eluant: neat cyclohexane) to afford 35.6 mg of **26** (35% yield, white solid, mp = 109–112 °C). The same reaction performed with 37.5 mg of (PPh_3_)AuCl (15 mol %) afforded **26** in a 79% yield. Spectroscopic data were in accordance with the literature data.^51^^1^H NMR (300 MHz, CDCl_3_) δ 7.95 (dd, *J* = 8.0, 1.2 Hz, 2H), 7.41 (dd, *J* = 7.5, 1.2 Hz, 2H), 7.20 (dd, *J* = 7.6, 1.7 Hz, 2H), 7.09 (td, *J* = 7.7, 1.7 Hz, 2H). ^13^C{^1^H} NMR (75 MHz, CDCl_3_) δ 149.1, 139.0, 130.0, 129.5, 128.2, 99.8.

##### 2,2′-Dimethoxy-1,1′-biphenyl (**27**)

From 118.0 mg (0.500 mmol) of **1z**, 25.0 mg (0.05 mmol, 10 mol %) of (PPh_3_)AuCl, 40.0 mg of NaHCO_3_ (1.0 mmol), and 40.0 mg of 1,10-phenanthroline (0.2 mmol, 40 mol %) in 5 mL of degassed acetonitrile/water (9:1). Purification was carried out by silica gel chromatographic column (eluant: neat cyclohexane) to afford 28.8 mg of **27** (54% yield, white solid, mp = 154–156 °C). The same reaction performed with 37.5 mg of (PPh_3_)AuCl (15 mol %) gave **27** in a 97% yield. Spectroscopic data were in accordance with the literature data.^45^^1^H NMR (300 MHz, CDCl_3_) δ 7.37 (ddd, *J* = 8.2, 7.4, 1.8 Hz, 2H), 7.29 (dd, *J* = 7.4, 1.9 Hz, 2H), 7.11–6.92 (m, 4H), 3.81 (s, 6H). ^13^C{^1^H} NMR (75 MHz, CDCl_3_) δ 157.2, 131.6, 128.7, 128.0, 120.5, 111.2, 55.8.

##### Irradiation of 1-(Methylsulfonyl)-2-(2-nitrophenyl)diazene (**1aa**)

From 114.5 mg (0.500 mmol) of **1aa**, 25.0 mg (0.05 mmol, 10 mol %) of (PPh_3_)AuCl, 40.0 mg of NaHCO_3_ (1.0 mmol), and 40.0 mg of 1,10-phenanthroline (0.2 mmol, 40 mol %) in 5 mL of degassed acetonitrile/water (9:1). Purification was carried out by silica gel chromatographic column (eluant: neat cyclohexane) to afford 61.5 mg of nitrobenzene (yellow oil, quantitative yield).

##### 2,2′-Diphenoxy-1,1′-biphenyl (**29**)

From 139.0 mg (0.500 mmol) of **1ab**, 25.0 mg (0.05 mmol, 10 mol %) of (PPh_3_)AuCl, 40.0 mg of NaHCO_3_ (1.0 mmol), and 40.0 mg of 1,10-phenanthroline (0.2 mmol, 40 mol %) in 5 mL of degassed acetonitrile/water (9:1). Purification was carried out by silica gel chromatographic column (eluant: cyclohexane) to afford 19.6 mg of **29** (24% yield, white solid, mp 100–102 °C). The same reaction performed with 37.5 mg of (PPh_3_)AuCl (15 mol %) gave **29** in a 58% yield. Spectroscopic data were in accordance with the literature data.^52^^1^H NMR (300 MHz, CDC_l3_) δ 7.47 (dd, *J* = 7.6, 1.8 Hz, 2H), 7.34–7.12 (m, 9H), 7.06–7.00 (m, 2H), 6.96–6.87 (m, 5H). ^13^C{^1^H} NMR (75 MHz, CDCl_3_) δ 157.6, 154.8, 132.1, 129.9, 129.5, 128.9, 123.2, 122.8, 118.9, 118.8.

##### 2,2′-Bis(methylthio)-1,1′-biphenyl (**30**)

From 115.0 mg (0.500 mmol) of **1ac**, 25.0 mg (0.05 mmol, 10 mol %) of (PPh_3_)AuCl, 40.0 mg of NaHCO_3_ (1.0 mmol), and 40.0 mg of 1,10-phenanthroline (0.2 mmol, 40 mol %) in 5 mL of degassed acetonitrile/water (9:1). Purification was carried out by silica gel chromatographic column (eluant: cyclohexane) to afford 32.6 mg of **30** (53% yield, white solid, mp 45–46 °C). Spectroscopic data were in accordance with the literature data.^48^^1^H NMR (300 MHz, CDCl_3_) δ 7.48–7.39 (m, 2H), 7.33 (d, *J* = 7.2 Hz, 2H), 7.28–7.15 (m, 4H), 2.41 (s, 6H). ^13^C{^1^H} NMR (75 MHz, CDCl_3_) δ 138.5, 137.8, 129.7, 128.2, 124.7, 124.2, 15.4.

##### 2,2′,5,5′-Tetrachloro-1,1′-biphenyl (**31**)

From 126.0 mg (0.500 mmol) of **1ad**, 25.0 mg (0.05 mol, 10 mol %) of (PPh_3_)AuCl, 40.0 mg of NaHCO_3_ (1.0 mmol), and 40.0 mg of 1,10-phenanthroline (0.2 mmol, 40 mol %) in 5 mL of degassed acetonitrile/water (9:1). Purification was carried out by silica gel chromatographic column (eluant: neat cyclohexane) to afford 69.6 mg of **31** (96% yield, white solid, mp 65–66 °C). Spectroscopic data were in accordance with the literature data.^48^^1^H NMR (300 MHz, CDCl_3_) δ 7.43 (s, 2H), 7.36 (dd, *J* = 8.6, 2.5 Hz, 2H), 7.28 (d, *J* = 2.4 Hz, 2H). ^13^C{^1^H} NMR (75 MHz, CDCl_3_) δ 138.2, 132.2, 131.5, 130.6, 130.4, 129.4.

##### 3,3′,4,4′-Tetrachloro-1,1′-biphenyl (**32**)

From 126.0 mg (0.500 mmol) of **1ae**, 25.0 mg (0.05 mmol, 10 mol %) of (PPh_3_)AuCl, 40.0 mg of NaHCO_3_ (1.0 mmol), and 40.0 mg of 1,10-phenanthroline (0.2 mmol, 40 mol %) in 5 mL of degassed acetonitrile/water (9:1). Purification was carried out by silica gel chromatographic column (eluant: neat cyclohexane) to afford 42.8 mg of **32** (59% yield, white solid, mp 172–173 °C). Spectroscopic data were in accordance with the literature data.^42^^1^H NMR (300 MHz, CDCl_3_) δ 7.62 (d, *J* = 2.2 Hz, 2H), 7.51 (d, *J* = 8.3 Hz, 2H), 7.36 (dd, *J* = 8.4, 2.2 Hz, 2H). ^13^C{^1^H} NMR (75 MHz, CDCl_3_) δ 138.9, 133.4, 132.6, 131.1, 128.9, 126.3.

##### 3,3′-Dichloro-4,4′-difluoro-1,1′-biphenyl (**33**)

From 119.2 mg (0.500 mmol) of **1af**, 25.0 mg (0.05 mmol, 10 mol %) of (PPh_3_)AuCl, 40.0 mg of NaHCO_3_ (1.0 mmol), and 40.0 mg of 1,10-phenanthroline (0.2 mmol, 40 mol %) in 5 mL of degassed acetonitrile/water (9:1). Purification was carried out by silica gel chromatographic column (eluant: neat cyclohexane) to afford 54.2 mg of **33** (83% yield, white solid, mp 139–141 °C). Spectroscopic data were in accordance with literature data.^53^^1^H NMR (300 MHz, CDCl_3_) δ 7.56 (dd, *J* = 6.9, 2.4 Hz, 2H), 7.38 (ddd, *J* = 8.5, 4.5, 2.4 Hz, 2H), 7.23 (dd, *J* = 9.5, 7.7 Hz, 2H). ^13^C{^1^H} NMR (75 MHz, CDCl_3_) δ 159.7 (d, *J* = 249 Hz), 136.4 (d, *J* = 3.8 Hz), 129.3, 126.9 (d, *J* = 7.5 Hz), 121.8 (d, *J* = 18 Hz), 117.3 (d, *J* = 21 Hz). ^19^F NMR (376 MHz, CDCl_3_): δ −112.0.

##### .2,2′-Dichloro-4,4′-difluoro-1,1′-biphenyl (**34**)

From 118.9 mg (0.500 mmol) of **1ag**, 25.0 mg (0.05 mmol, 10 mol %) of (PPh_3_)AuCl, 40.0 mg of NaHCO_3_ (1.0 mmol), and 40.0 mg of 1,10-phenanthroline (0.2 mmol, 40 mol %) in 5 mL of degassed acetonitrile/water (9:1). Purification was carried out by silica gel chromatographic column (eluant: neat cyclohexane) to afford 20.5 mg of **34** (31% yield, colorless liquid). The same reaction performed with 37.5 mg of (PPh_3_)AuCl (15 mol %) gave **34** in a 58% yield. Spectroscopic data were in accordance with the literature data.^54^^1^H NMR (300 MHz, CDCl_3_) δ 7.29–7.21 (m, 4H), 7.08 (td, *J* = 8.3, 2.6 Hz, 2H). ^13^C{^1^H} NMR (75 MHz, CDCl_3_) δ 164.0 (d, *J* = 249 Hz), 134.8 (d, *J* = 9.8 Hz), 133.7 (d, *J* = 3.0 Hz), 132.6 (d, *J* = 9.0 Hz), 114.3 (d, *J* = 21 Hz). ^19^F NMR (376 MHz, CDCl_3_): δ −117.3.

##### 3,3′,5,5′-Tetrakis(trifluoromethyl)-1,1′-biphenyl (**35**)

From 171.0 mg (0.501 mmol) of **1ah**, 25.0 mg (0.05 mmol, 10 mol %) of (PPh_3_)AuCl, 40.0 mg of NaHCO_3_ (1.0 mmol), and 40.0 mg of 1,10-phenanthroline (0.2 mmol, 40 mmol %) in 5 mL of degassed acetonitrile/water (9:1). Purification was carried out by silica gel chromatographic column (eluant: neat cyclohexane) to afford 37.6 mg of **35** (32% yield, white solid, mp 79–81 °C). The same reaction performed with 37.5 mg of (PPh_3_)AuCl (15 mol %) gave **35** in a 58% yield. Spectroscopic data were in accordance with the literature data.^42^^1^H NMR (300 MHz, CDCl_3_) δ 8.01 (d, *J* = 12.4 Hz, 6H). ^13^C{^1^H} NMR (75 MHz, CDCl_3_) δ 140.6, 133.8 (q, *J* = 33.8 Hz), 128.6 (q, *J* = 272 Hz), 127.7 (d, *J* = 3.0 Hz), 122.9 (m, *J* = 3.8 Hz). ^19^F NMR (376 MHz, CDCl_3_): δ −63.3.

##### 2,2′-Dibromo-5,5′-bis(trifluoromethyl)-1,1′-biphenyl (**36**)

From 177.0 mg (0.504 mmol) of **1ai**, 25.0 mg (0.05 mmol, 10 mol %) of (PPh_3_)AuCl, 40.0 mg of NaHCO_3_ (1.0 mmol), and 40.0 mg of 1,10-phenanthroline (0.2 mmol, 40 mol %) in 5 mL of degassed acetonitrile/water (9:1). Purification was carried out by silica gel chromatographic column (eluant: neat cyclohexane) to afford 36.9 mg of **36** (30% yield, pale yellow solid, mp 98–100 °C). The same reaction performed with 37.5 mg of (PPh_3_)AuCl (15 mol %) gave **36** in a 90% yield. Spectroscopic data were in accordance with the literature data.^55^^1^H NMR (300 MHz, CDCl_3_) δ 7.88–7.76 (m, 4H), 7.57 (dd, *J* = 8.3, 2.3 Hz, 2H). ^13^C{^1^H} NMR (75 MHz, CDCl_3_) δ 138.3, 135.9, 131.8 (q, *J* = 31.5 Hz), 128.3 (q, *J* = 272 Hz), 126.5 (q, *J* = 6.8 Hz), 120.3 (q, *J* = 1.5 Hz). ^19^F NMR (376 MHz, CDCl_3_): δ −63.2.

##### 2,2′-Dibromo-[1,1′-biphenyl]-4,4′-dicarbonitrile (**37**)

From 144.5 mg (0.500 mmol) of **1aj**, 25.0 mg (0.05 mmol, 10 mol %) of (PPh_3_)AuCl, 40.0 mg of NaHCO_3_ (1.0 mmol), and 40.0 mg of 1,10-phenanthroline (0.2 mmol, 40 mol %) in 5 mL of degassed acetonitrile/water (9:1). Purification was carried out by silica gel chromatographic column (eluant: cyclohexane/ethyl acetate 9:1) to afford 40.8 mg of **37** (56% yield, pale orange solid, mp 180–183 °C). ^1^H NMR (300 MHz, CDCl_3_) δ 8.00 (d, *J* = 1.5 Hz, 2H), 7.74–7.70 (m, 2H), 7.34 (d, *J* = 7.9 Hz, 2H). ^13^C{^1^H} NMR (75 MHz, CDCl_3_) δ 145.2, 136.3, 131.2, 123.7, 117.3, 116.9, 114.5. HRMS (ESI) *m*/*z* calcd for C_14_H_6_N_2_Br_2_^+^ ([M + H]^+^) 360.8970; found 360.8947.

##### Irradiation of 1-(4-Methoxy-2-nitrophenyl)-2-(methylsulfonyl)diazene (**1ak**)

From 129.6 mg (0.5 mmol) of **1ak**, 25.0 mg (0.05 mmol, 10 mol %) of (PPh_3_)AuCl, 40.0 mg of NaHCO_3_ (1.0 mmol), and 40.0 mg of 1,10-phenanthroline (0.2 mmol, 40 mol %) in 5 mL of degassed acetonitrile/water (9:1). Purification was carried out by silica gel chromatographic column (eluant: neat cyclohexane) to afford 53.6 mg of 3-nitroanisole (70% yield).

##### 1,1′-Binaphthalene (**39**)

From 117.1 mg (0.500 mmol) of **1al**, 25.0 mg (0.05 mmol, 10 mol %) of (PPh_3_)AuCl, 40.0 mg of NaHCO_3_ (1.0 mmol), and 40.0 mg of 1,10-phenanthroline (0.2 mmol, 40 mol %) in 5 mL of degassed acetonitrile/water (9:1). Purification was carried out by silica gel chromatographic column (eluant: neat cyclohexane) to afford 62.6 mg of **39** (99% yield, white solid, mp 159–161 °C). Spectroscopic data were in accordance with the literature data.^42^^1^H NMR (300 MHz, CDCl_3_) δ 7.98 (ddd, *J* = 8.3, 3.1, 1.4 Hz, 2H), 7.63 (dd, *J* = 8.2, 7.0 Hz, 1H), 7.52 (ddt, *J* = 8.2, 6.8, 3.1 Hz, 2H), 7.43 (dd, *J* = 8.6, 1.2 Hz, 1H), 7.35–7.27 (m, 1H). ^13^C{^1^H} NMR (75 MHz, CDCl_3_) δ 138.3, 133.4, 132.7, 128.0, 127.8, 127.7, 126.4, 125.9, 125.7, 125.3.

##### 4,4′-Dichloro-1,1′-binaphthalene (**40**)

From 135.6 mg (0.501 mmol) of **1am**, 25.0 mg (0.05 mmol, 10 mol %) of (PPh_3_)AuCl, 40.0 mg of NaHCO_3_ (1.0 mmol), and 40.0 mg of 1,10-phenanthroline (0.2 mmol, 40 mol %) in 5 mL of degassed acetonitrile/water (9:1). Purification was carried out by silica gel chromatographic column (eluant: neat cyclohexane) to afford 81.4 mg of **40** (99% yield, red solid, mp 217–218 °C). Spectroscopic data were in accordance with the literature data.^56^^1^H NMR (300 MHz, CDCl_3_) δ 8.39 (d, *J* = 8.5 Hz, 2H), 7.69 (d, *J* = 7.6 Hz, 2H), 7.67–7.52 (m, 4H), 7.38–7.35 (m, 4H). ^13^C{^1^H} NMR (75 MHz, CDCl_3_) δ 137.1, 134.0, 132.2, 130.9, 127.9, 127.2, 127.0, 125.8, 124.9.

##### 3,3′-Bipyridine (**41**)

From 92.51 mg (0.500 mmol) of **1an**, 25.0 mg (0.05 mmol, 10 mol %) of (PPh_3_)AuCl, 40.0 mg of NaHCO_3_ (1.0 mmol), and 40.0 mg of 1,10-phenanthroline (0.2 mmol, 40 mol %) in 5 mL of degassed acetonitrile/water (9:1). Purification was carried out by silica gel chromatographic column (eluant: cyclohexane/ethyl acetate 3:7) to afford 74.1 mg of **41** (94% yield, slightly yellow solid, mp 64–66 °C). Spectroscopic data were in accordance with the literature data.^57^^1^H NMR (300 MHz, CDCl_3_) δ 8.86 (d, *J* = 2.4 Hz, 2H), 8.67 (dd, *J* = 4.9, 1.6 Hz, 2H), 7.91 (dt, *J* = 7.9, 2.0 Hz, 2H), 7.44 (dd, *J* = 7.9, 4.8 Hz, 2H). ^13^C{^1^H} NMR (75 MHz, CDCl_3_) δ 149.3, 148.1, 134.8, 133.7, 124.0.

##### 2,2′-Dichloro-3,3′-bipyridine (**42**)

From 109.5 mg (0.500 mmol) of **1ao**, 25.0 mg (0.05 mmol, 10 mol %) of (PPh_3_)AuCl, 40.0 mg of NaHCO_3_ (1.0 mmol), and 40.0 mg of 1,10-phenanthroline (0.2 mmol, 40 mol %) in 5 mL of degassed acetonitrile/water (9:1). Purification was carried out by silica gel chromatographic column (eluant: cyclohexane/ethyl acetate 7:3) to afford 56.0 mg of **42** (>99% yield, slightly yellow solid, mp 202–204 °C). Spectroscopic data were in accordance with the literature data.^58^^1^H NMR (300 MHz, CDCl_3_) δ 8.50 (dd, *J* = 4.8, 1.9 Hz, 2H), 7.67 (dd, *J* = 7.6, 1.9 Hz, 2H), 7.47–7.36 (m, 2H). ^13^C{^1^H} NMR (75 MHz, CDCl_3_) δ 150.4, 149.9, 140.0, 133.0, 122.5.

##### 2,2′-Bipyridine (**43**)

From 92.50 mg (0.500 mmol) of **1ap**, 25.0 mg (0.05 mmol, 10 mol %) of (PPh_3_)AuCl, 40.0 mg of NaHCO_3_ (1.0 mmol), and 40.0 mg of 1,10-phenanthroline (0.2 mmol, 40 mol %) in 5 mL of degassed acetonitrile/water (9:1). Purification was carried out by silica gel chromatographic column (eluant: cyclohexane/ethyl acetate 3:7) to afford 38.6 mg of **43** (49% yield, slightly yellow solid, mp 70–71 °C). The same reaction was performed with 37.5 mg of (PPh_3_)AuCl (15 mol %) and afforded **43** in an 86% yield. Spectroscopic data were in accordance with the literature data.^59^^1^H NMR (300 MHz, CDCl_3_) δ 8.69–8.65 (m, 2H), 8.40 (dd, *J* = 8.0, 1.2 Hz, 2H), 7.81 (td, *J* = 7.8, 1.8 Hz, 2H), 7.29 (ddd, *J* = 7.5, 4.8, 1.2 Hz, 2H). ^13^C{^1^H} NMR (75 MHz, CDCl_3_) δ 156.0, 149.2, 137.1, 123.9, 121.3.

#### Procedure for the Photochemical Synthesis of Biaryls **2** on a Larger Scale

A pyrex glass vessel was charged with the arylazo sulfone **1a** (2.36 mmol, 1.0 equiv, 0.1 M) and 400 mg of sodium bicarbonate (4.72 mmol, 2 equiv, 0.2 M), and the solid was dissolved in degassed acetonitrile/water (9:1, 24.0 mL); then, 118.2 mg of triphenylphosphine gold(I) chloride (0.24 mmol, 10 mol %) and 187 mg of 1,10-phenanthroline (40 mol %) were added and the obtained mixture was flushed with argon. Irradiation was carried out for 24 h by means of a 40 W Kessil lamp (emission at 427 nm; see Figure S3). The photolyzed solution was concentrated under reduced pressure and purified by silica gel column chromatography (cyclohexane–ethyl acetate 95:5 mixture as an eluant). Product **2** was obtained as a pale yellow solid in a 70% yield (337 mg, 1.66 mmol).

## Supporting Information Available

Experimental details and copy of ^1^H and ^13^C NMR spectra of the prepared compounds (PDF)
