# Supplementary material for: Visible Light-Driven, Gold(I)-Catalyzed Preparation of Symmetrical (Hetero)biaryls by Homocoupling of Arylazo Sulfones
Source: J Org Chem. 2022 Mar 22;87(7):4863–72. doi: 10.1021/acs.joc.2c00225 (PMC8981317; doi:10.1021/acs.joc.2c00225)

Supporting information

Visible Light-Driven, Gold(I) Catalyzed Preparation of Symmetrical (Hetero)biaryls by Homocoupling of Arylazo Sulfones

*Lorenzo Di Terlizzi,[a] Simone Scaringi,[a],[b] Carlotta Raviola,[a] Riccardo Pedrazzani,[c] Marco Bandini,[c] Maurizio Fagnoni[a] and Stefano Protti[a]**

[a] PhotoGreen Lab, Department of Chemistry, University of Pavia, Viale Taramelli 12, 27100 Pavia, Italy

[b] Department of Organic Chemistry, University of Geneva, 1211 Geneva, Switzerland

[c] Department of Chemistry "Giacomo Ciamician", Alma Mater Studiorum University of Bologna Via Selmi 2, 40126 Bologna, Italy

1. **Figures and Tables** **S2**
2. **Copy of the ^{1}H and ^{13}C NMR of arylazosulfones**

   (1ab, 1aj, 1ak 1am and 1ap) and biaryls 2-43. **S6**

1. Figure and Tables

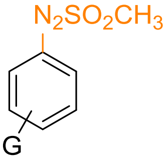

1a, G = 4-CN
1b, G = 4-F
1c, G = 4-Cl
1d, G = 4-Br
1e, G = 4-I
1f, G = 4-CH_3
1g, G = 4-*t*Bu
1h, G = 4- C≡CH
1i, G = 4-OCH_3
1j, G = 4-NO_2
1k, G = 4-$COCH_3$
1l, G = 4-CF_3
1m, G = 4-$COOCH_3$
1n, G = 4-COPh

1o, G = 3-CN
1p, G = 3-F
1q, G = 3-Cl
1r, G = 3-Br
1s, G = 3-I
1t, G = 3-$COCH_3$
1u, G = 2-CN
1v, G = 2-F
1w, G = 2-Cl
1x, G = 2-Br
1y, G = 2-I
1z, G = 2-OCH_3
1aa, G = 2-NO_2
1ab, G = 2-OPh
1ac, G = 2-SCH_3

1ad, G = 2,5-Cl
1ae, G = 3,4-Cl
1af, G = 4-F, 3-Cl
1ag, G = 4-F, 2-Cl
1ah, G = 2,5-CF_3
1ai, G = 2-Br, 5-CF_3
1aj, G = 2-Br, 4-CN
1ak, G = 2-NO_2, 4-OCH_3

1al,

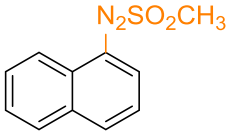

1an,

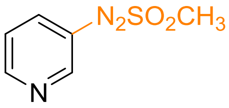

1ap,

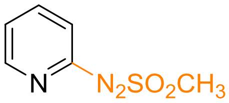

1am,

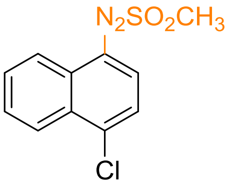

1ao,

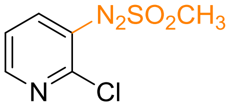

Figure S1: Arylazo sulfones employed in the present work.

Table S1. Optimization of the photochemical protocol.

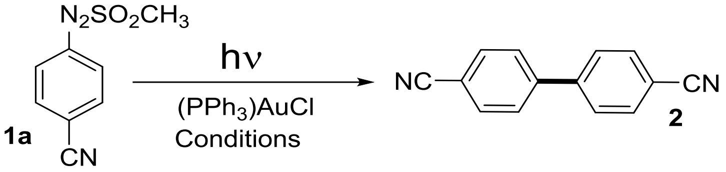

Entry	Conditions	2 (yield %)
1	**1a** (0.5 M), $(PPh_3)AuCl$ (5 mol%), 2,2'-bipyridine (20 mol%), NaOAc (2 equiv.), hν (456 nm), $CH_3OH:CH_3CN$ (3:1)	23%
2	**1a** (0.1 M), $(PPh_3)AuCl$ (5 mol%), 2,2'-bipyridine (20 mol%), NaOAc (2 equiv.), hν (456 nm), $CH_3OH:CH_3CN$ (3:1)	58%
3	**1a** (0.1 M), $(PPh_3)AuCl$ (5 mol%), 2,2'-bipyridine (20 mol%), NaOAc (2 equiv.), hν (456 nm), $CH_3CN:H_2O$ (9:1)	42%
4	**1a** (0.1 M), $(PPh_3)AuCl$ (5 mol%), 2,2'-bipyridine (20 mol%), NaOAc (2 equiv.), hν (427 nm), $CH_3CN:H_2O$ (9:1)	51%
5	**1a** (0.1 M), $(PPh_3)AuCl$ (5 mol%), 1,10-phenanthroline (20 mol%), NaOAc (2 equiv.), hν (427 nm), $CH_3CN:H_2O$ (9:1)	43%
6	**1a** (0.1 M), $(PPh_3)AuCl$ (10 mol%), 2,2'-bipyridine (20 mol%), NaOAc (2 equiv.), hν (427 nm), $CH_3CN:H_2O$ (9:1)	53%
7	**1a** (0.1 M), $(PPh_3)AuCl$ (10 mol%), 2,2'-bipyridine (20 mol%), $NaHCO_3$ (2 equiv.), hν (427 nm), $CH_3CN:H_2O$ (9:1)	21%
8	**1a** (0.1 M), $(PPh_3)AuCl$ (10 mol%), 2,2'-bipyridine (20 mol%), $PhI(OAc)_2$ (20 mol%), $NaHCO_3$ (2 equiv.), hν (427 nm), $CH_3CN:H_2O$ (9:1)	0%
9	**1a** (0.1 M), $(PPh_3)AuCl$ (10 mol%), 1,10-phenathroline (20 mol%), $NaHCO_3$ (2 equiv.), hν (427 nm), $CH_3CN:H_2O$ (9:1)	60%
**10**	**1a (0.1 M), $(PPh_3)AuCl$ (10 mol%), 1,10-phenathroline (40 mol%), $NaHCO_3$ (2 equiv.), hν (427 nm), $CH_3CN:H_2O$ (9:1)**	**75%**
11	**1a** (0.1 M), $(PPh_3)AuCl$ (10 mol%), 1,10-phenathroline (40 mol%), $NaHCO_3$ (2 equiv), hν (390 nm), $CH_3CN:H_2O$ (9:1)	36%
12	$4\text{-}CNC_6H_4N_2^+ BF_4^-$ (0.1 M), $(PPh_3)AuCl$ (10 mol%), 1,10-phenanthroline (40 mol%), $NaHCO_3$ (0.2 M), hν (427 nm), $CH_3CN:H_2O$ (9:1)	4%
13	**1a** (0.1 M), $(PPh_3)AuCl$ (10 mol%), 1,10-phenanthroline (40 mol%), hν (427 nm), $CH_3CN:H_2O$ (9:1)	3%
14	**1a** (0.1 M), hν (427 nm), $CH_3CN:H_2O$ (9:1)	0%
15	**1a** (0.1 M), $(PPh_3)AuCl$ (10 mol%), $CH_3CN:H_2O$ (9:1)	0%

Reaction optimization was carried out to find the best reaction conditions. Irradiation of a solution of **1a** (0.5 M), with gold triphenylphosphine chloride (5 mol%), 2,2'-bipyridine (20 mol%) and NaOAc (2 equiv) gave biaryl **2** in 23% yield (**entry 1**). When repeating the same reaction with a lower concentration of **1a** (0.1 M) the desired biaryl was isolated in 58% yield (**entry 2**). Shifting to a acetonitrile:water 9:1 mixture did not improve significantly the product formation (ca. 42% **entry 3**). However, moving the irradiation wavelength from 456 nm to 427 nm led to a 51% yield of the biaryl product (**entry 4**). A change of the ligand (from 2,2'-bipiridine to 1,10-phenanthroline) led to a lower yield of **2** (43 % **entry 5**), but when the reaction was carried out using 1,10-phenanthroline as a ligand and $NaHCO_3$ (2 equiv.) as a base, **2** was obtained in 60% yield when the ligand was used in 20 mol% amount and 75% when used in 40 mol% (**entries 9** and **10,** respectively). The same conditions were employed with the irradiation wavelength centred at 390 nm resulting in the formation of **2** in 36% of yield (**entry 11**). Moreover, performing the reaction with the corresponding diazonium salt instead of the arylazo sulfone led to a limited product formation (4%, **entry 12**). Carrying out the reaction without sodium bicarbonate, lowered the yield to 3% (**entry 13**). Finally, the photochemical homocoupling of **1a** in the absence of the gold catalyst or light (covering the reaction vessel with an aluminium foil) led to no product formation (**entries 14** and **15**).

General Procedure for the photochemical synthesis of biaryls. A pyrex glass vessel was charged with the chosen arylazo sulfone (**1a-ap**, 0.5 mmol, 1.0 equiv, 0.1 M) and 40 mg of sodium bicarbonate (1.0 mmol, 0.2 M) and the solid was dissolved in degassed acetonitrile:water (9:1, 5.0 mL), then, triphenylphosphine gold (I) chloride (0.05 mmol, 10 mol%) and 1,10-phenanthroline (40 mol%, 0.04 M) were added and the obtained mixture flushed with Argon. Irradiation was carried out for 24 h by means of a 40 W Kessil lamp (emission at 427 nm, see Figure S2). The photolyzed solution was concentrated under reduced pressure and purified by silica gel column chromatography (cyclohexane-ethyl acetate mixture as eluant).

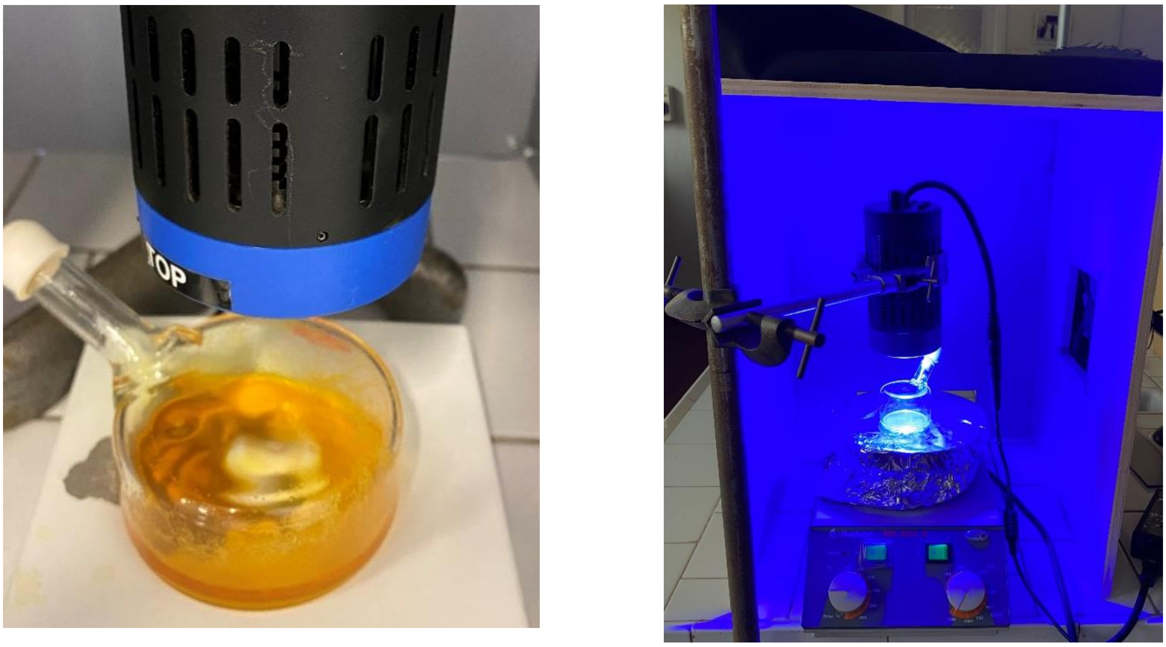

Figure S2: Irradiation system used in this work: A 40 W Kessil lamp (with emission centered at 427 nm) is held three centimetres above the glass reaction vessel which was stirred gently for 24 hours. A fan is placed on the right of the reaction vessel to avoid any heating of the solution.

Procedure for the photochemical synthesis of biaryls 2 on large scale. A pyrex glass vessel was charged with the arylazo sulfone **1a (**2.36 mmol, 1.0 equiv, 0.1 M) and 400 mg of sodium bicarbonate (4.72 mmol, 2 equiv. 0.2 M) and the solid was dissolved in degassed acetonitrile:water (9:1, 24.0 mL), then, 118.2 mg of triphenylphosphine gold (I) chloride (0.24 mmol, 10 mol%) and 187 mg of 1,10-phenanthroline (40 mol%) were added and the obtained mixture flushed with Argon. Irradiation was carried out for 24 h by means of a 40 W Kessil lamp (emission at 427 nm). The photolyzed solution was concentrated under reduced pressure and purified by silica gel column chromatography (cyclohexane-ethyl acetate 95:5 mixture as eluant). The product **2** was obtained as a pale yellow solid in 70% yield (337 mg, 1.66 mmol).

2. **Copy of the ^{1}H and ^{13}C{^{1}H} NMR of arylazosulfones (1ab, 1aj, 1ak, 1am and 1ap) and biaryls 2-43.**

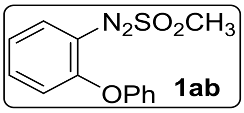

^{1}H-NMR (300 MHz, $CDCl_3$)

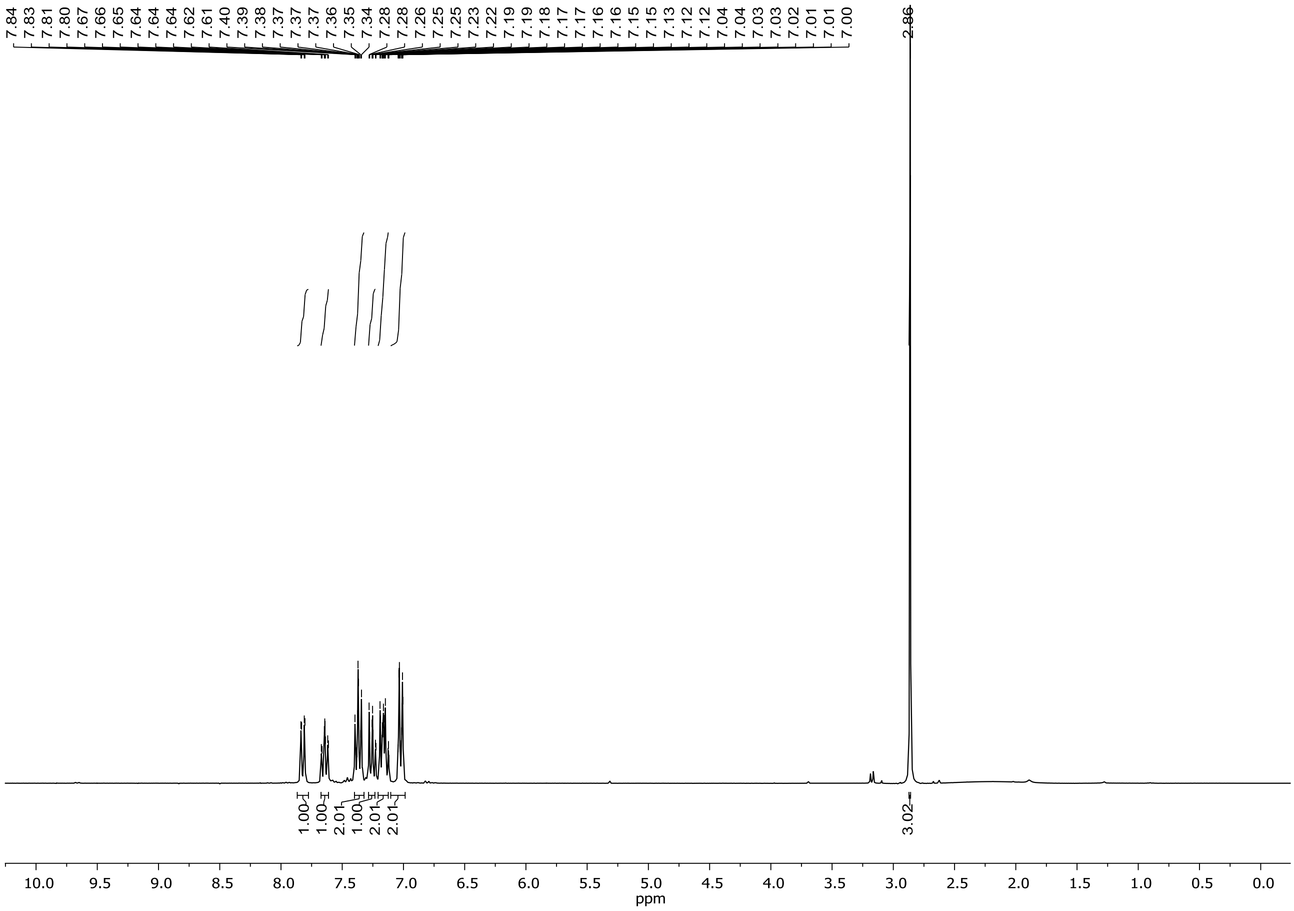

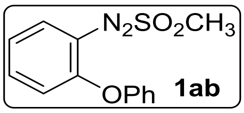

$^{13}C\{^{1}H\}$-NMR (75 MHz, $CDCl_3$)

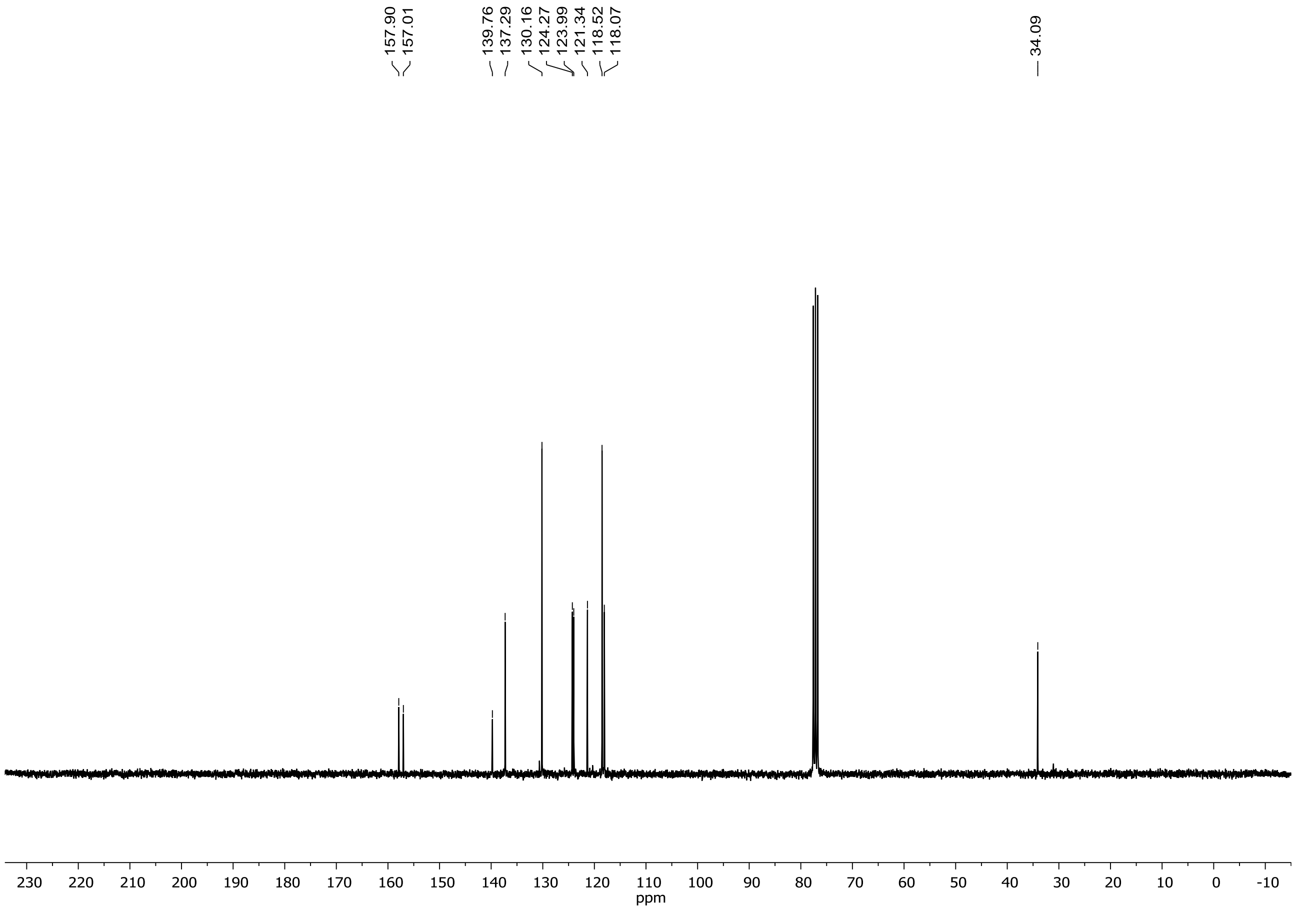

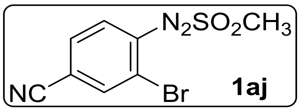

^{1}H-NMR (300 MHz, $CDCl_3$)

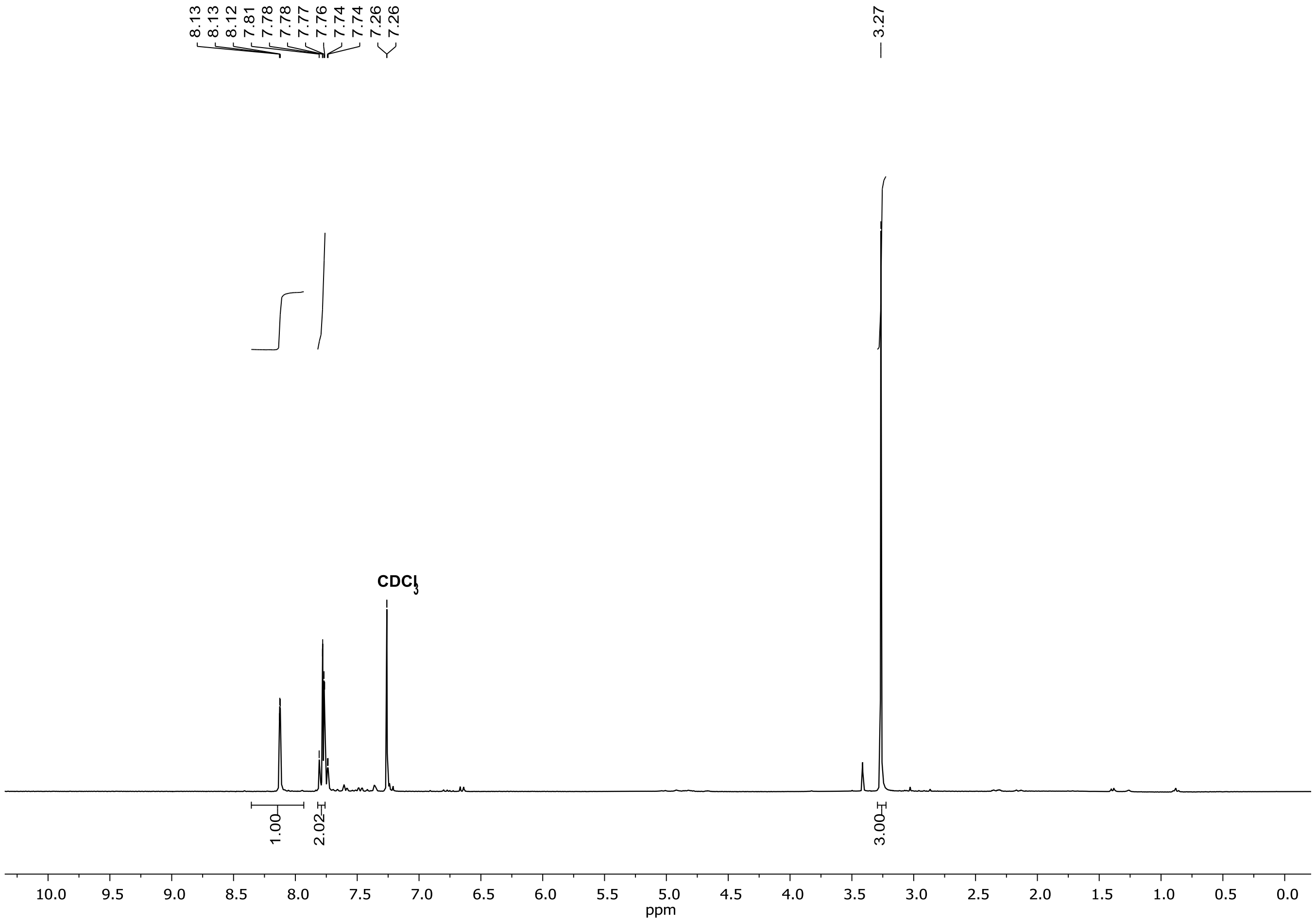

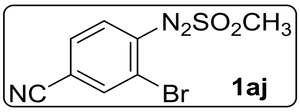

$^{13}C\{^{1}H\}$-NMR (75 MHz, $CDCl_3$)

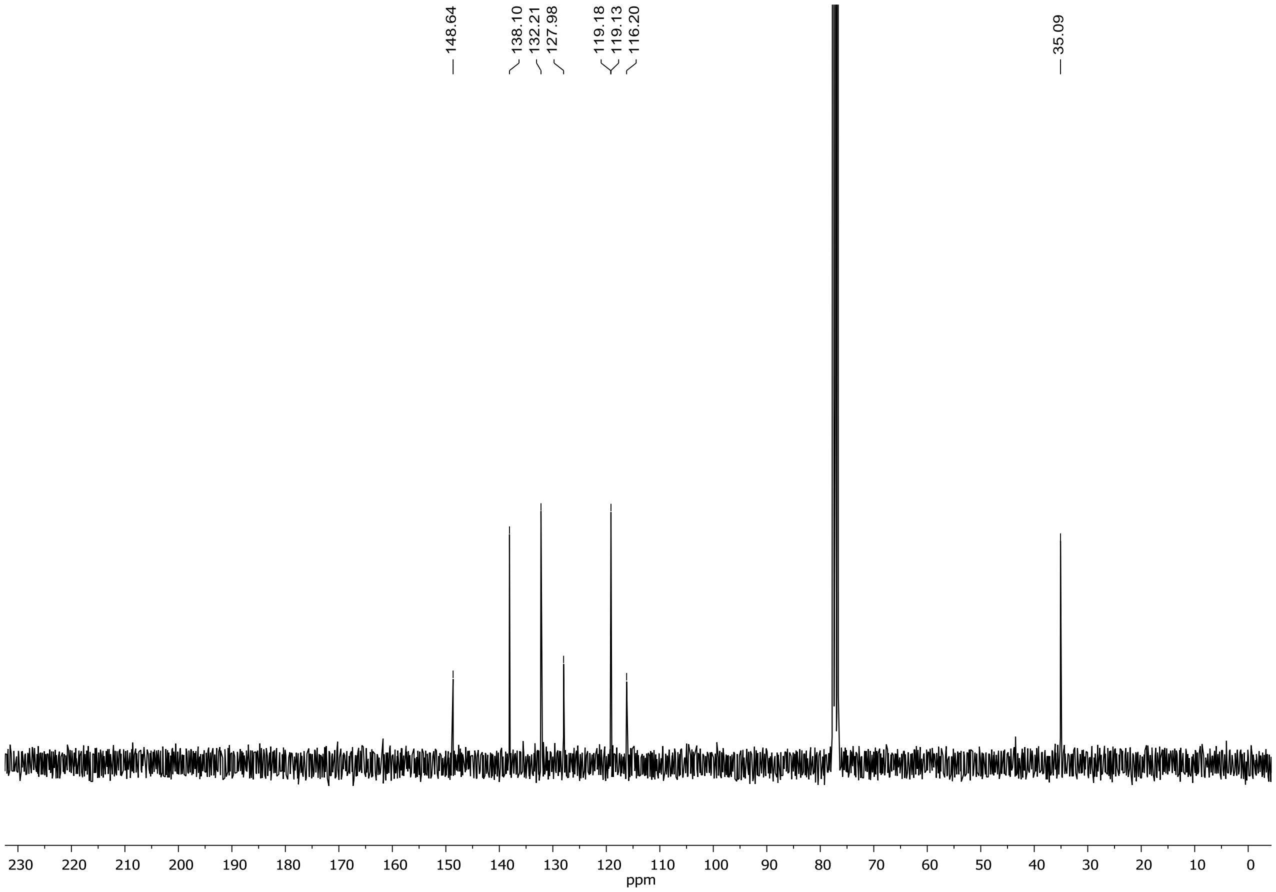

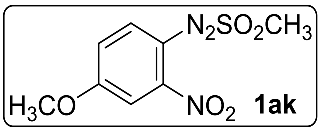

^{1}H-NMR (300 MHz, $CDCl_3$)

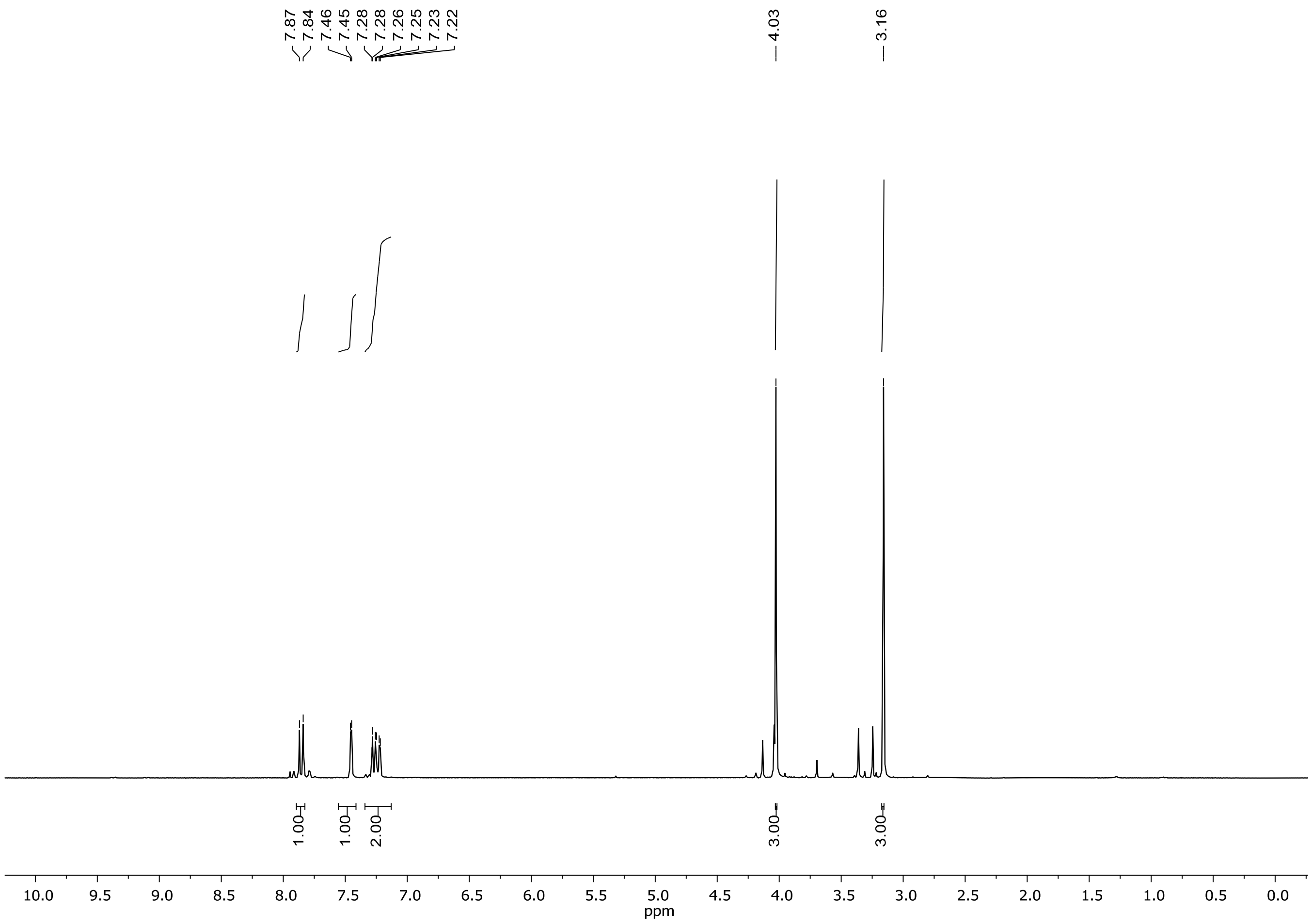

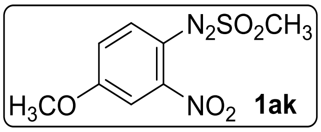

$^{13}C\{^1H\}$-NMR (75 MHz, $CDCl_3$)

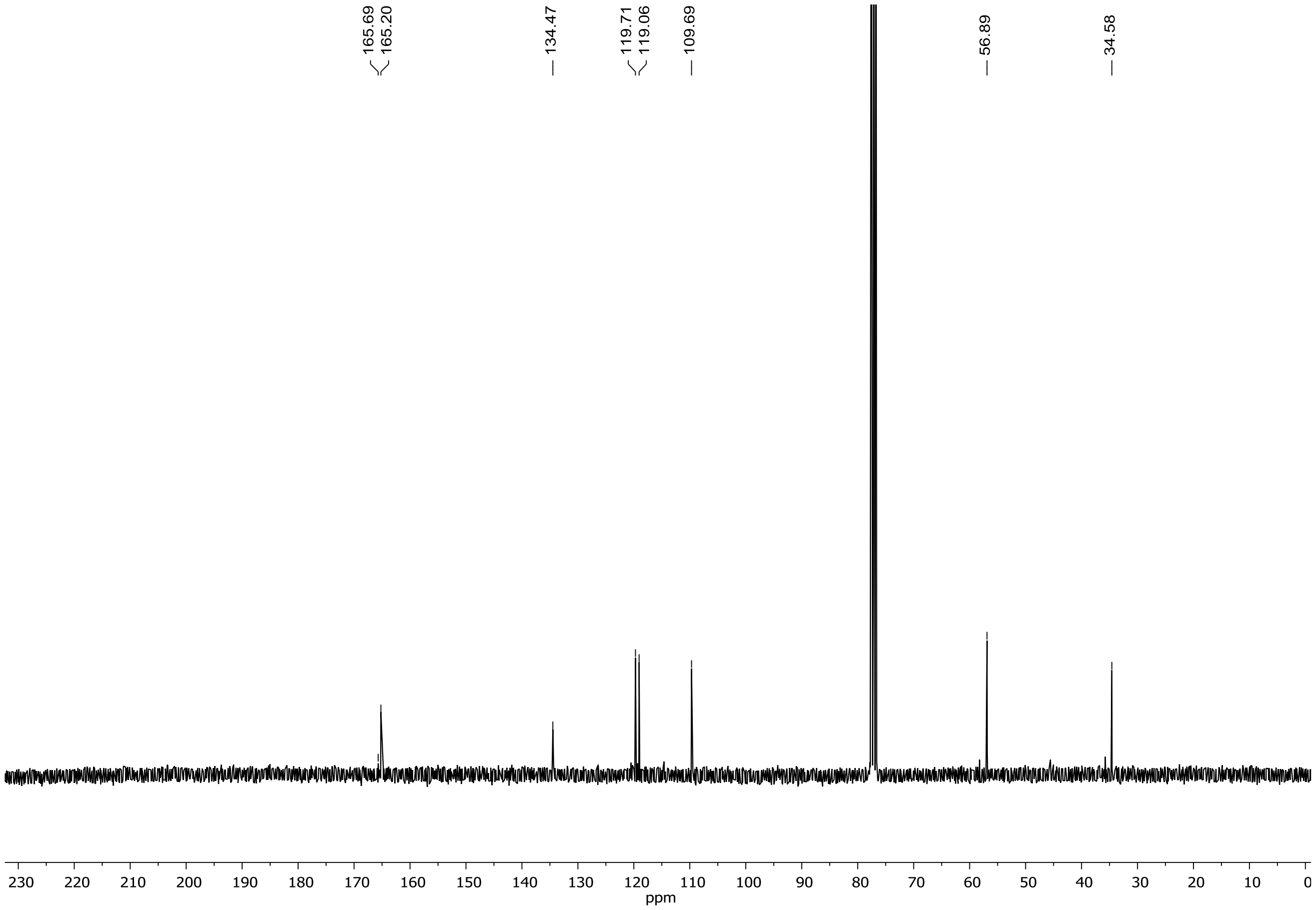

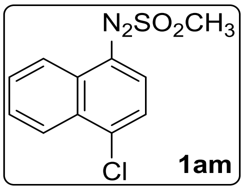

^{1}H-NMR (300 MHz, $CDCl_3$).

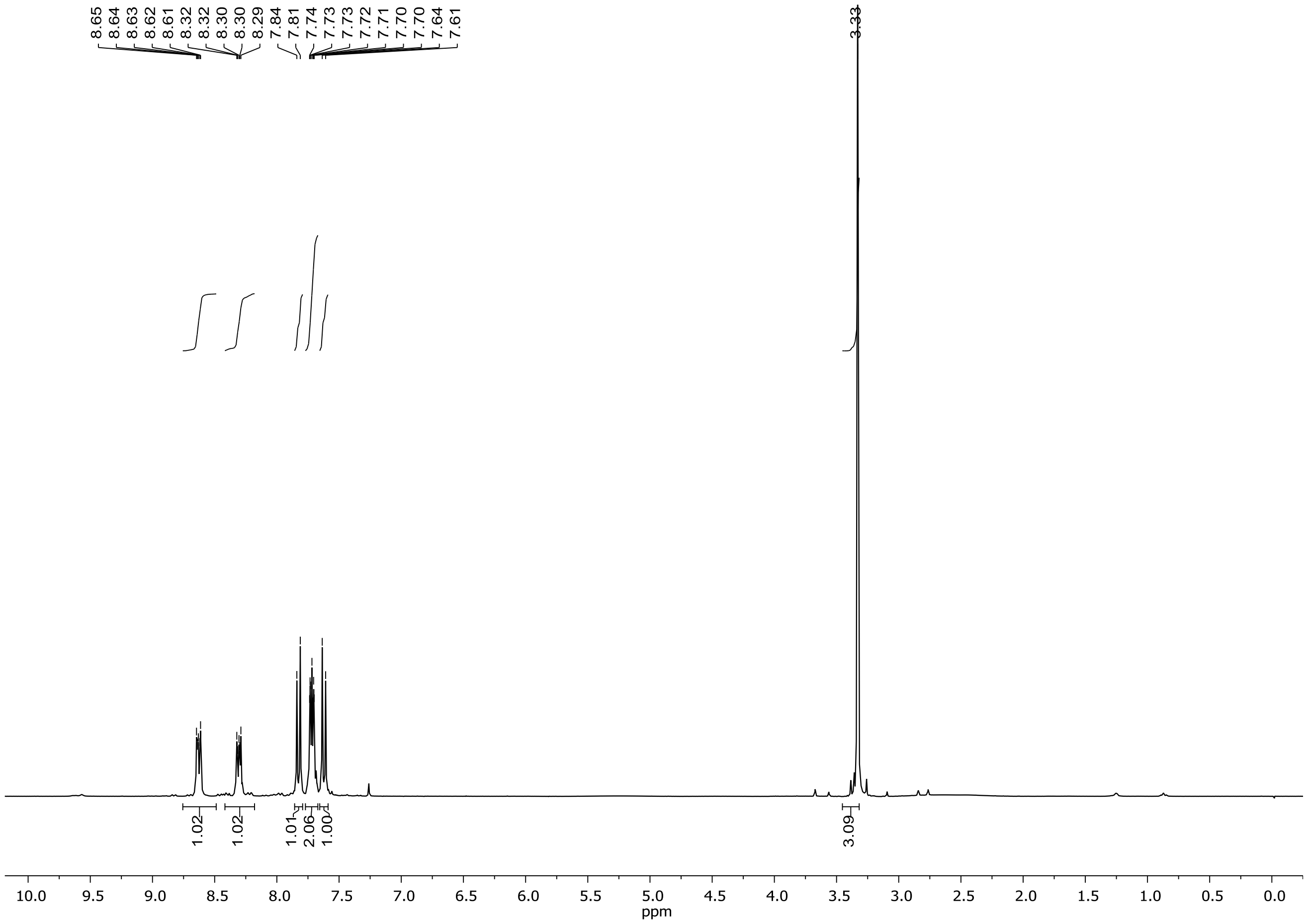

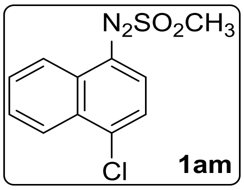

$^{13}C\{^{1}H\}$-NMR (75 MHz, $CDCl_3$)

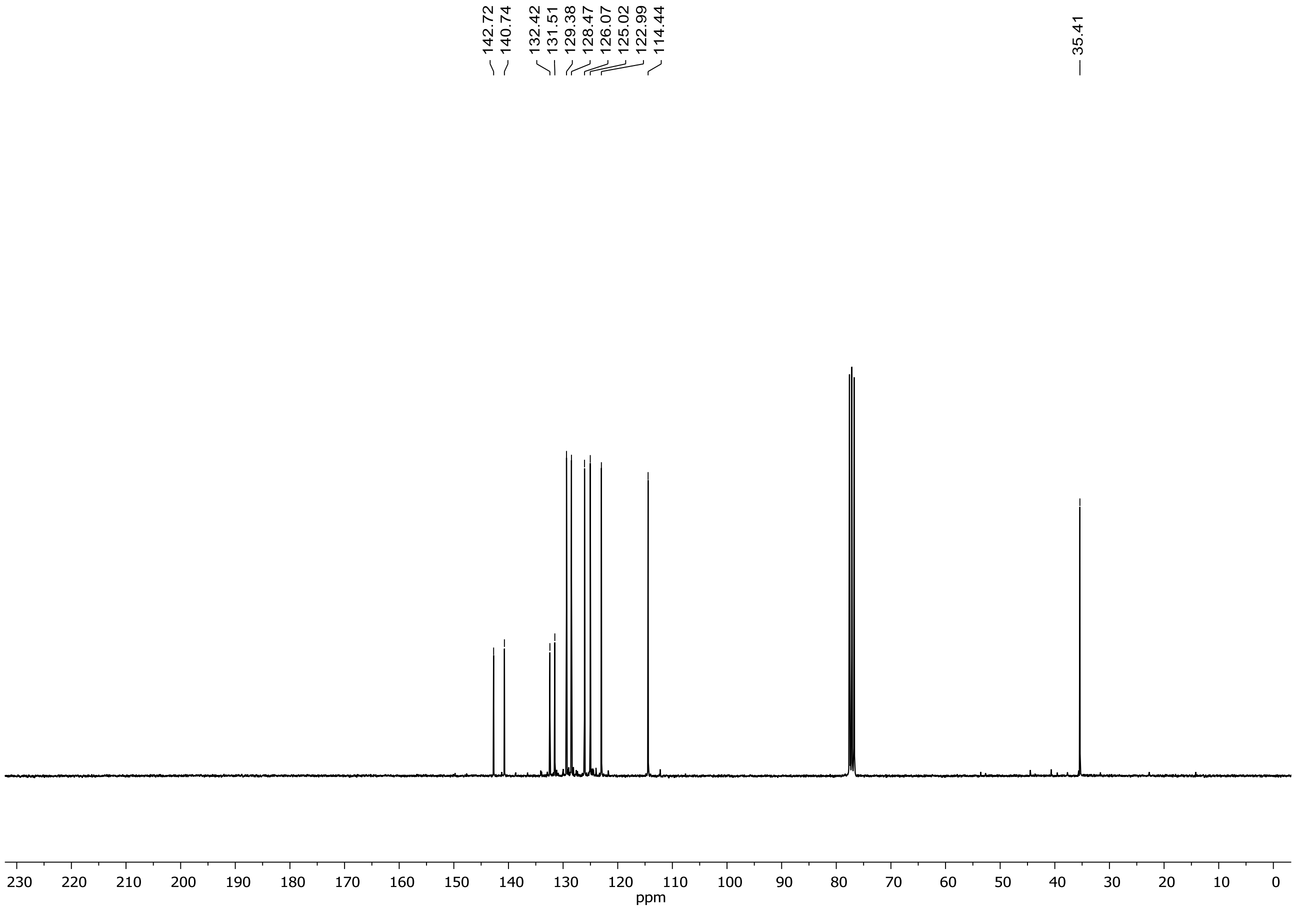

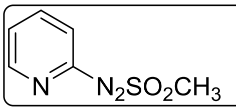

1ap ^{1}H NMR (300 MHz, $CDCl_3$).

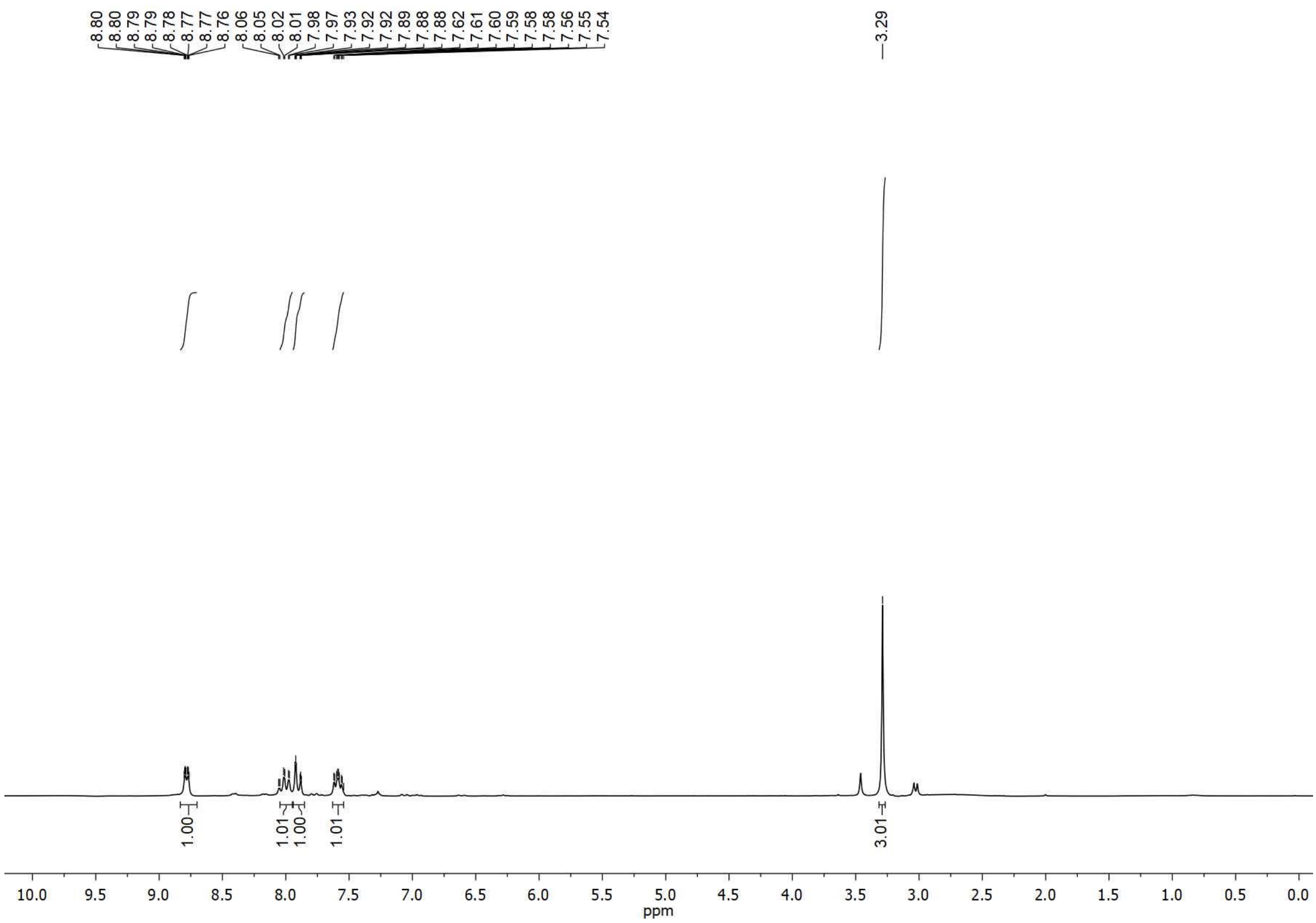

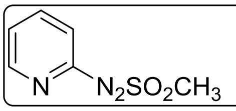

1ap $^{13}C\{^{1}H\}$-NMR (75 MHz, $CDCl_3$) Some peaks belong to the degradation products of **1ap**.

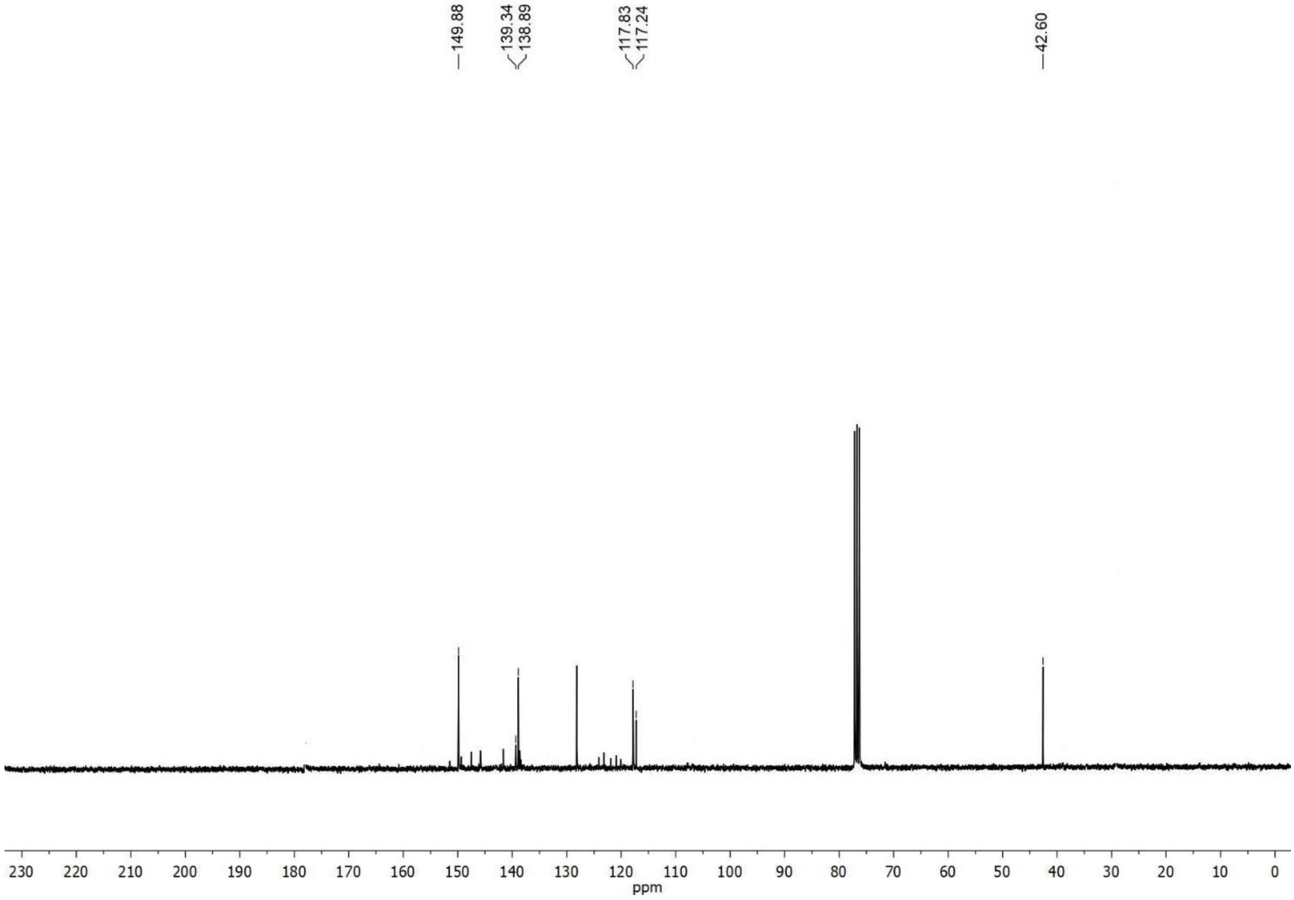

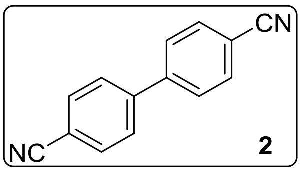

^{1}H-NMR (300 MHz, $CDCl_3$)

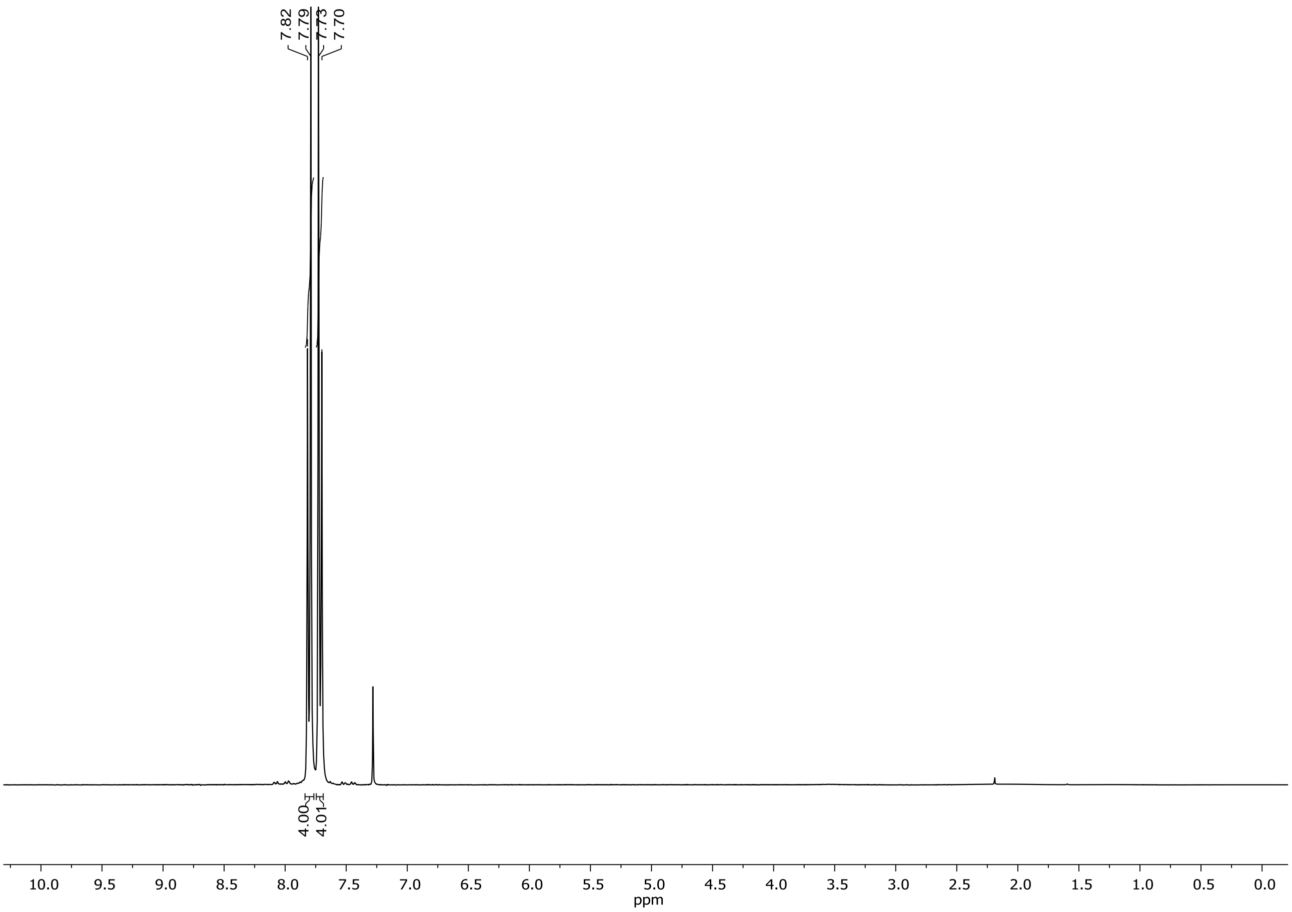

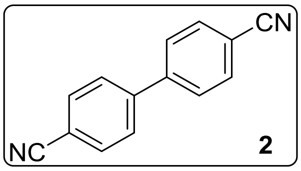

2

$^{13}C\{^1H\}$-NMR (75 MHz, $CDCl_3$)

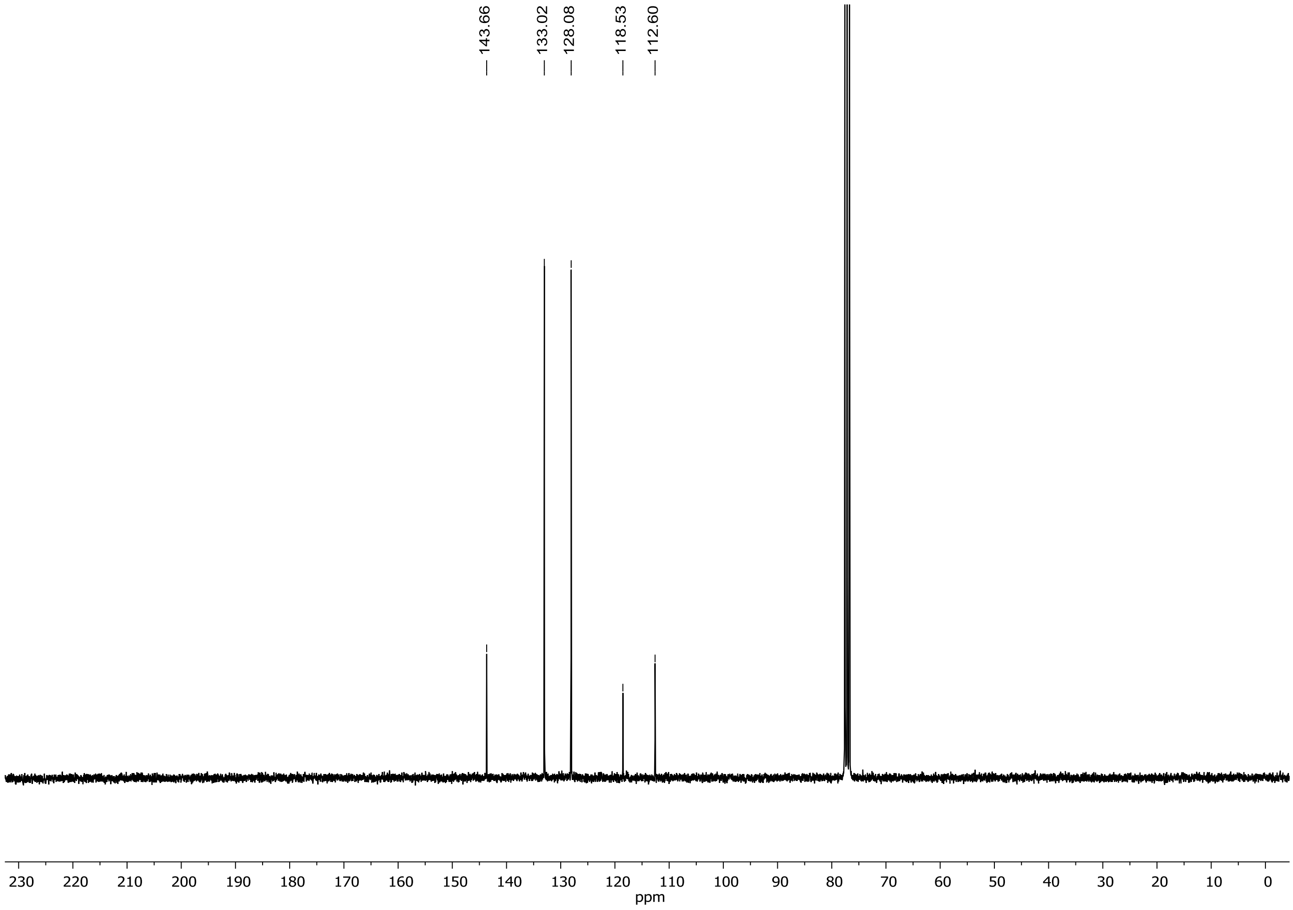

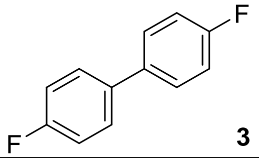

3

^{1}H-NMR (300 MHz, $CDCl_3$)

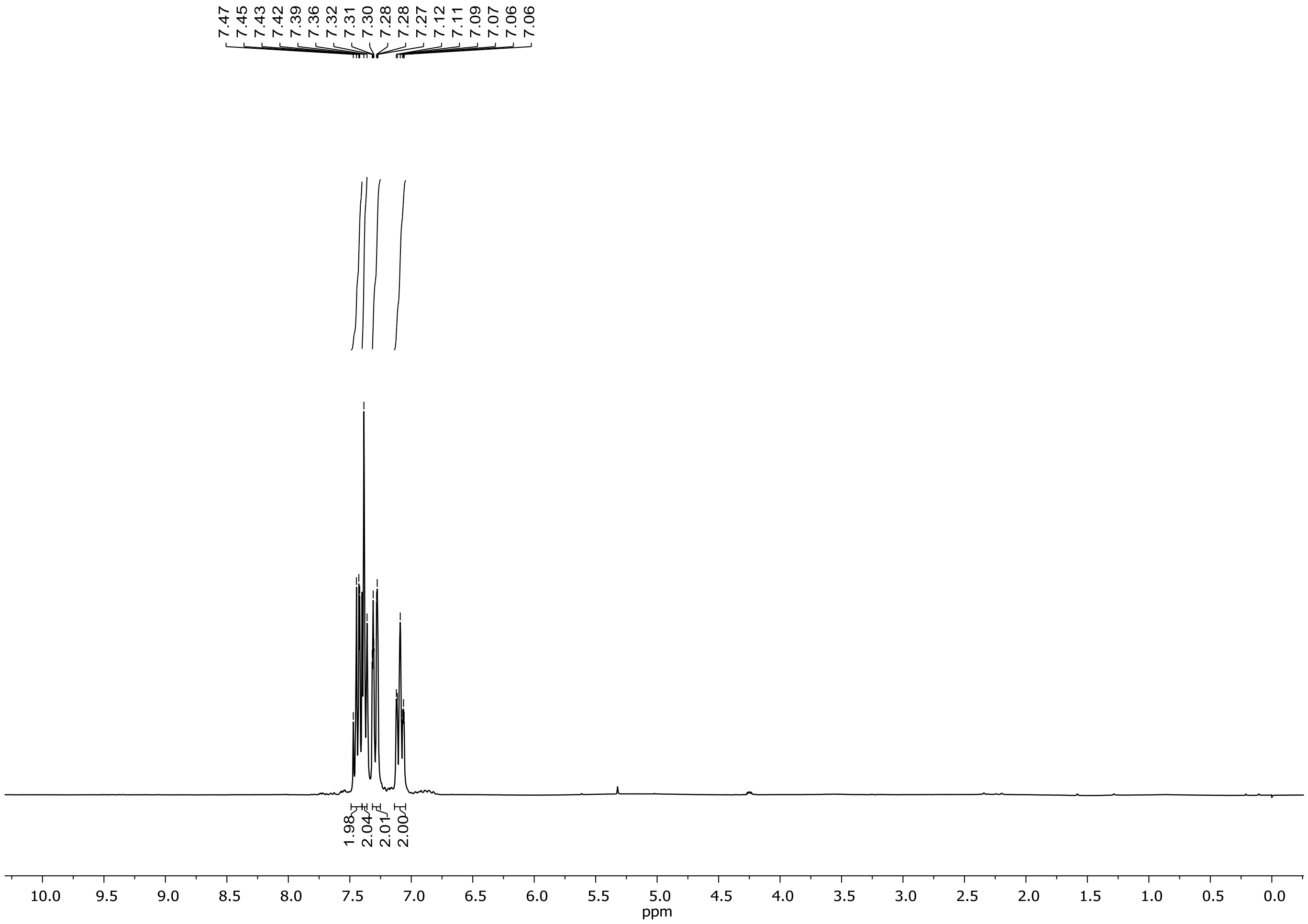

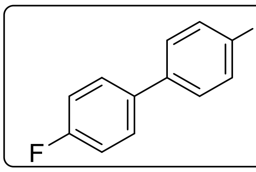

3

$^{13}C\{^{1}H\}$-NMR (75 MHz, $CDCl_3$)

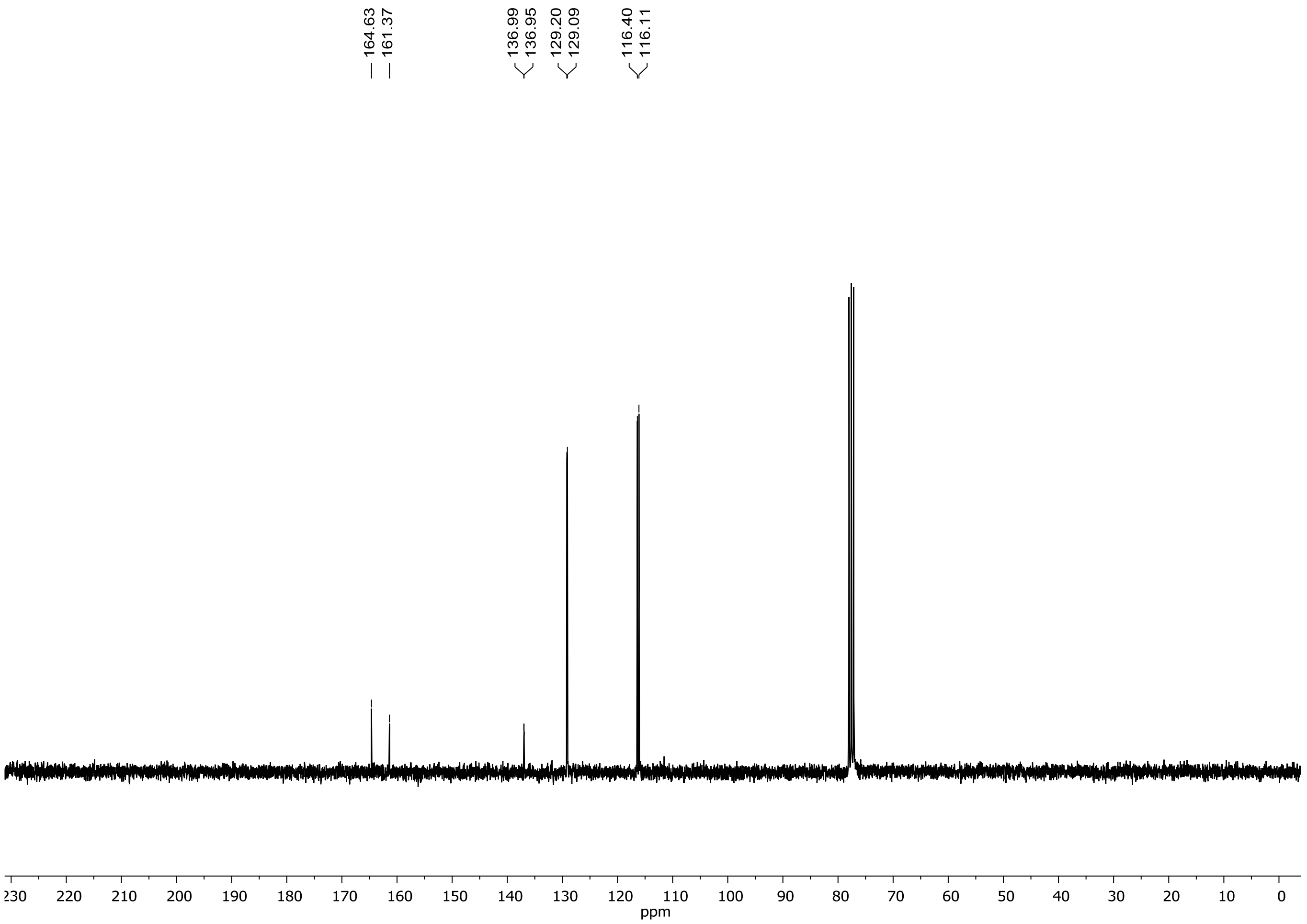

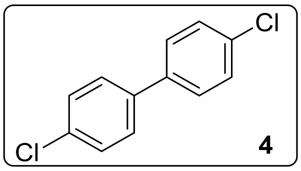

4

^{1}H-NMR (300 MHz, $CDCl_3$)

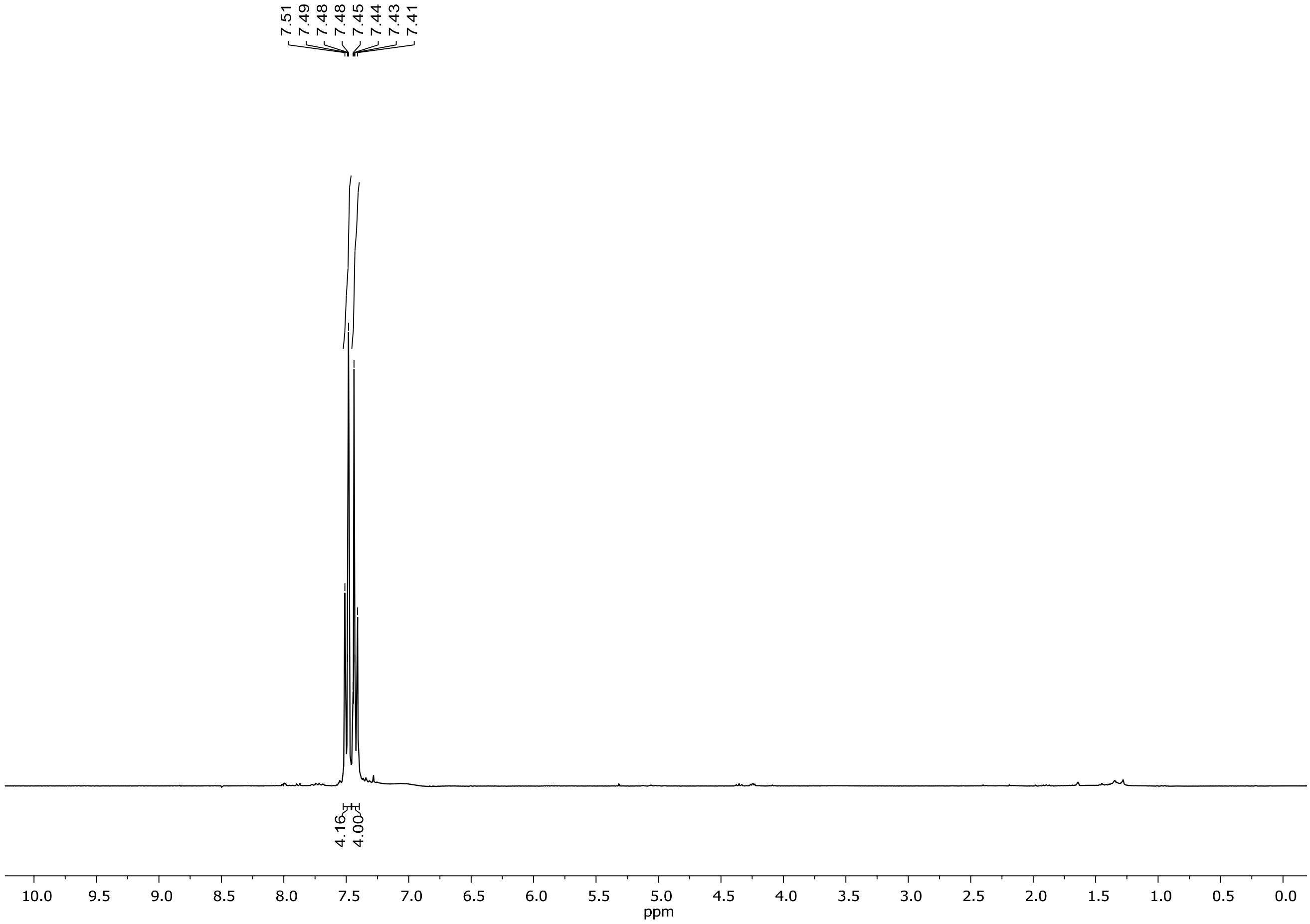

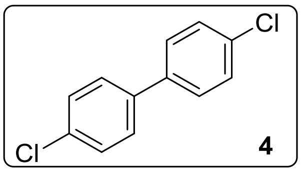

4

$^{13}C\{^{1}H\}$-NMR (75 MHz, $CDCl_3$)

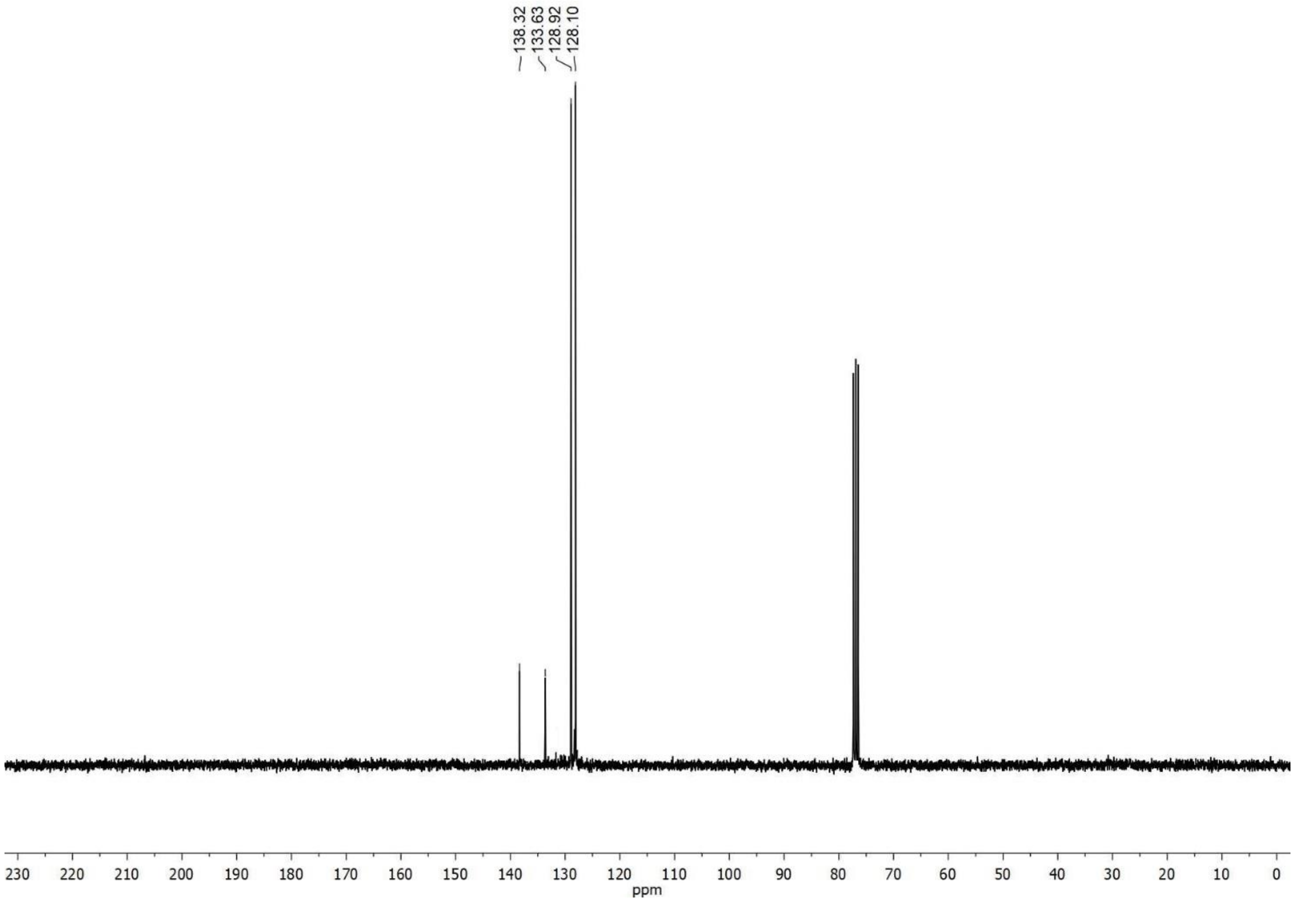

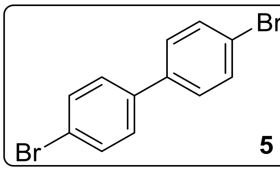

5

^{1}H-NMR (300 MHz, $CDCl_3$)

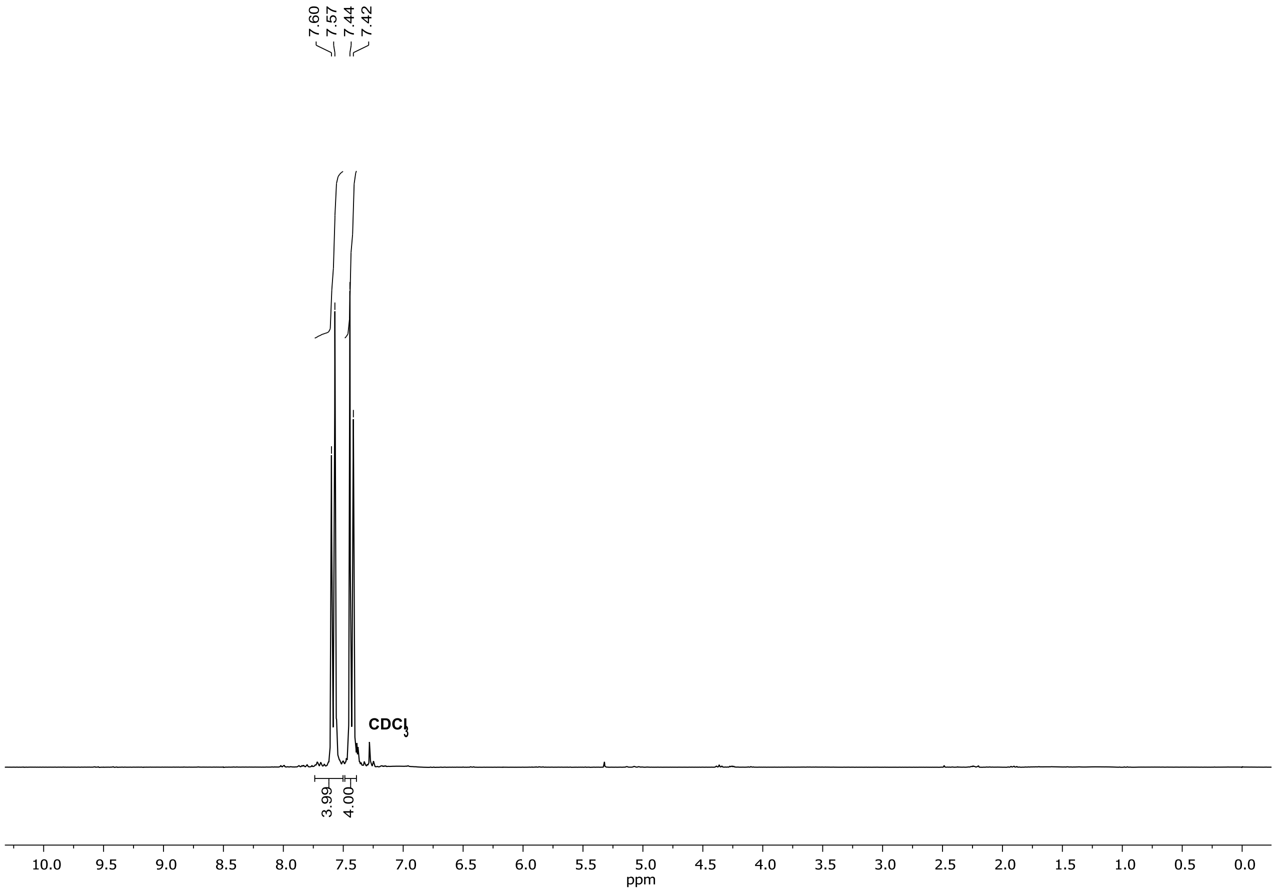

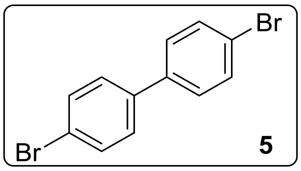

5

$^{13}C\{^{1}H\}$-NMR (75 MHz, $CDCl_3$)

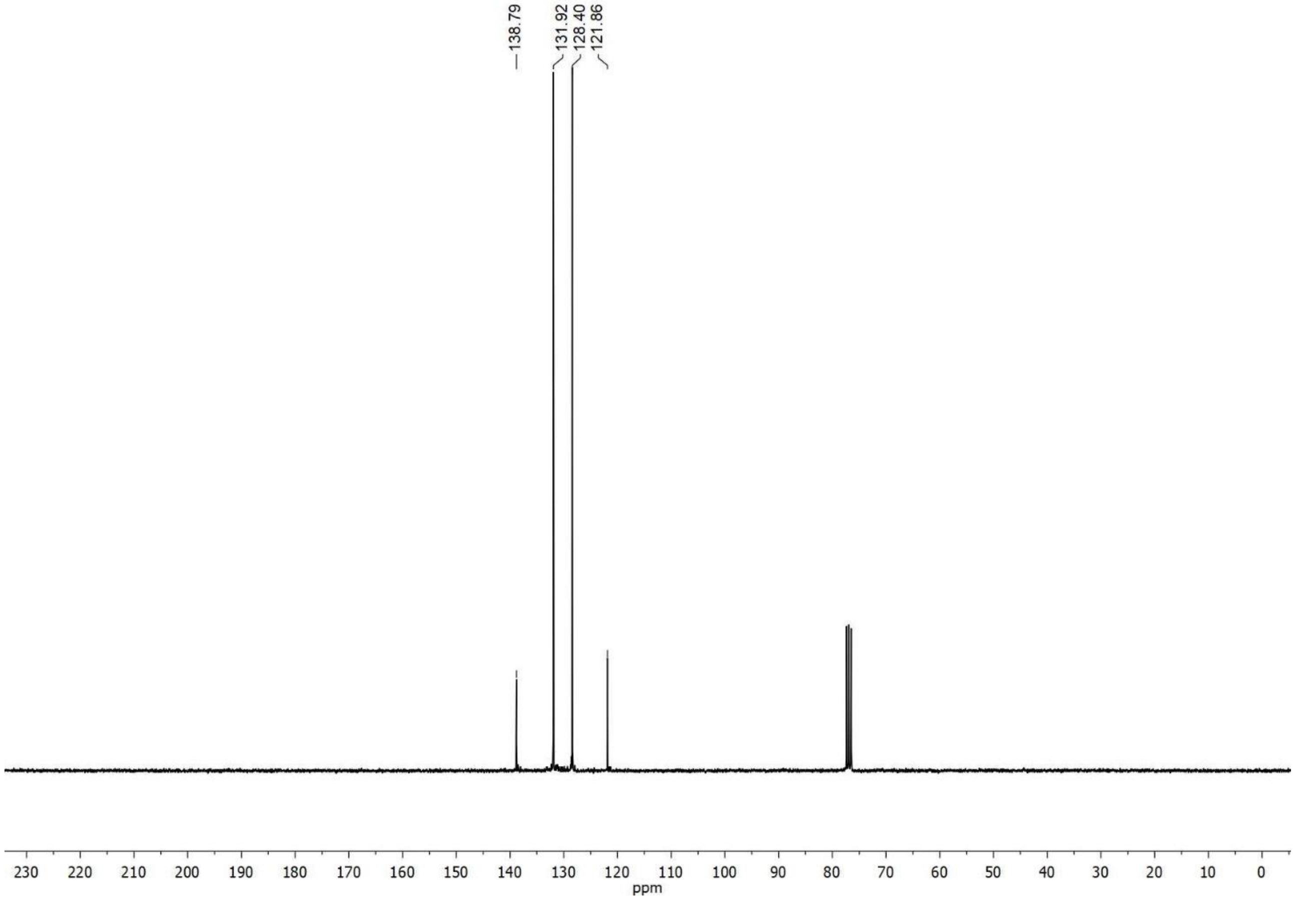

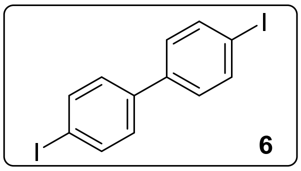

^{1}H-NMR (300 MHz, $CDCl_3$)

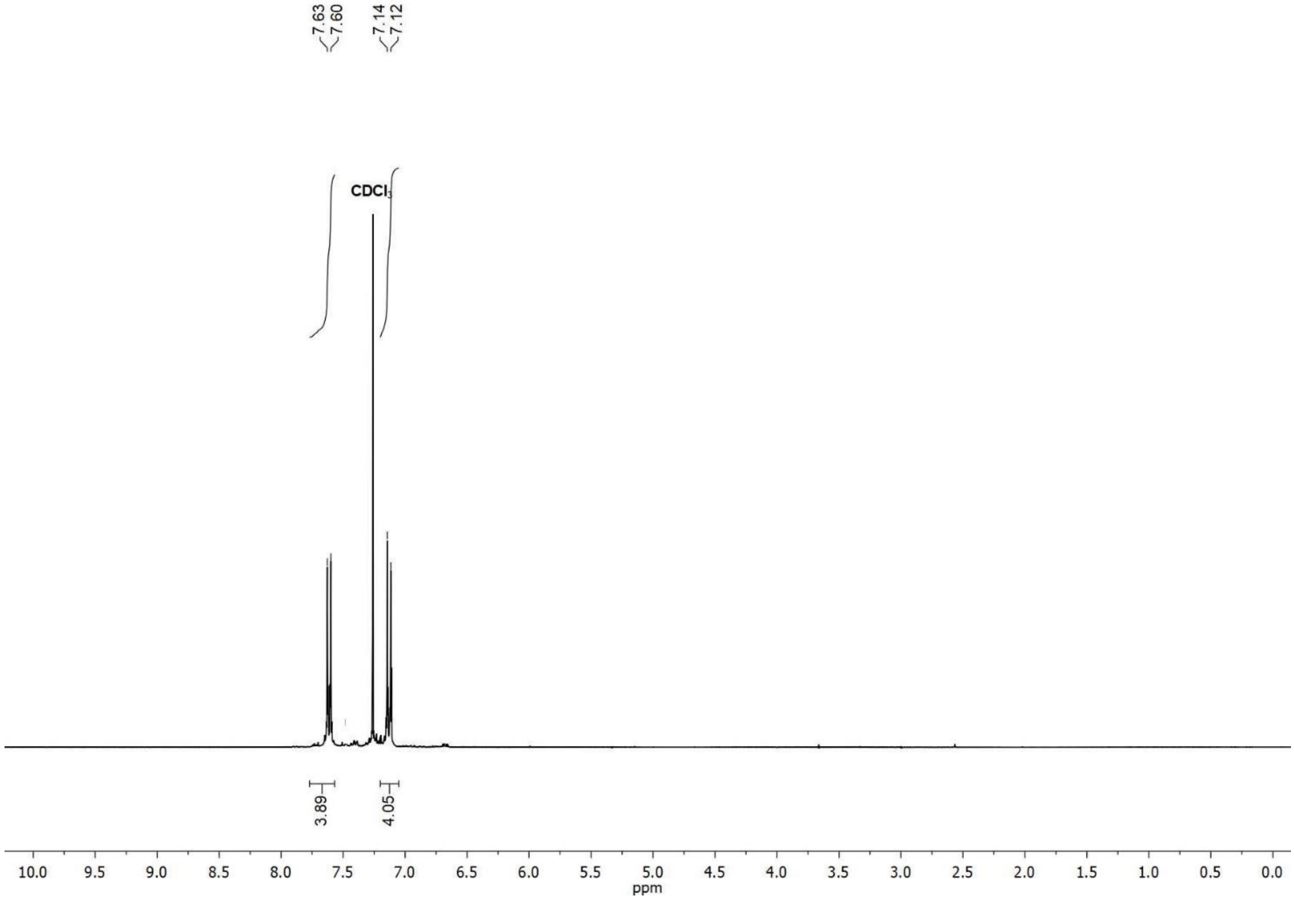

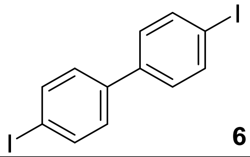

6

$^{13}C\{^{1}H\}$-NMR (75 MHz, $CDCl_3$)

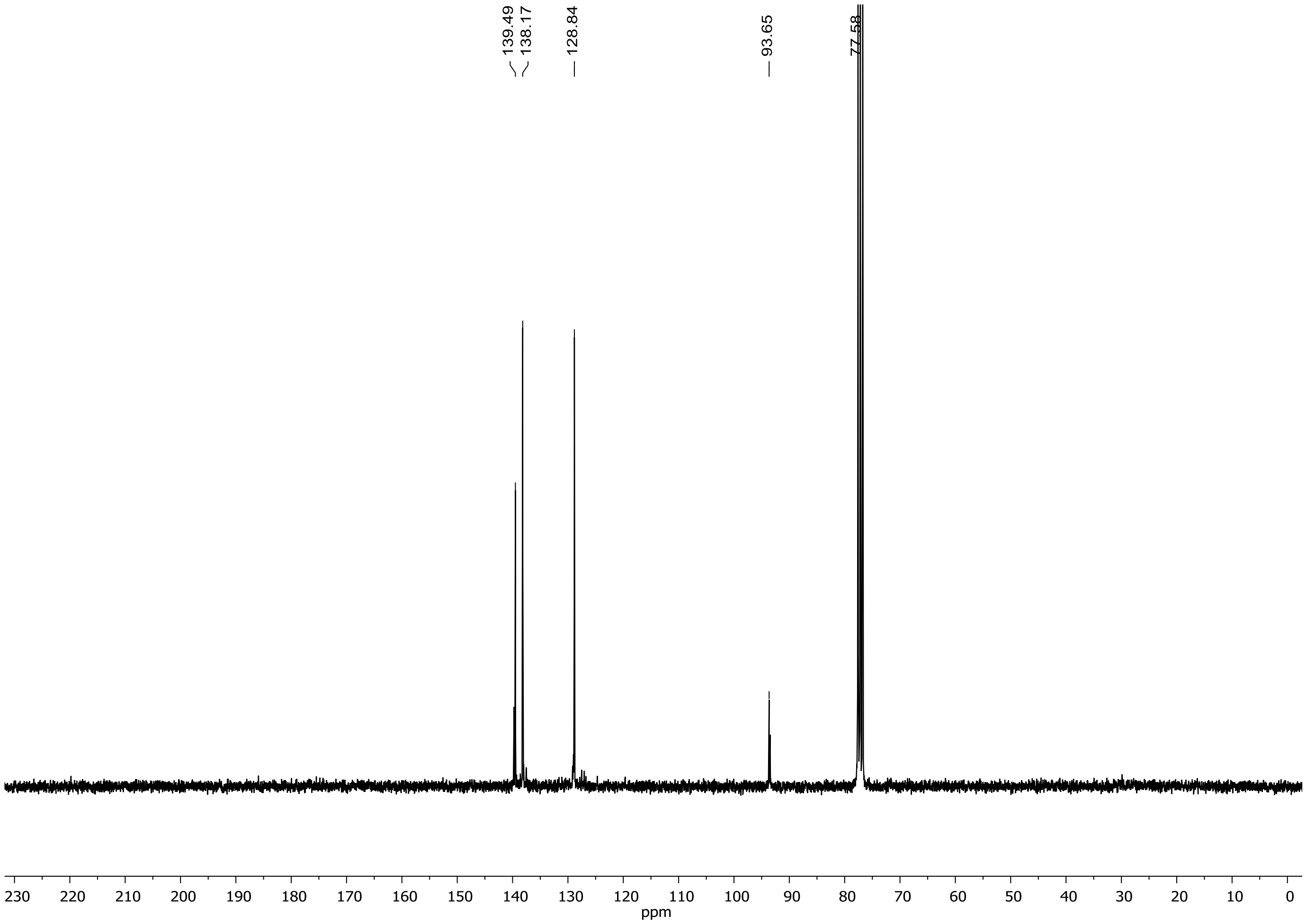

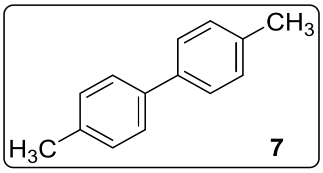

7

^{1}H-NMR (300 MHz, $CDCl_3$)

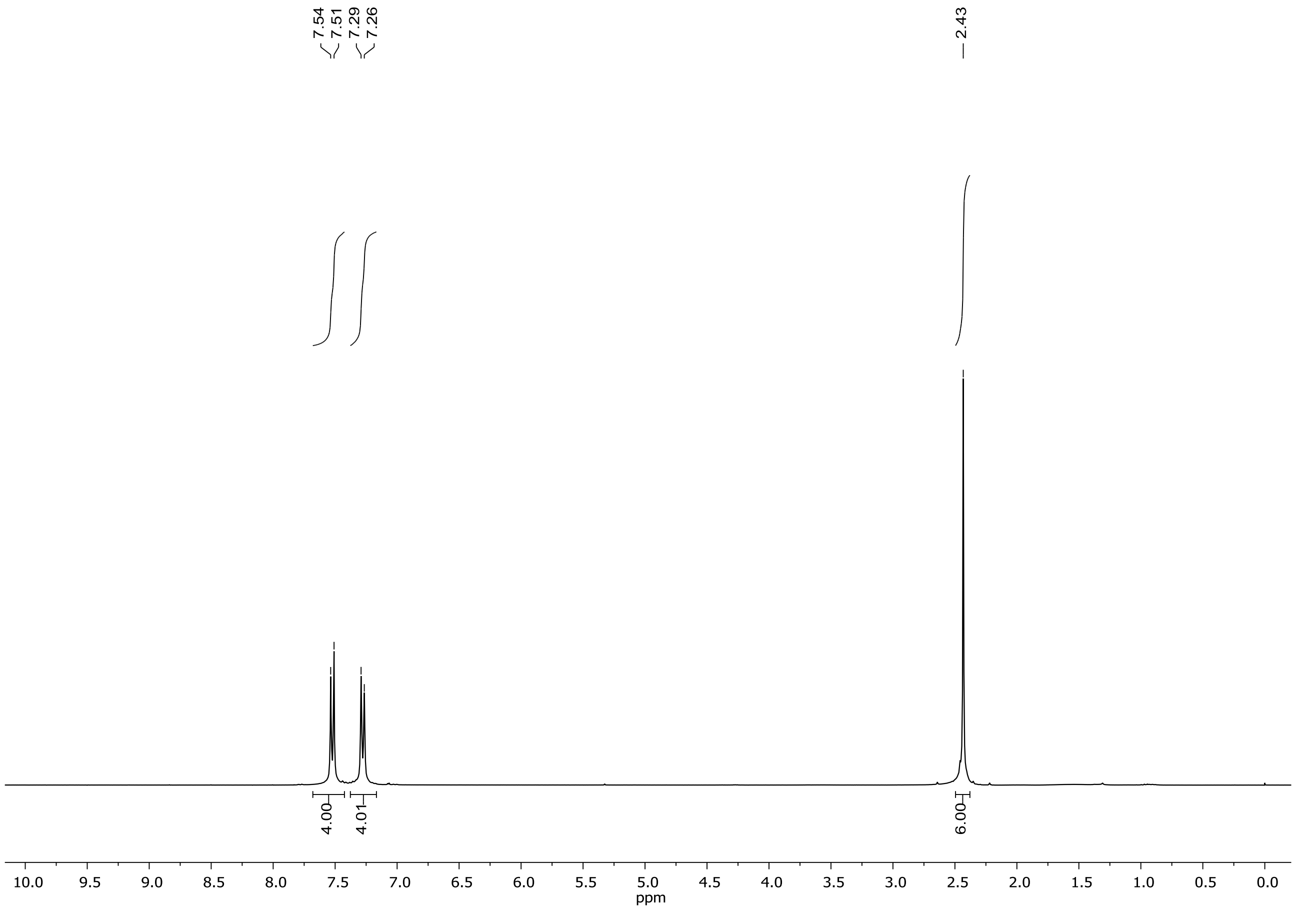

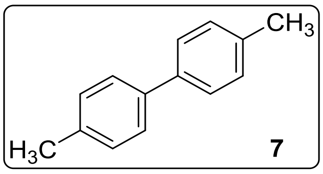

7

$^{13}C\{^{1}H\}$-NMR (75 MHz, $CDCl_3$))

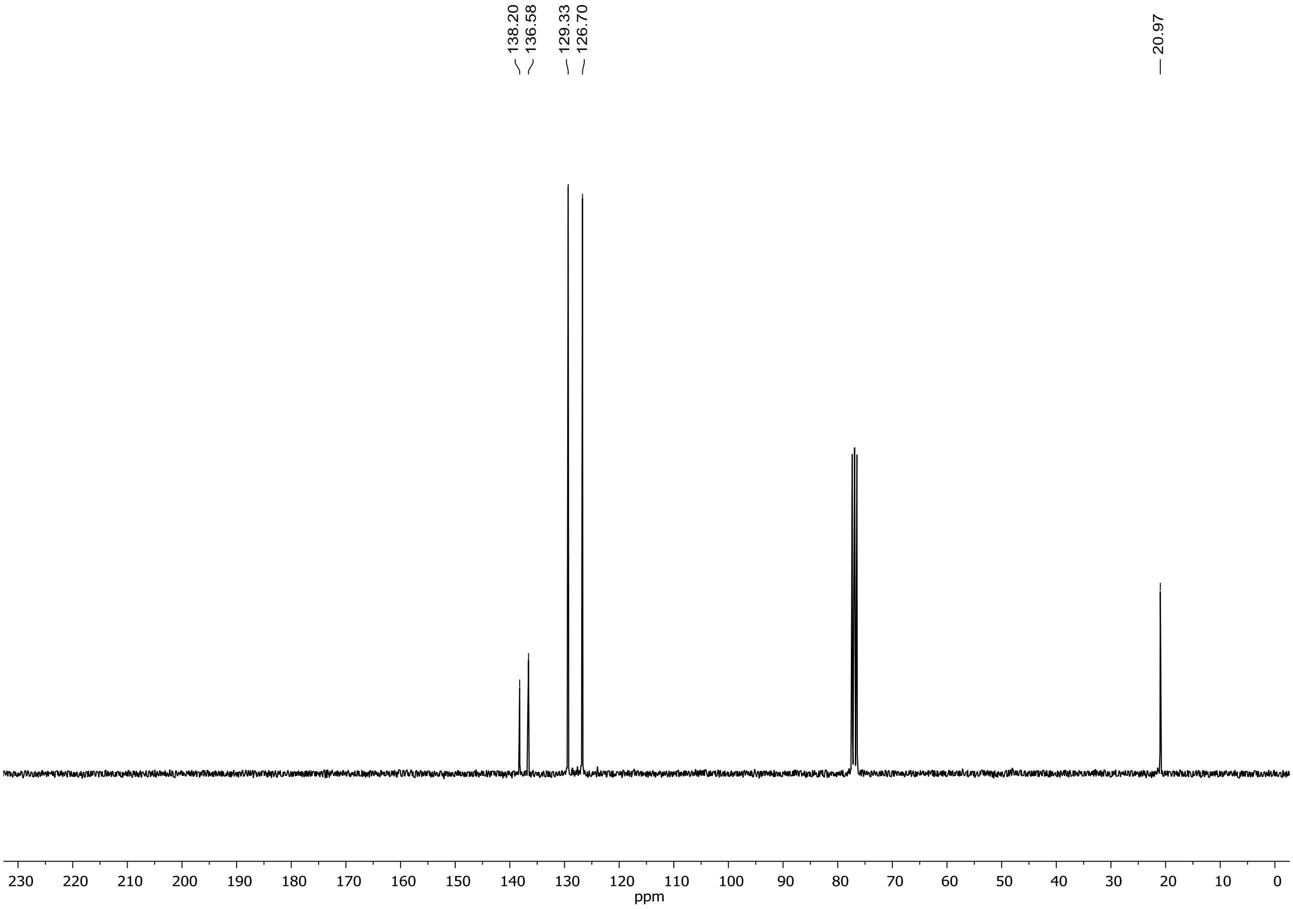

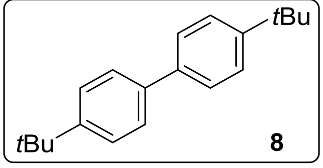

8

^{1}H-NMR (300 MHz, $CDCl_3$)

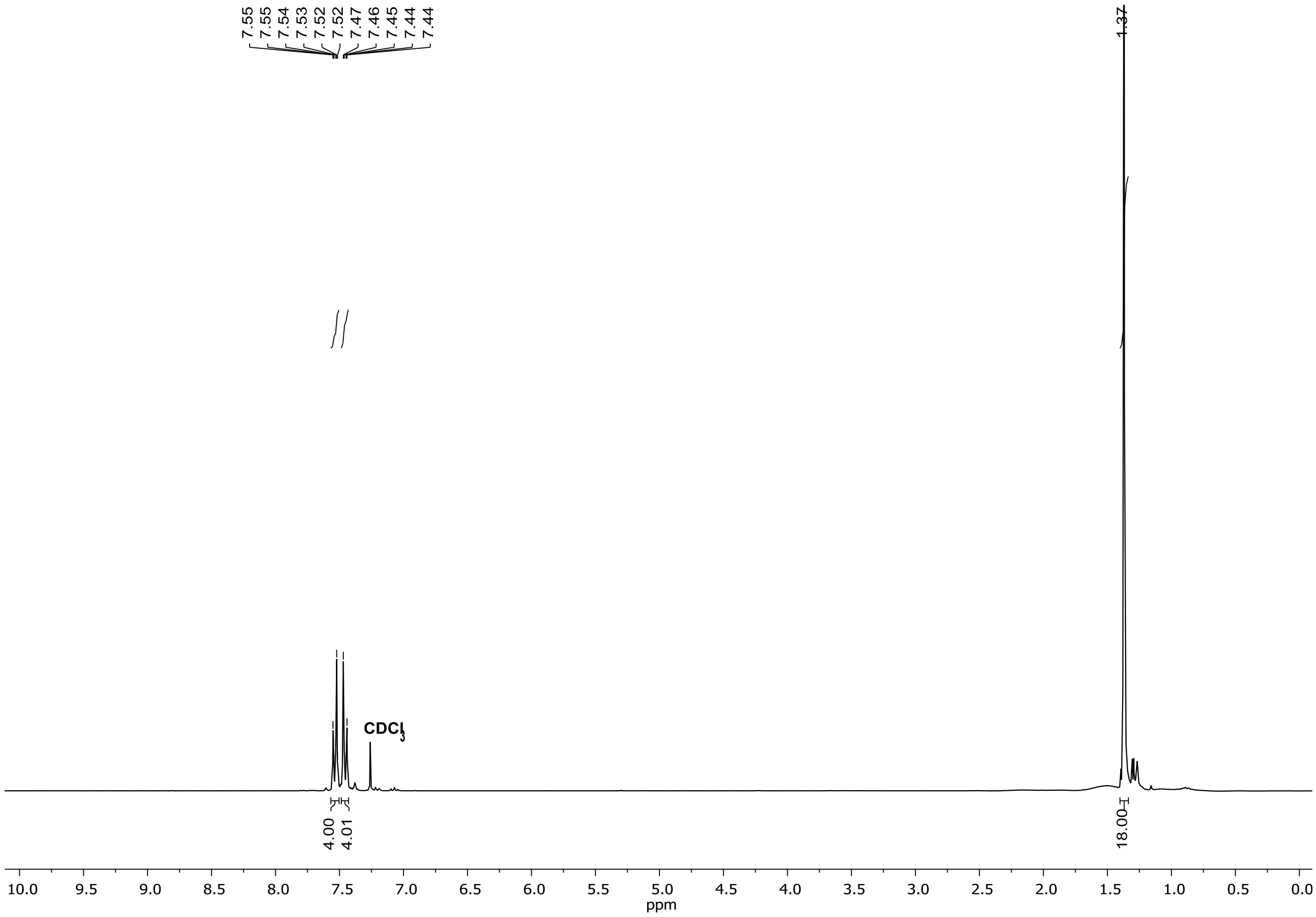

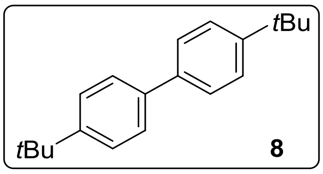

$^{13}C\{^{1}H\}$-NMR (75 MHz, $CDCl_3$)

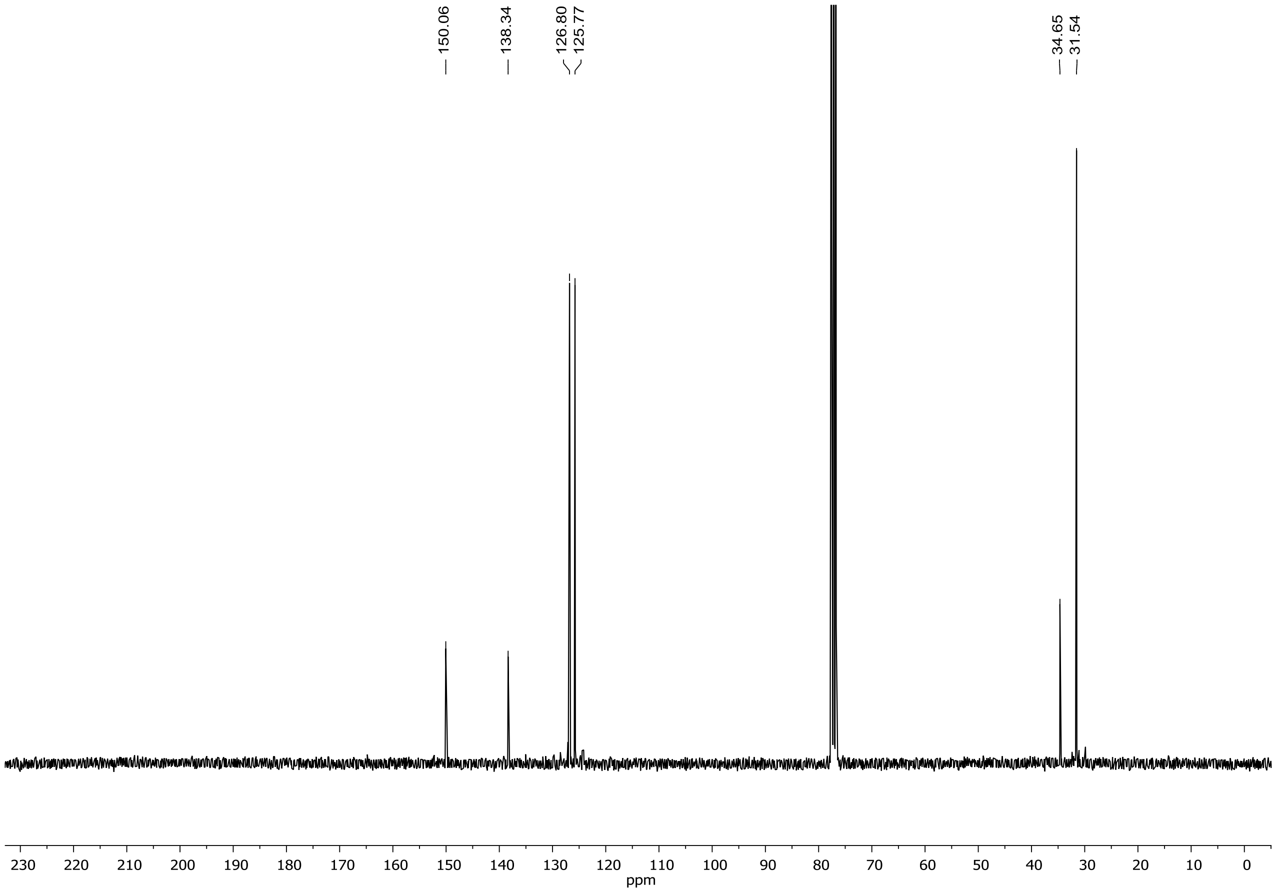

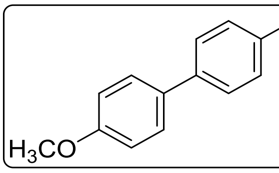

10

^{1}H-NMR (300 MHz, $CDCl_3$)

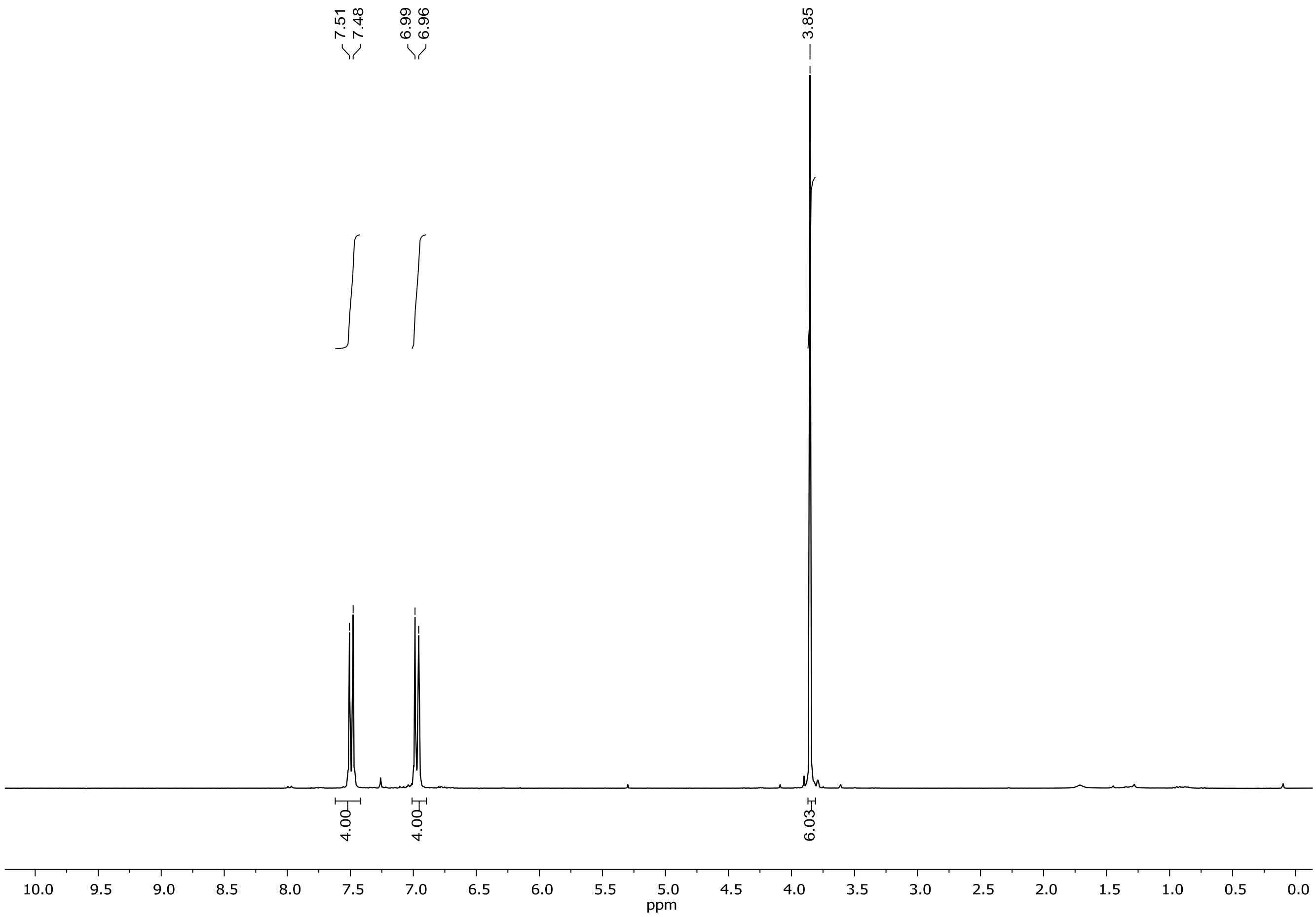

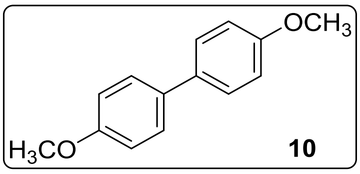

$^{13}C\{^{1}H\}$-NMR (75 MHz, $CDCl_3$)

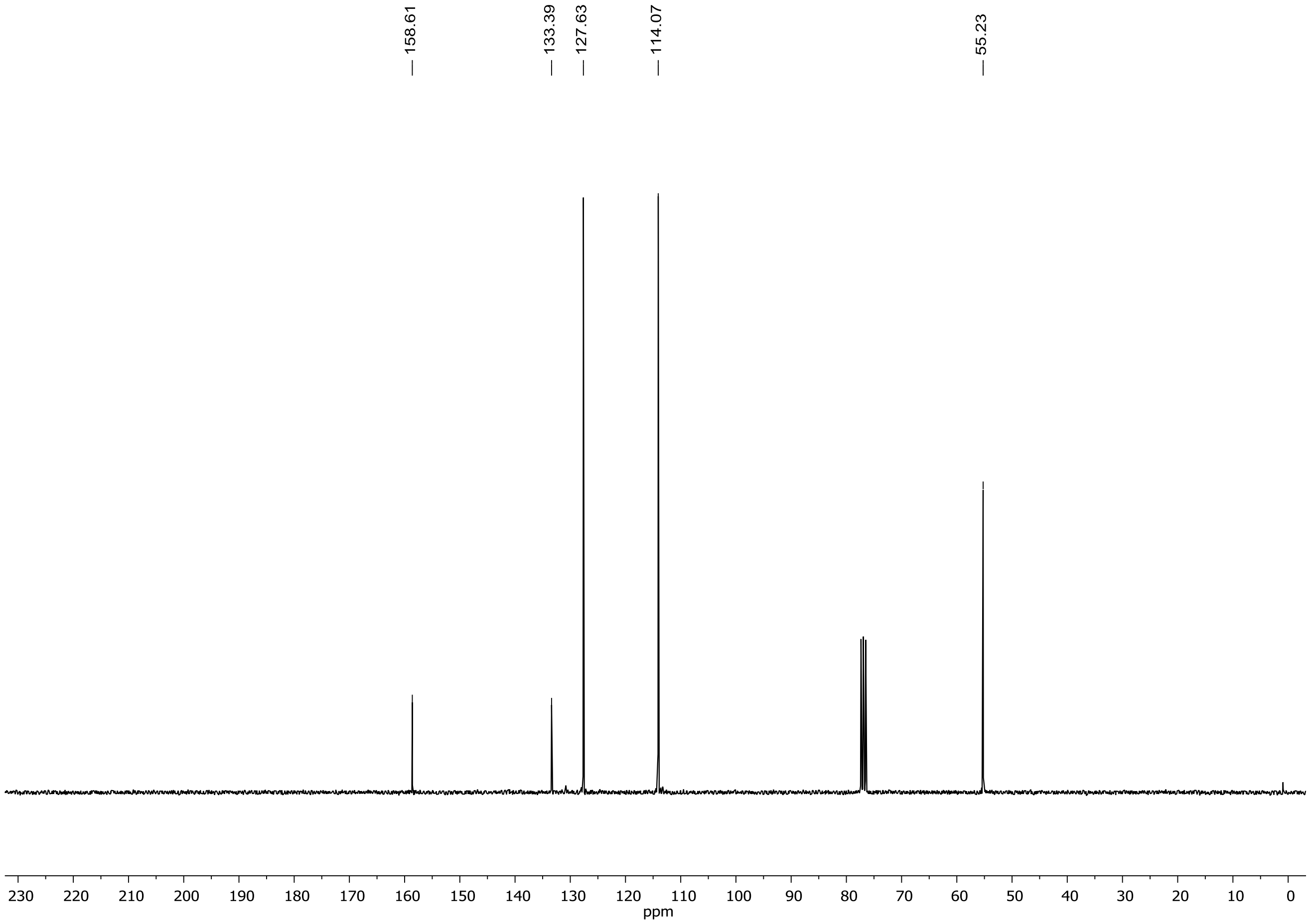

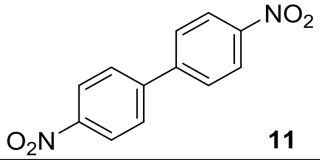

11

^{1}H-NMR (300 MHz, $CDCl_3$)

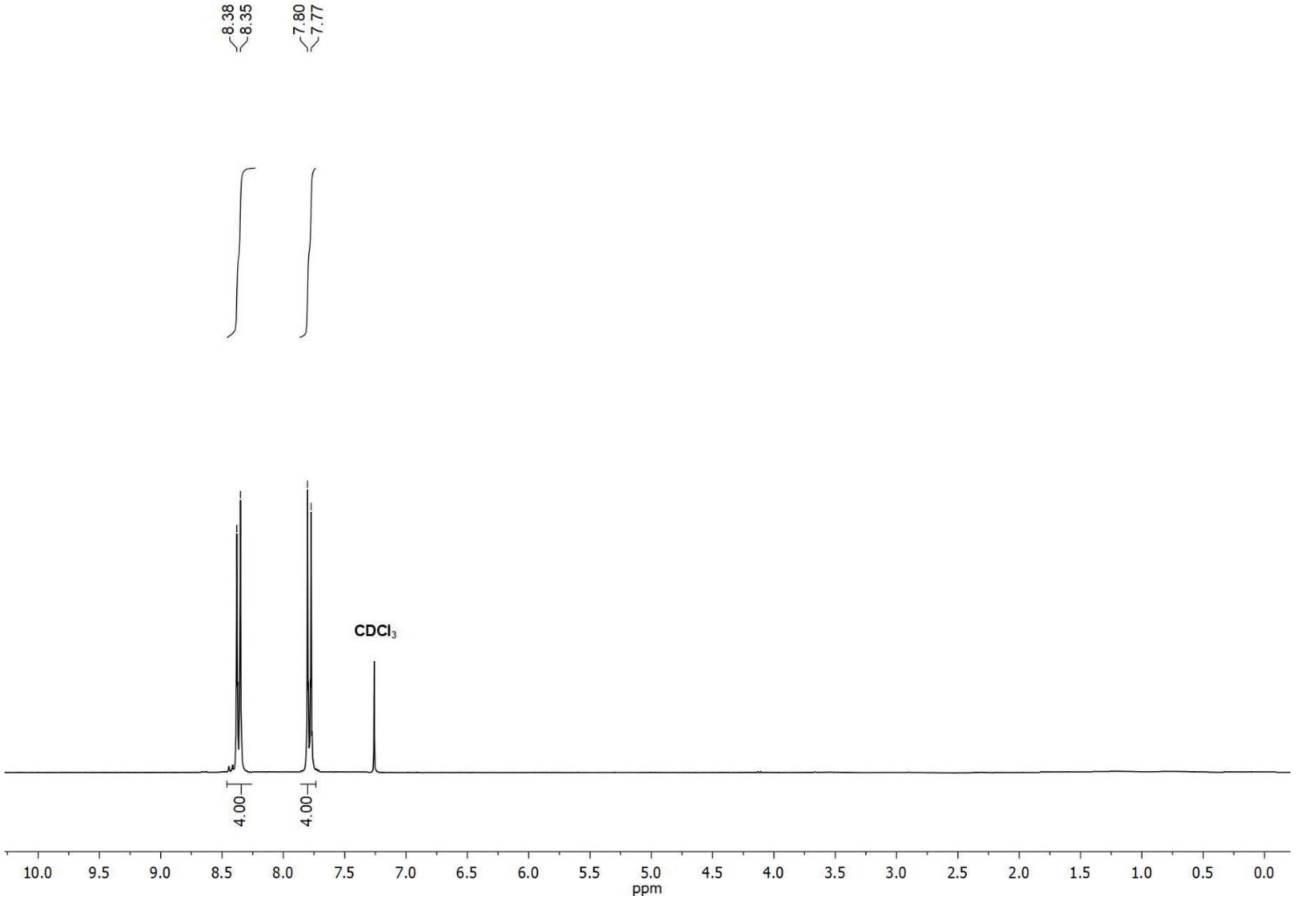

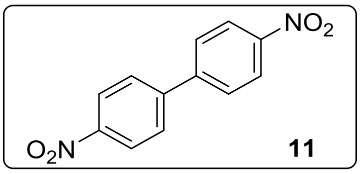

11

$^{13}C\{^{1}H\}$-NMR (75 MHz, $CDCl_3$)

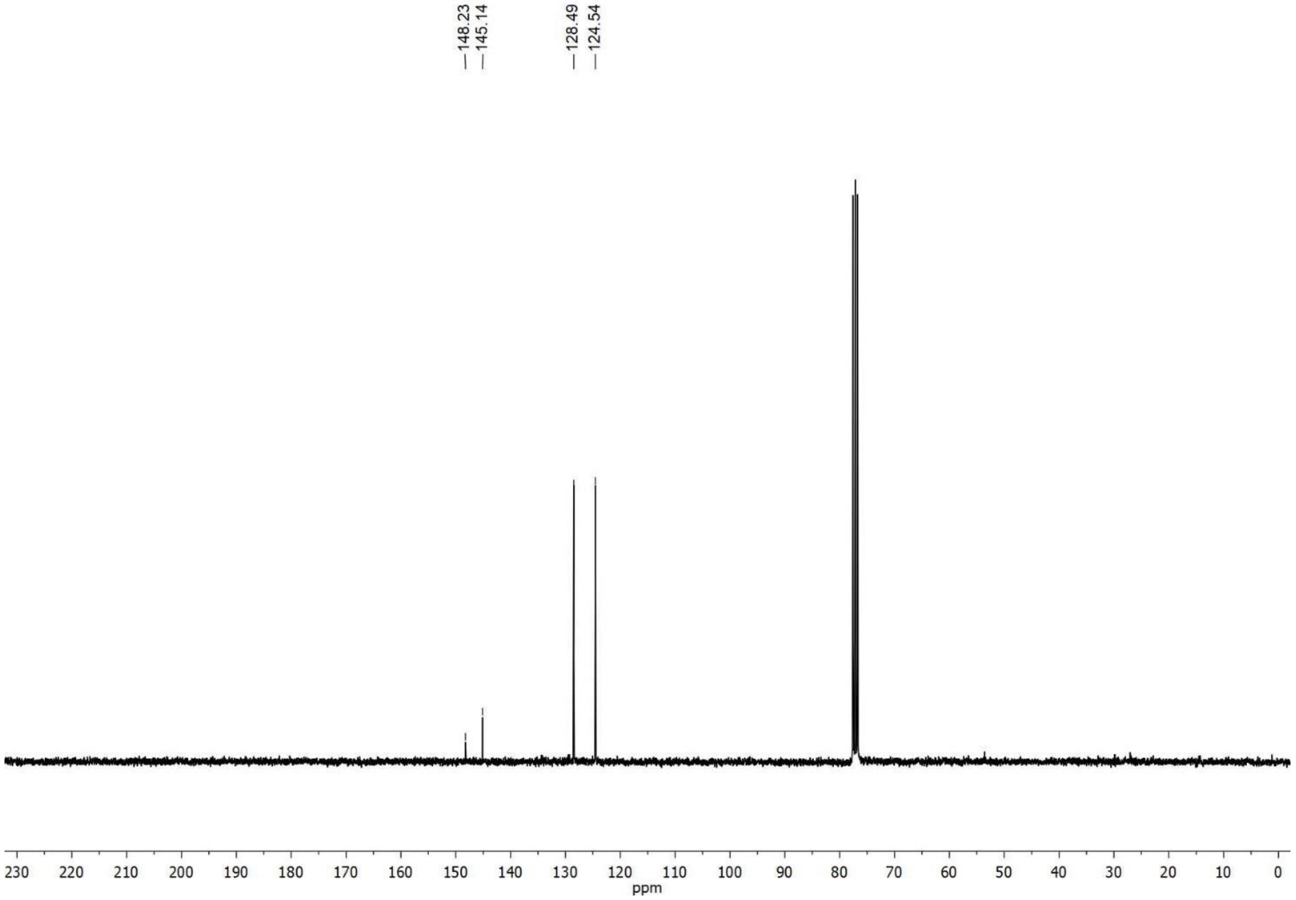

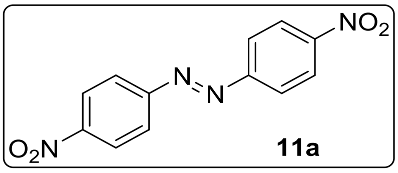

^{1}H-NMR (300 MHz, $CDCl_3$)

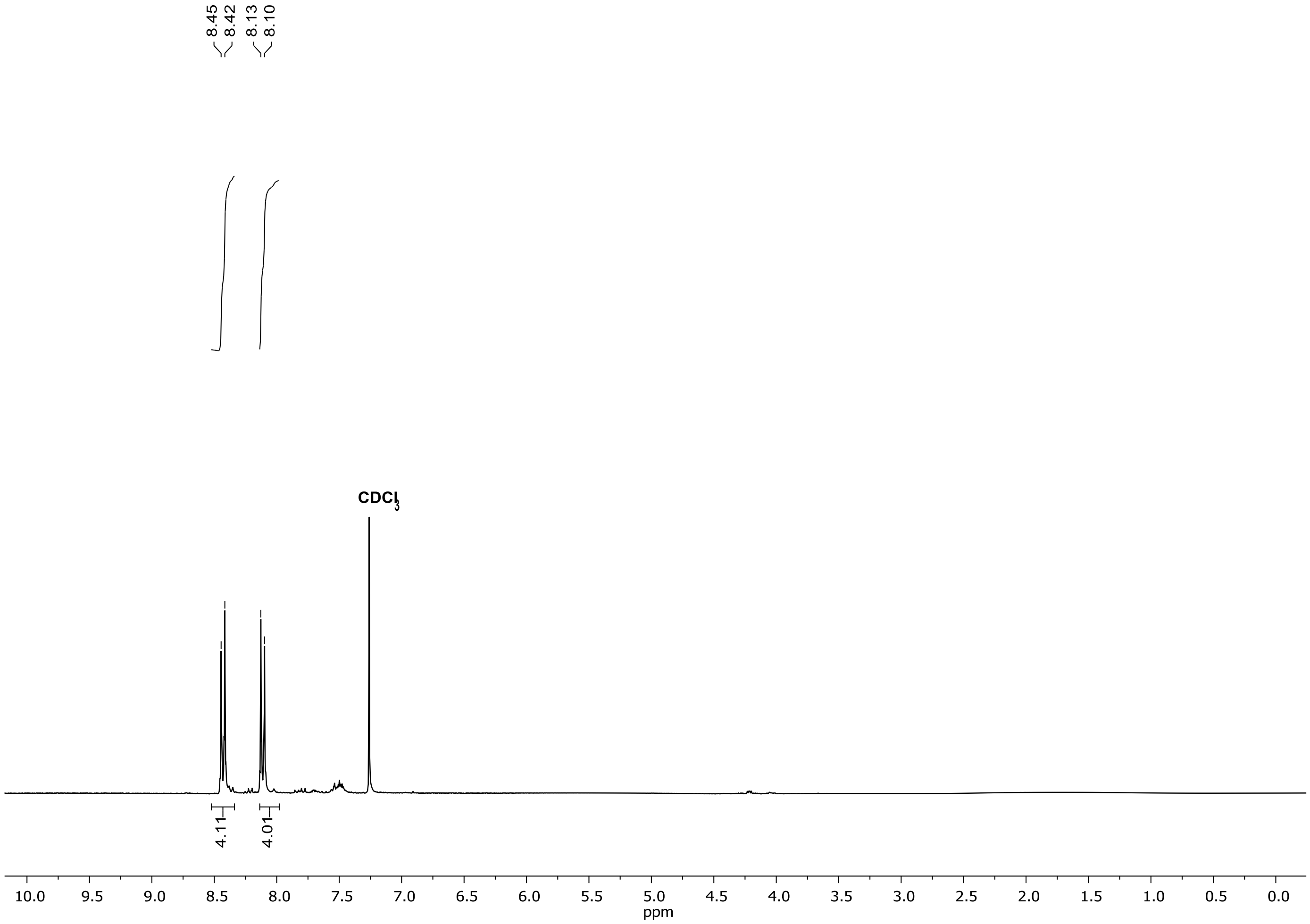

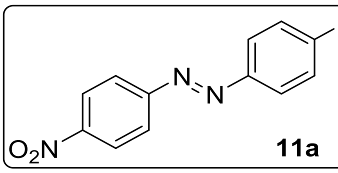

11a

$^{13}C\{^{1}H\}$-NMR (75 MHz, $CDCl_3$)

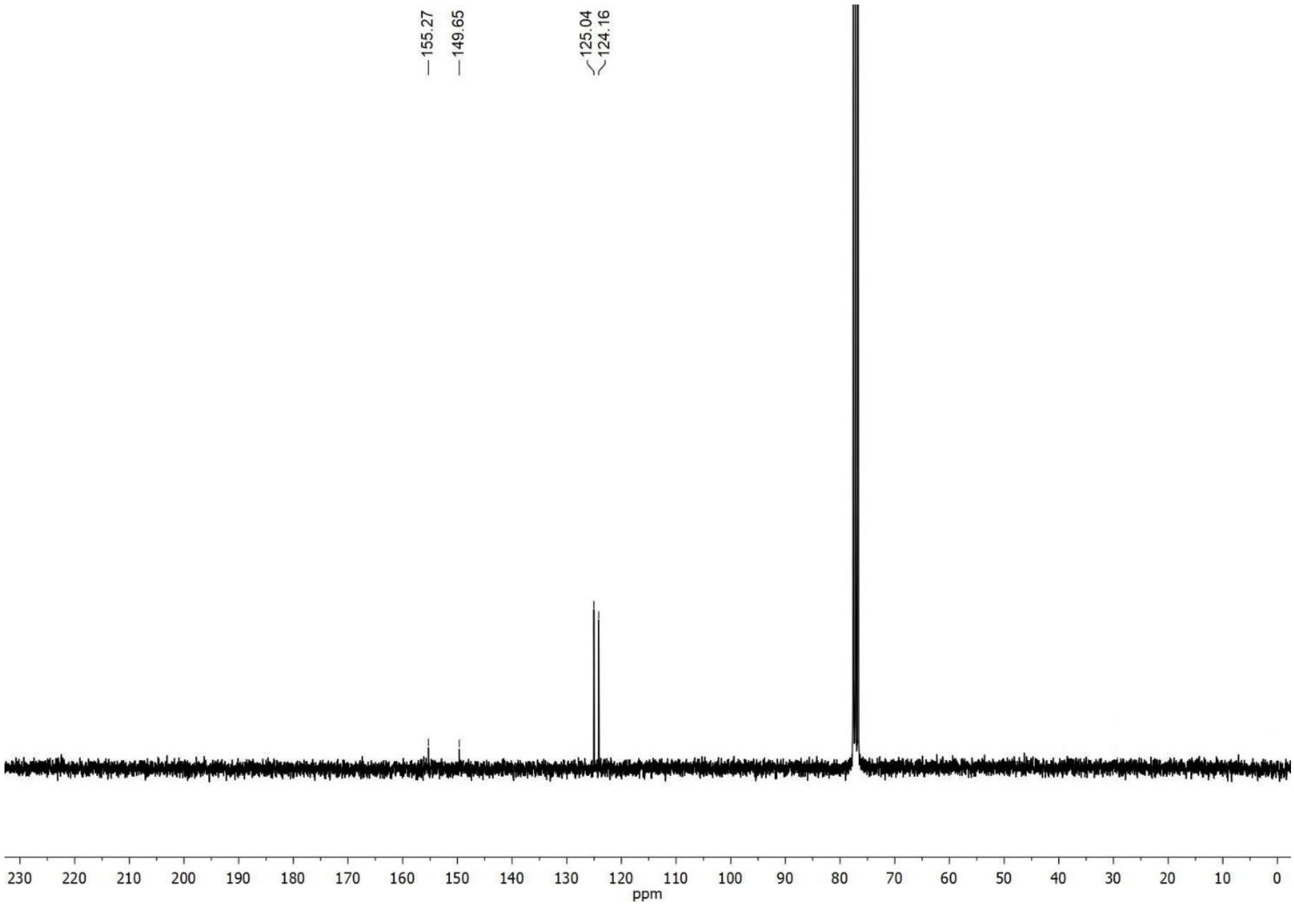

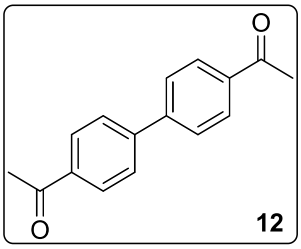

12

^{1}H-NMR (300 MHz, $CDCl_3$)

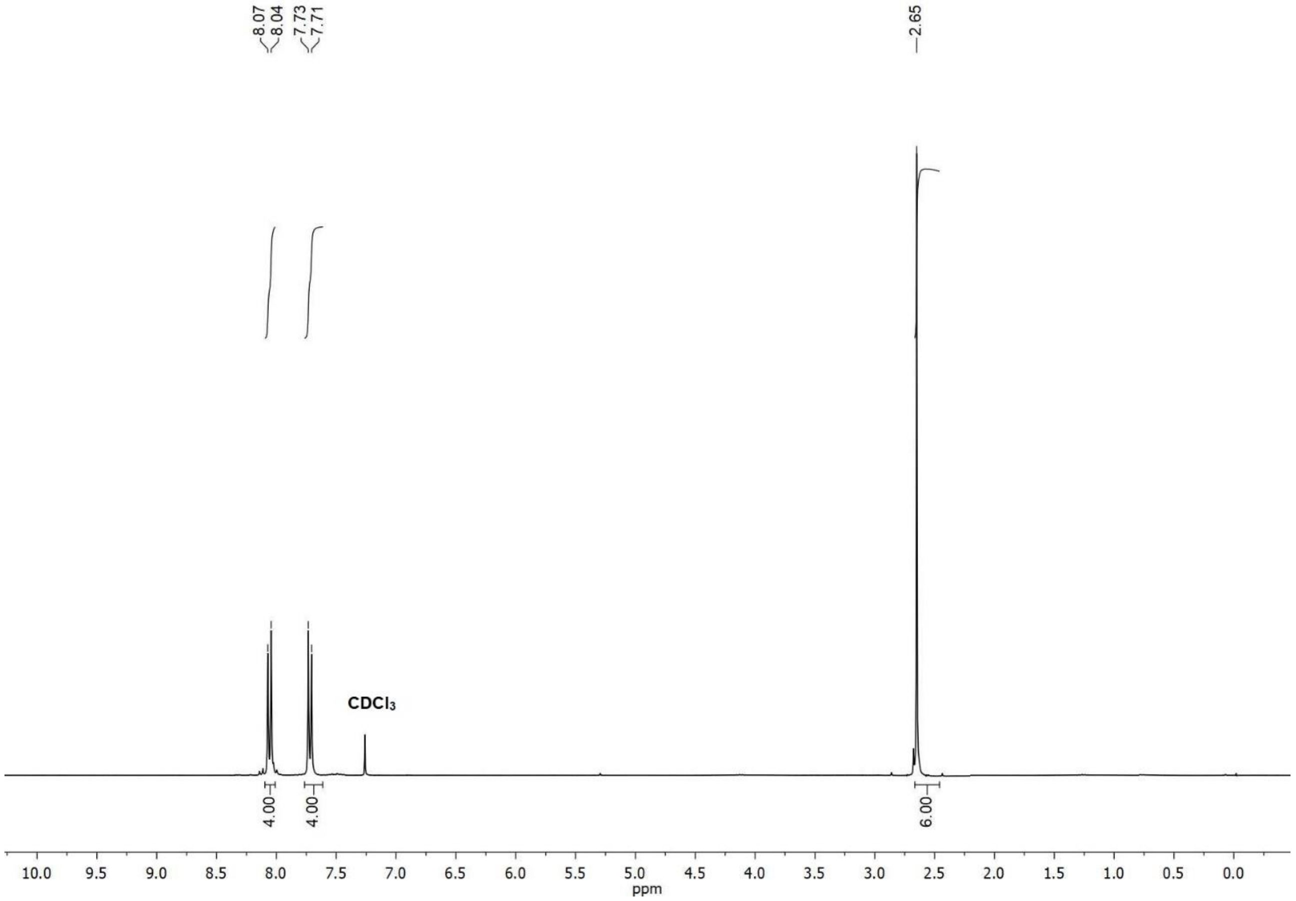

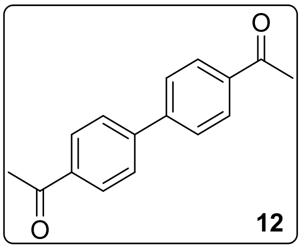

12

$^{13}C\{^{1}H\}$-NMR (75 MHz, $CDCl_3$)

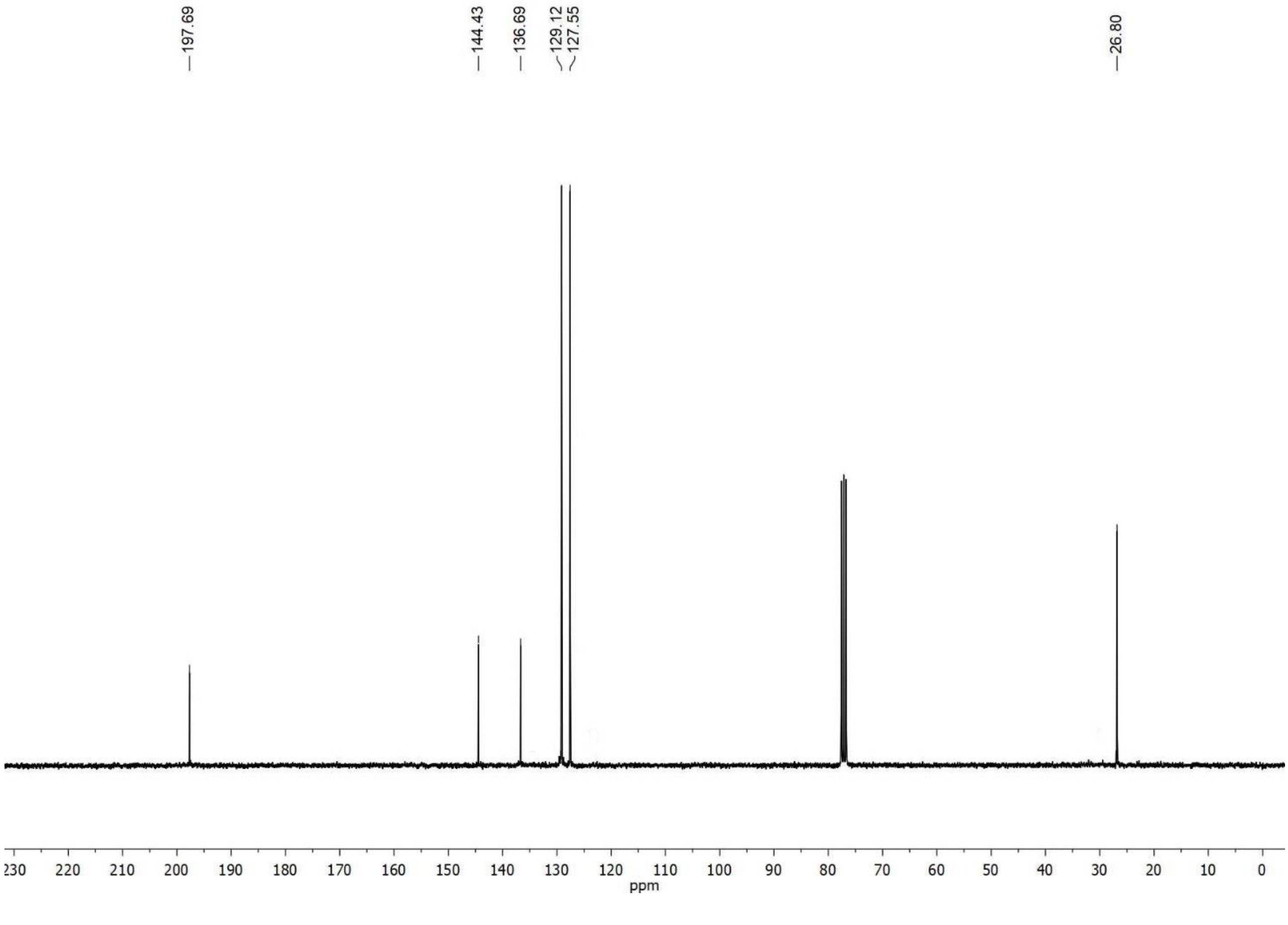

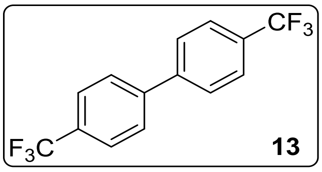

^{1}H-NMR (300 MHz, $CDCl_3$)

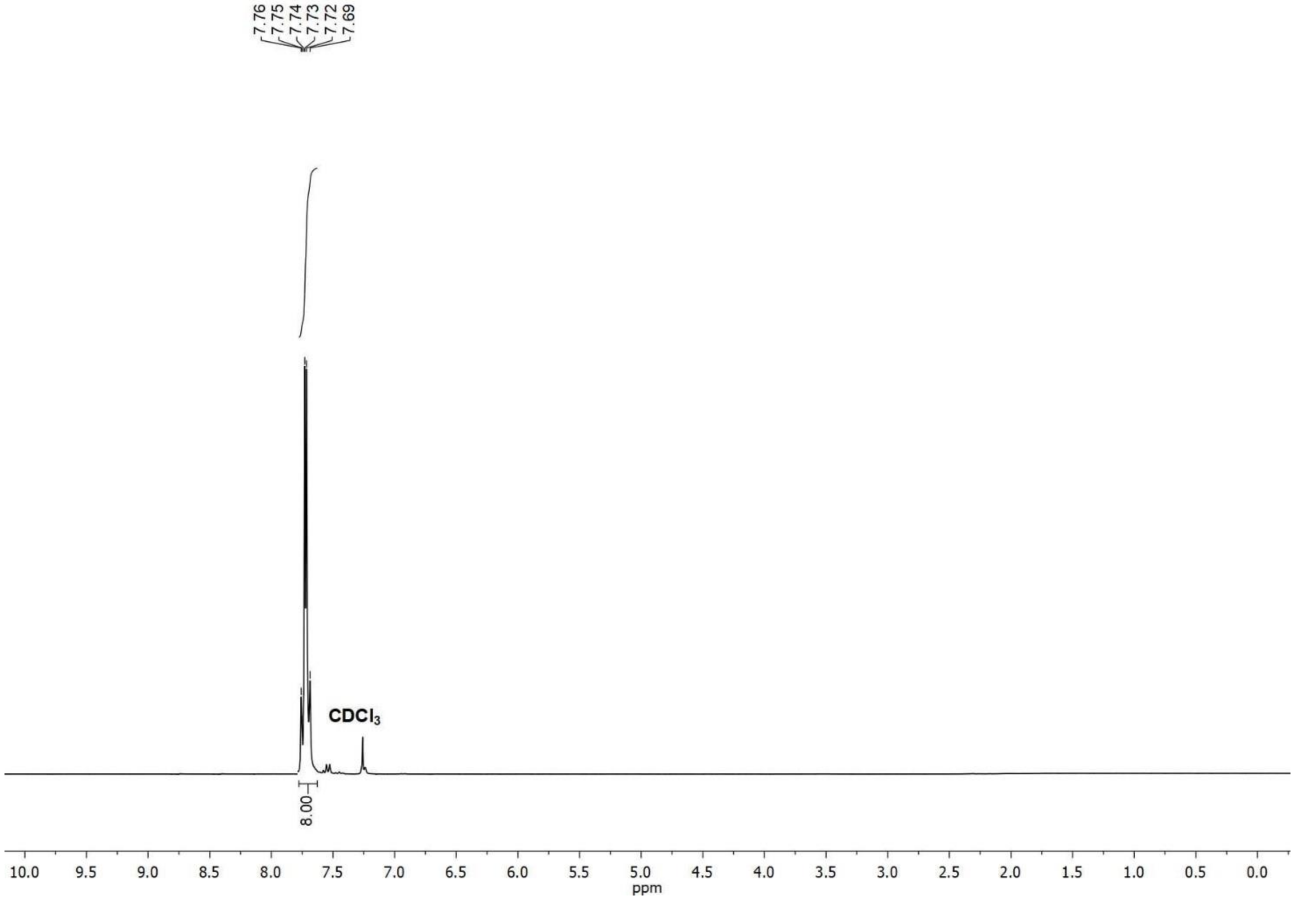

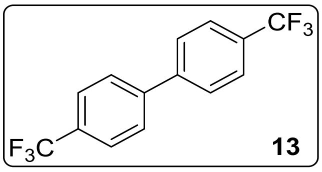

13

$^{13}C\{^{1}H\}$-NMR (75 MHz, $CDCl_3$)

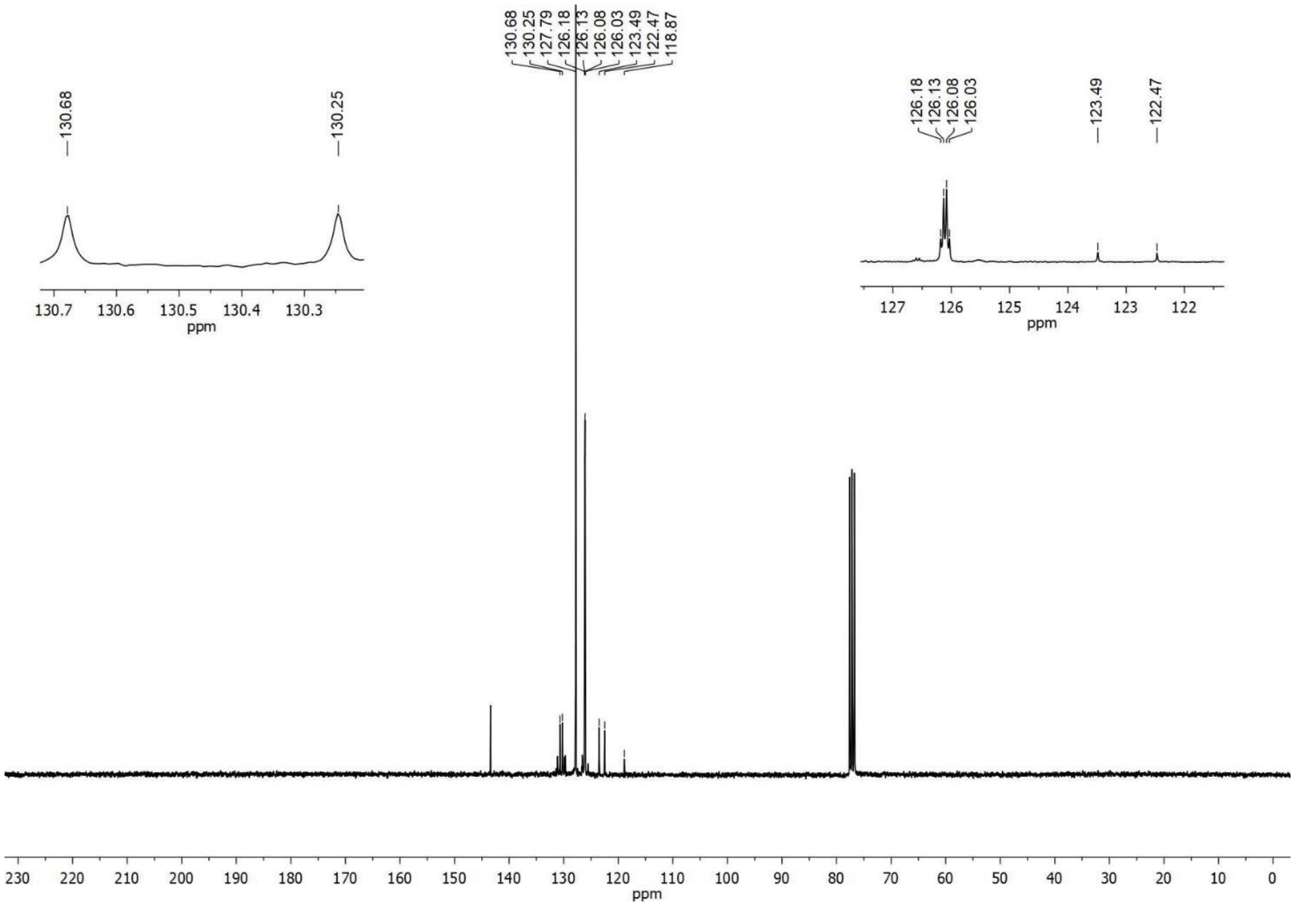

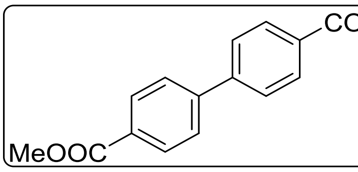

14

^{1}H-NMR (300 MHz, $CDCl_3$)

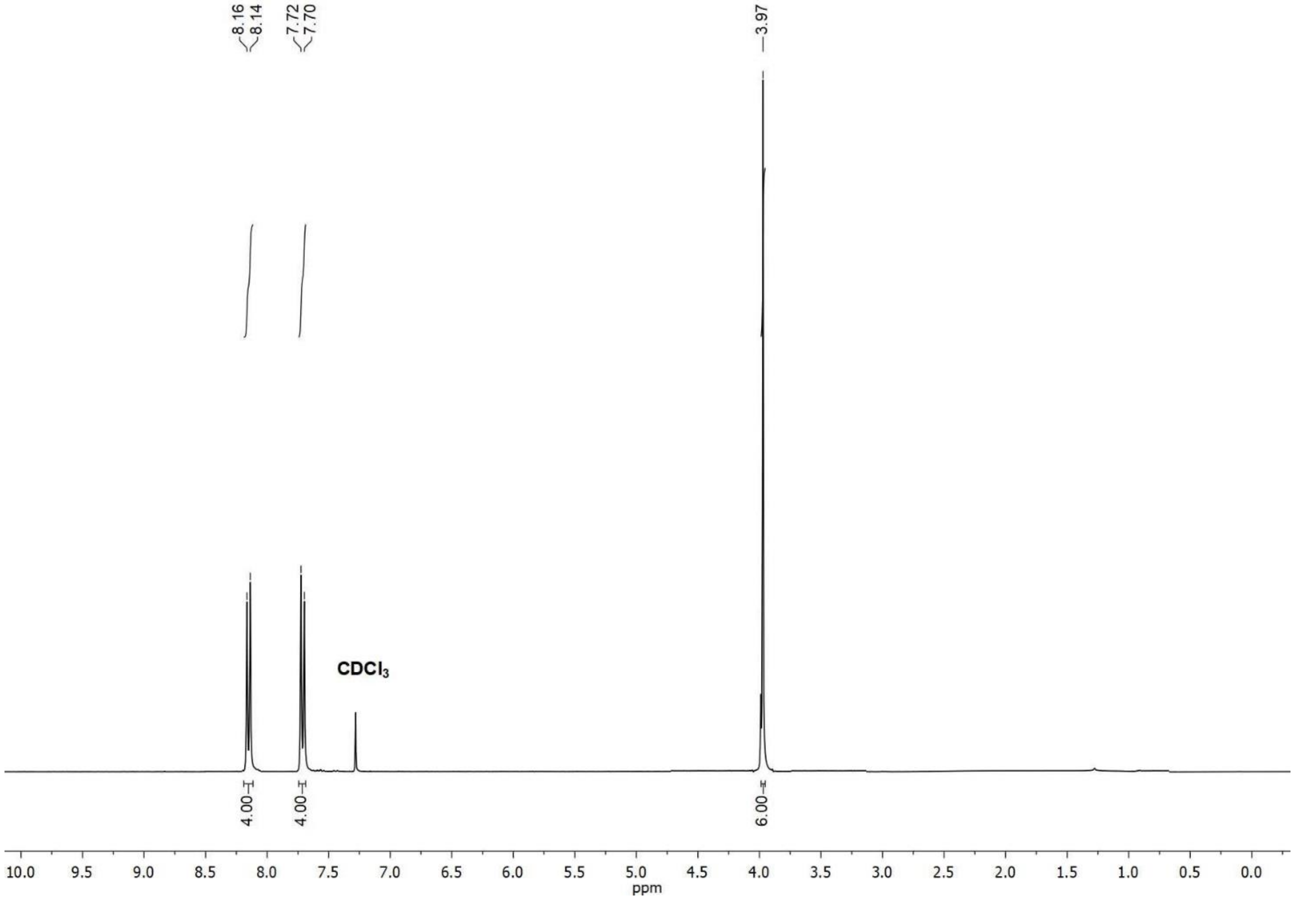

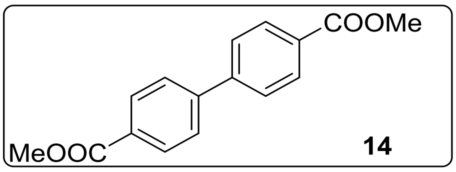

14

$^{13}C\{^{1}H\}$-NMR (75 MHz, $CDCl_3$)

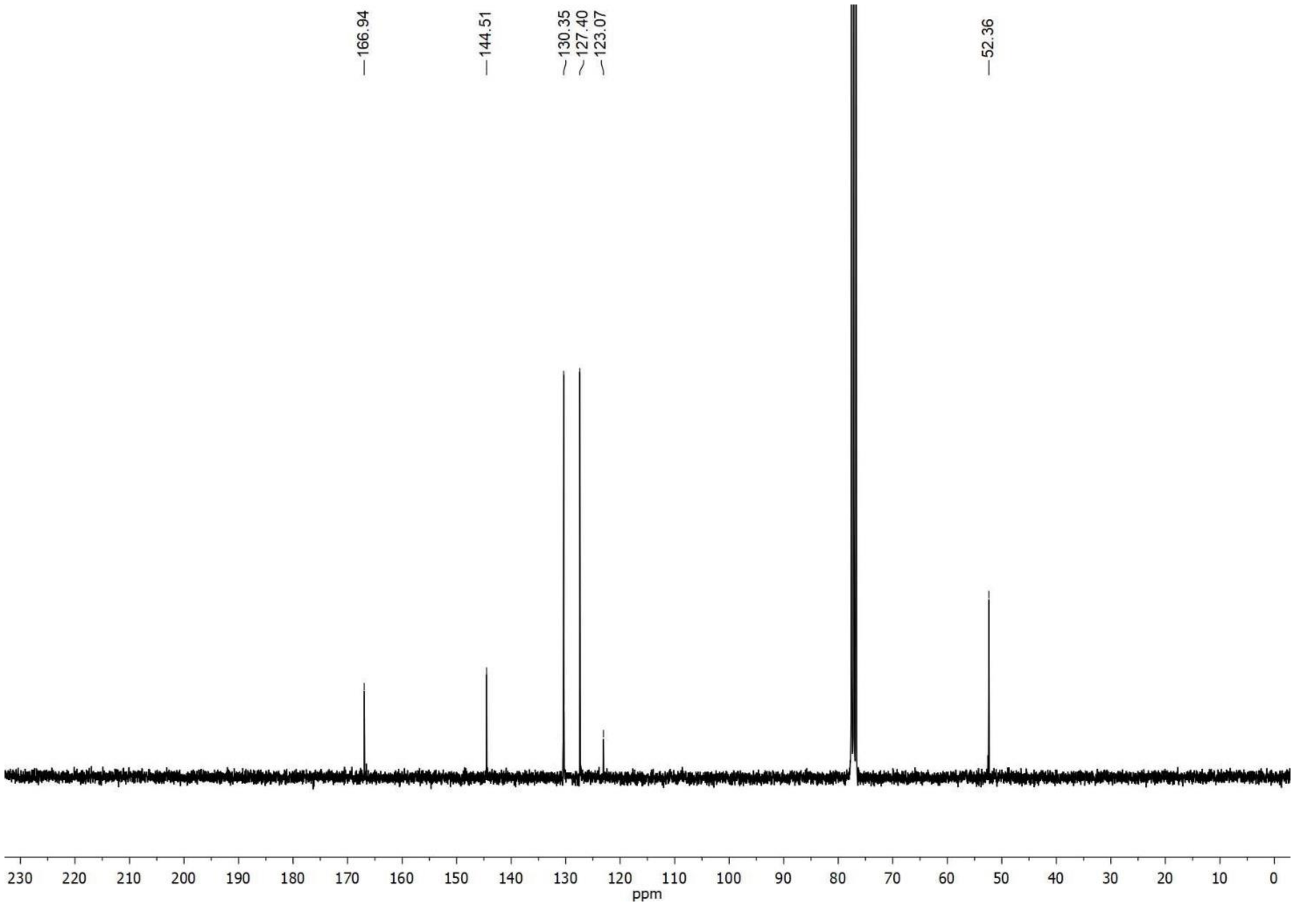

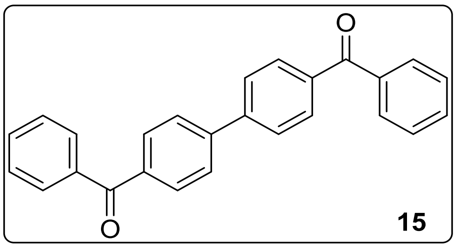

^{1}H-NMR (300 MHz, $CDCl_3$)

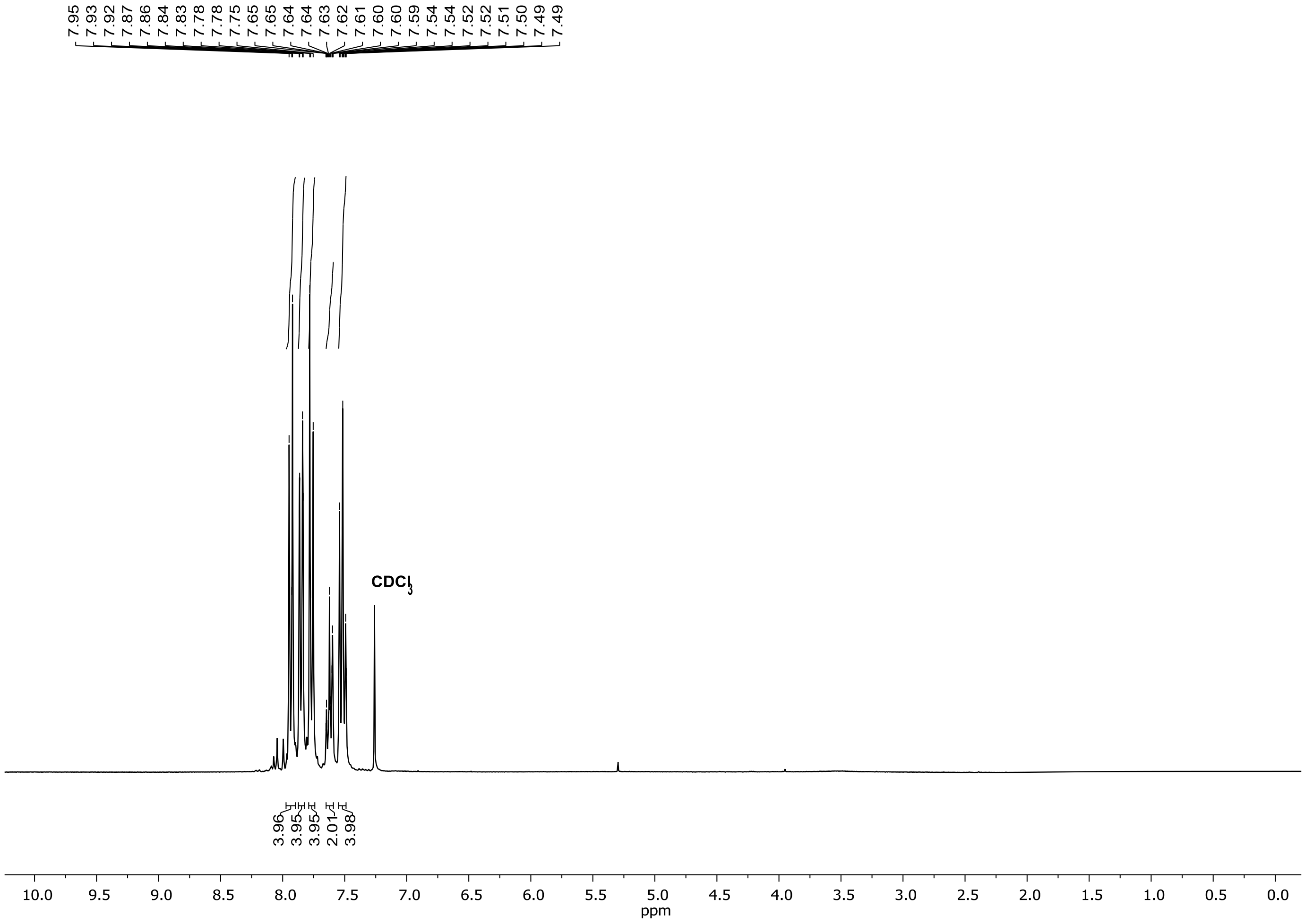

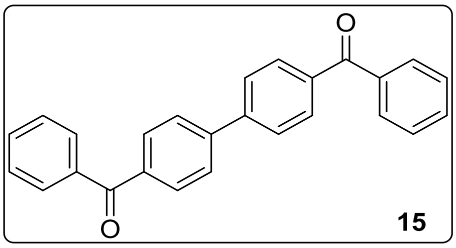

15

$^{13}C\{^{1}H\}$-NMR (75 MHz, $CDCl_3$)

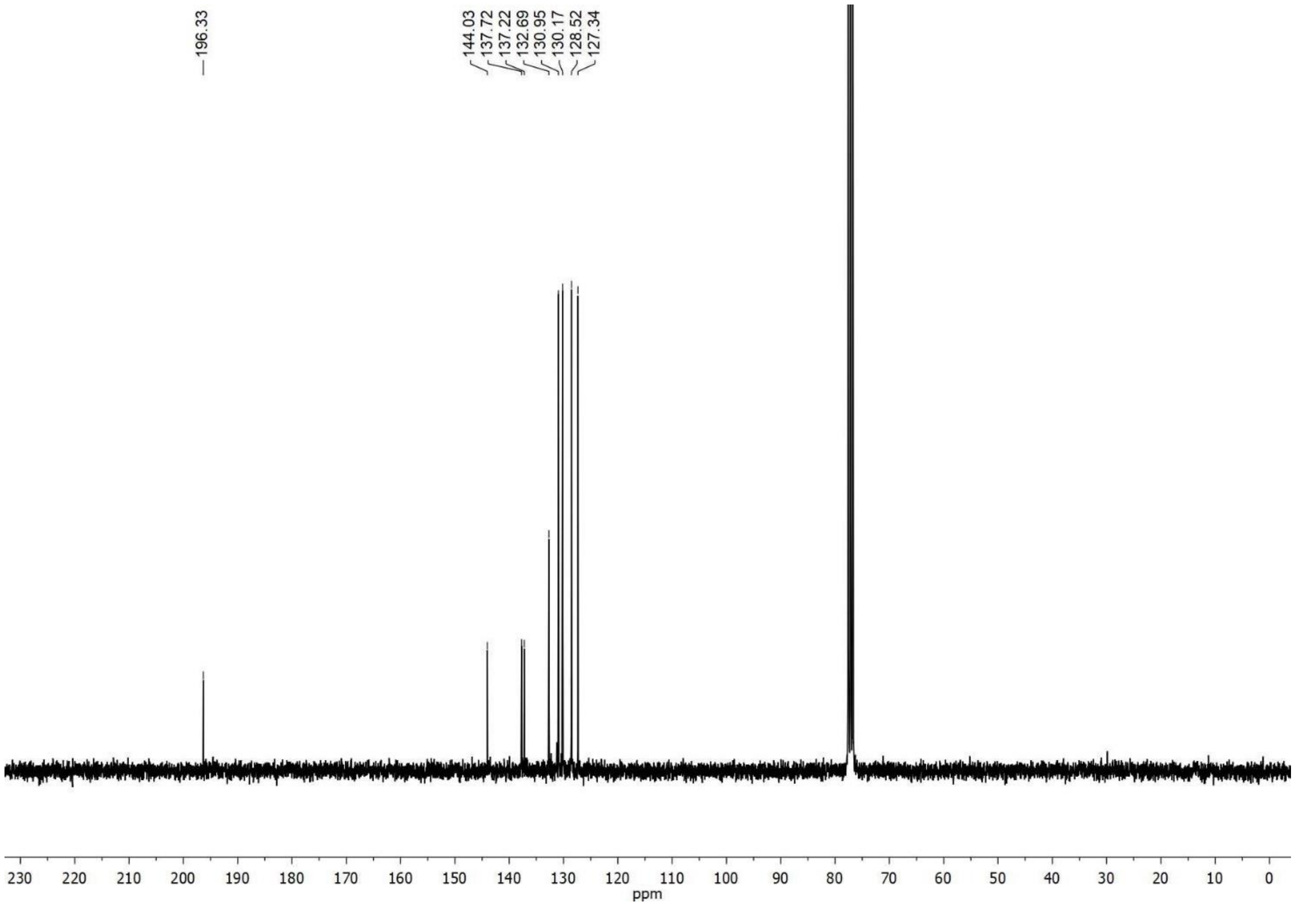

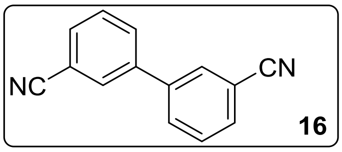

^{1}H-NMR (300 MHz, $CDCl_3$)

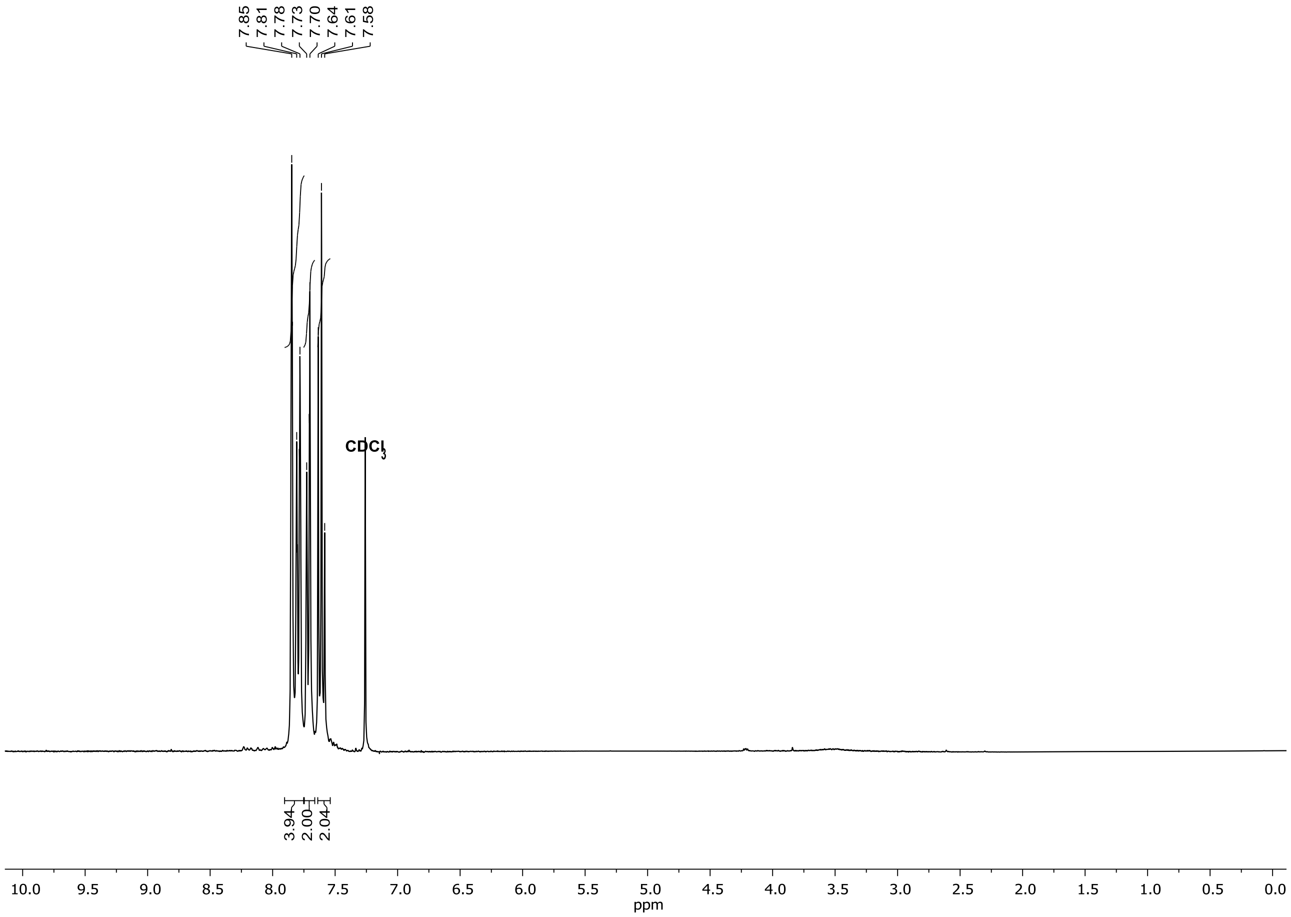

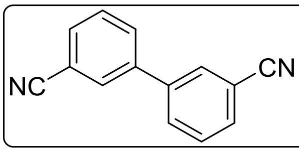

16

$^{13}C\{^{1}H\}$-NMR (75 MHz, $CDCl_3$)

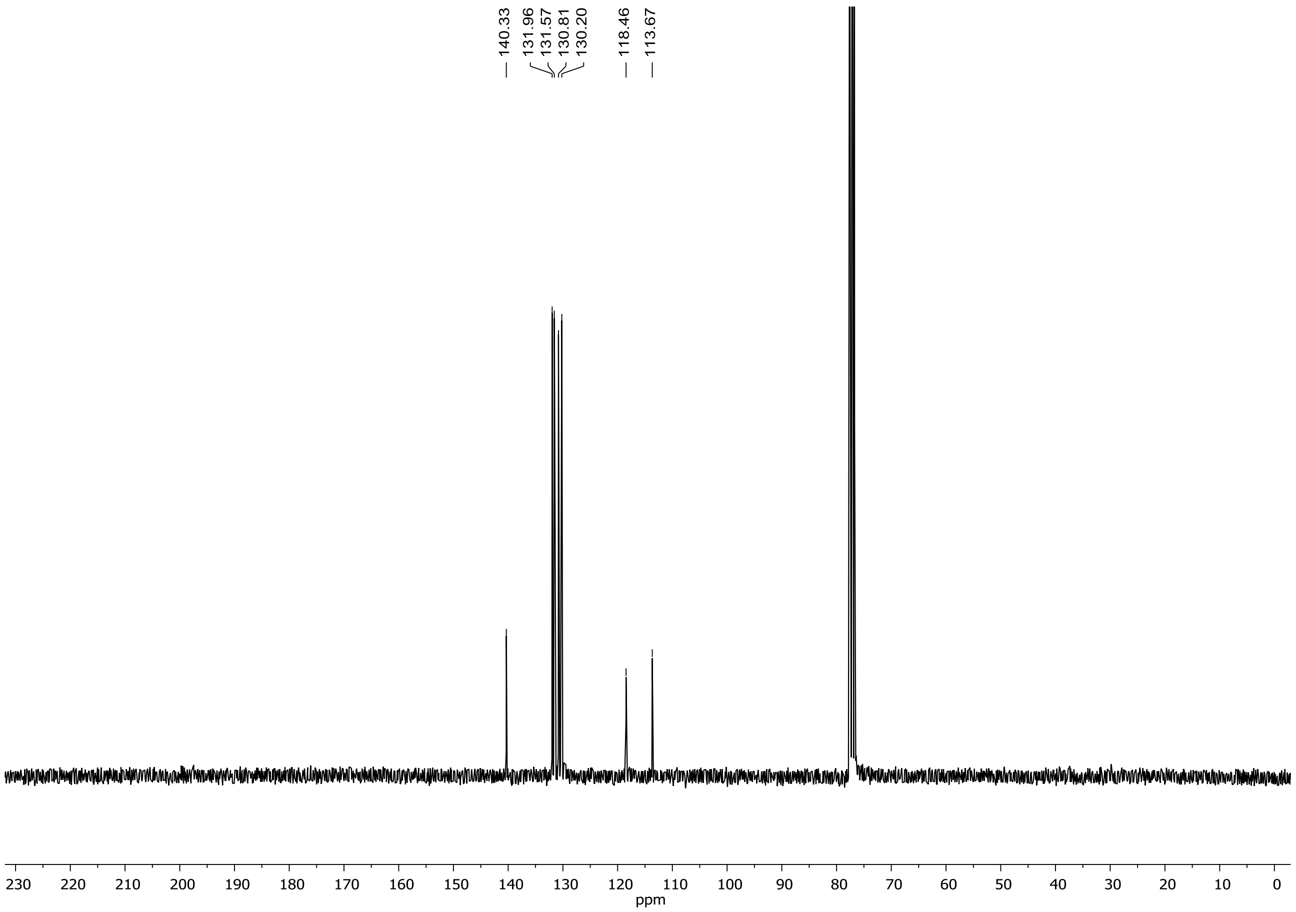

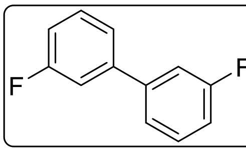

17 ^{1}H-NMR (300 MHz, $CDCl_3$)

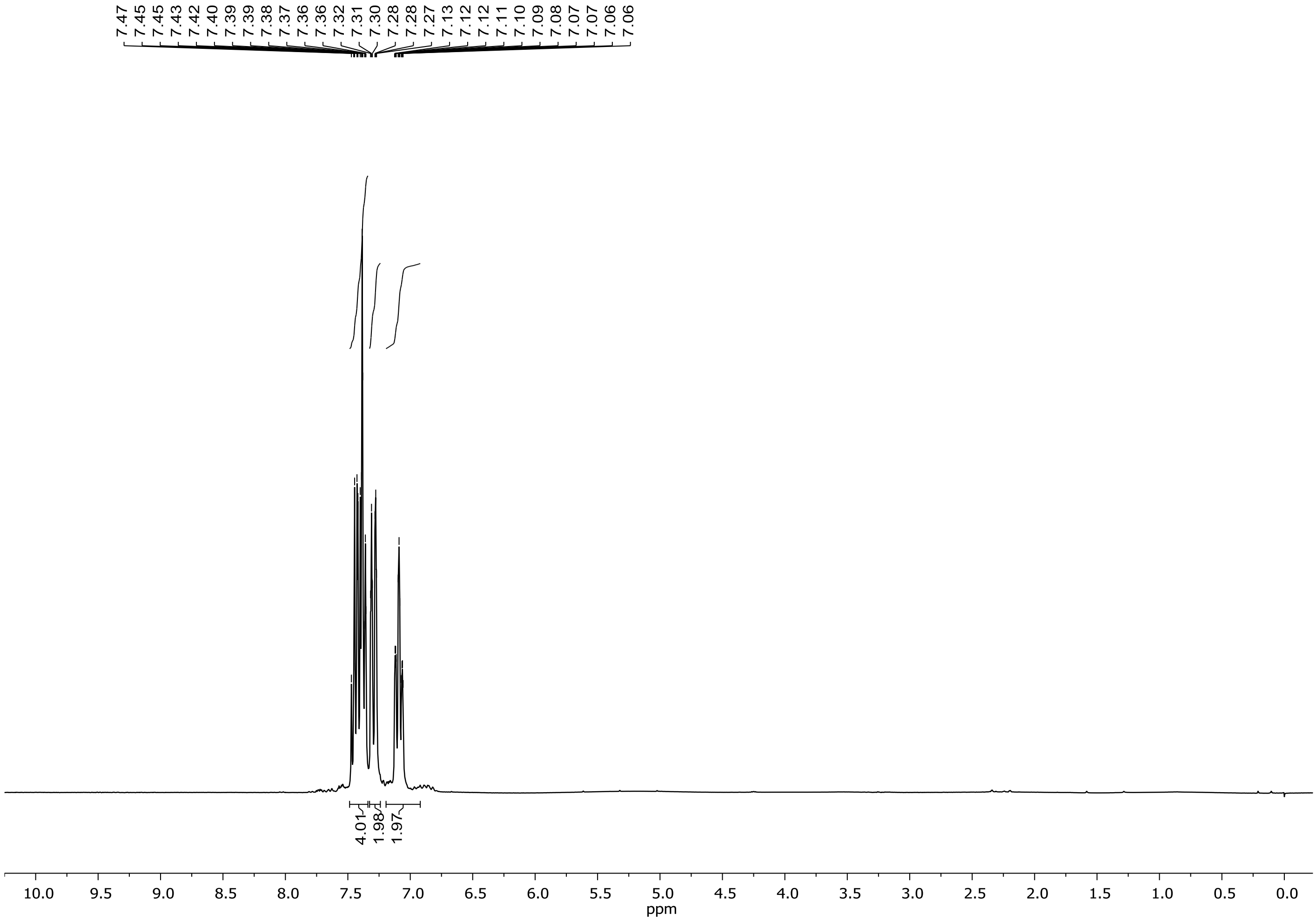

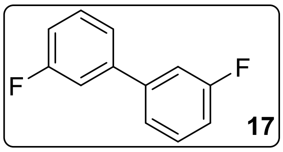

$^{13}C\{^{1}H\}$-NMR (75 MHz, $CDCl_3$)

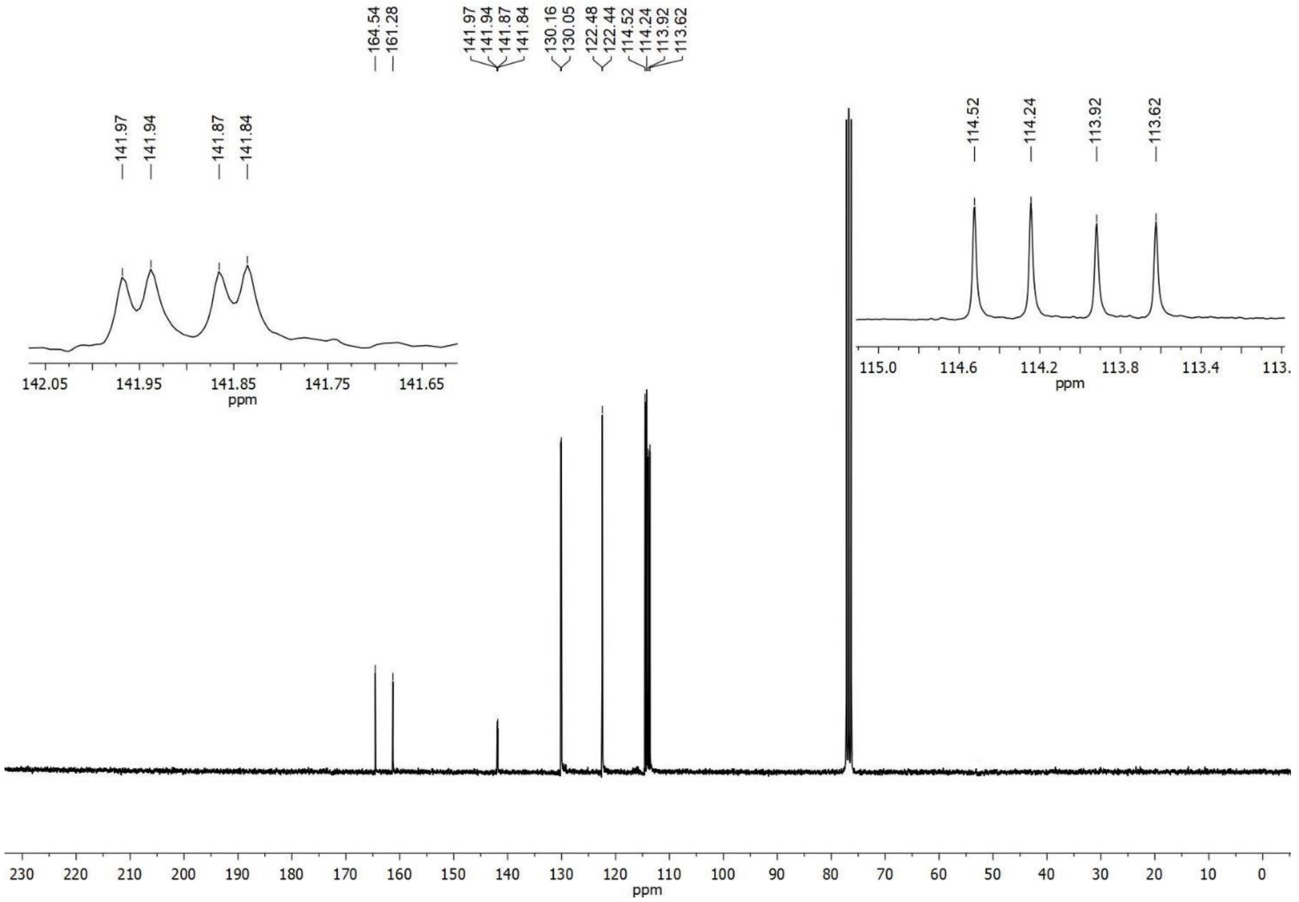

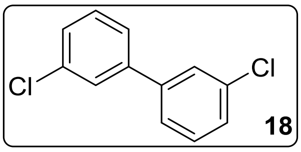

18 ^{1}H-NMR (300 MHz, $CDCl_3$)

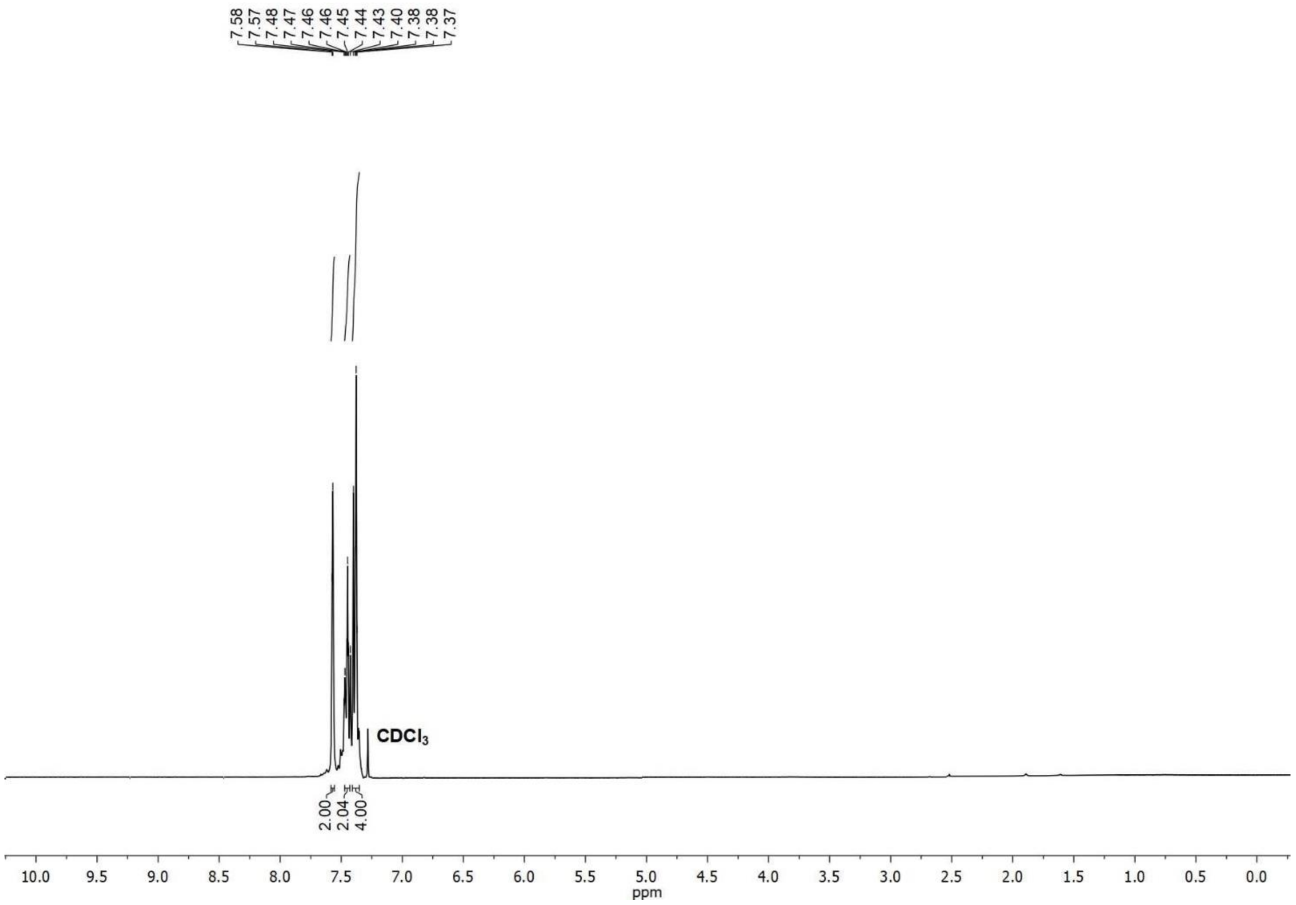

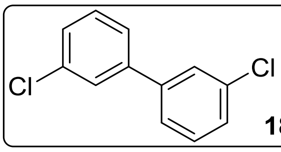

18 $^{13}C\{^{1}H\}$-NMR (75 MHz, $CDCl_3$)

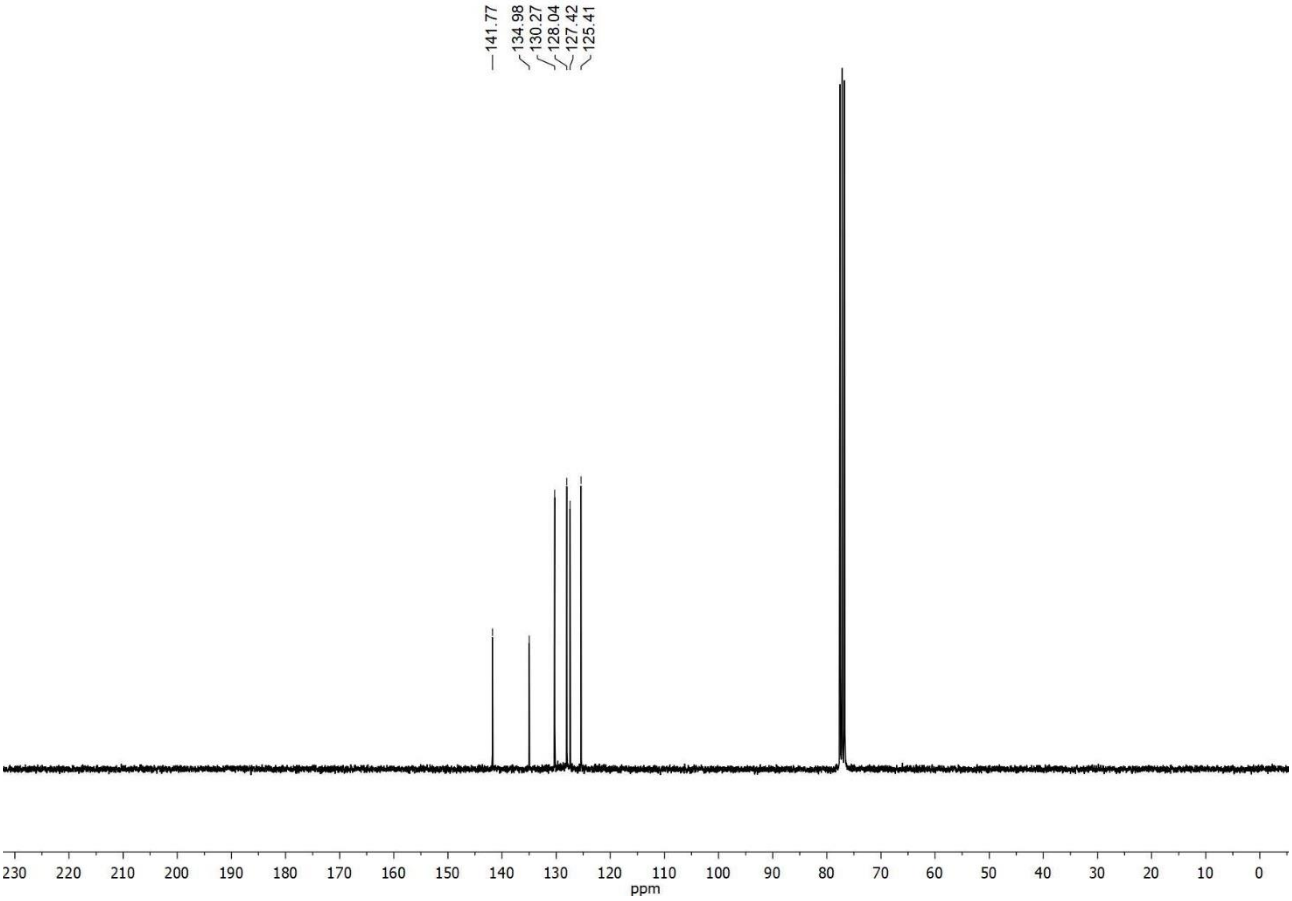

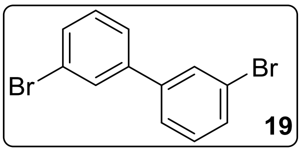

19 ^{1}H-NMR (300 MHz, $CDCl_3$)

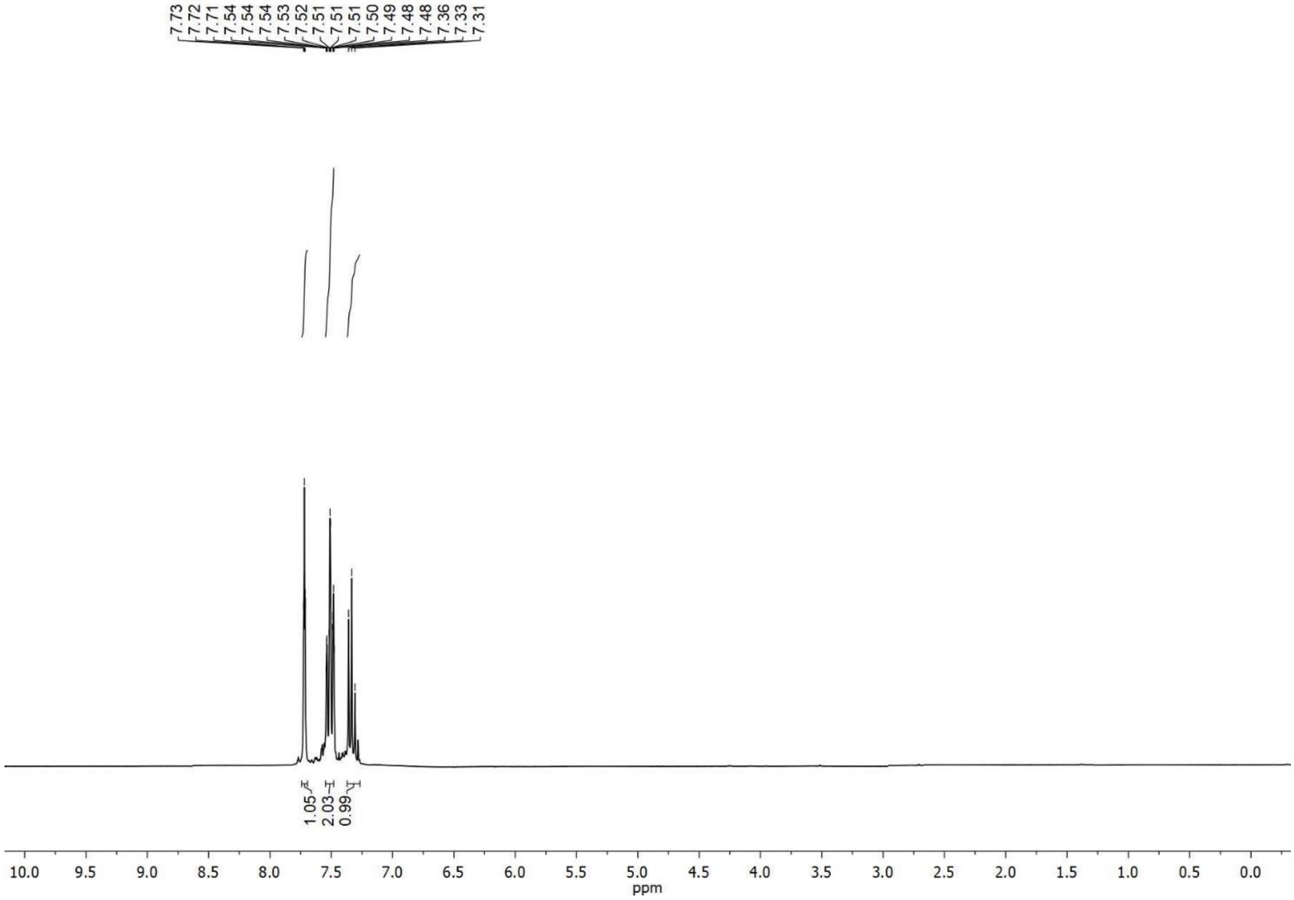

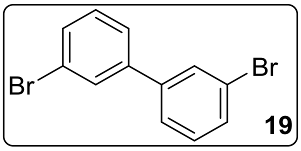

19 $^{13}C\{^1H\}$-NMR (75 MHz, $CDCl_3$)

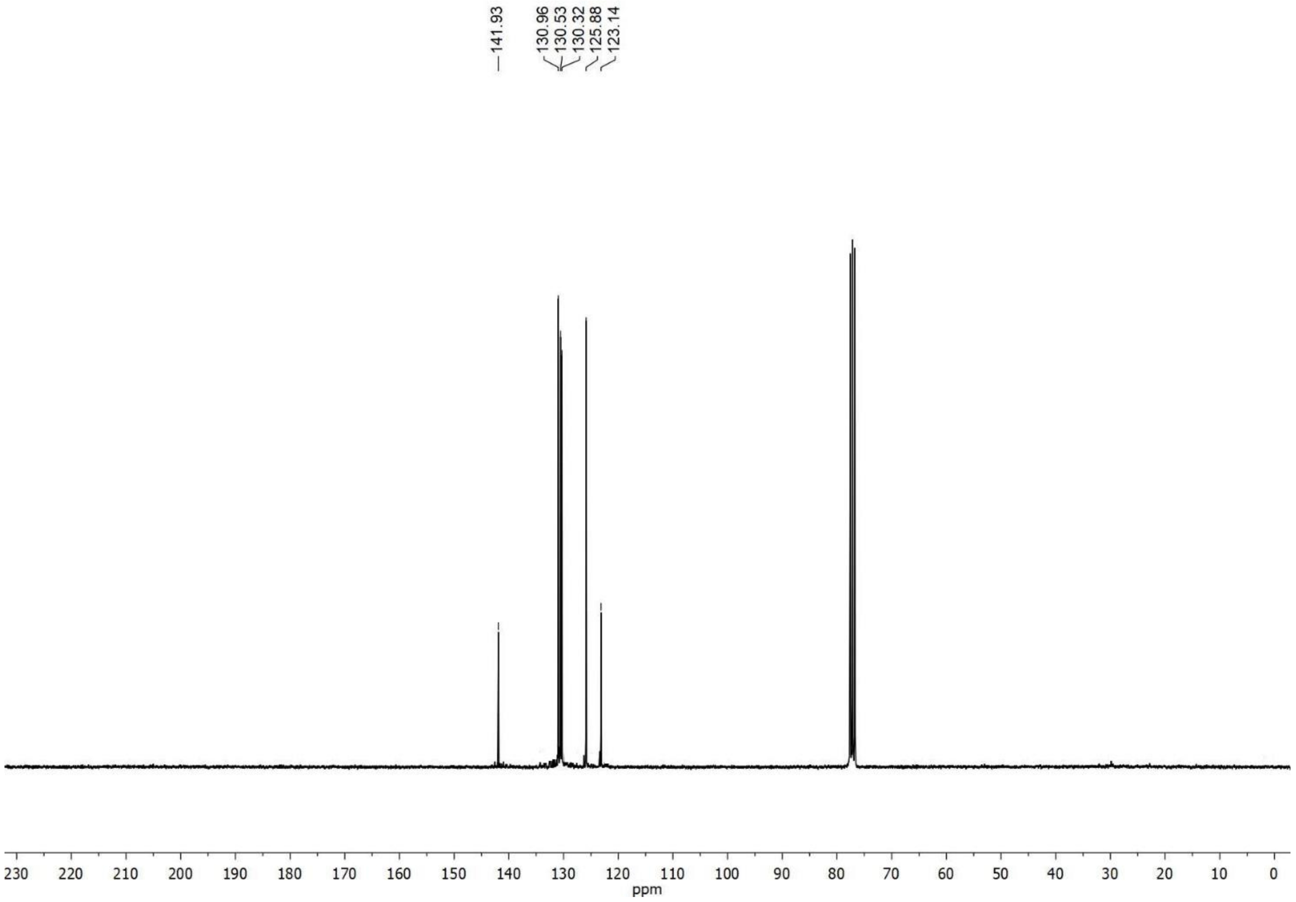

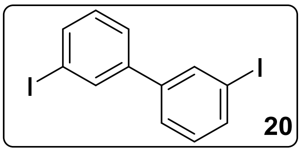

20

^{1}H-NMR (300 MHz, $CDCl_3$)

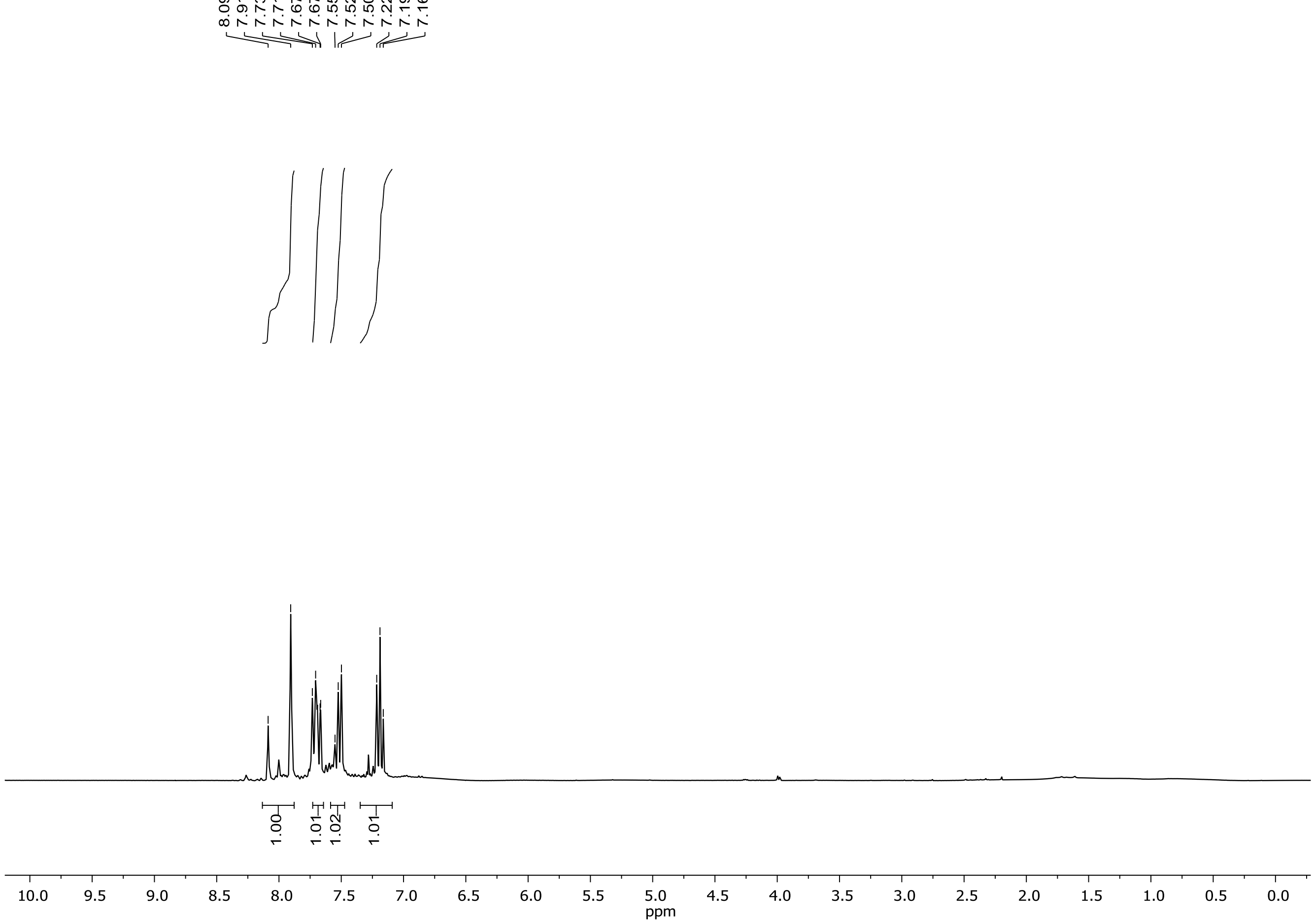

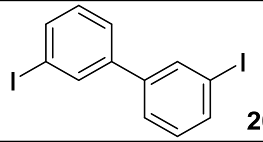

20 $^{13}C\{^{1}H\}$-NMR (75 MHz, $CDCl_3$)

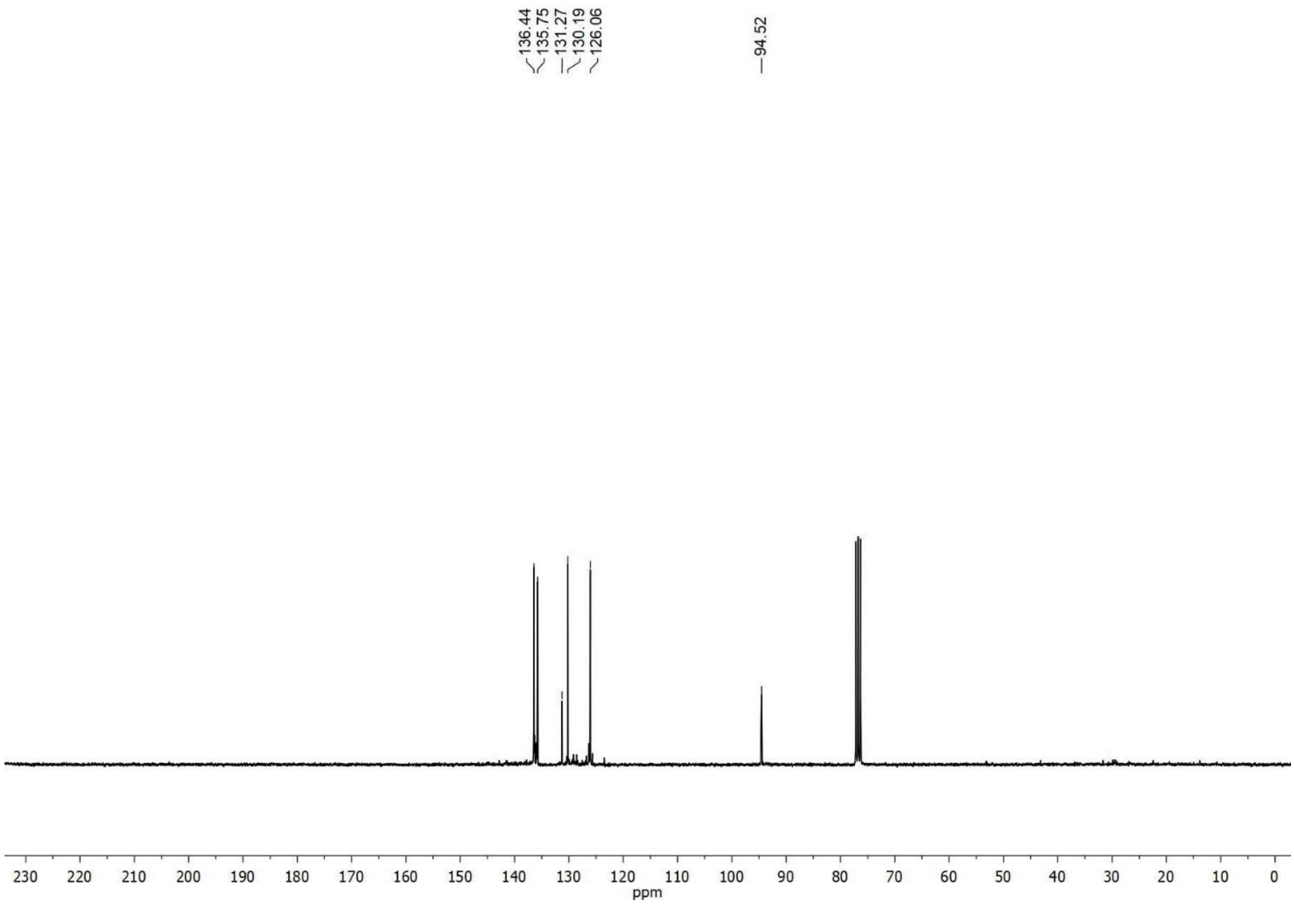

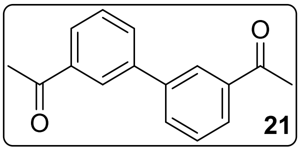

21 ^{1}H-NMR (300 MHz, $CDCl_3$)

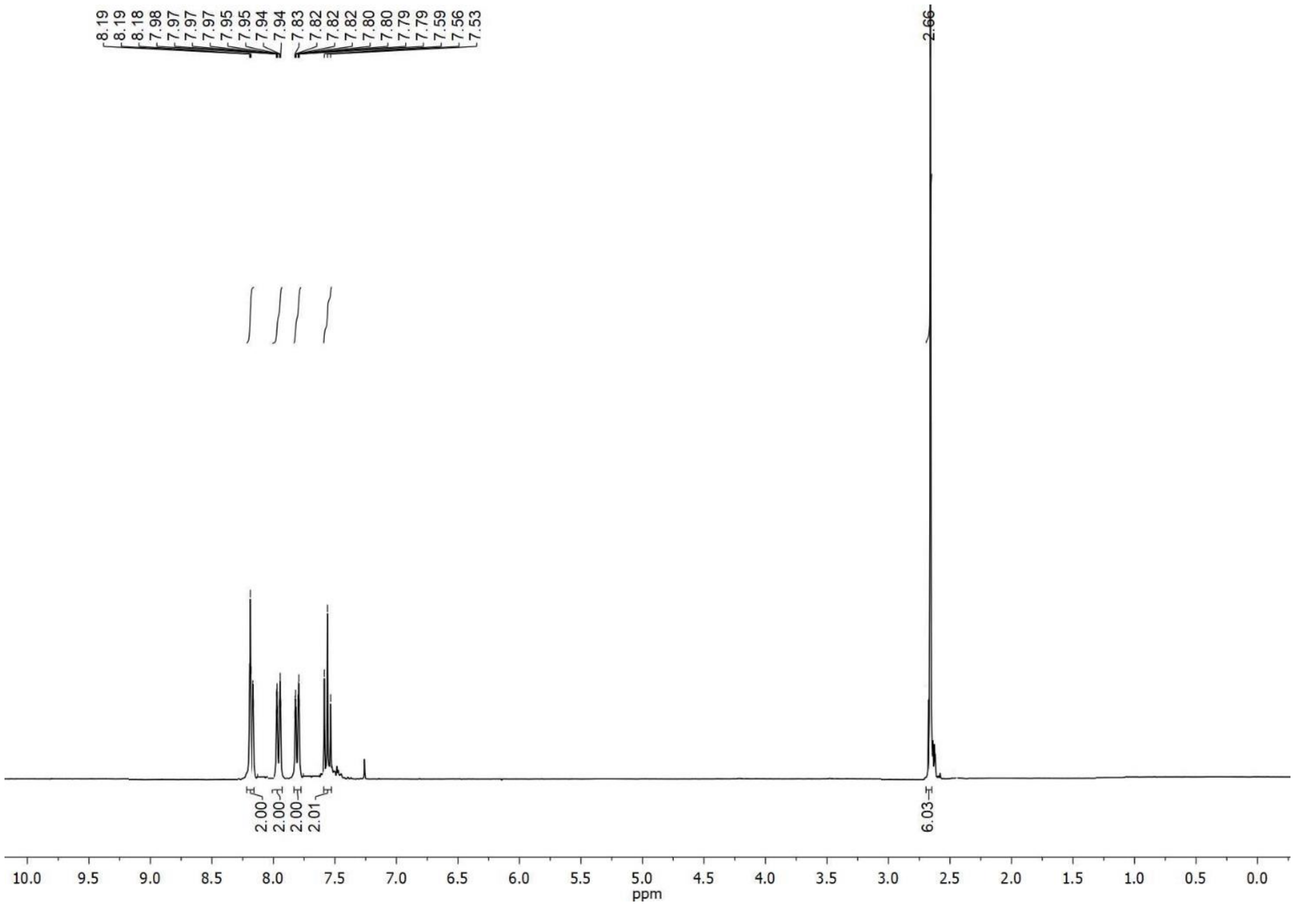

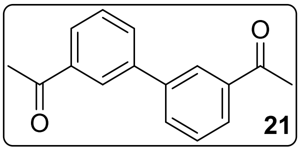

21

$^{13}C\{^{1}H\}$-NMR (75 MHz, $CDCl_3$)

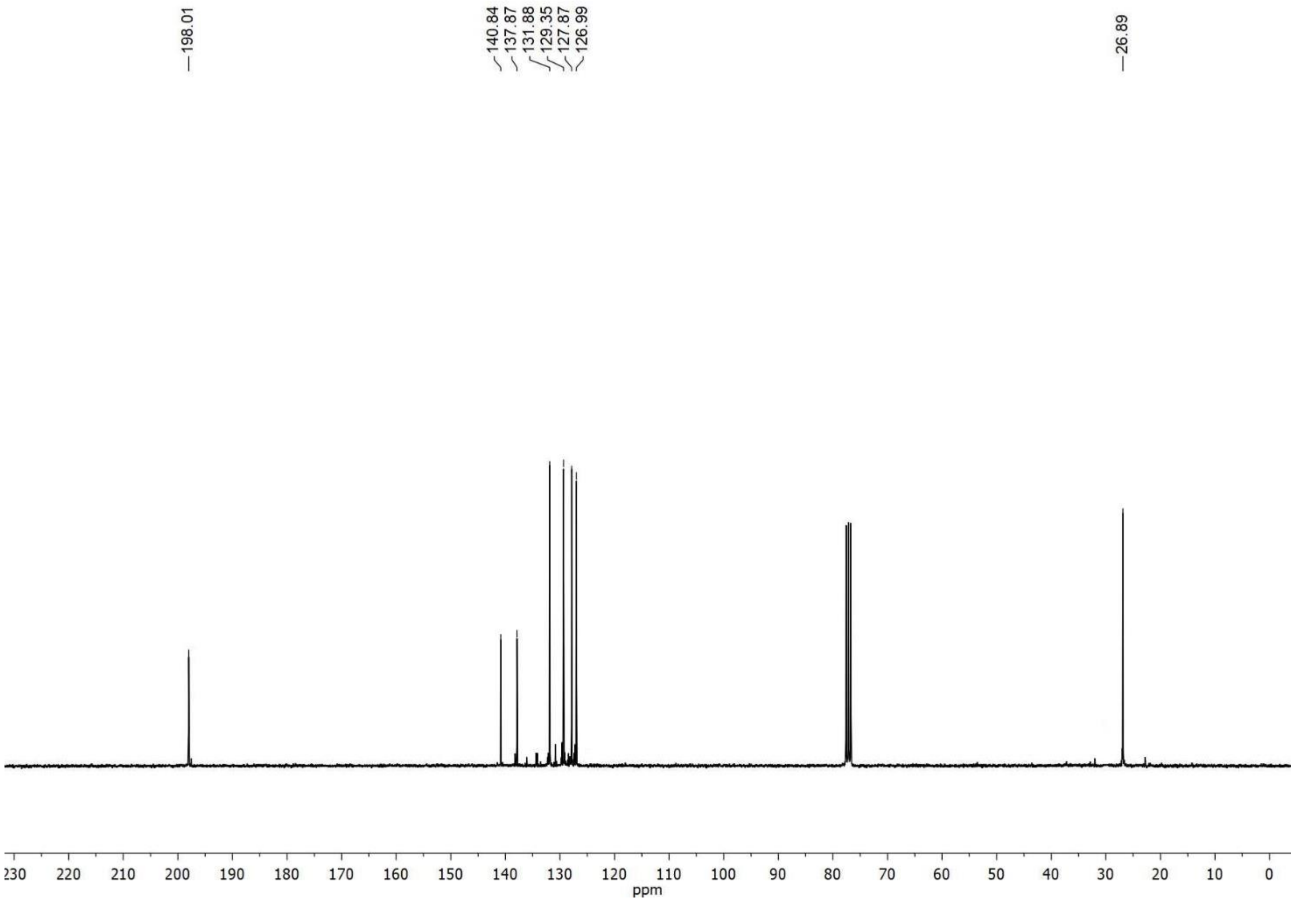

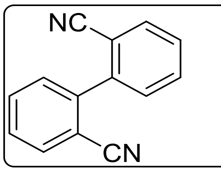

22

^{1}H-NMR (300 MHz, $CDCl_3$)

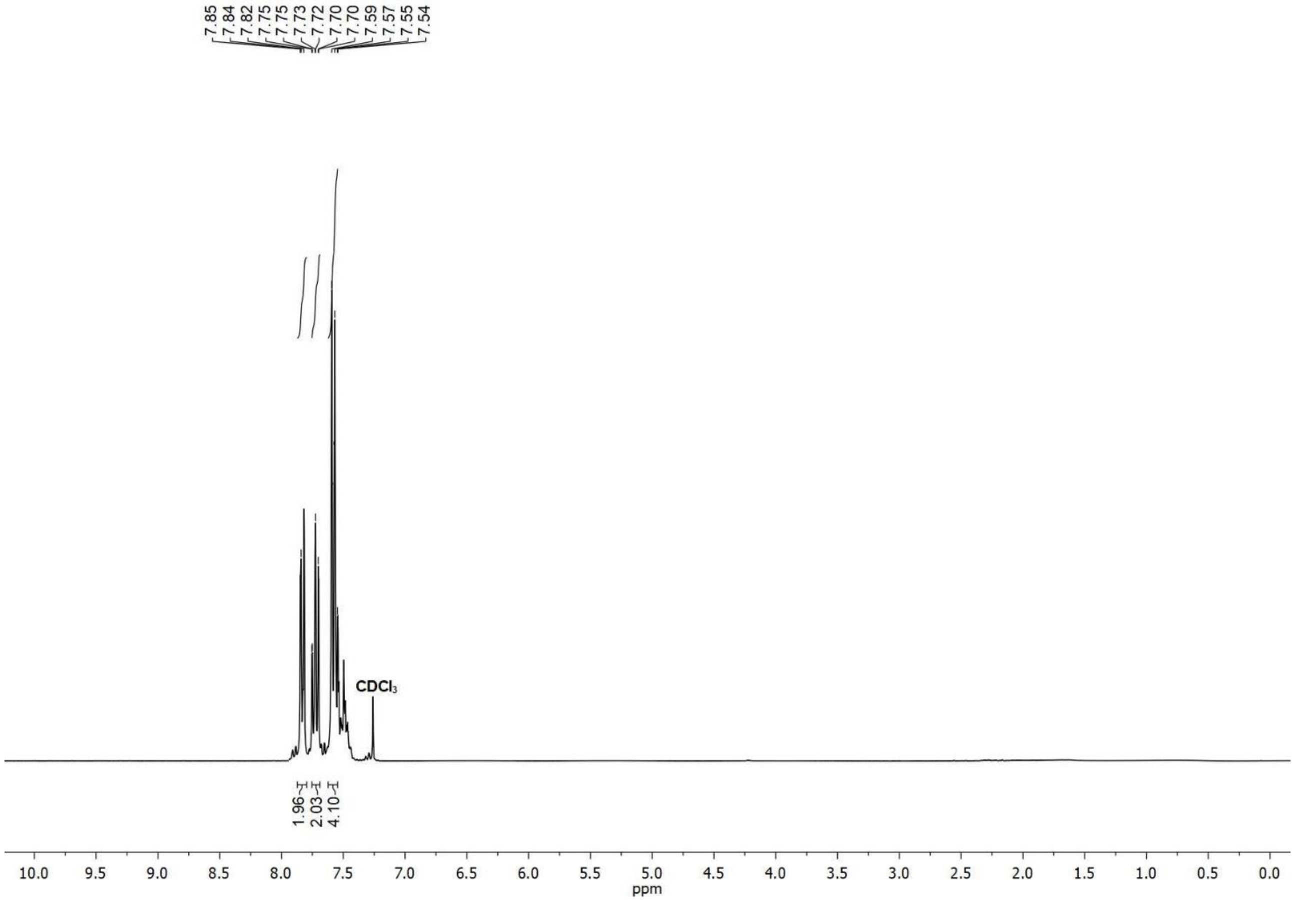

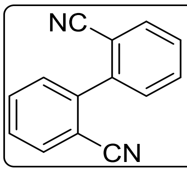

22 $^{13}C\{^1H\}$-NMR (75 MHz, $CDCl_3$)

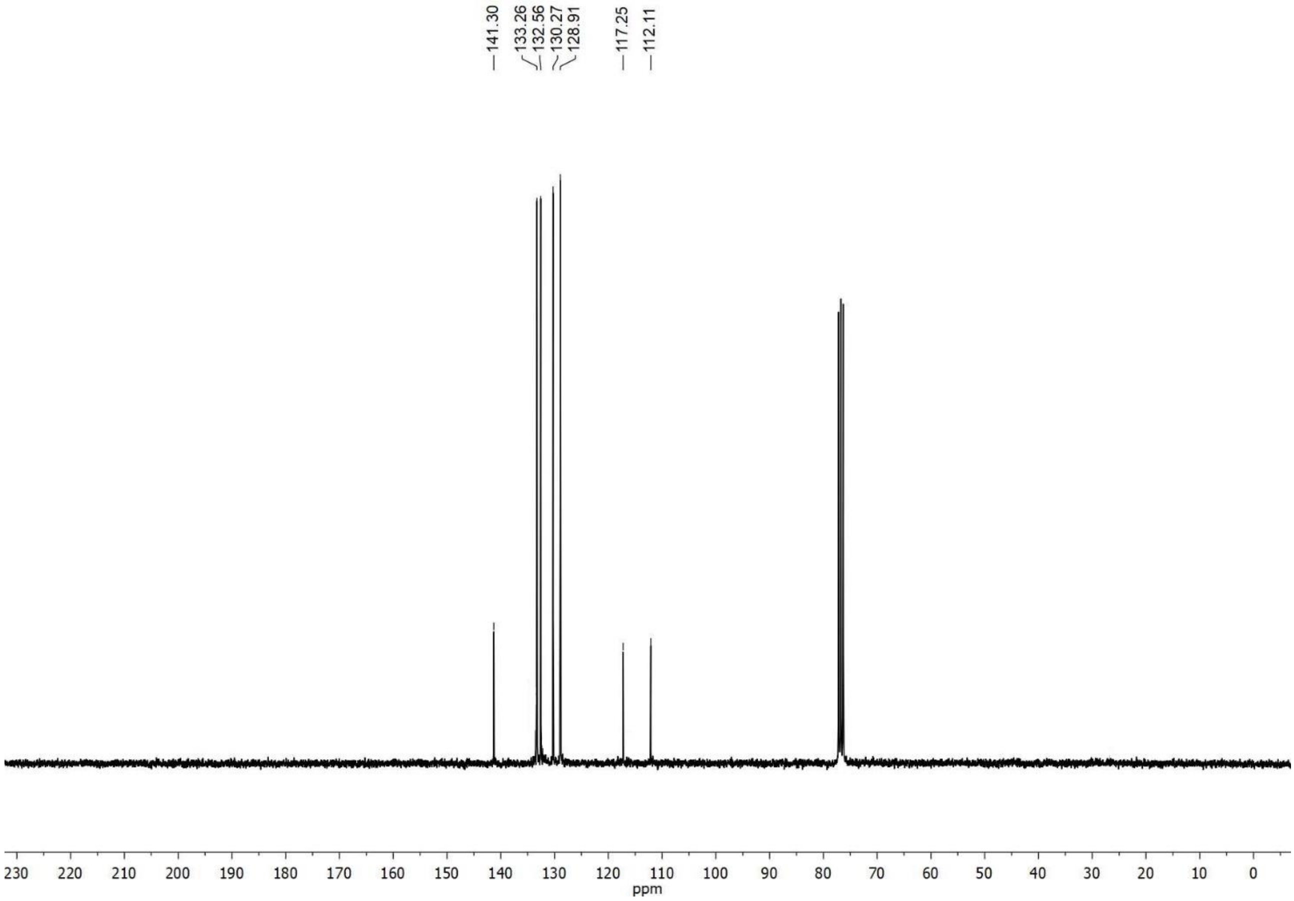

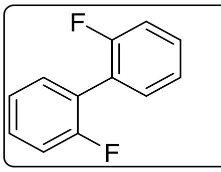

23

^{1}H-NMR (300 MHz, $CDCl_3$)

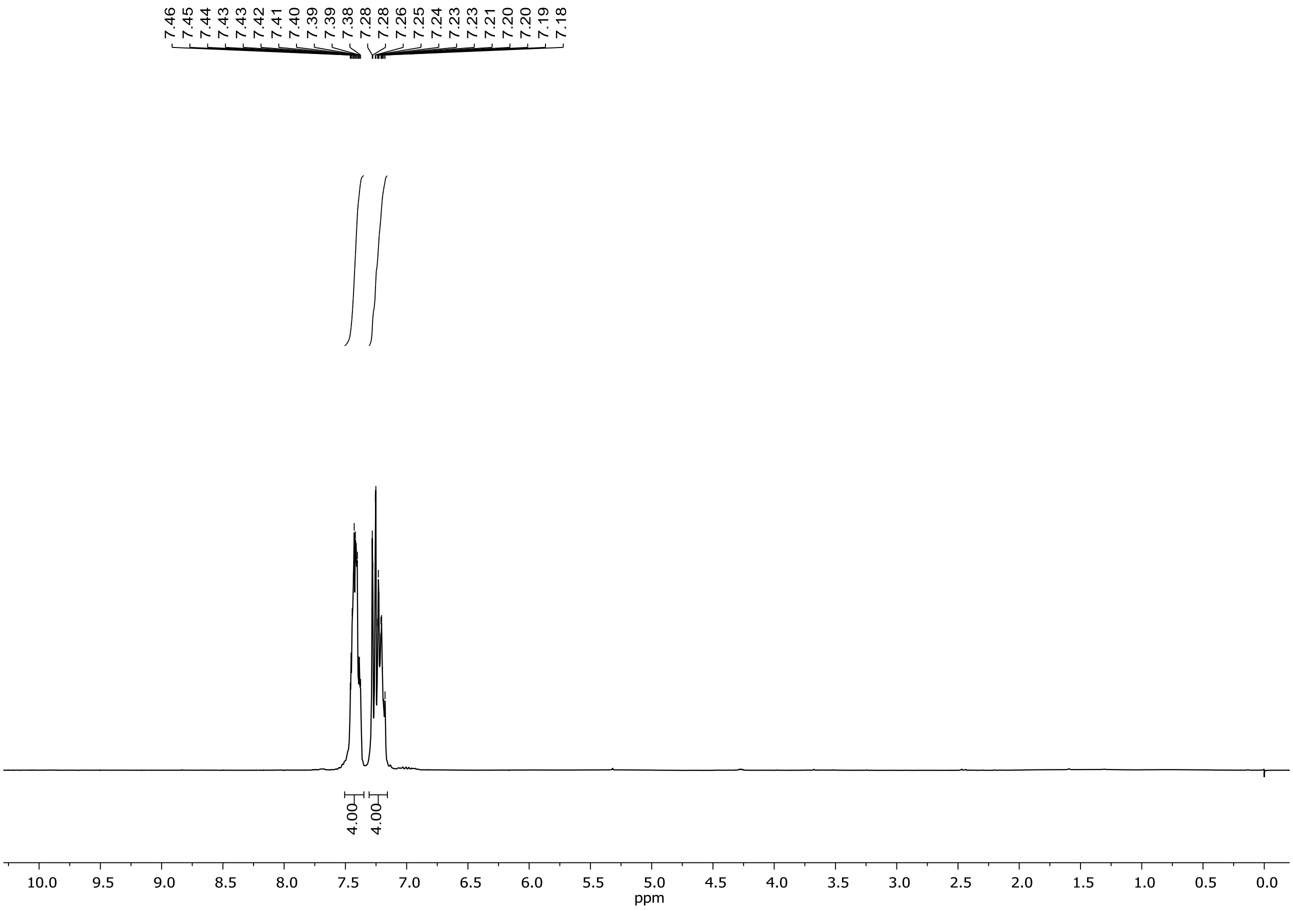

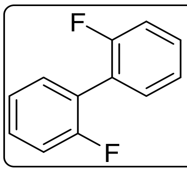

23

$^{13}C\{^{1}H\}$-NMR (75 MHz, $CDCl_3$)

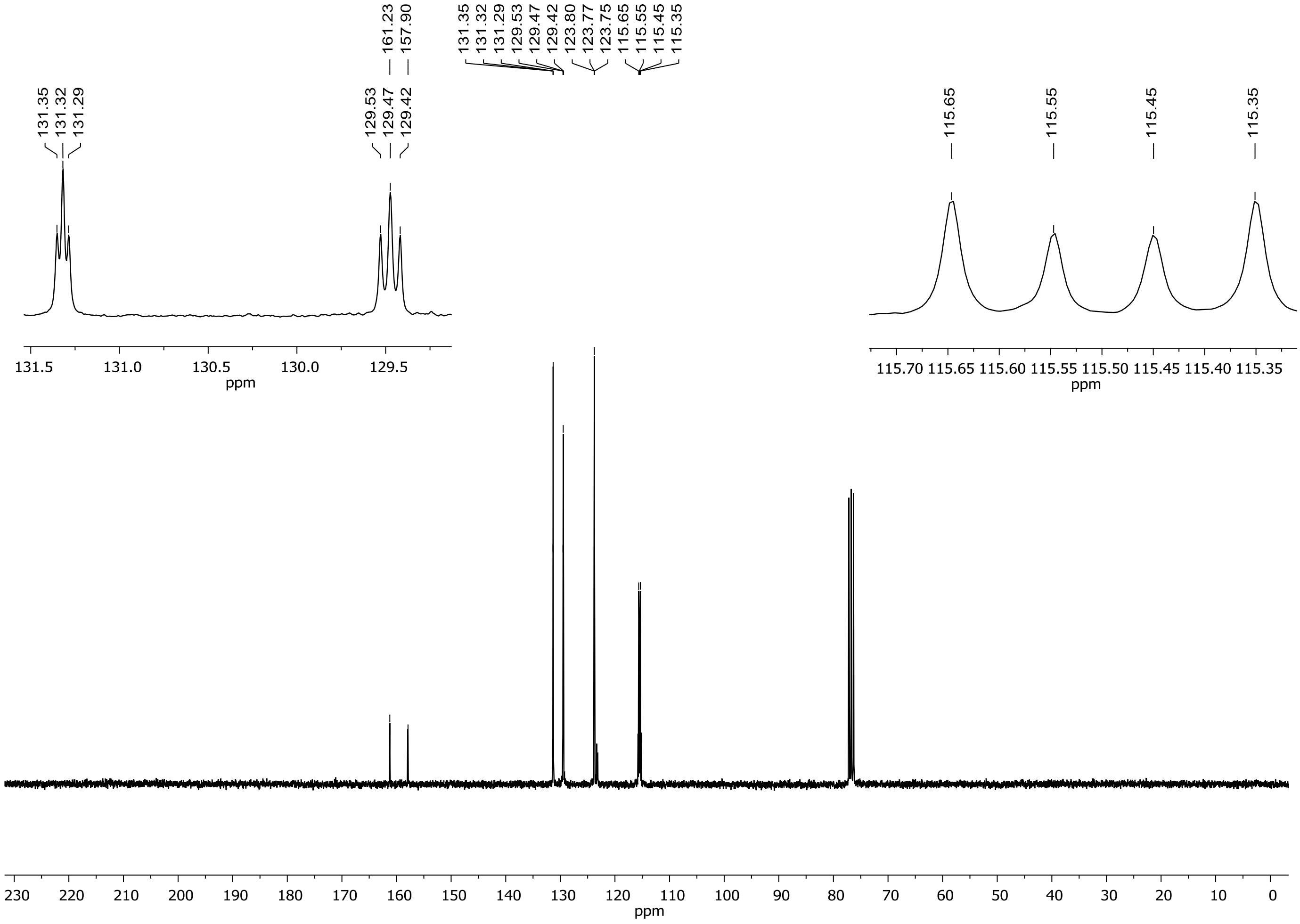

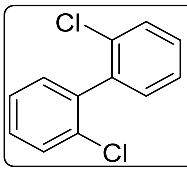

24

^{1}H-NMR (300 MHz, $CDCl_3$)

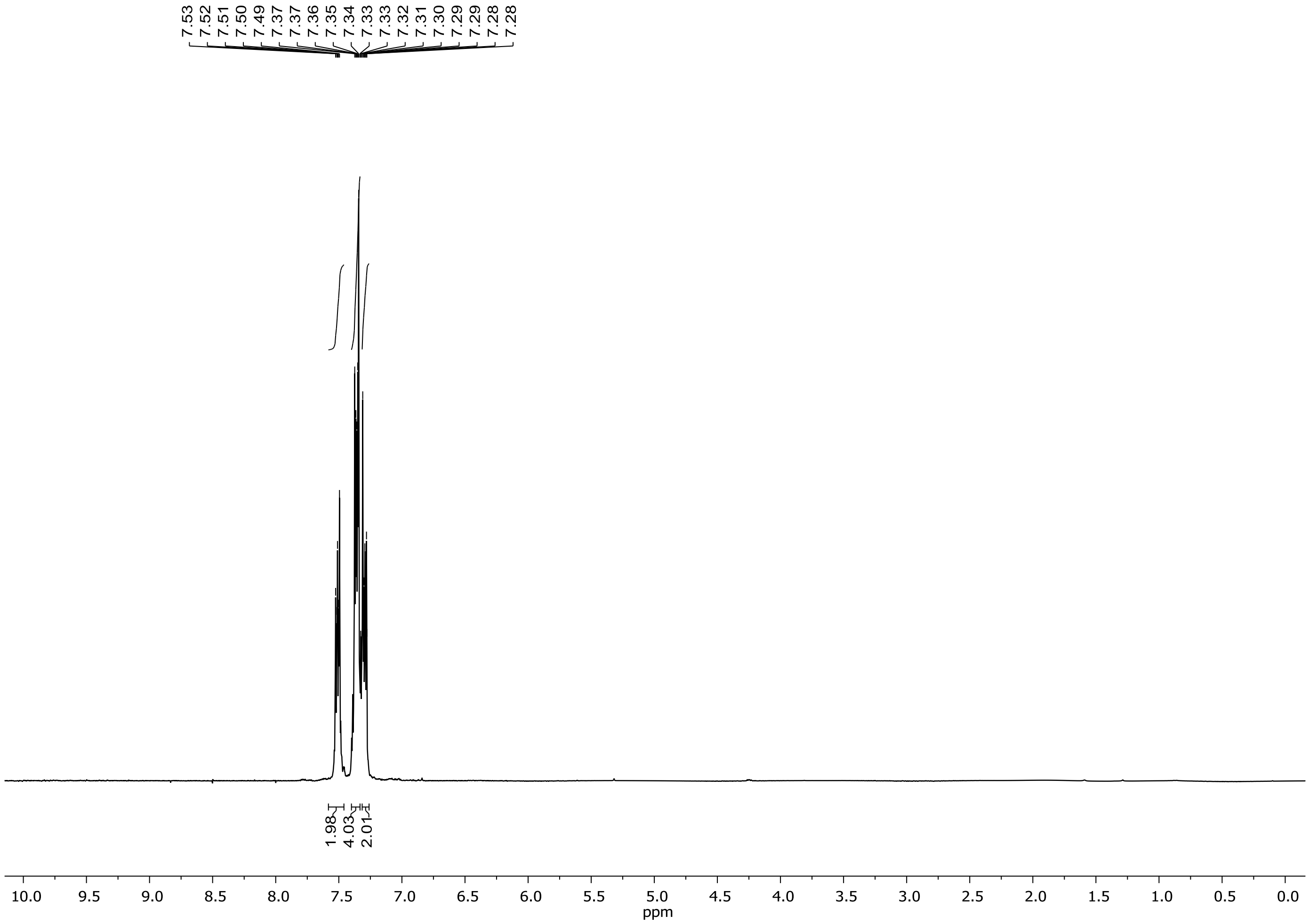

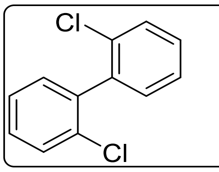

24 $^{13}C\{^1H\}$-NMR (75 MHz, $CDCl_3$)

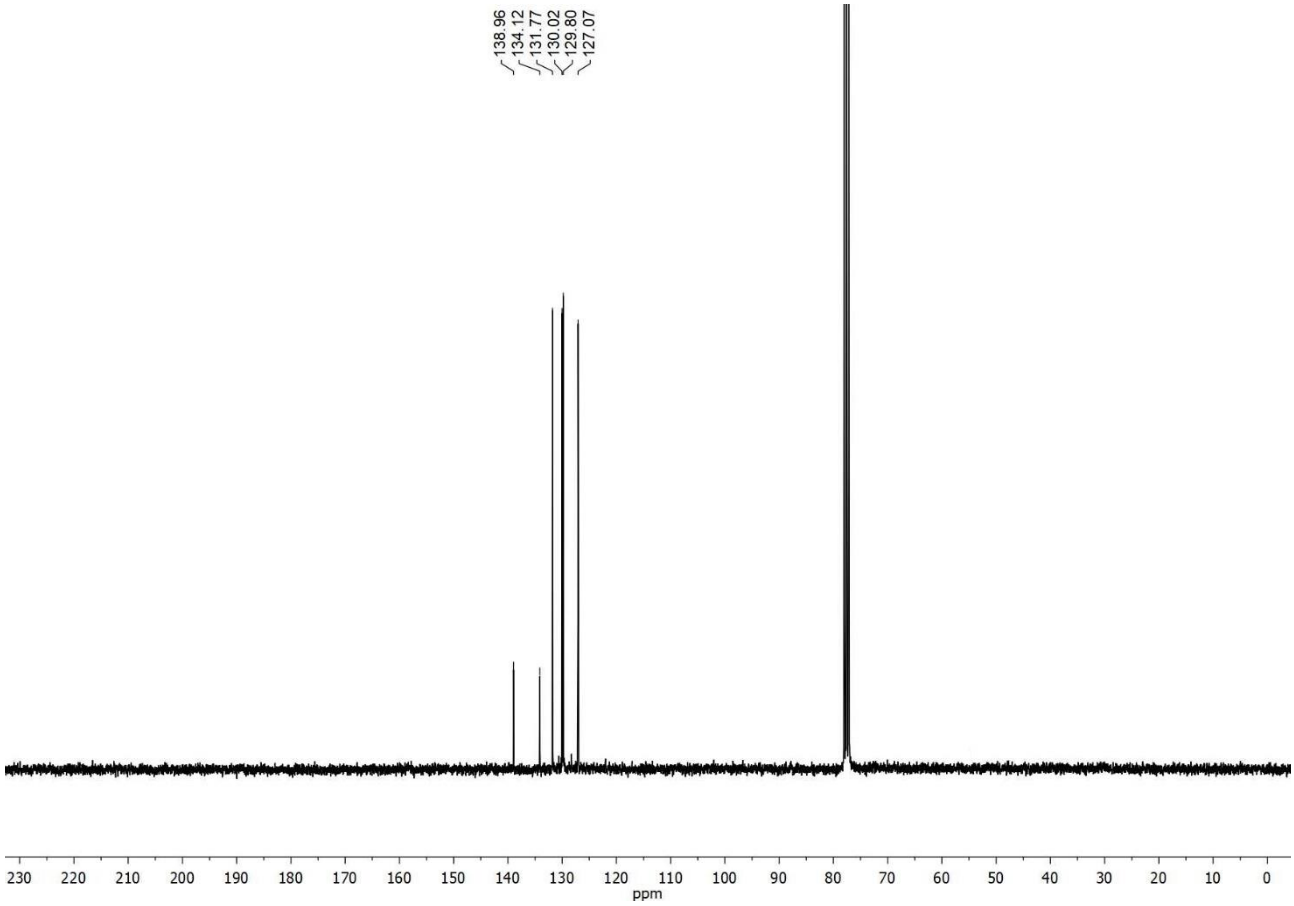

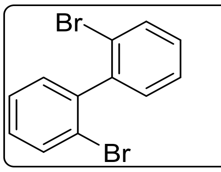

25

^{1}H-NMR (300 MHz, $CDCl_3$)

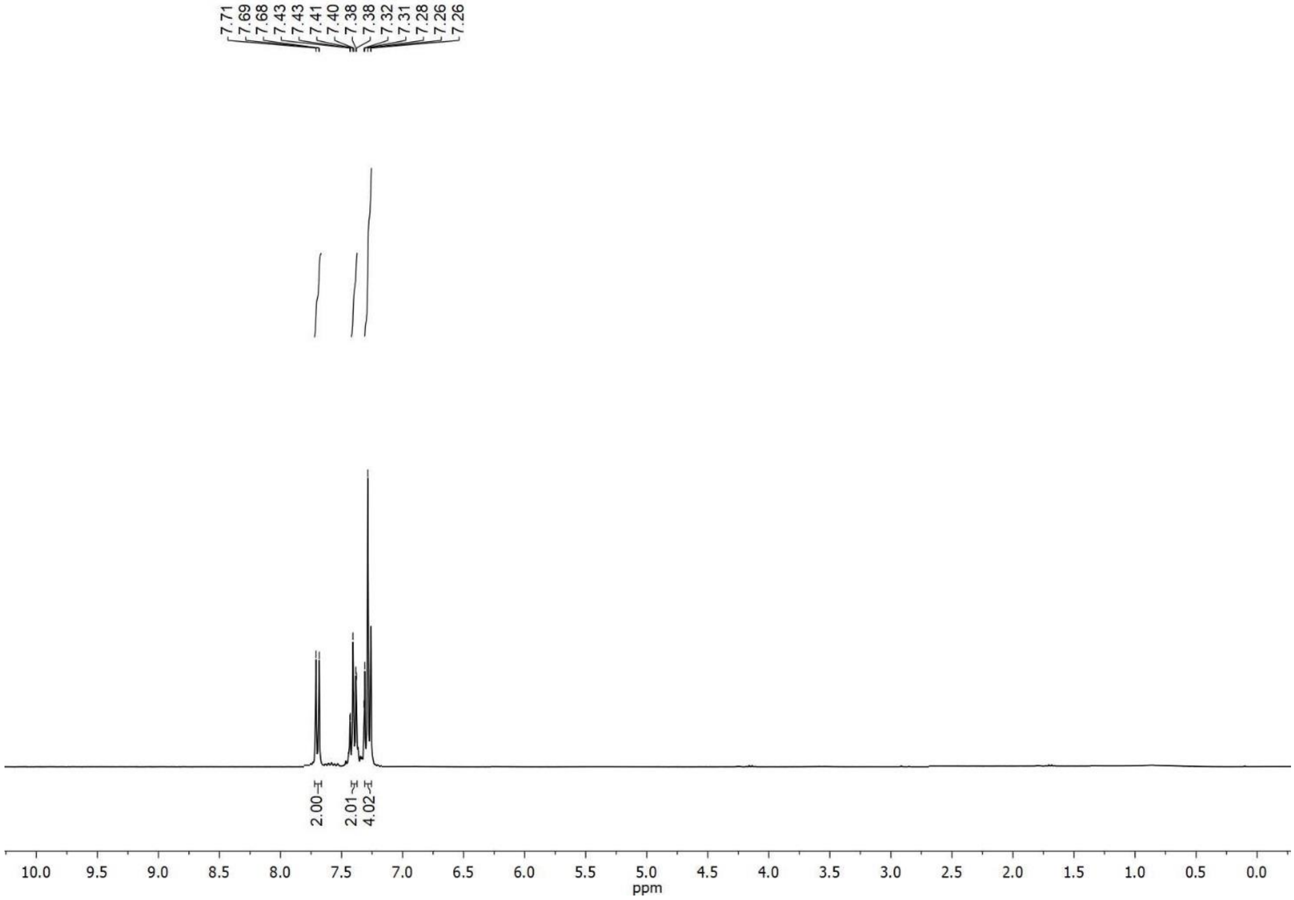

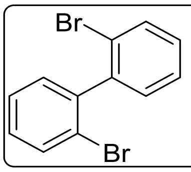

25

$^{13}C\{^{1}H\}$-NMR (75 MHz, $CDCl_3$)

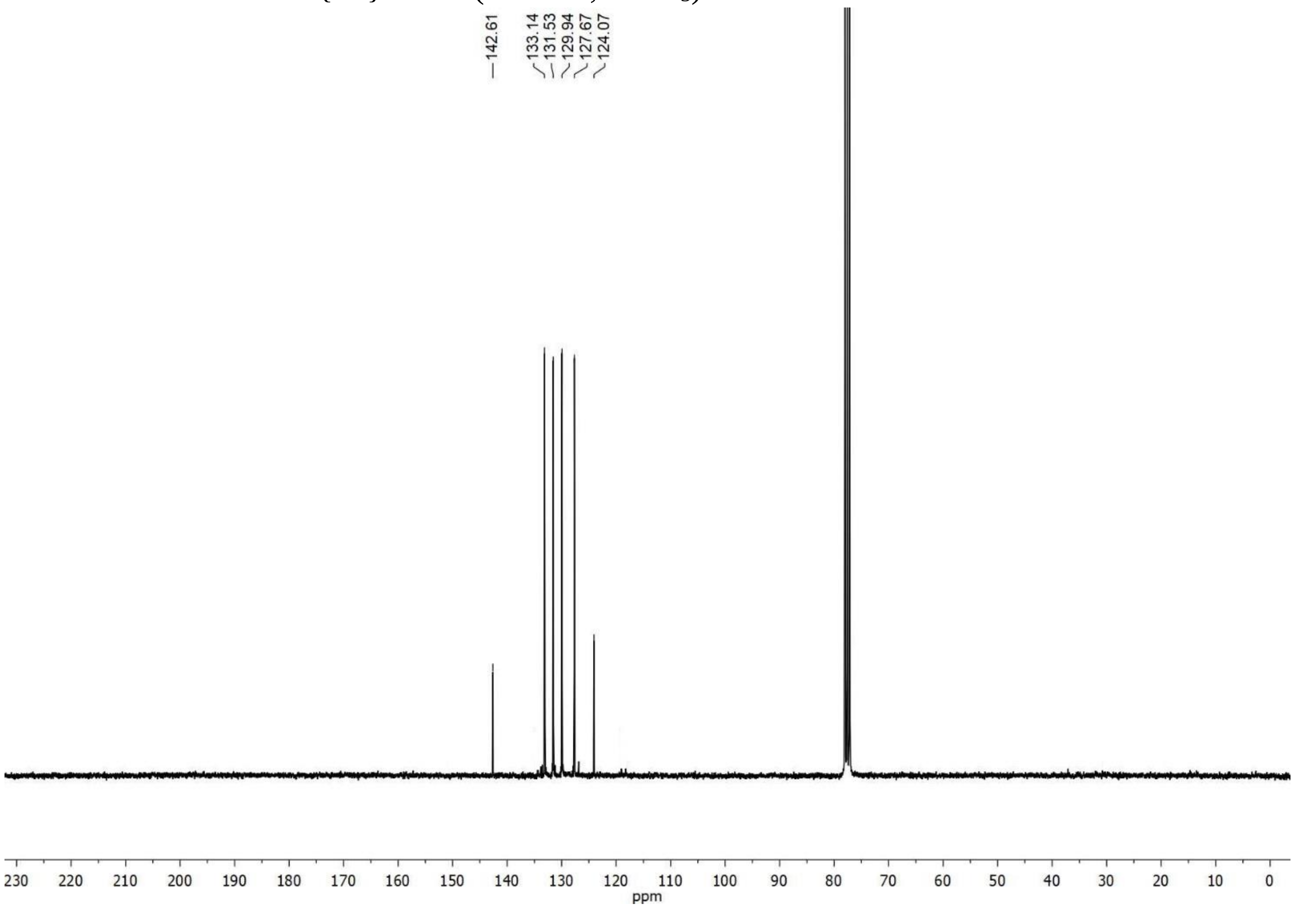

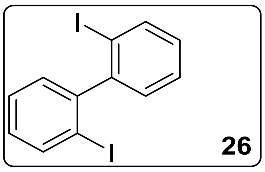

^{1}H-NMR (300 MHz, $CDCl_3$)

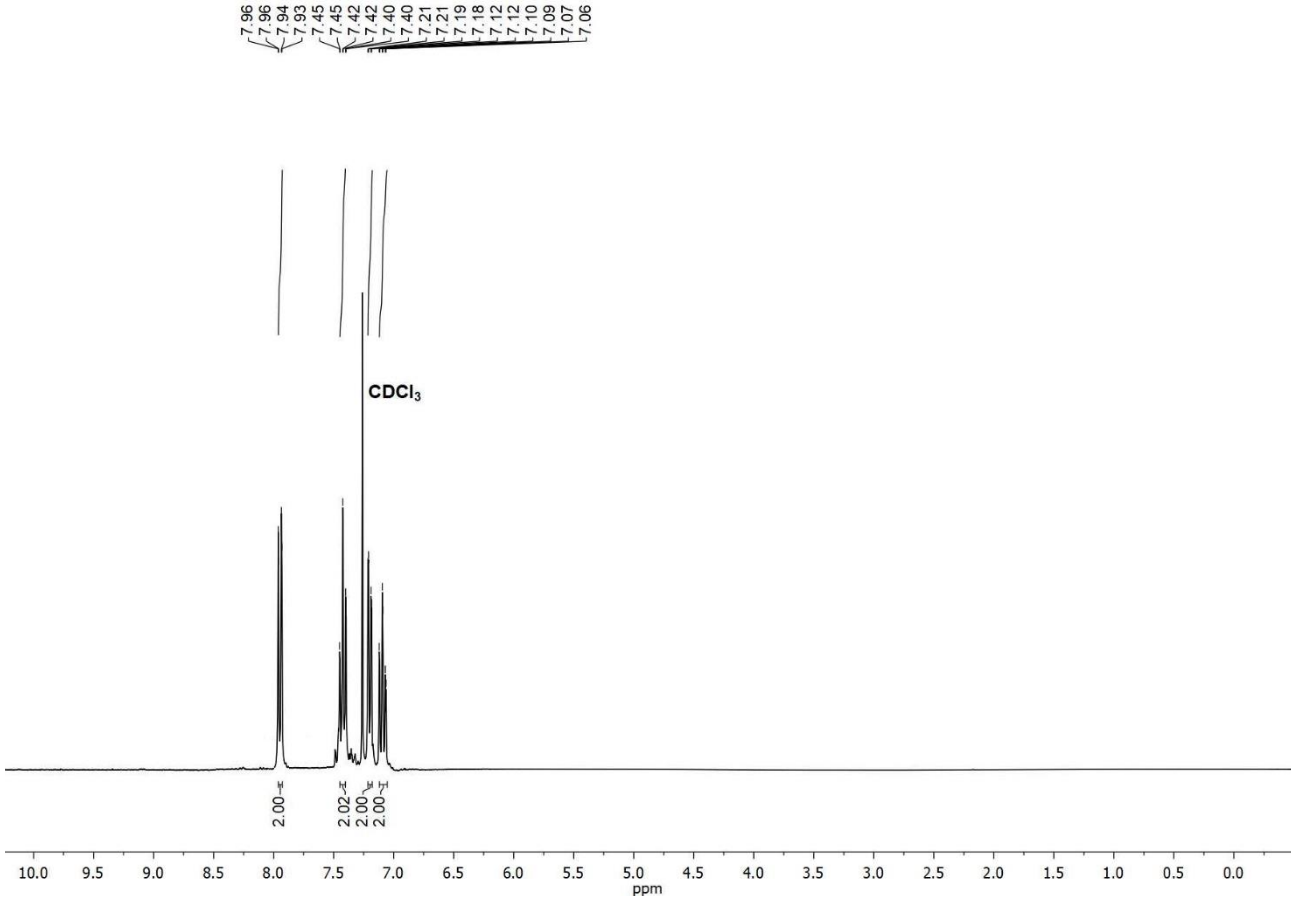

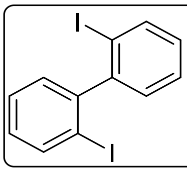

26 $^{13}C\{^{1}H\}$-NMR (75 MHz, $CDCl_3$)

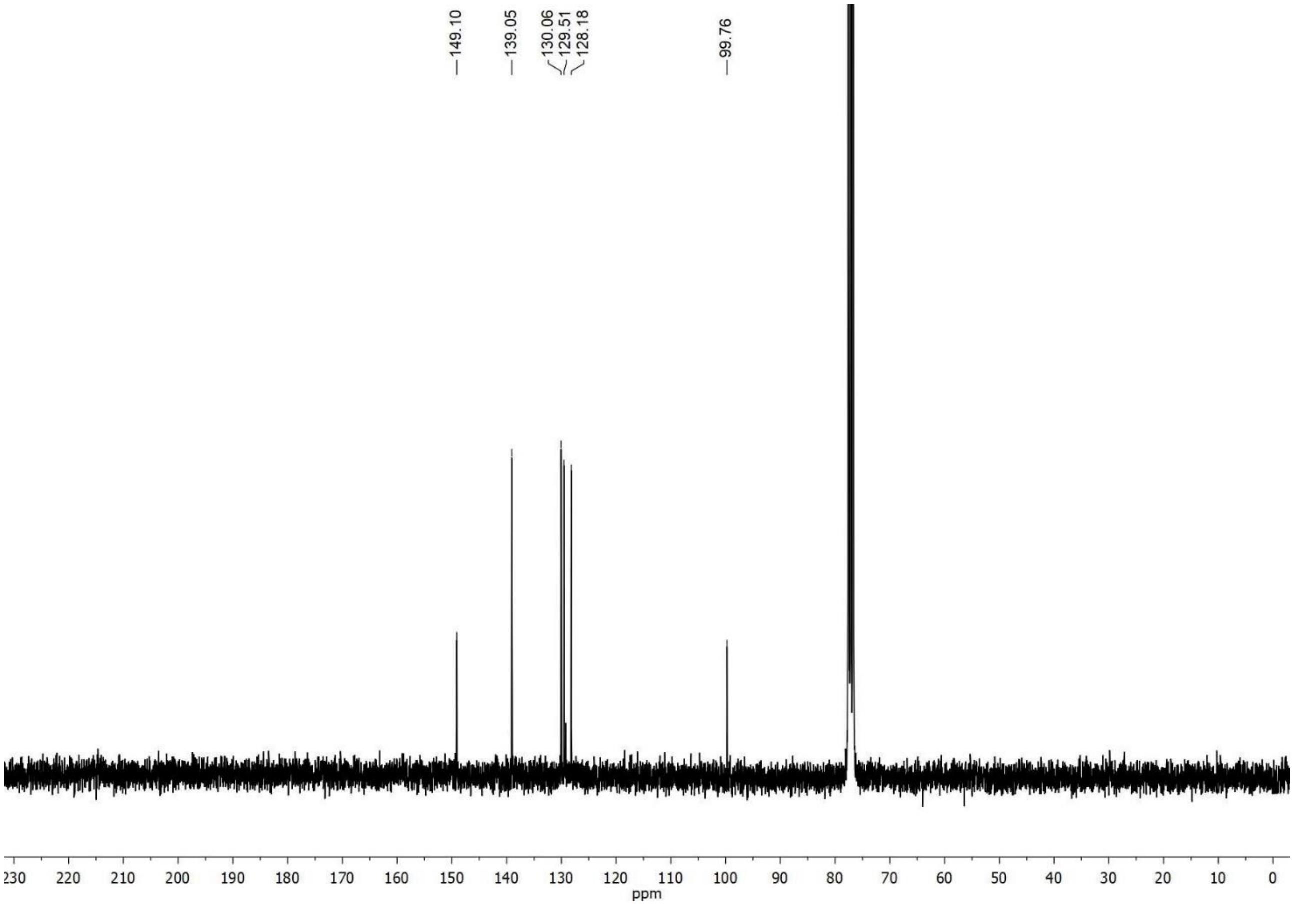

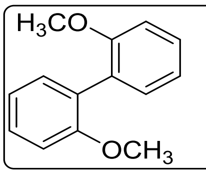

27

^{1}H-NMR (300 MHz, $CDCl_3$)

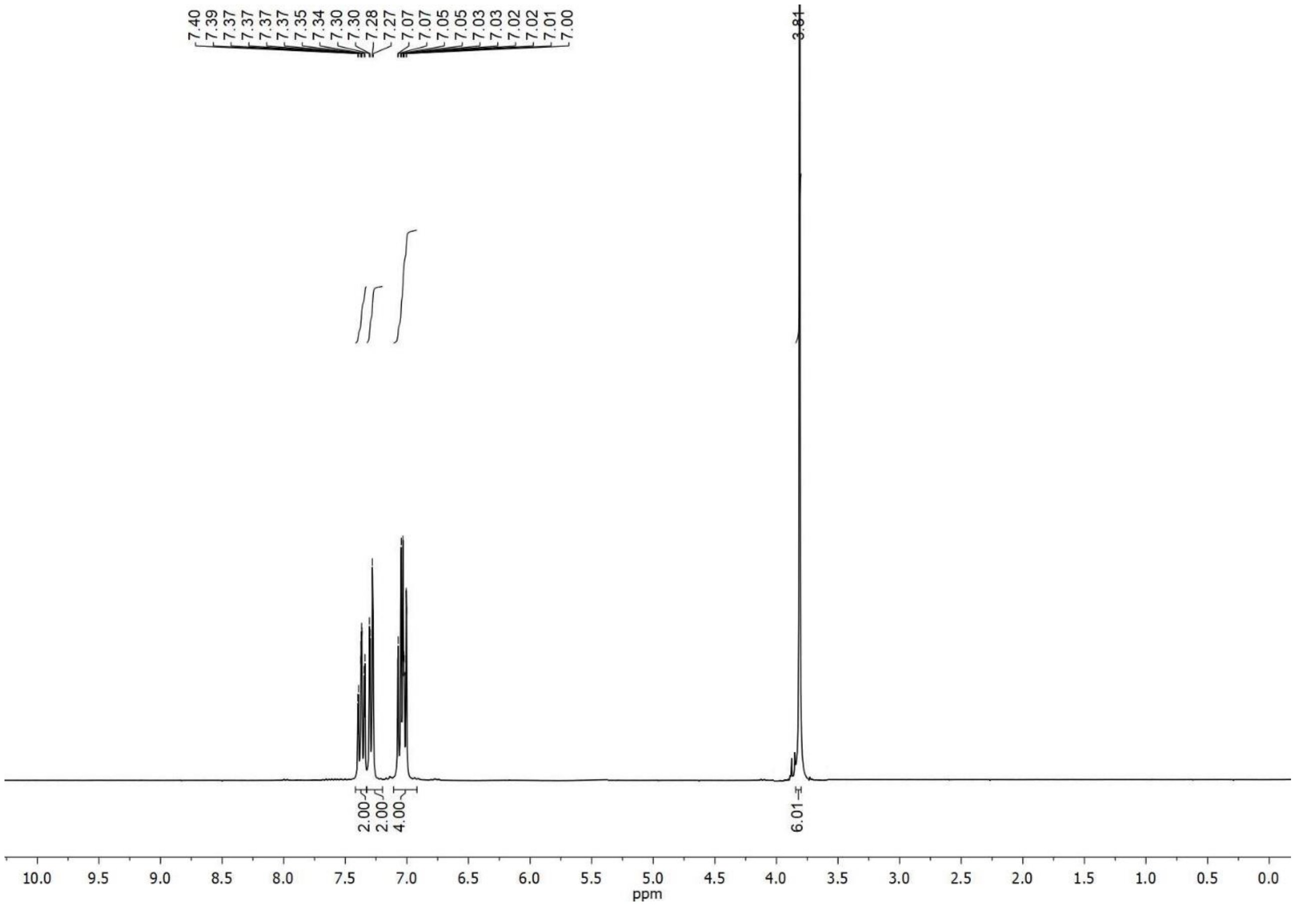

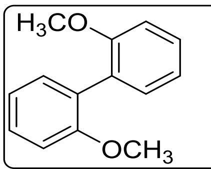

27

$^{13}C\{^{1}H\}$-NMR (75 MHz, $CDCl_3$)

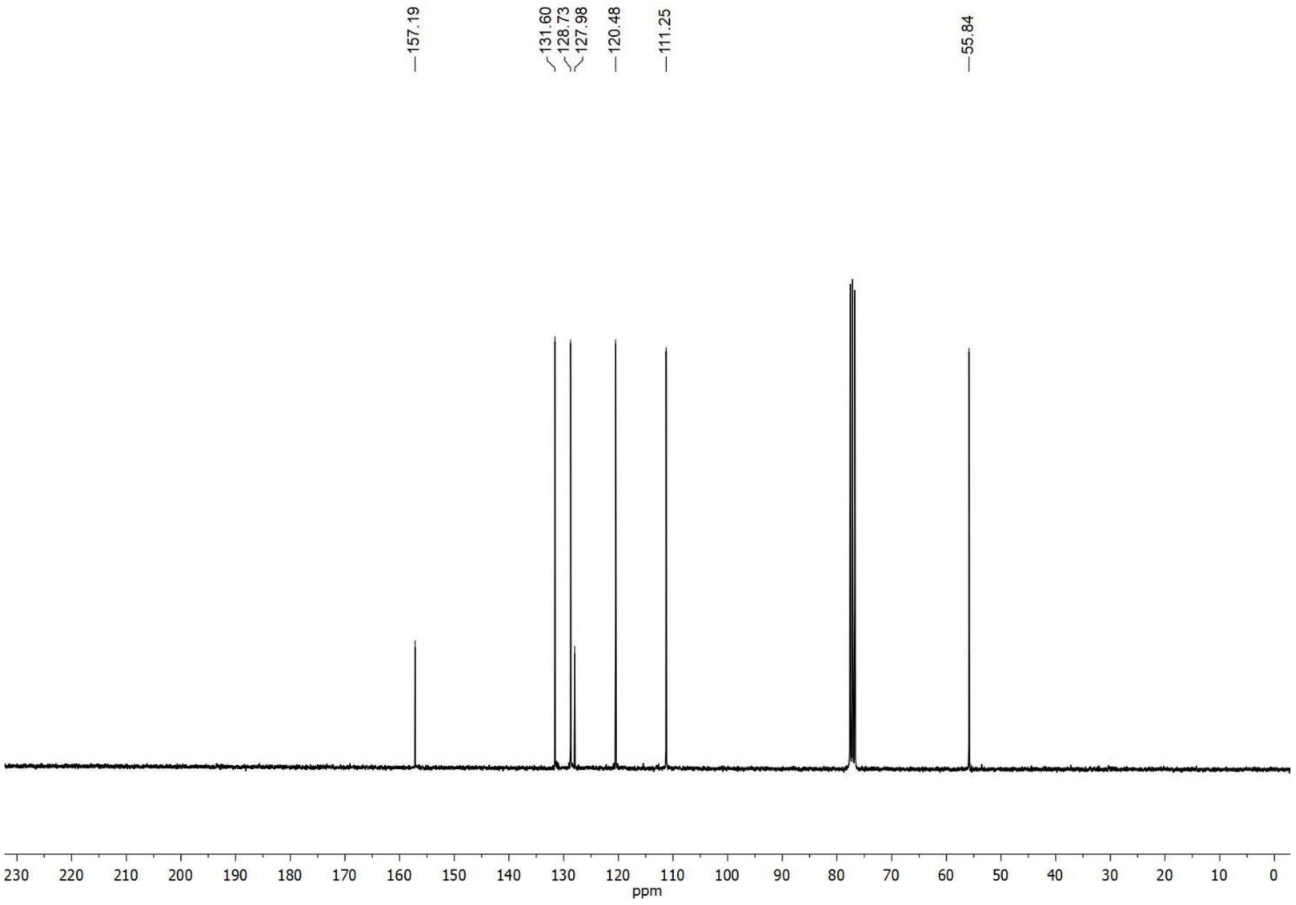

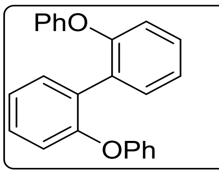

29 ^{1}H-NMR (300 MHz, $CDCl_3$)

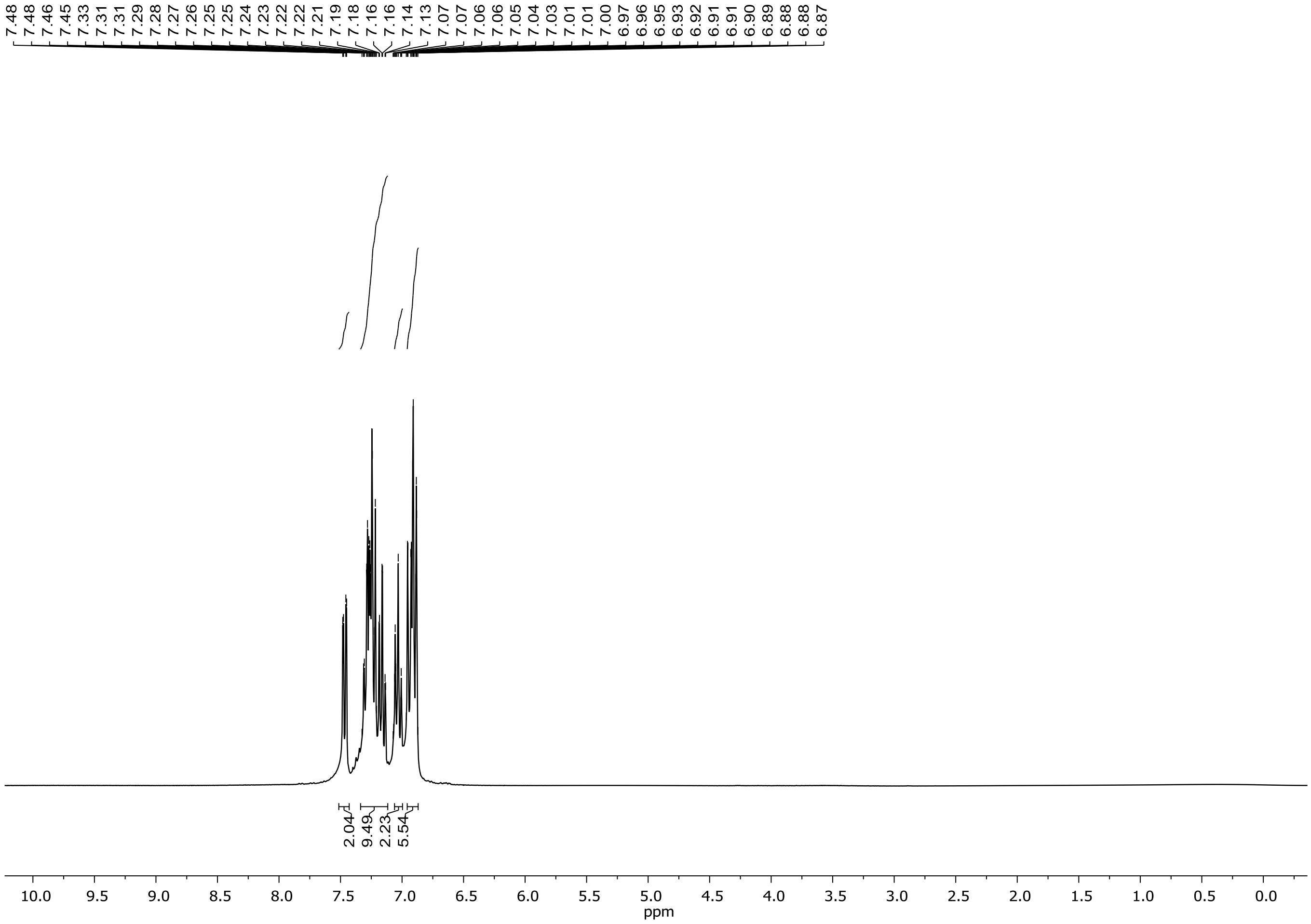

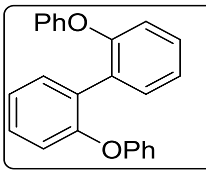

29 $^{13}C\{^{1}H\}$-NMR (75 MHz, $CDCl_3$)

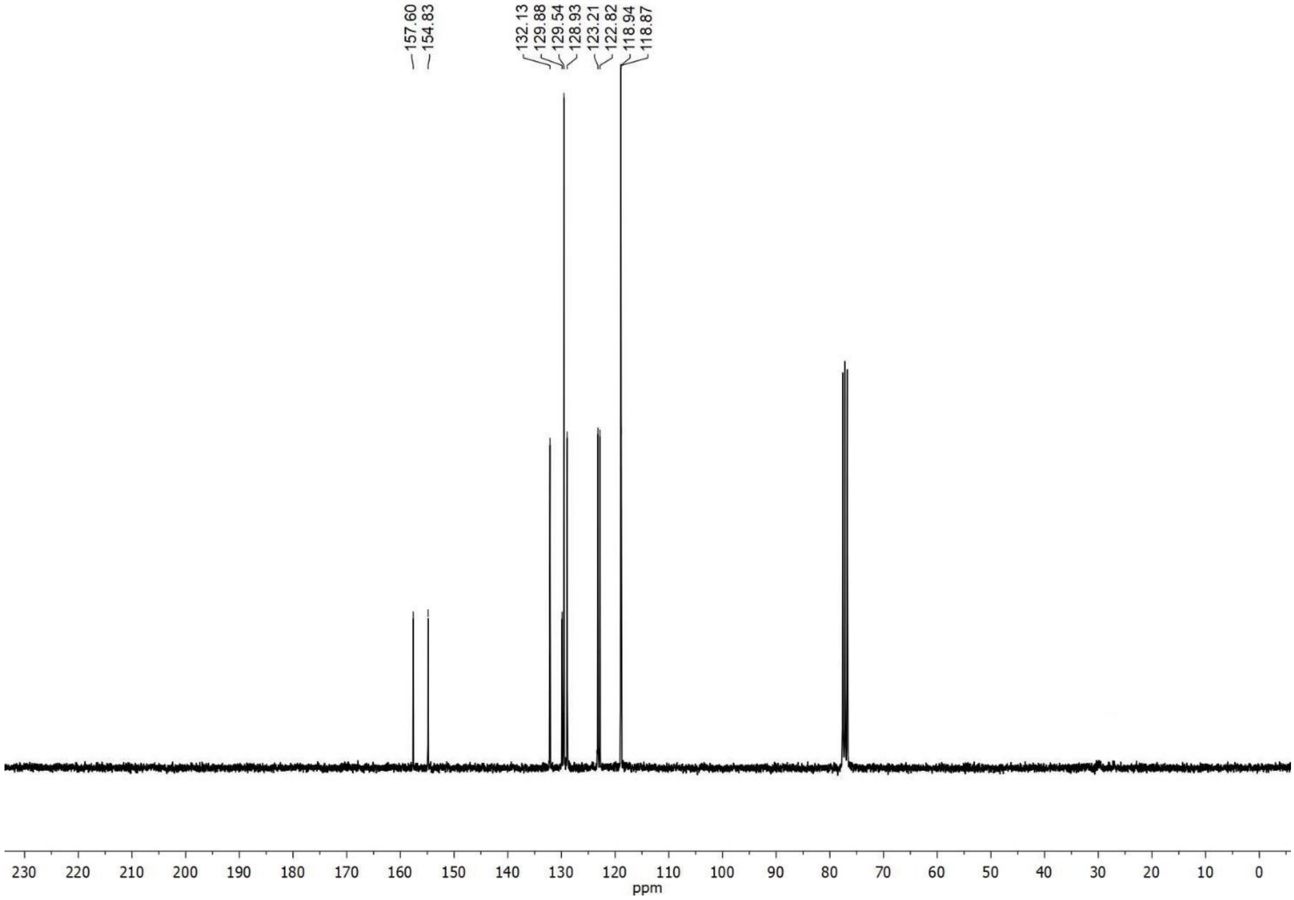

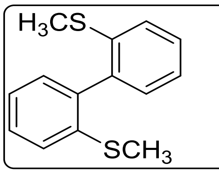

30

^{1}H-NMR (300 MHz, $CDCl_3$)

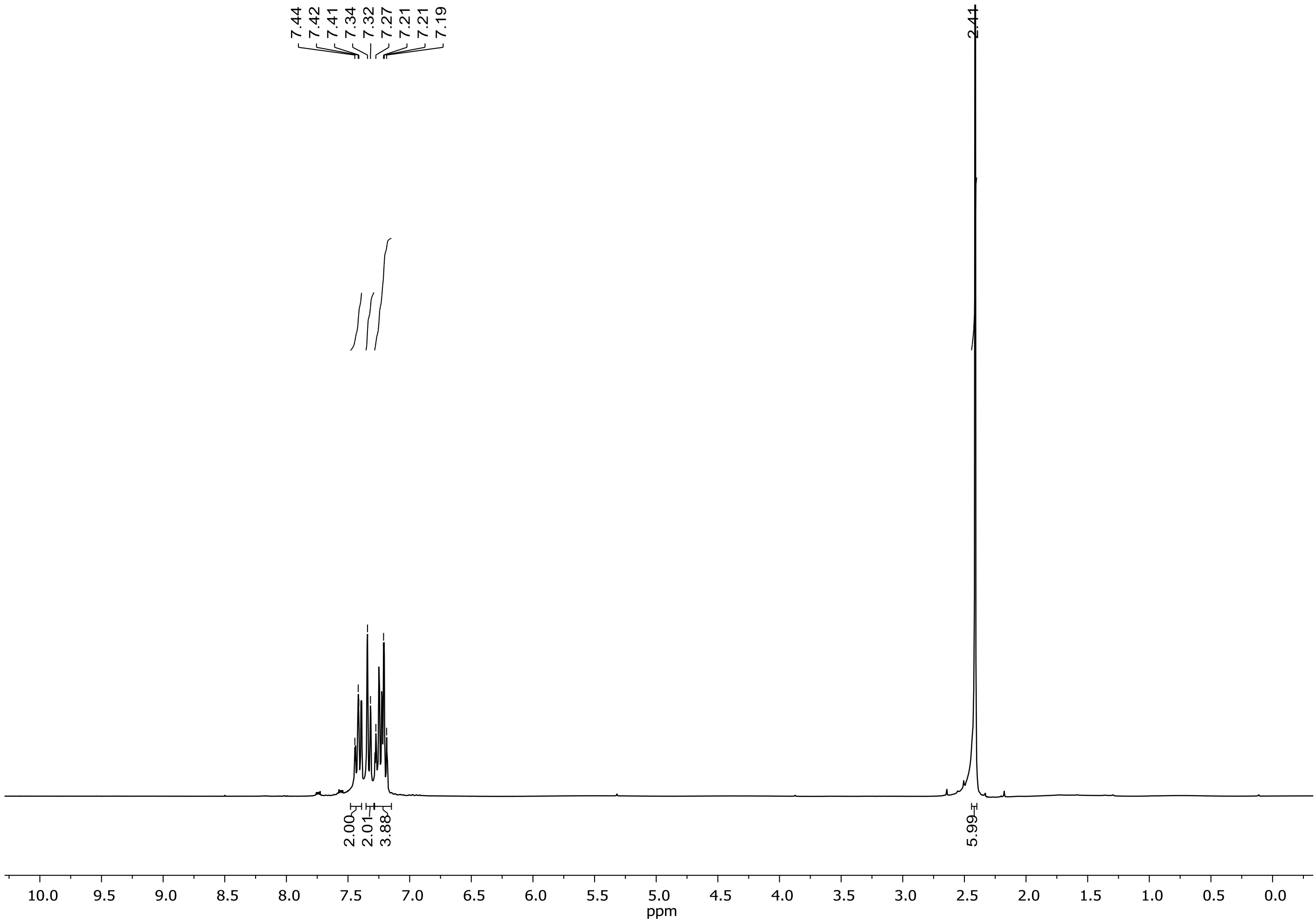

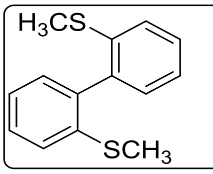

30 $^{13}C\{^{1}H\}$-NMR (75 MHz, $CDCl_3$)

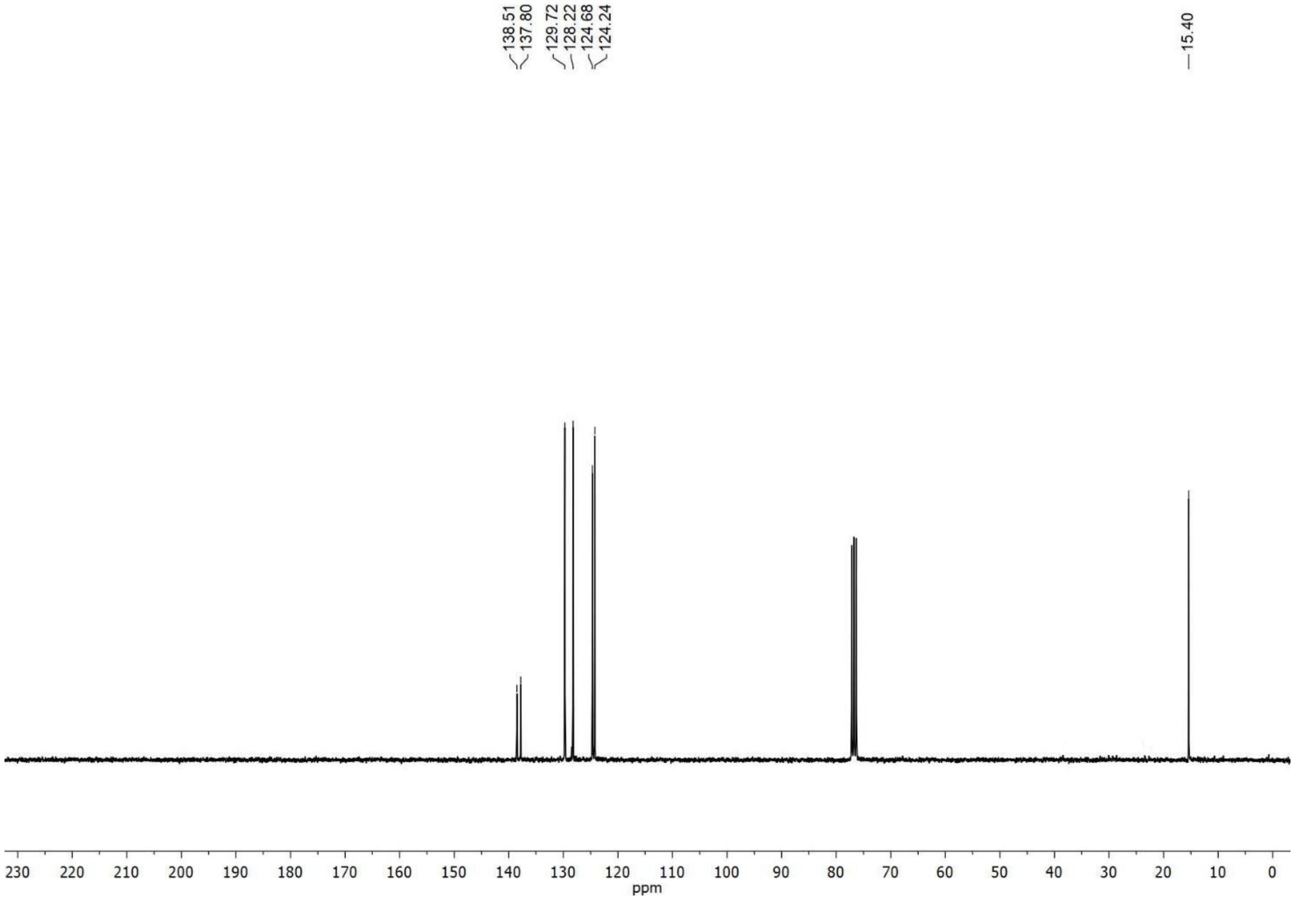

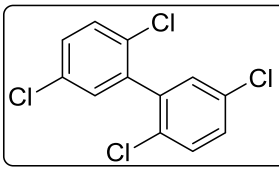

31 ^{1}H-NMR (300 MHz, $CDCl_3$)

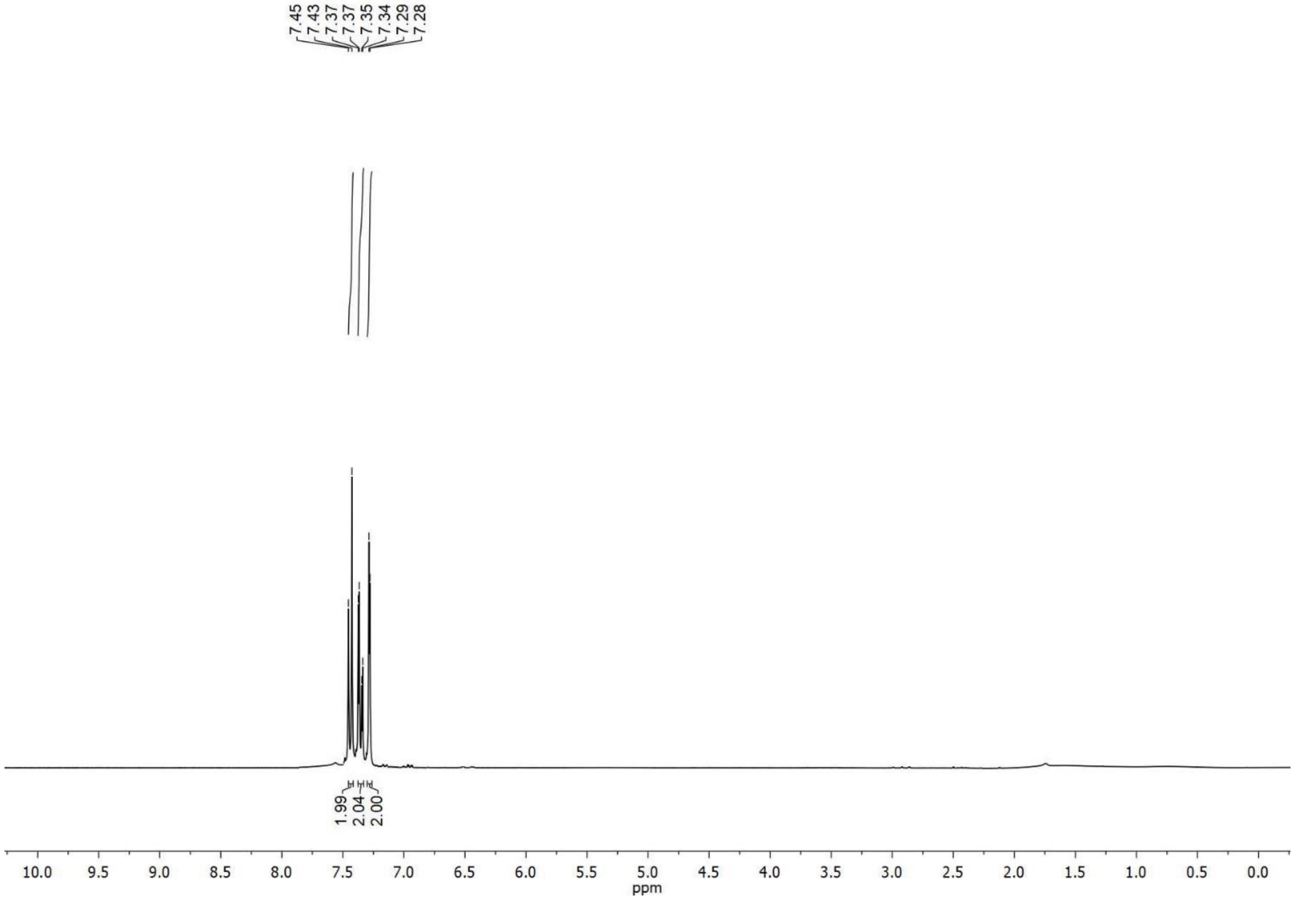

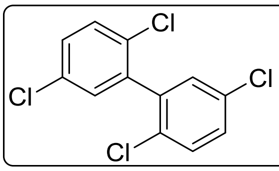

31 $^{13}C\{^{1}H\}$-NMR (75 MHz, $CDCl_3$)

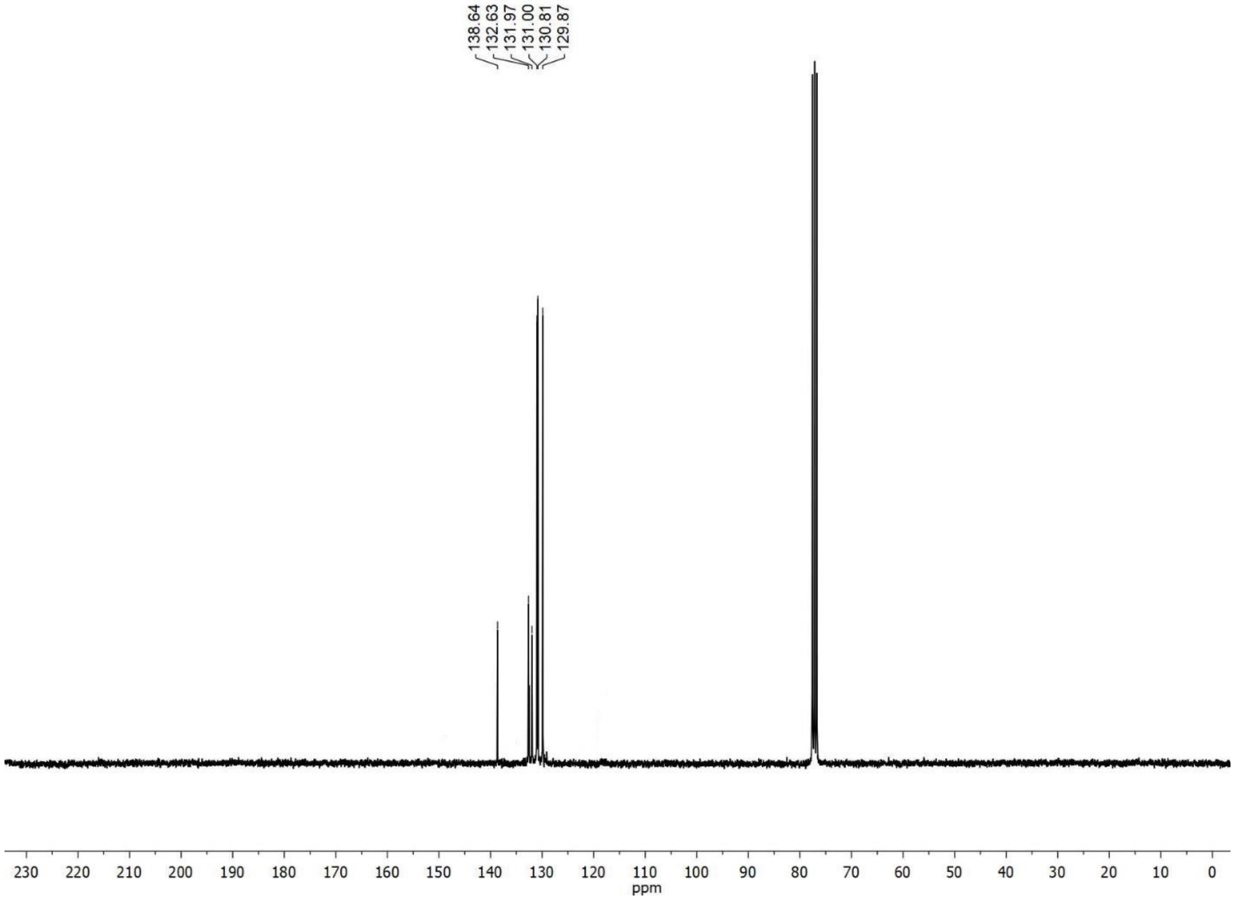

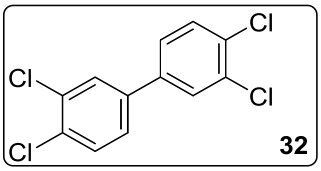

32 ^{1}H-NMR (300 MHz, $CDCl_3$)

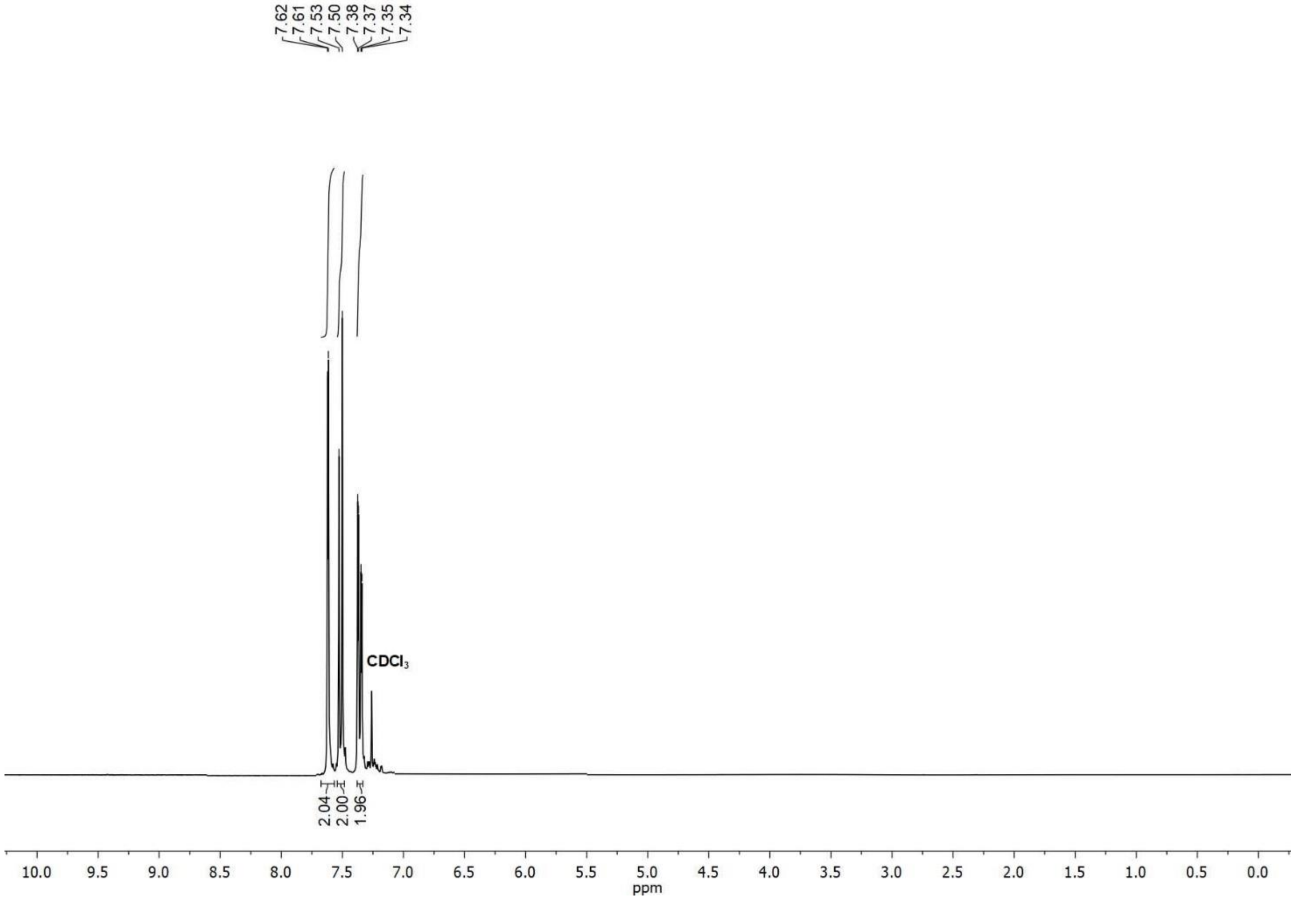

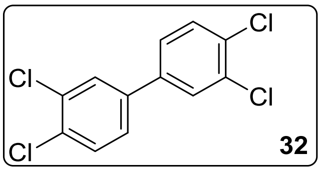

32 $^{13}C\{^{1}H\}$-NMR (75 MHz, $CDCl_3$)

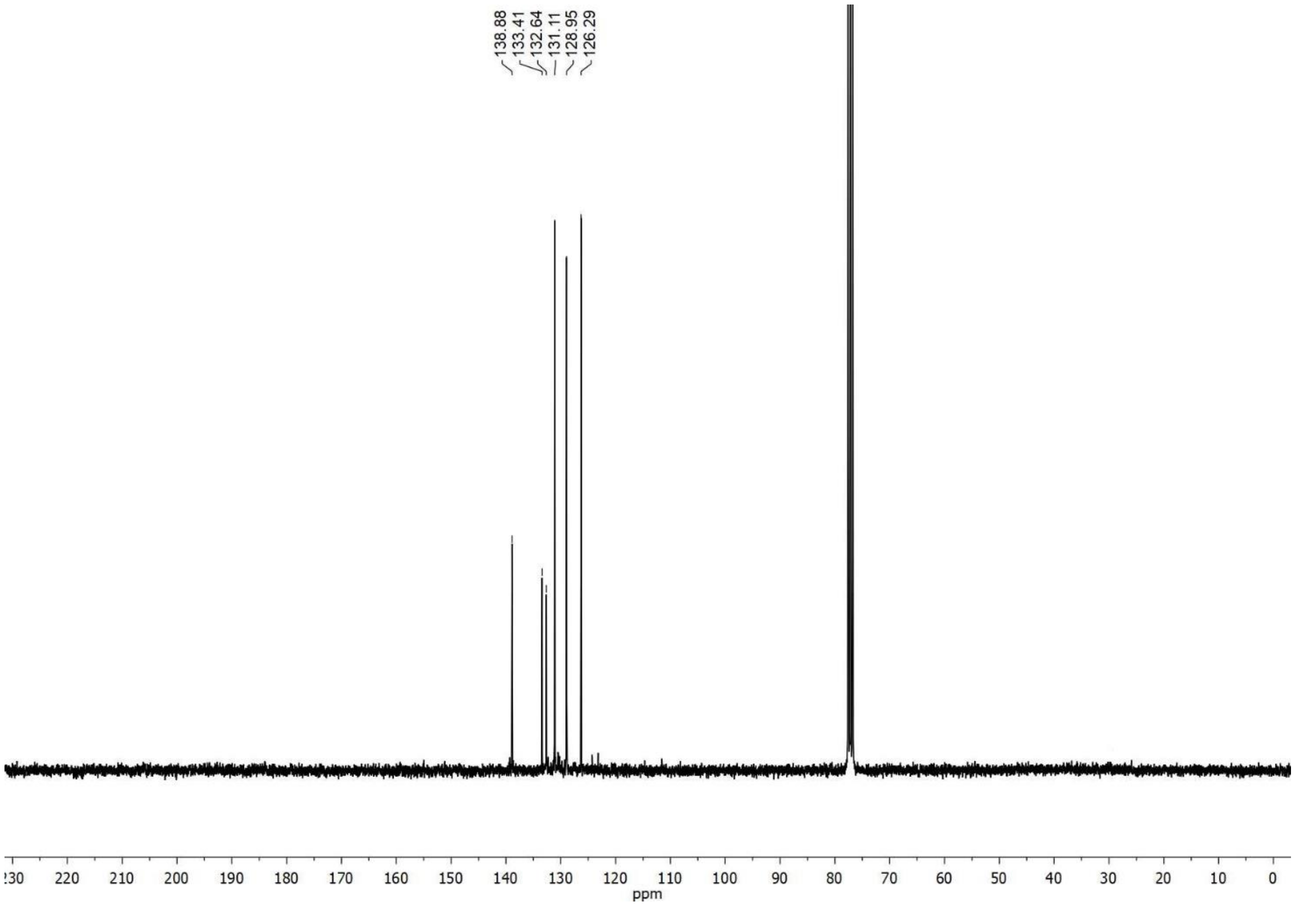

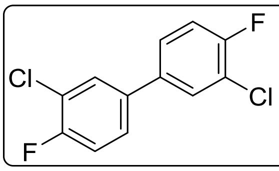

33

^{1}H-NMR (300 MHz, $CDCl_3$)

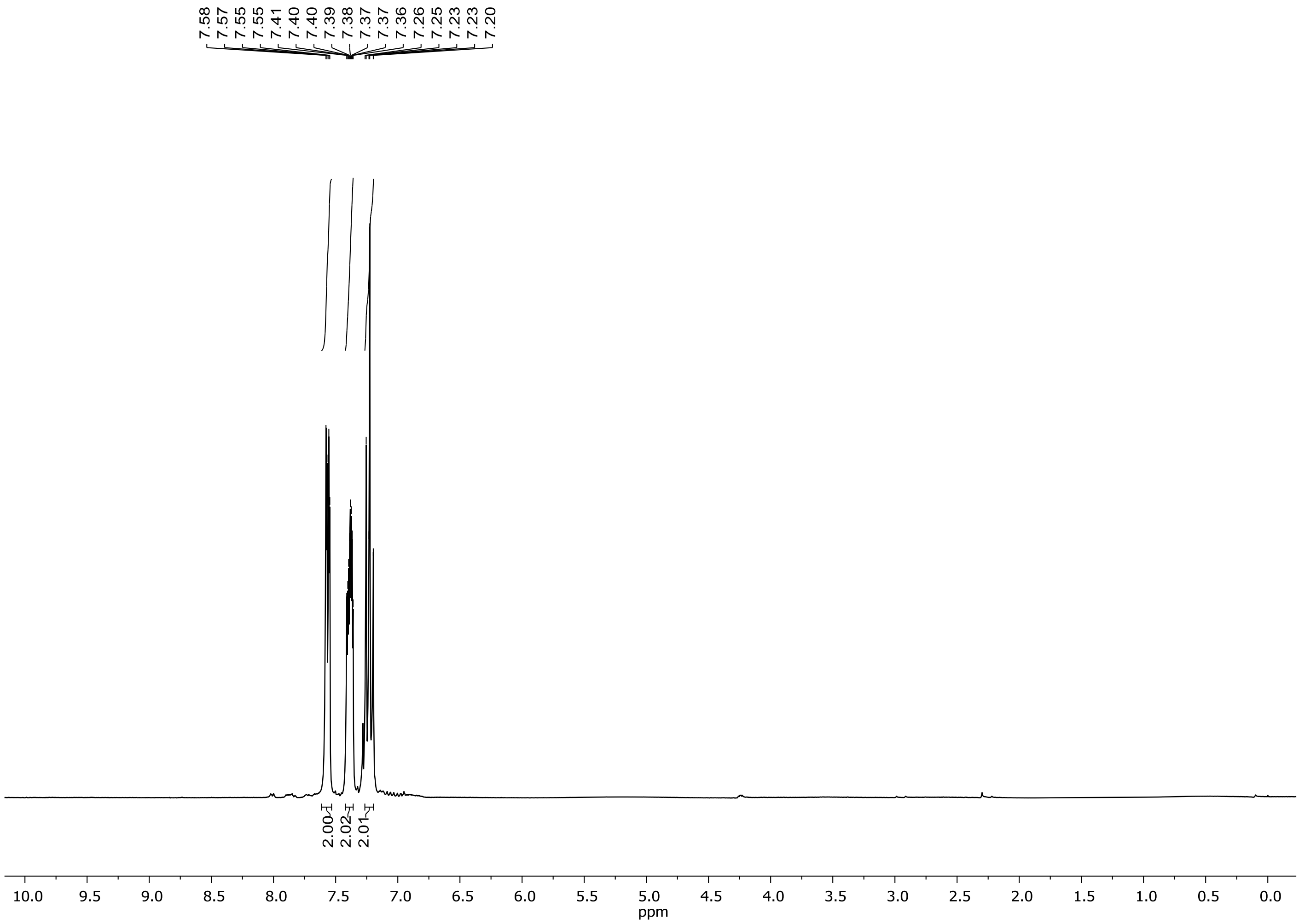

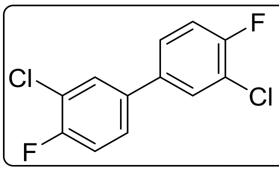

33

$^{13}C\{^{1}H\}$-NMR (75 MHz, $CDCl_3$)

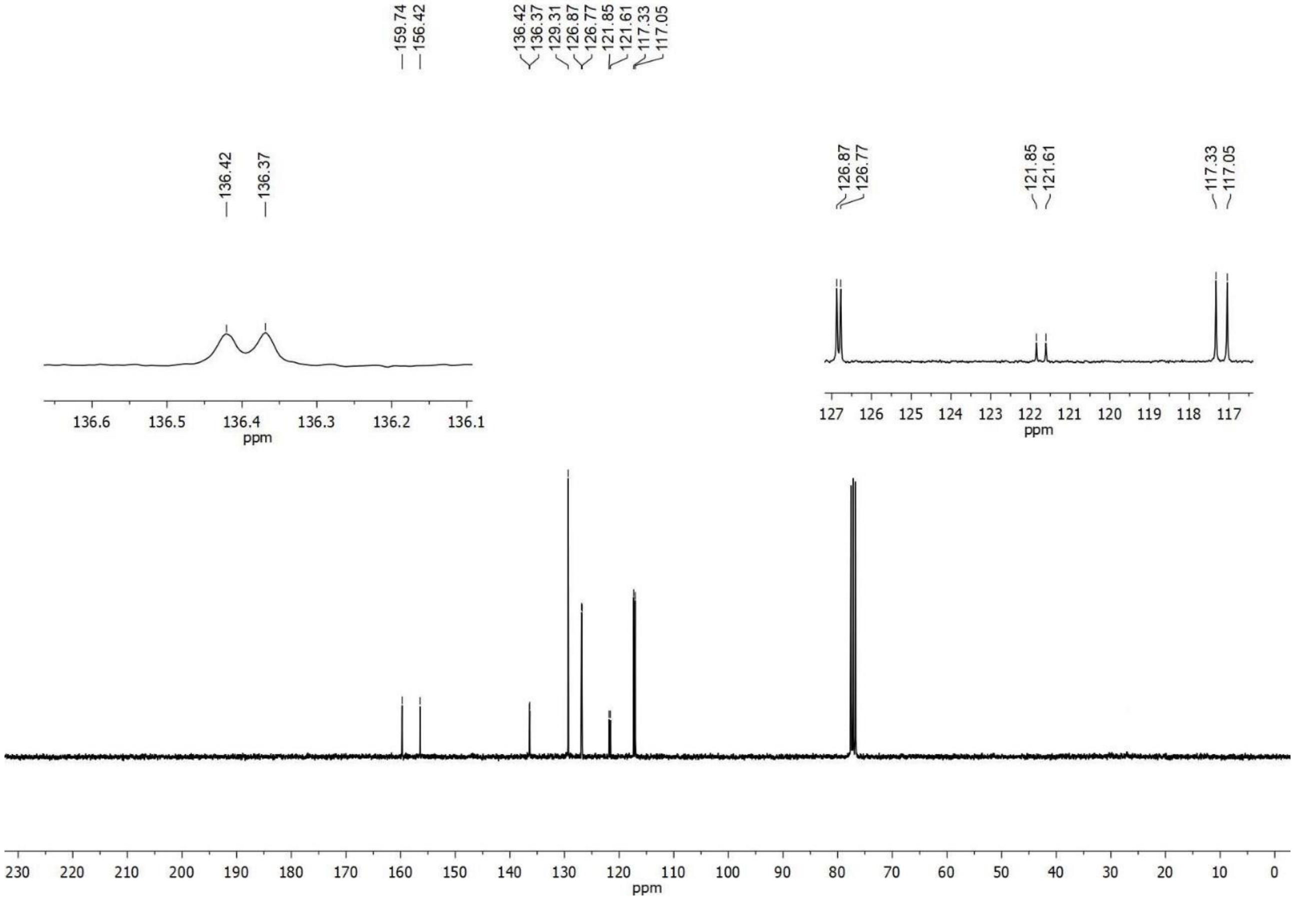

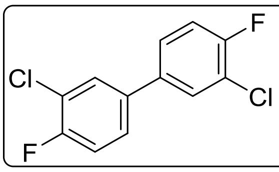

33

^{19}F-NMR (376 MHz, $CDCl_3$)

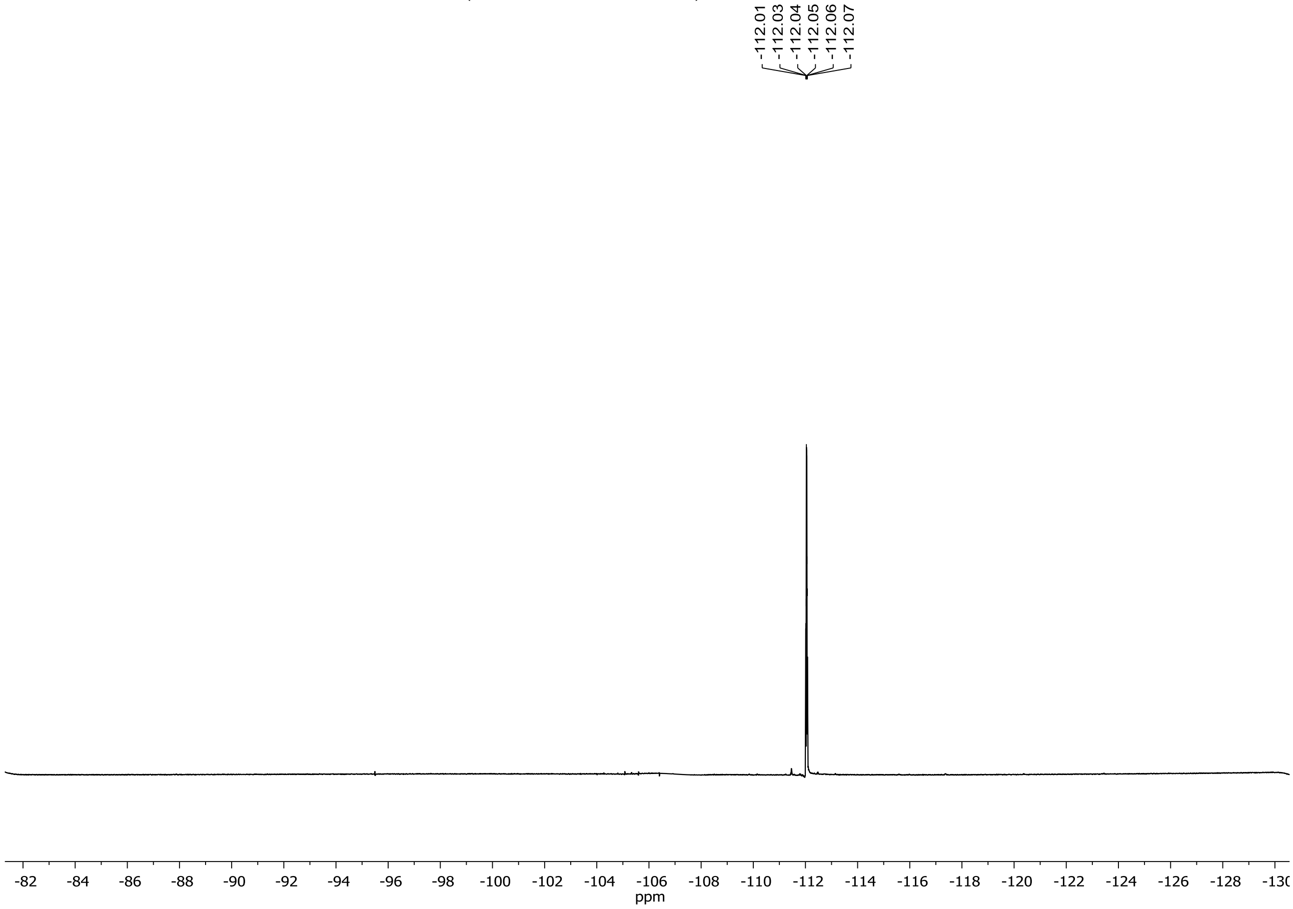

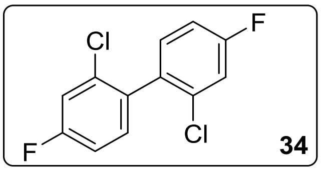

^{1}H-NMR (300 MHz, $CDCl_3$)

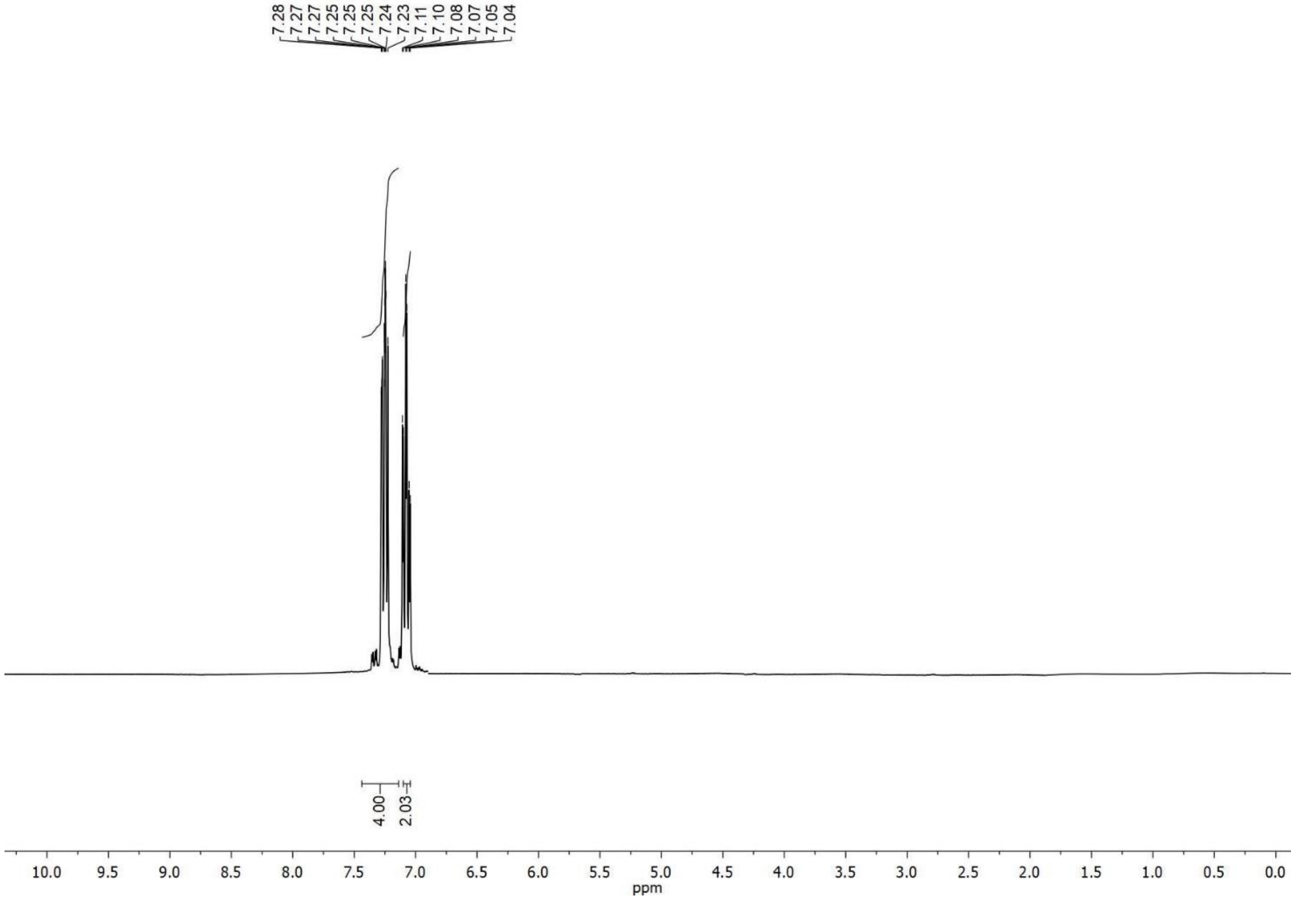

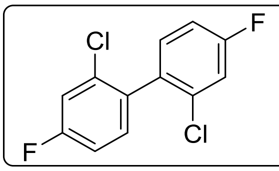

34 $^{13}C\{^{1}H\}$-NMR (75 MHz, $CDCl_3$)

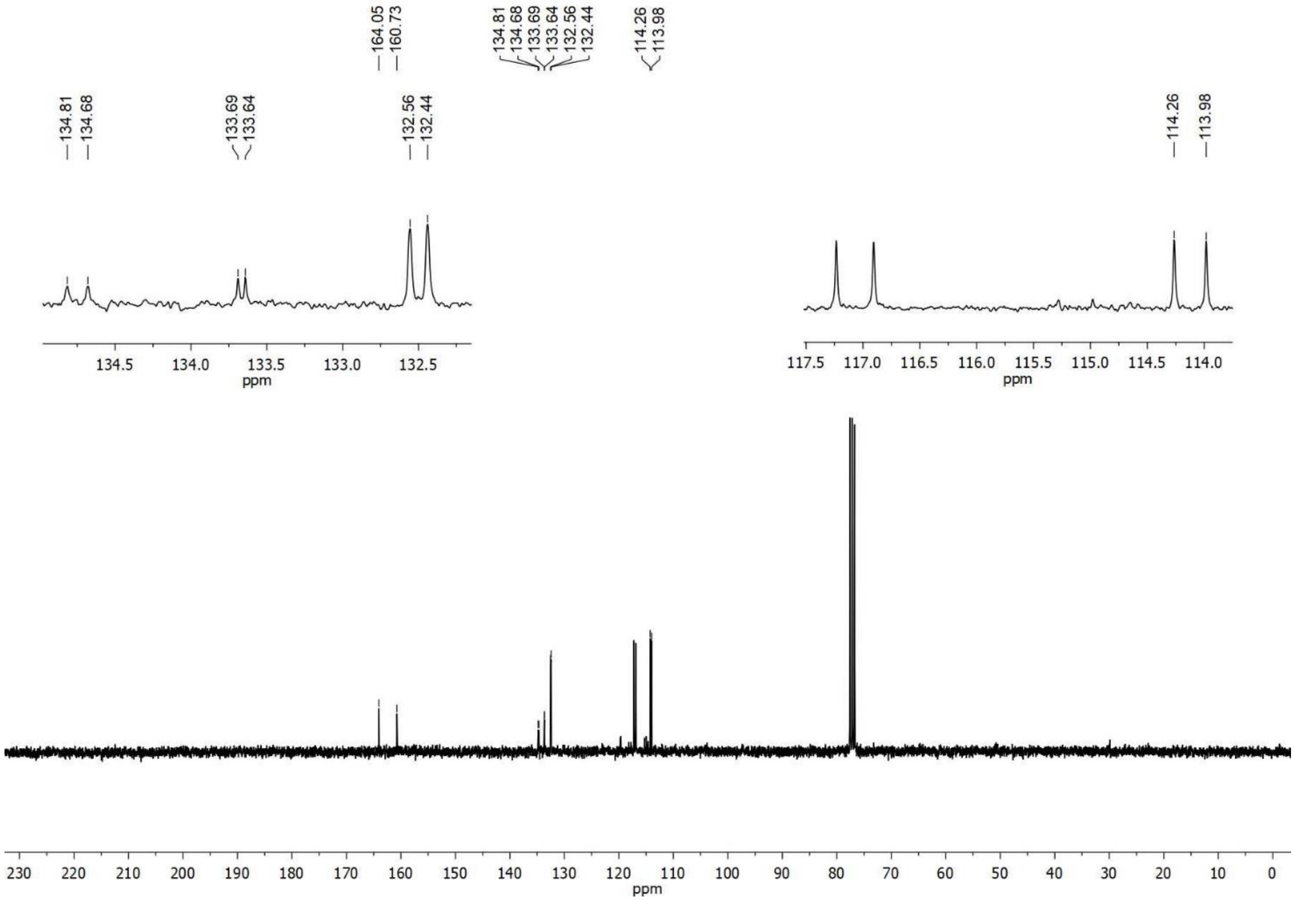

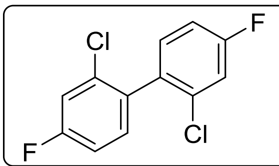

34 ^{19}F-NMR (376 MHz, $CDCl_3$)

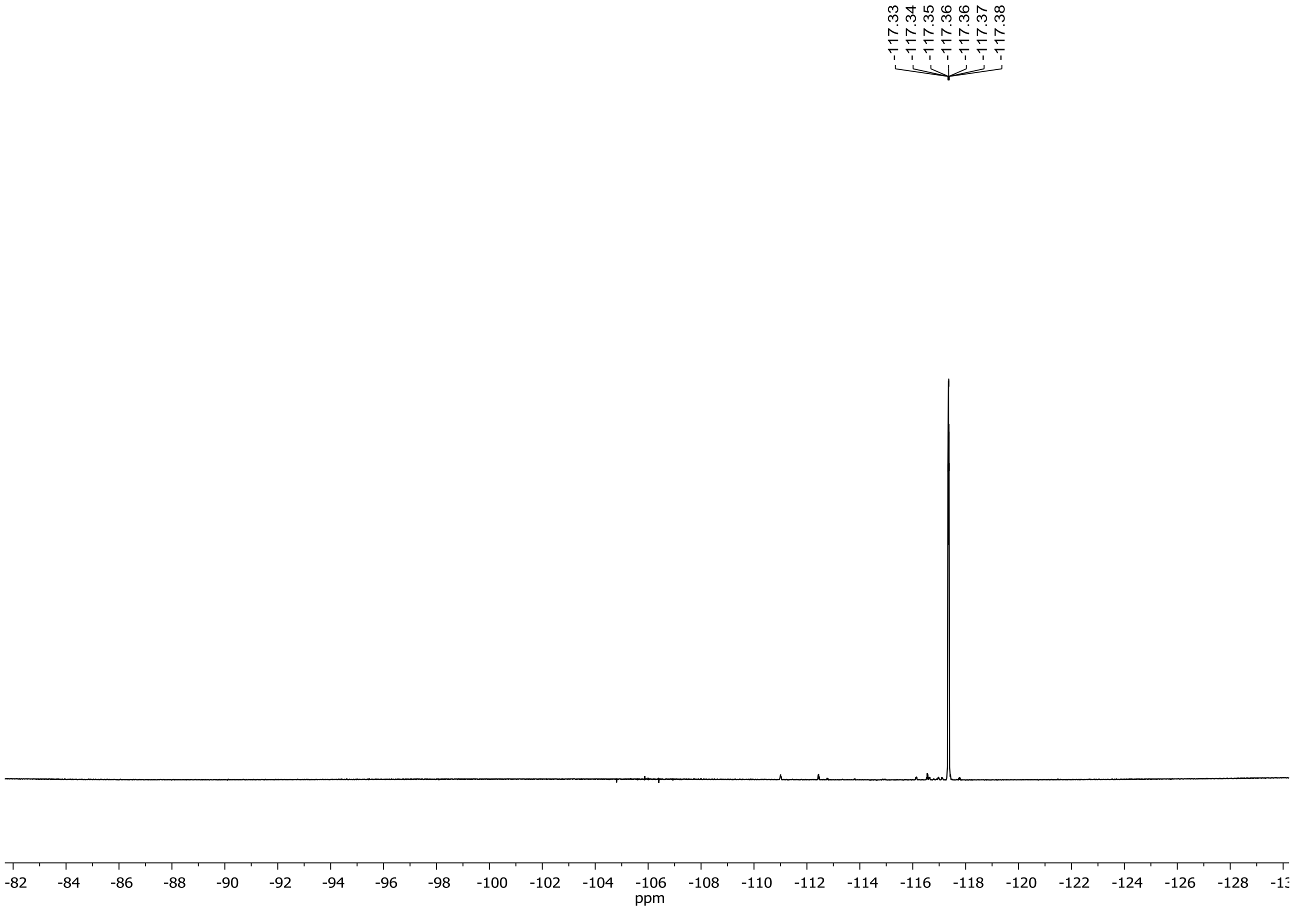

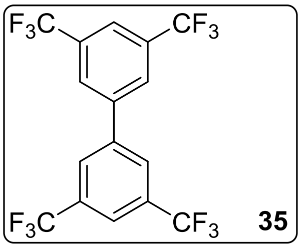

35

^{1}H-NMR (300 MHz, $CDCl_3$)

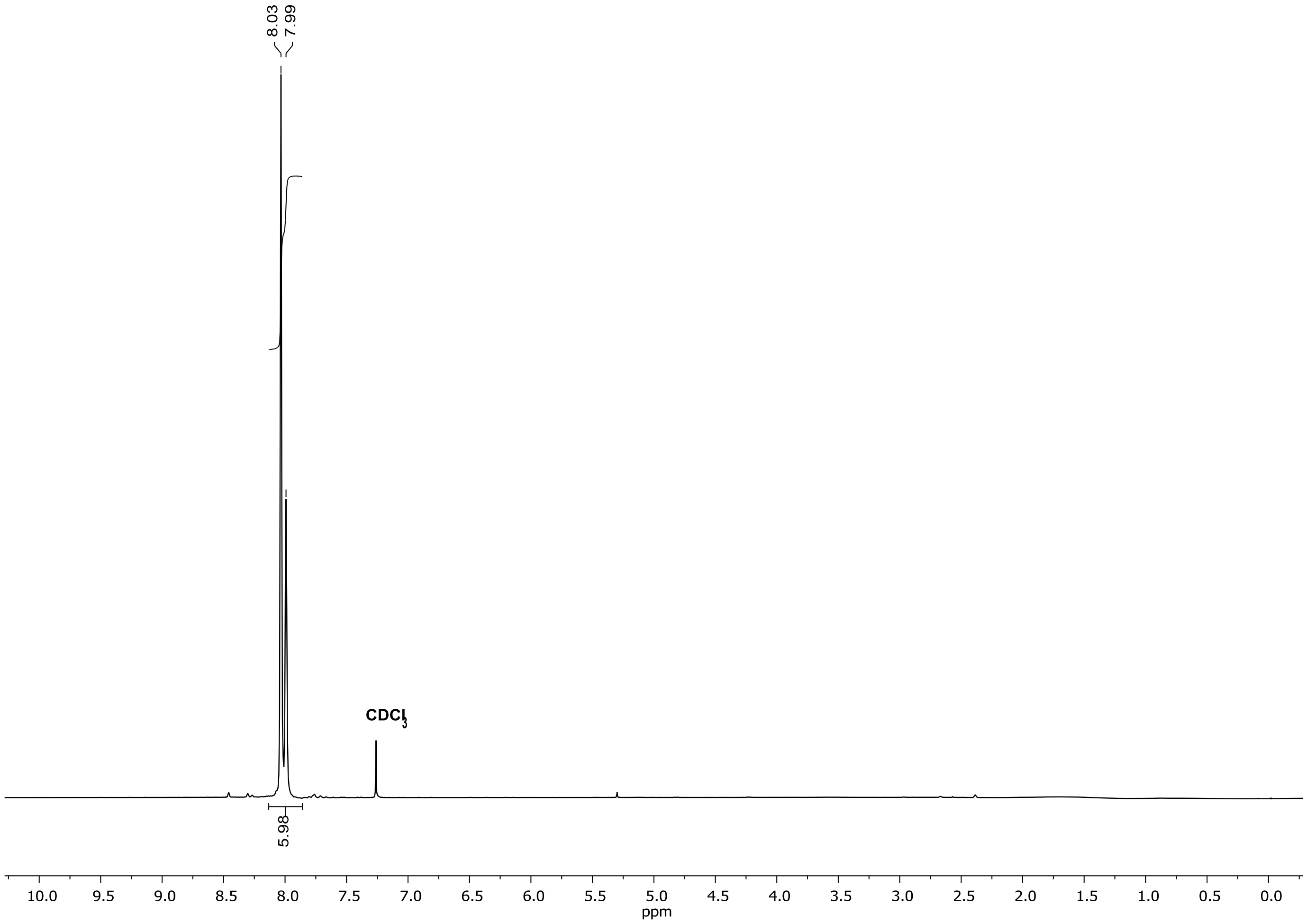

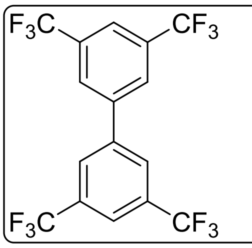

35 $^{13}C\{^{1}H\}$-NMR (75 MHz, $CDCl_3$)

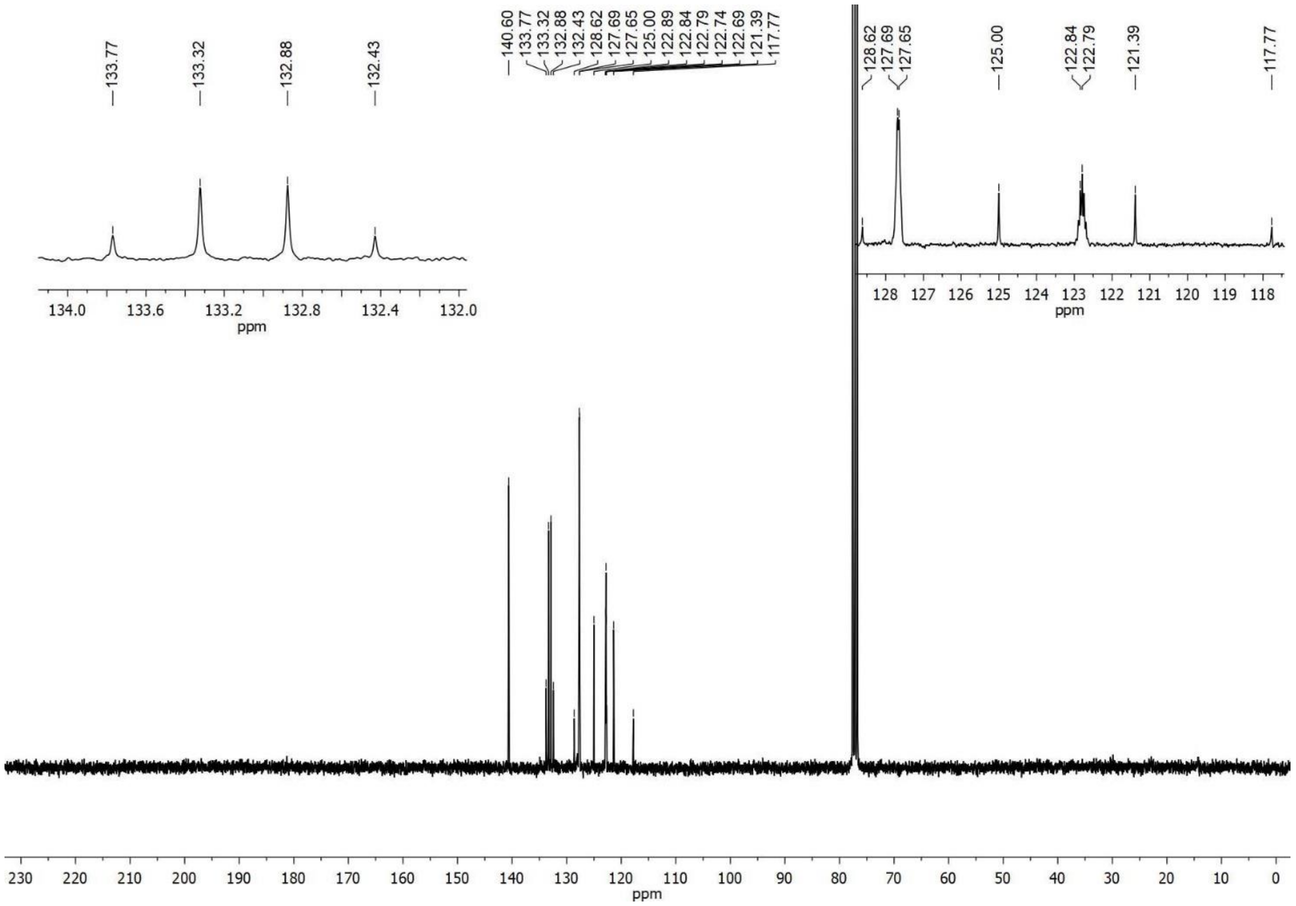

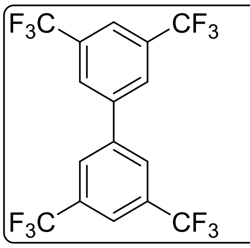

35

^{19}F-NMR (376 MHz, $CDCl_3$)

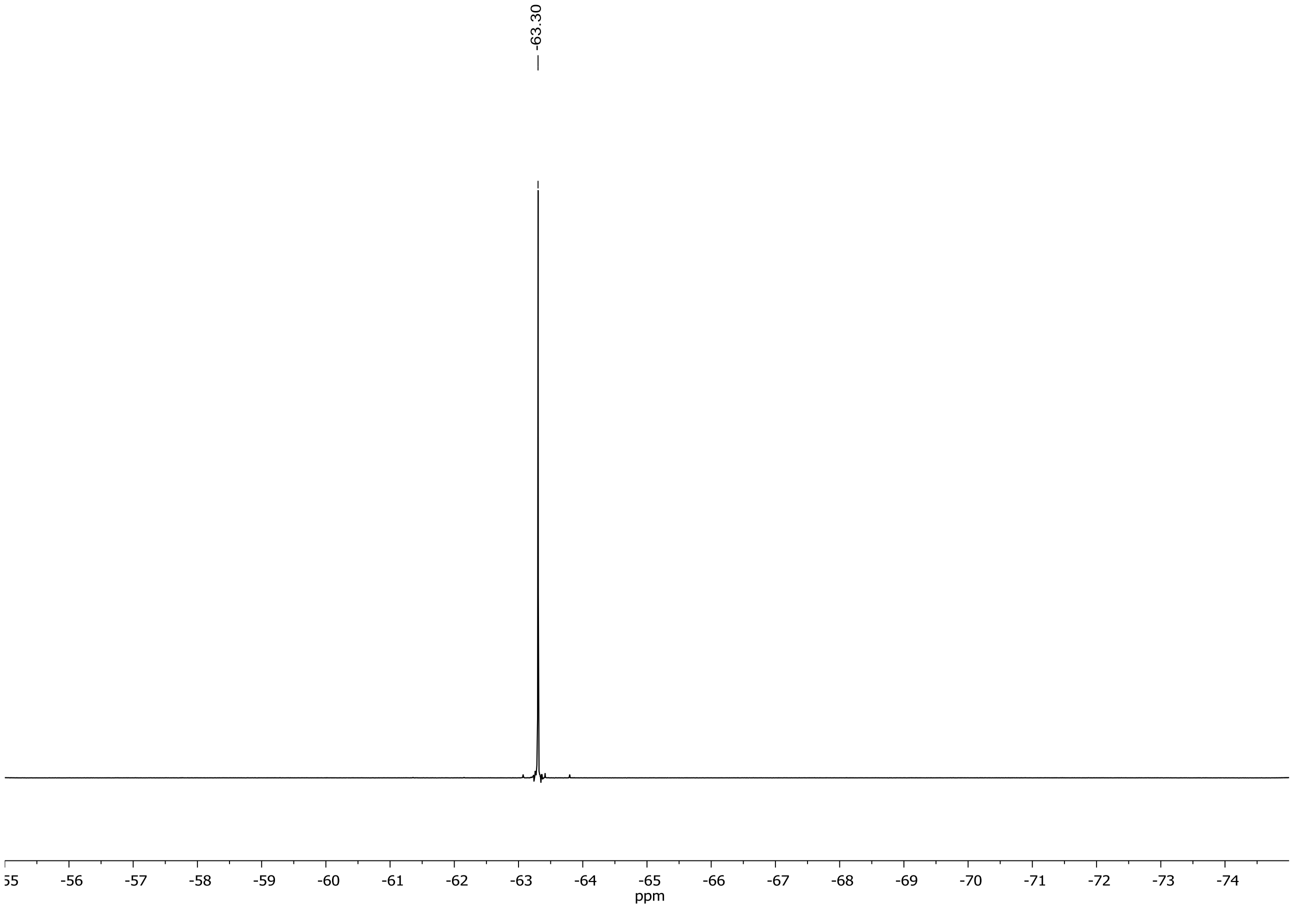

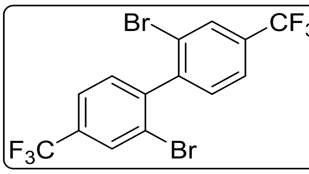

36

^{1}H-NMR (300 MHz, $CDCl_3$)

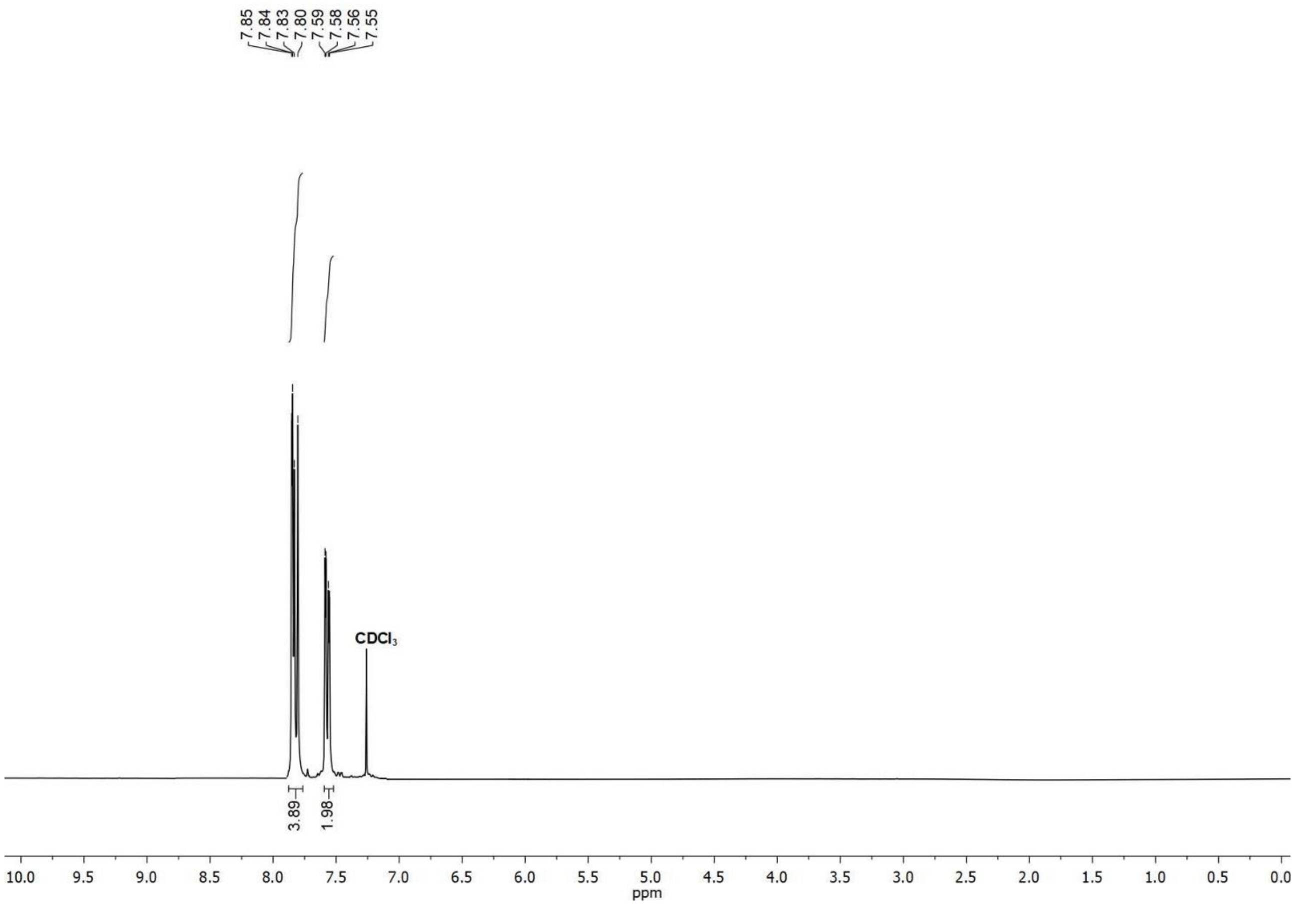

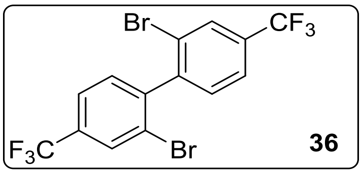

$^{13}C\{^{1}H\}$-NMR (75 MHz, $CDCl_3$)

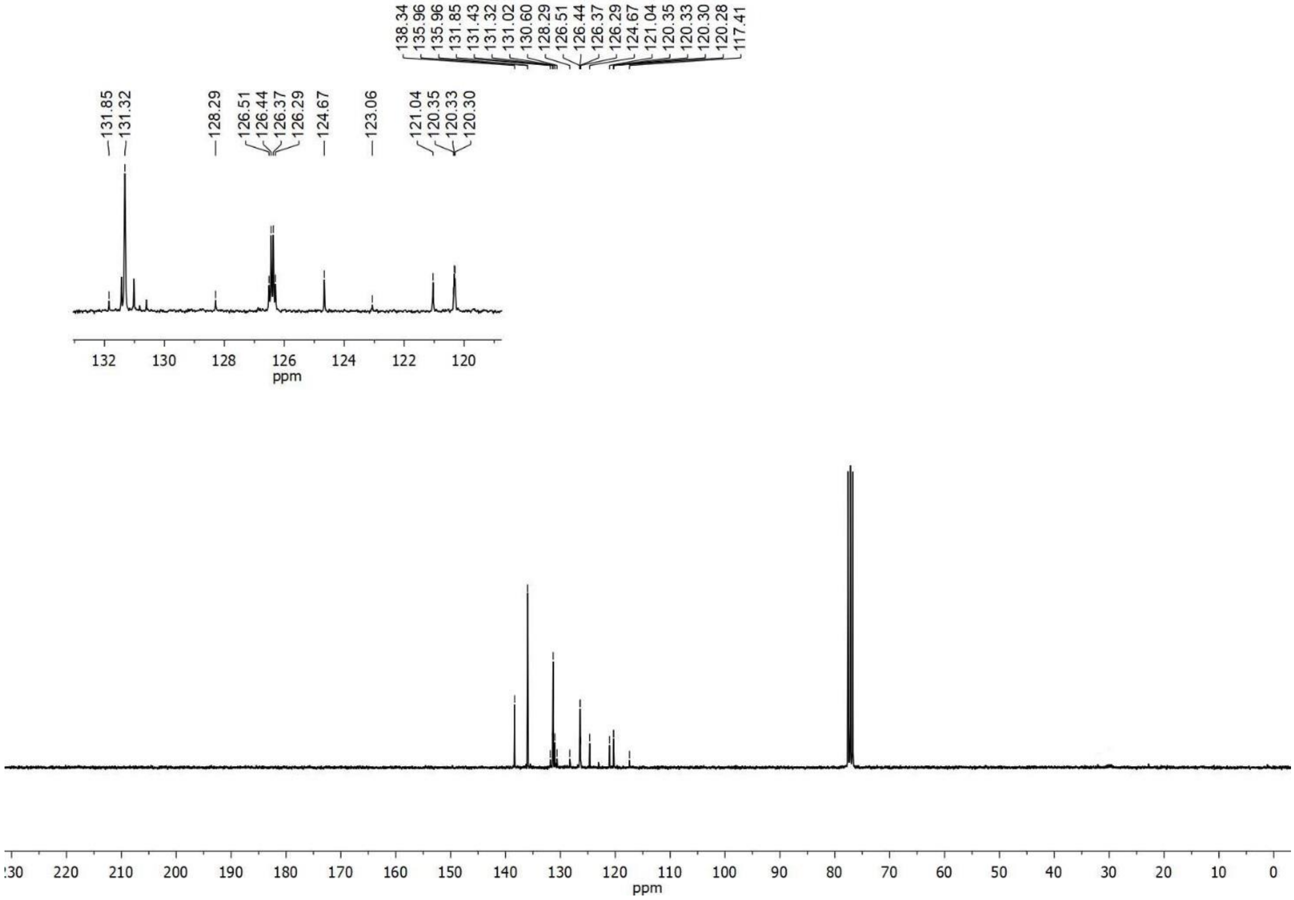

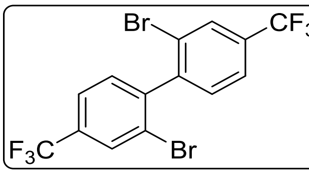

36

^{19}F-NMR (376 MHz, $CDCl_3$)

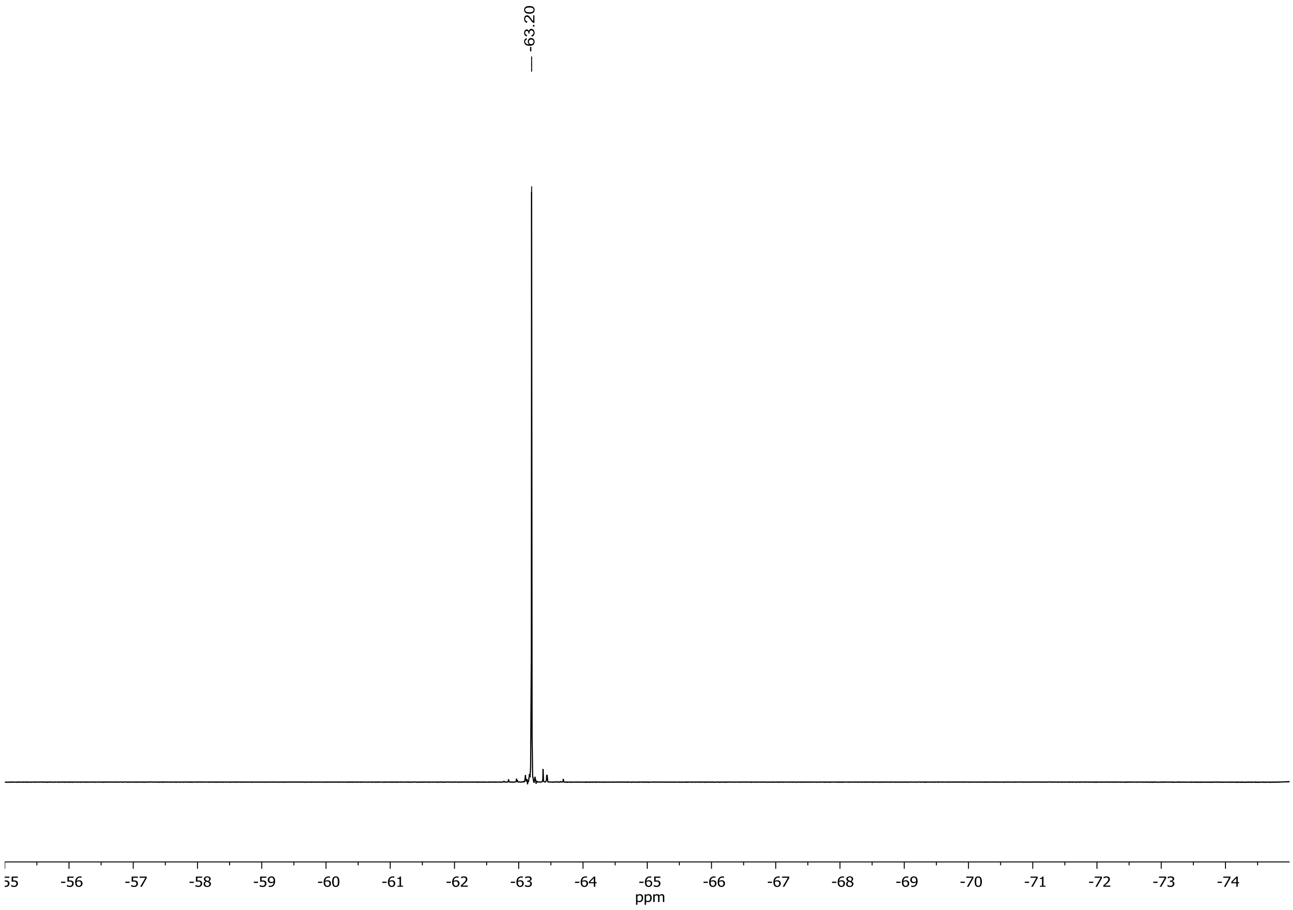

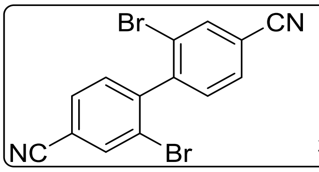

37 ^{1}H-NMR (300 MHz, $CDCl_3$)

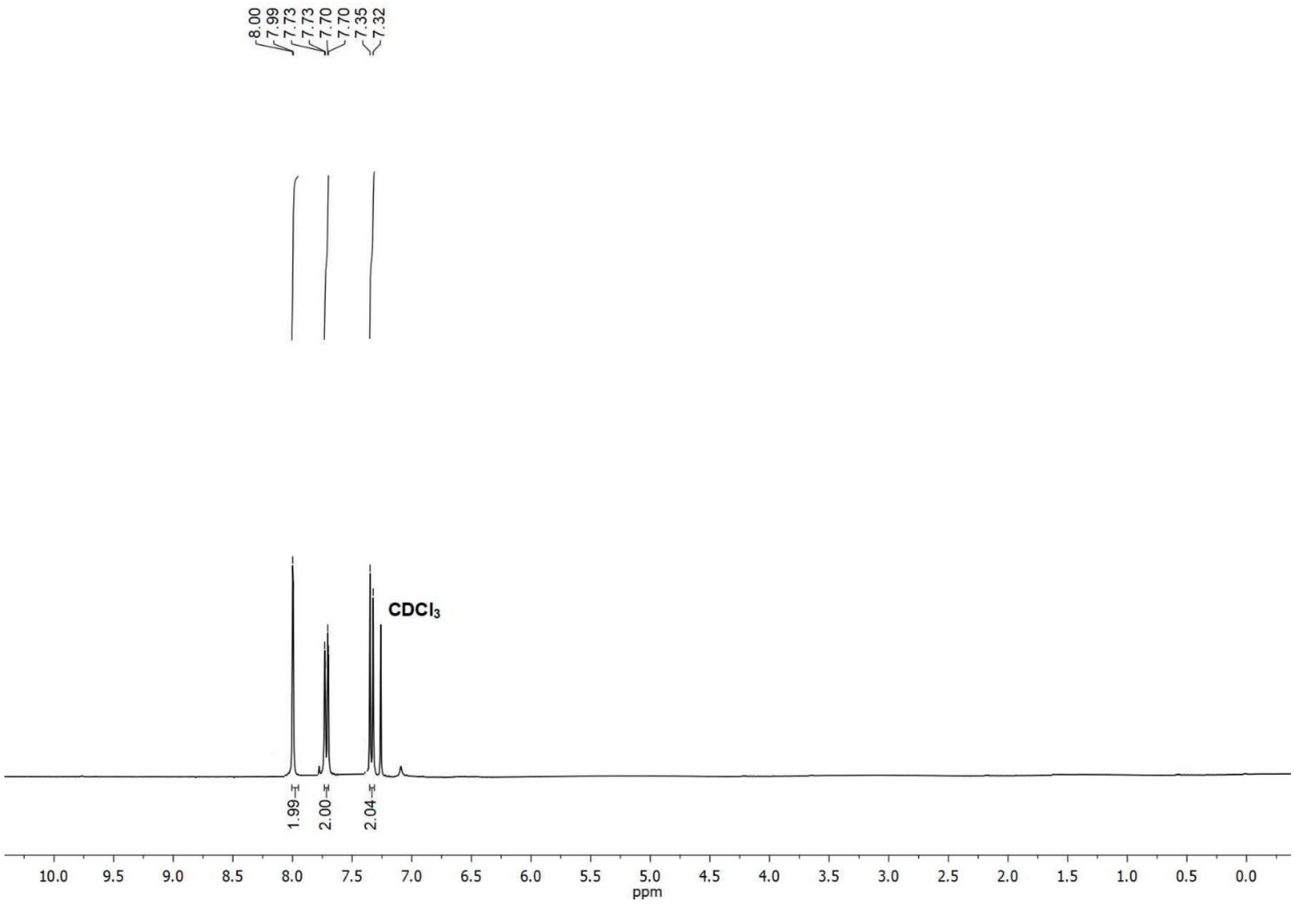

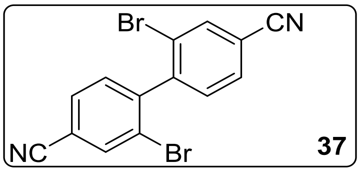

$^{13}C\{^{1}H\}$-NMR (75 MHz, $CDCl_3$)

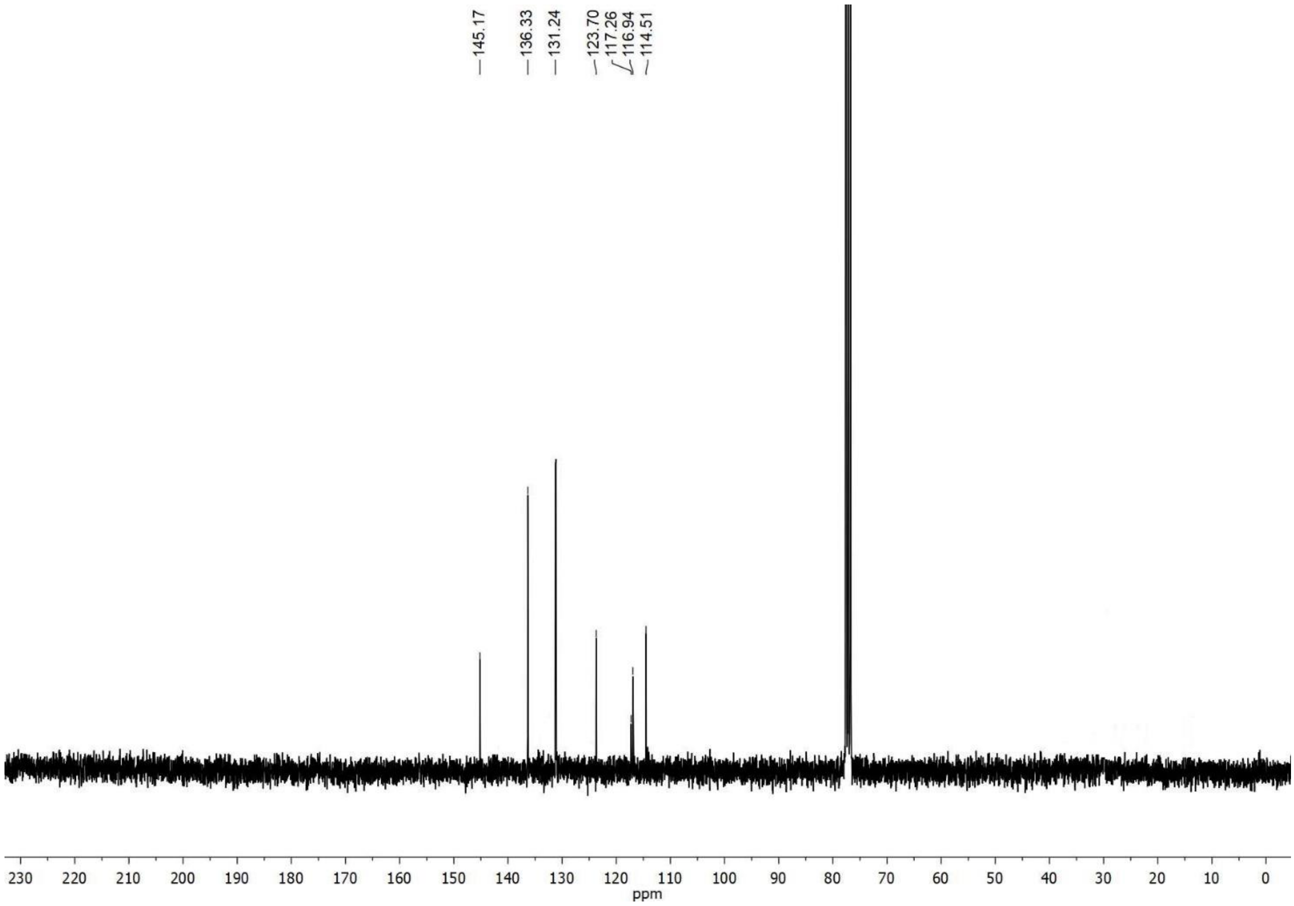

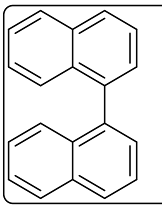

39 ^{1}H-NMR (300 MHz, $CDCl_3$)

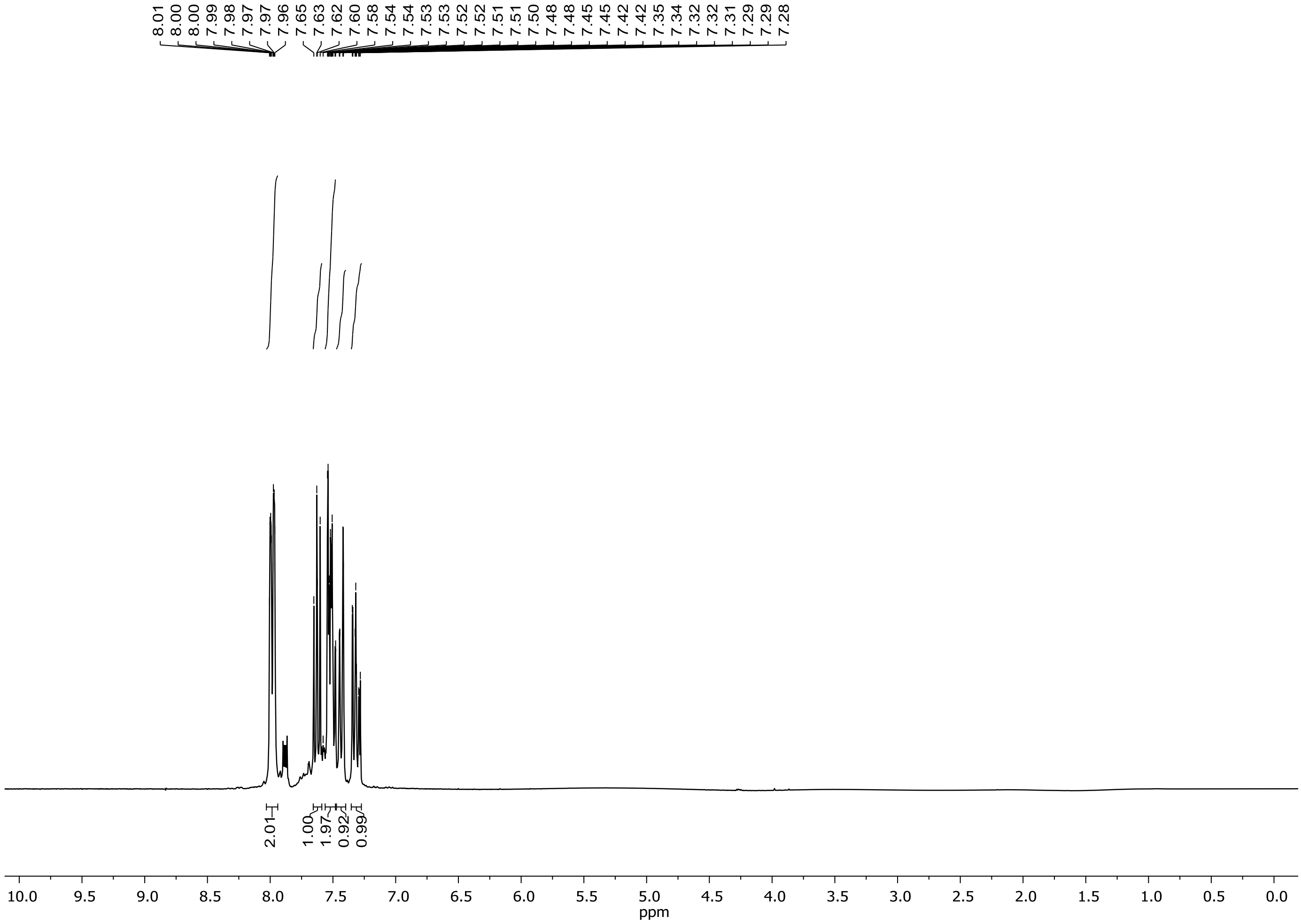

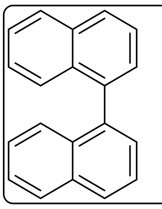

39

$^{13}C\{^{1}H\}$-NMR (75 MHz, $CDCl_3$)

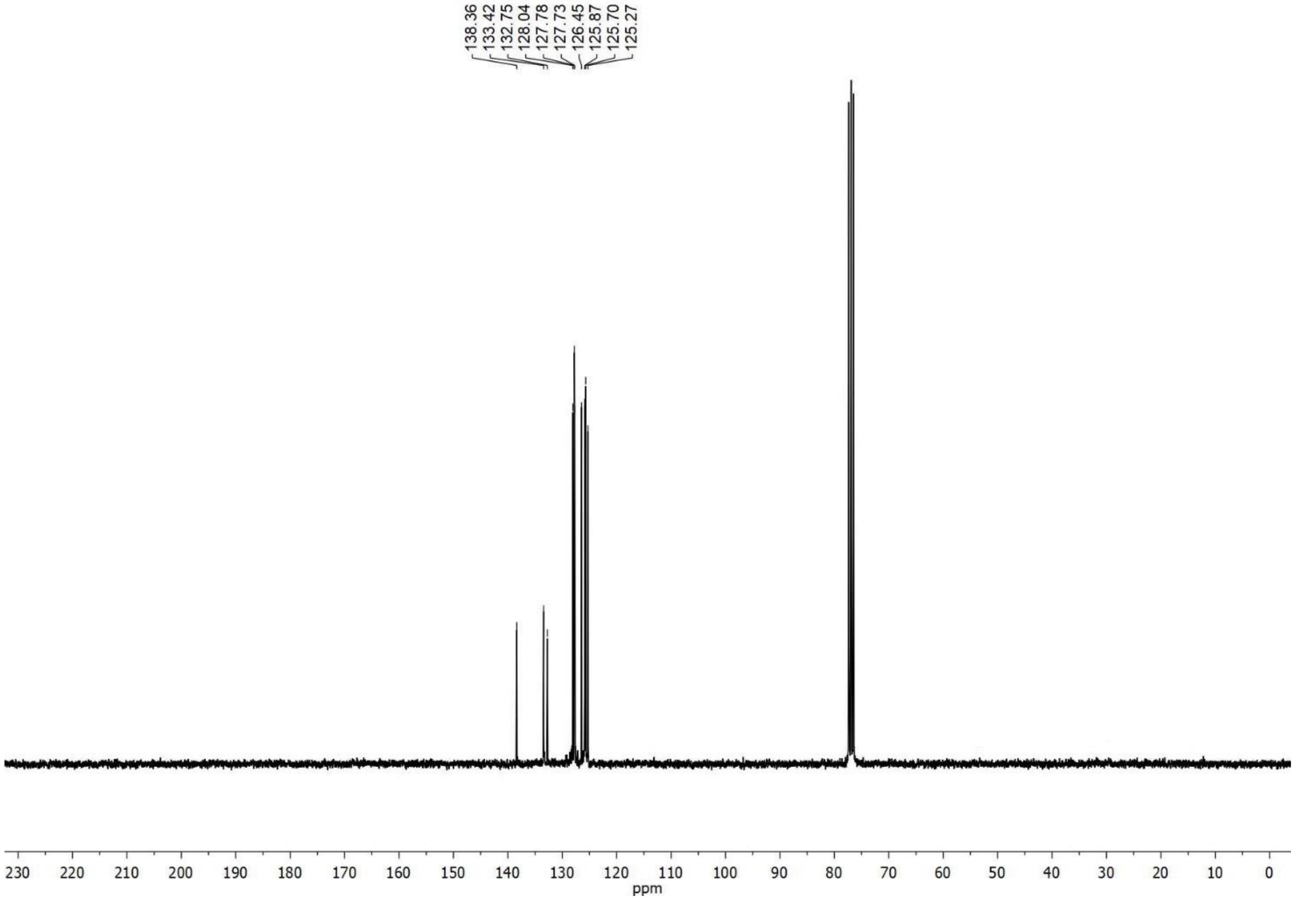

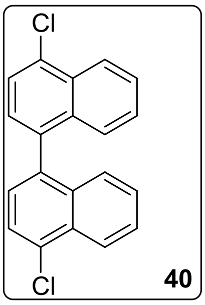

40

^{1}H-NMR (300 MHz, $CDCl_3$)

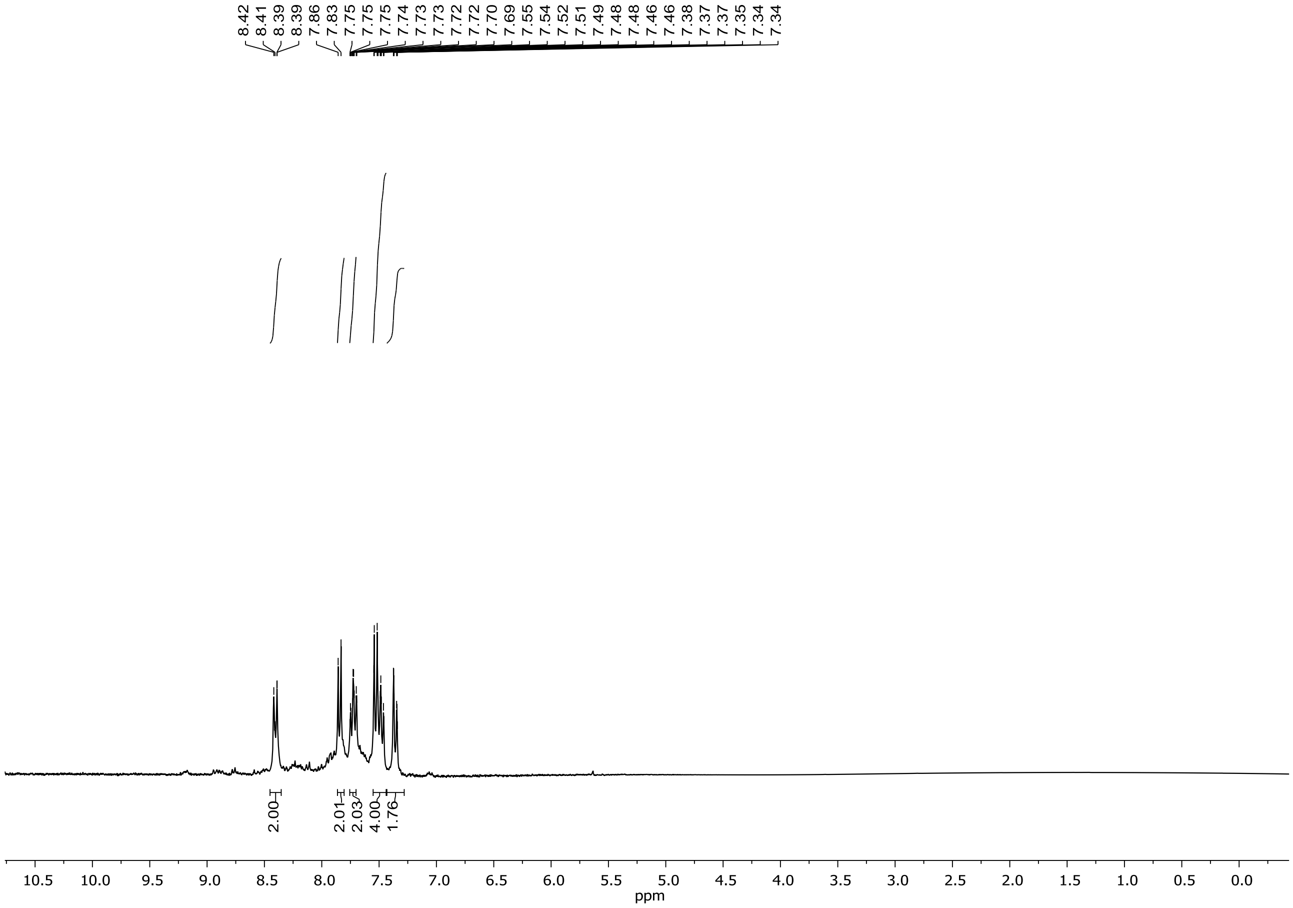

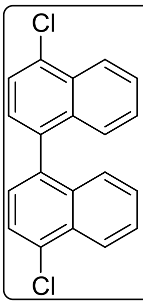

40 $^{13}C\{^{1}H\}$-NMR (75 MHz, $CDCl_3$)

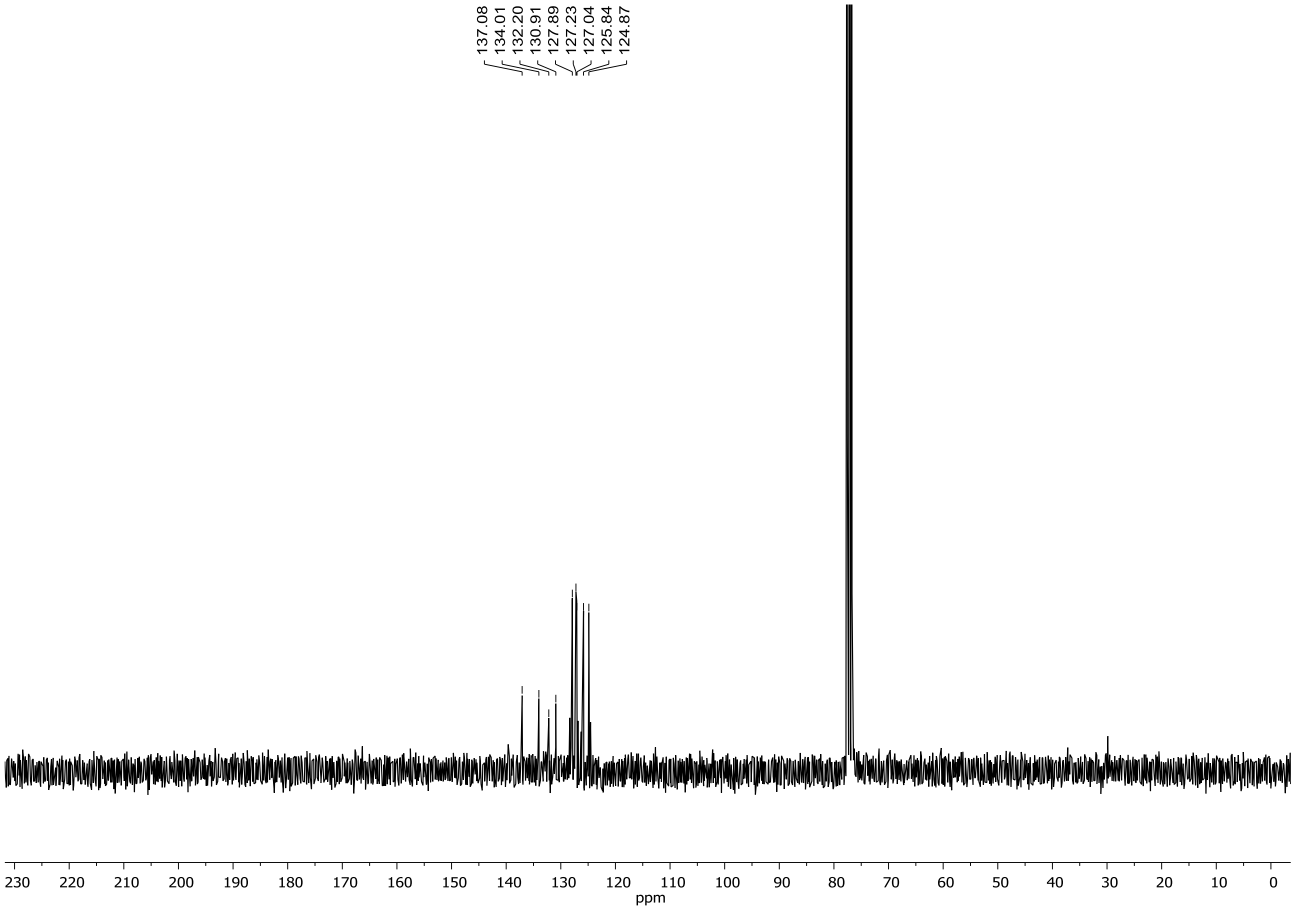

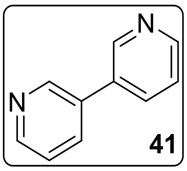

41

^{1}H-NMR (300 MHz, $CDCl_3$)

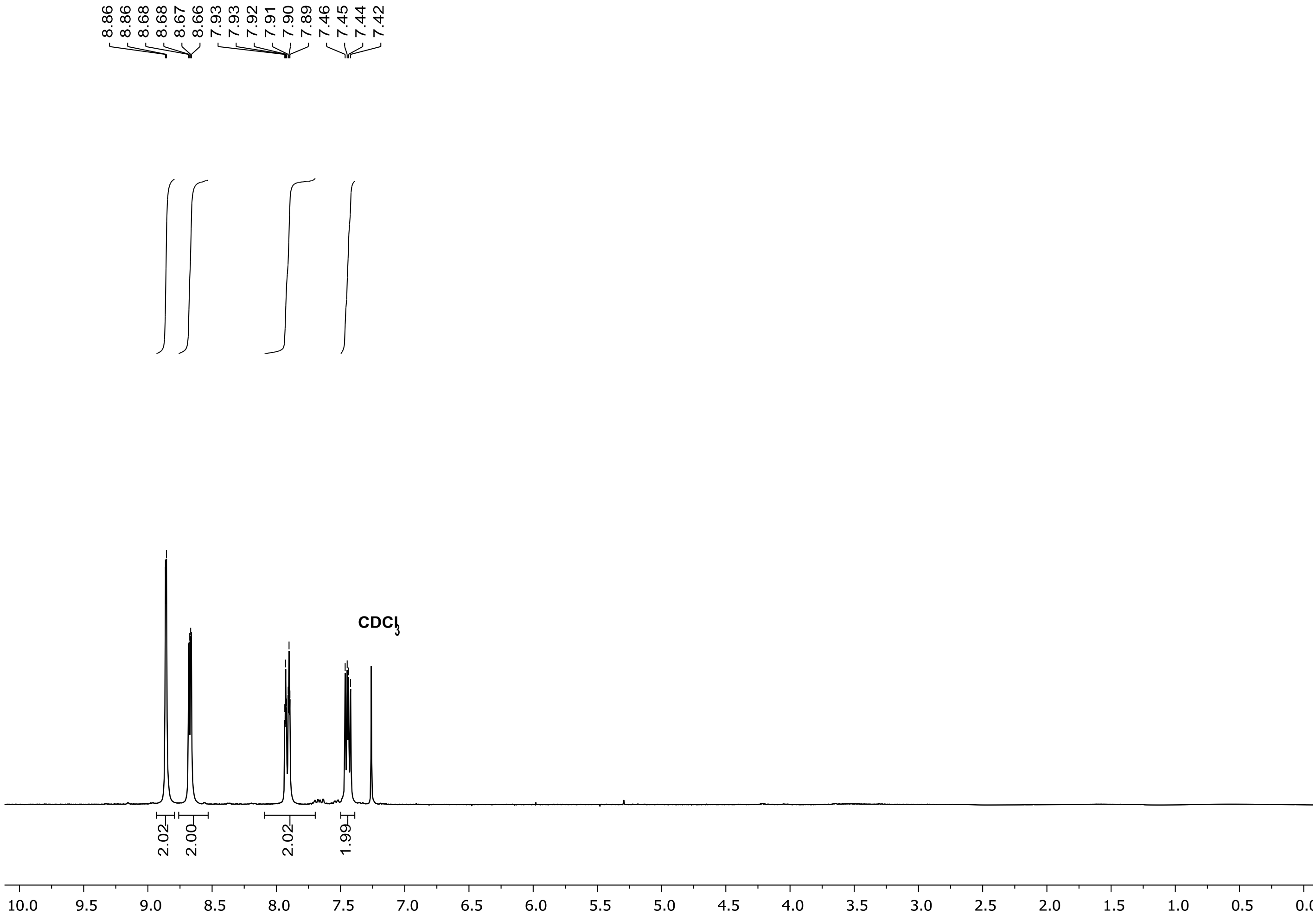

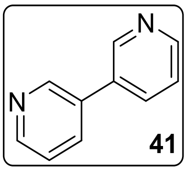

41

$^{13}C\{^{1}H\}$-NMR (75 MHz, $CDCl_3$)

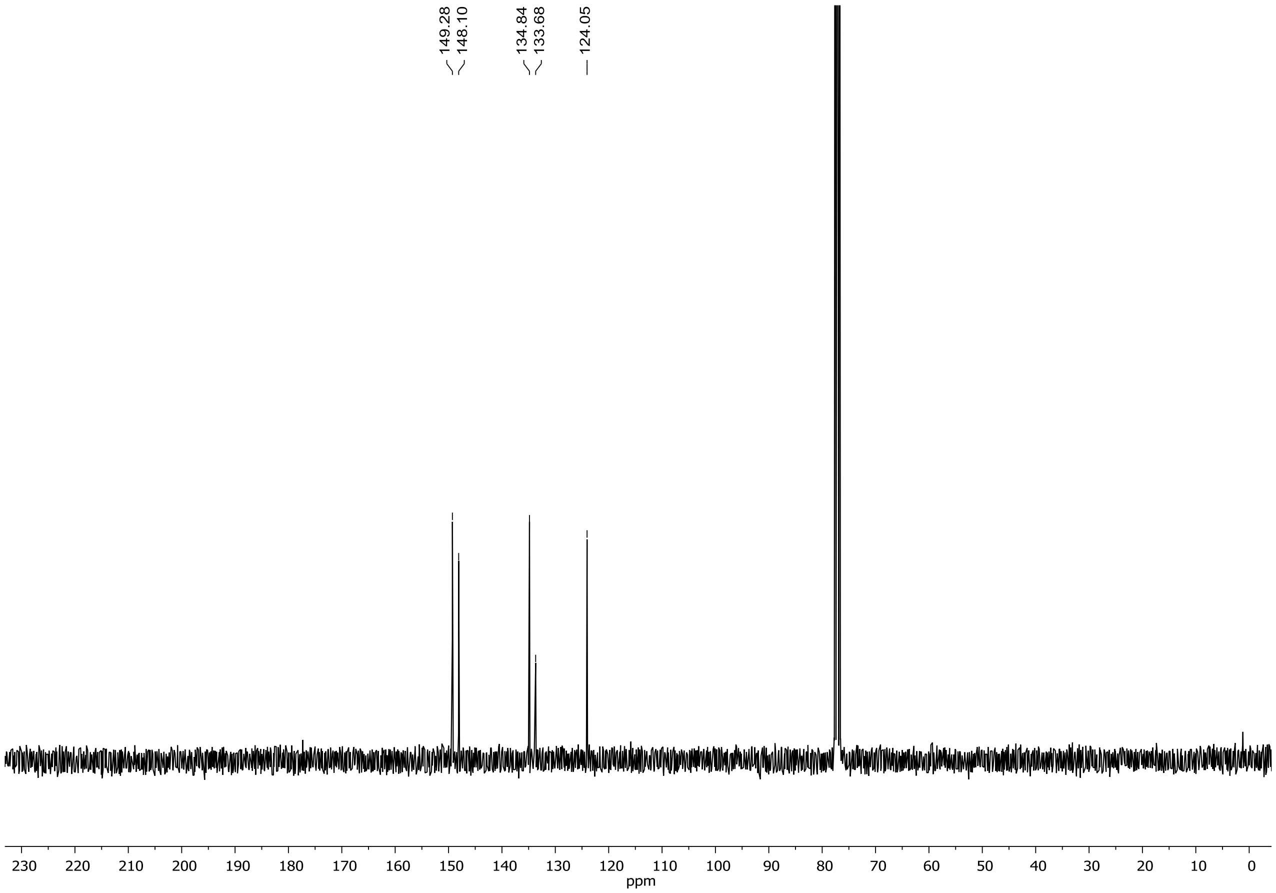

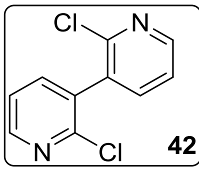

42 ^{1}H-NMR (300 MHz, $CDCl_3$)

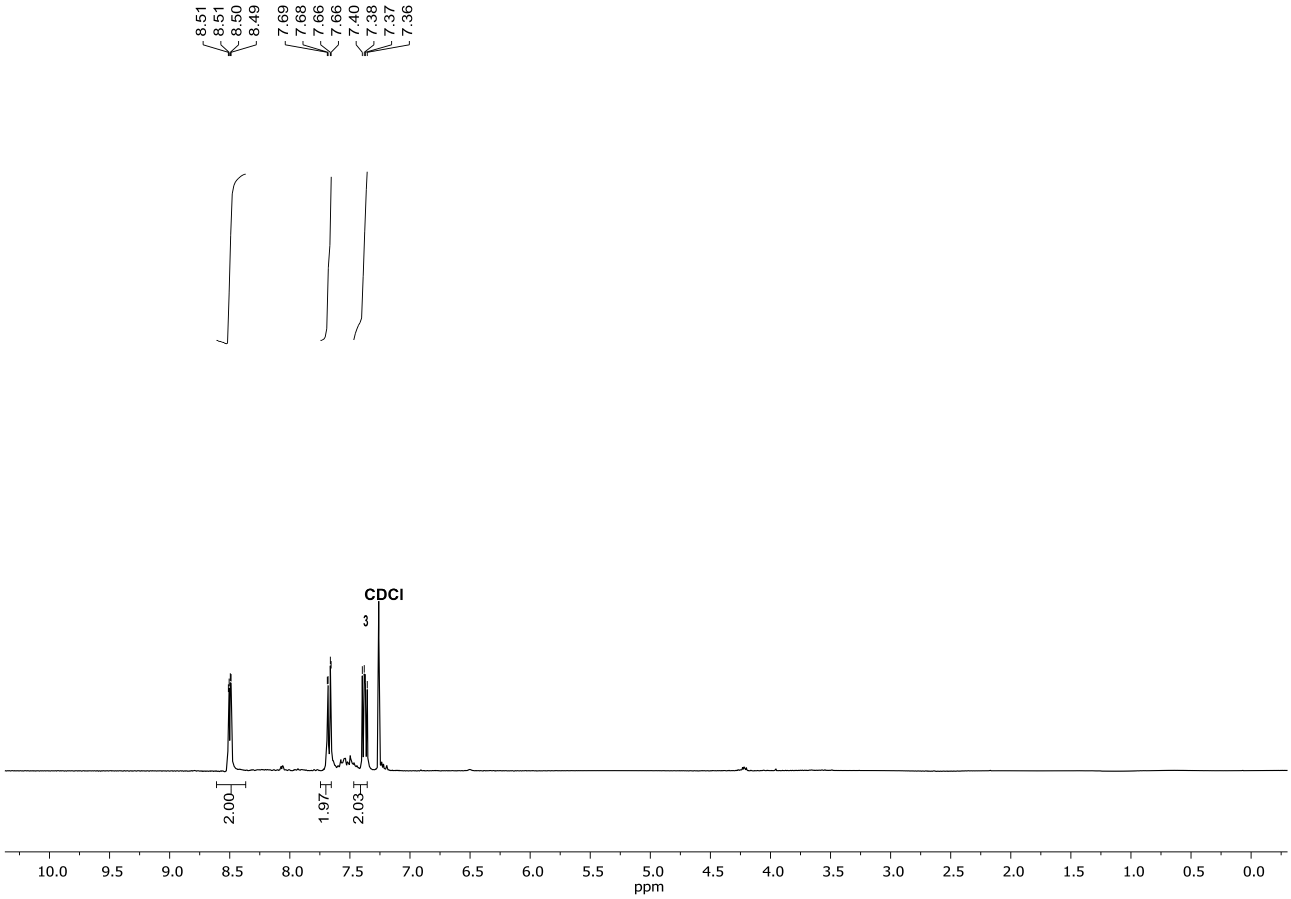

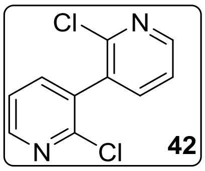

42 $^{13}C\{^1H\}$-NMR (75 MHz, $CDCl_3$)

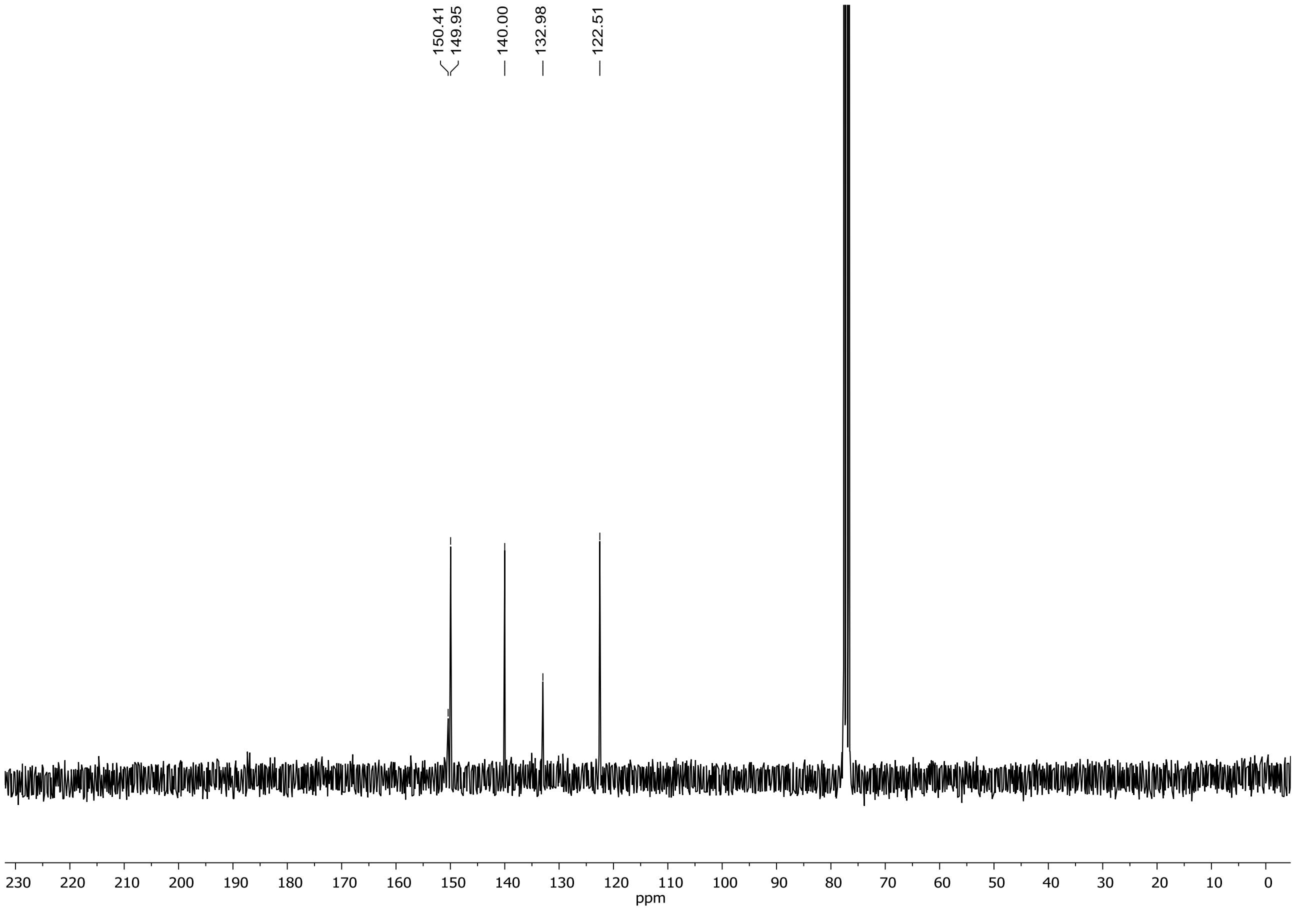

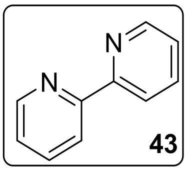

43

^{1}H-NMR (300 MHz, $CDCl_3$)

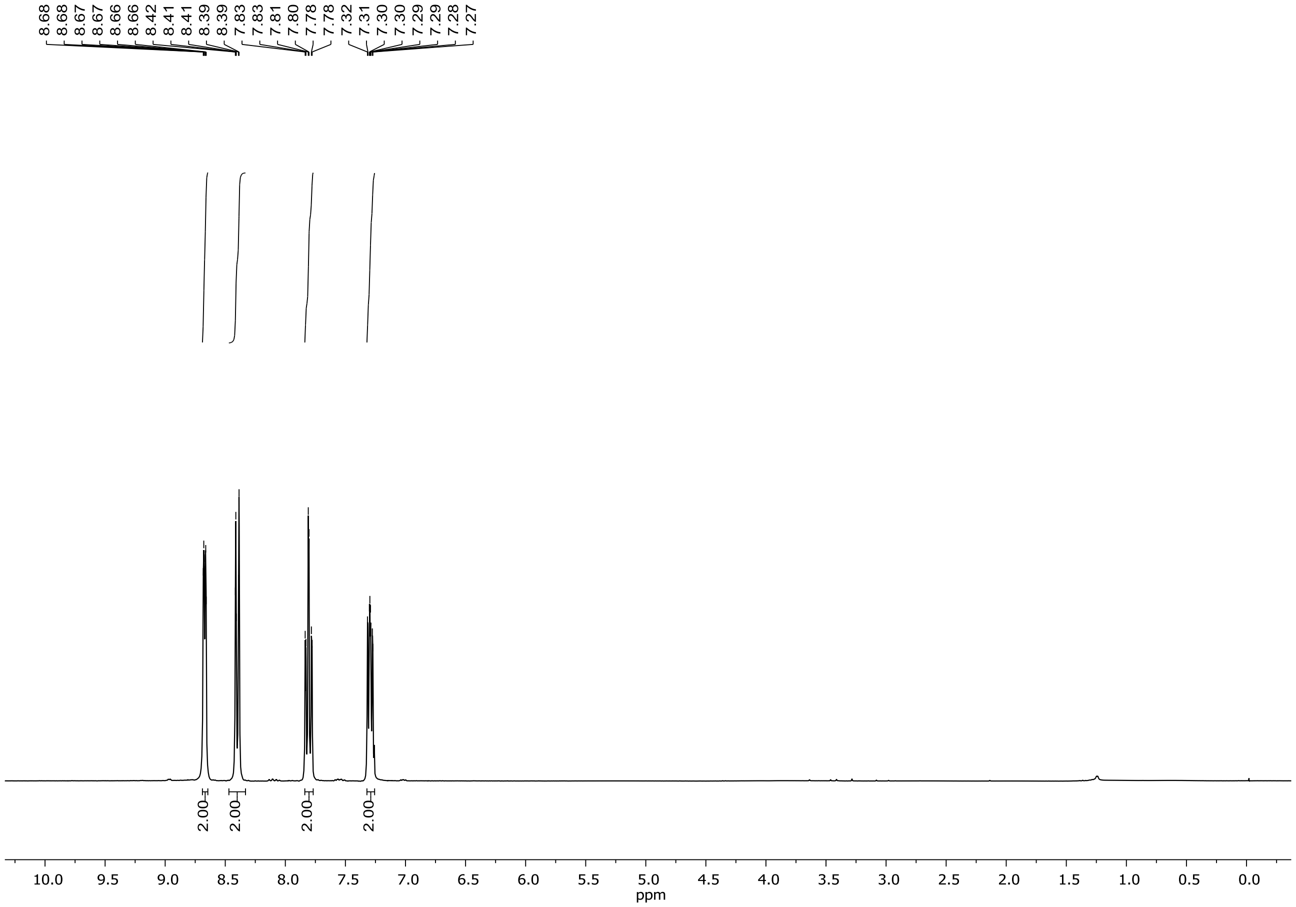

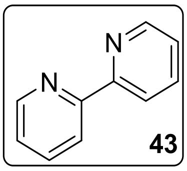

43

$^{13}C\{^{1}H\}$-NMR (75 MHz, $CDCl_3$)

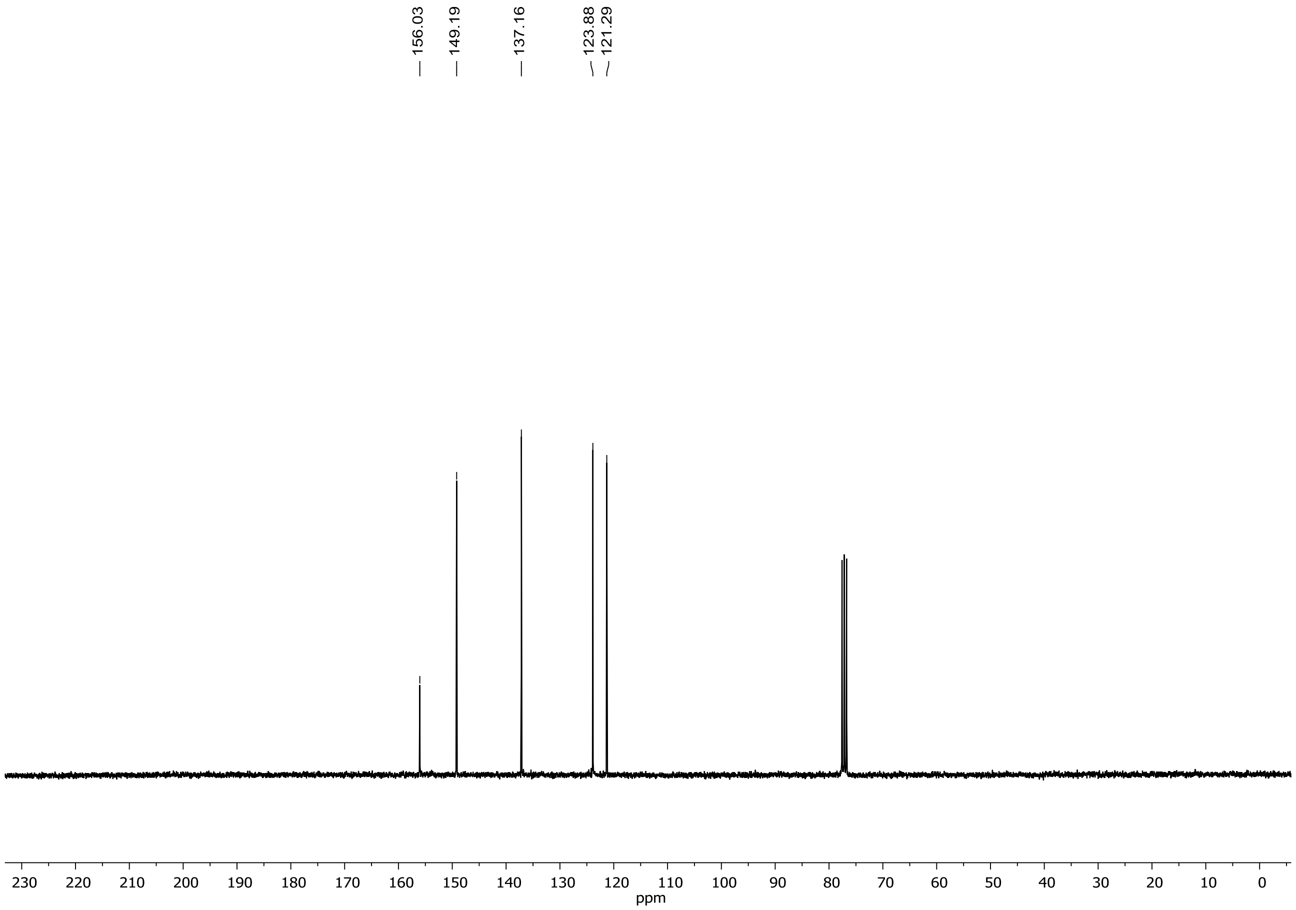

Supplement: Supplementary file 1 — jo2c00225_si_001.pdf [file jo2c00225_si_001.pdf]
